# Computational Analysis of Enantioselective Pd-Catalyzed α-Arylation of Ketones

**DOI:** 10.1021/acs.joc.0c01768

**Published:** 2020-07-31

**Authors:** Manuel Orlandi, Giulia Licini

**Affiliations:** † Department of Chemical Sciences, University of Padova, via Marzolo 1, 35131 Padova, Italy; ‡ CIRCC—Consorzio Interuniversitario per le Reattività Chimiche e la Catalisi, Padova Unit., via Marzolo 1, 35131 Padova, Italy

## Abstract

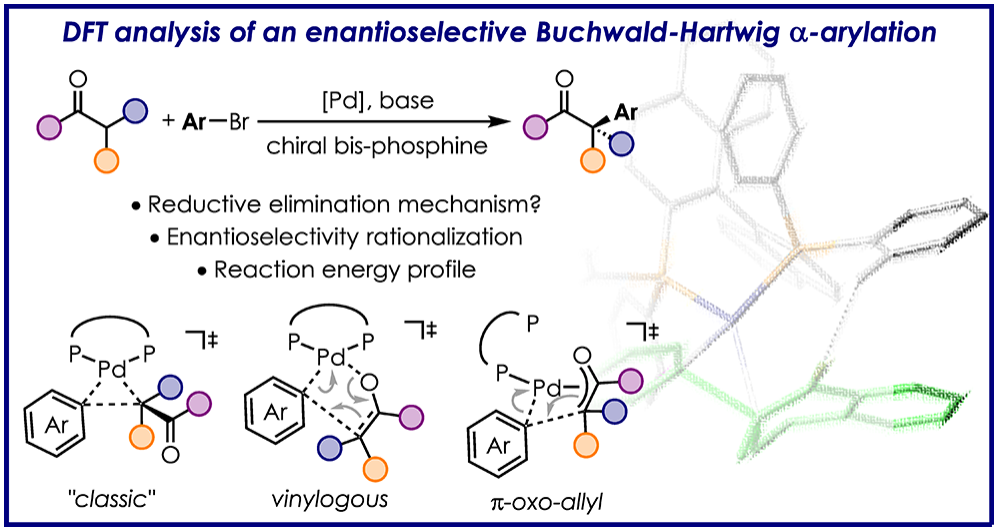

The direct α-arylation of carbonyl compounds emerged over the last two decades as a straightforward method for the formation of C(sp^3^)–C(sp^2^) bonds. Mechanistic studies suggested a classical cross-coupling catalytic cycle. This consists of oxidative addition of the aryl halide (ArX) to the Pd(0)-catalyst, transmetallation of the Na- or K-enolate generated *in situ*, and subsequent reductive elimination. Even though the general reaction mechanism was thoroughly investigated, studies focusing on enantioselective variants of this transformation are rare. Here, the computational study of the [Pd(BINAP)]-catalyzed α-arylation of 2-methyltetralone with bromobenzene is reported. The whole reaction energy profile was computed and several mechanistic scenarios were investigated for the key steps of the reaction, which are the enolate transmetallation and the C–C bond-forming reductive elimination. Among the computed mechanisms, the reductive elimination from the C-bound enolate Pd complex was found to be the most favorable one, providing a good match with the stereoselectivity observed experimentally with different ligands and substrates. Detailed analysis of the stereodetermining transition structures allowed us to establish the origin of the reaction enantioselectivity.

## Introduction

**Figure 1:**
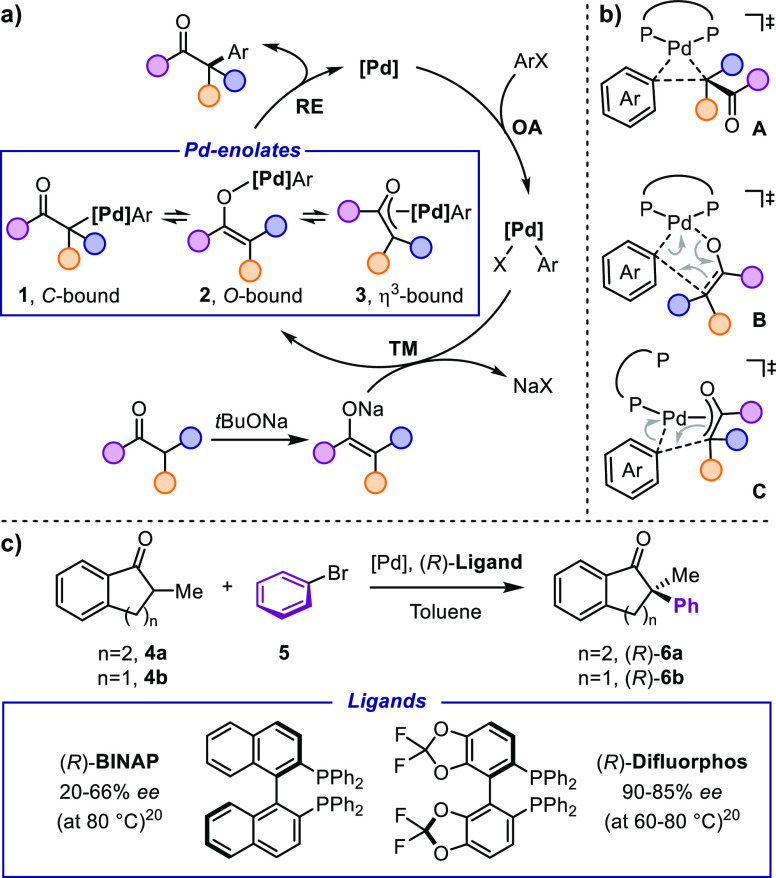
(a) Catalytic cycle of the α-arylation of carbonyl compounds. (b) Possible pathways for the reductive elimination step. (c) Benchmark reaction investigated in this study.

The first examples of direct, Pd-catalyzed α-arylation of carbonyl compounds with aryl halides^1,2^ were first reported by the groups of Buchwald and Hartwig in 1997.^3,4^ Great advances have been made in the field since then. Nowadays, carbonyl compounds ranging from ketones, aldehydes, esters, amides, and nitriles can be coupled with a plethora of aryl halides or pseudohalides with great efficiency.^1,2^ Hartwig and co-workers investigated the reaction mechanism in detail and showed that it follows the cross-coupling catalytic cycle depicted in Figure 1a.^3,5−8^ The reaction begins with the oxidative addition (**OA**) of the aryl halide ArX to the Pd(0)-catalyst. The resulting Ar-Pd-X species undergoes transmetallation (**TM**) with the Na- or K-enolate generated *in situ* by a suitable base (typically *t*BuONa or KHMDS). The enolate ligand can bind to the Pd center in three different modes,^5−13^ the C-bound enolate **1** being electronically more favored.^5−8^ The aryl Pd-enolate connectivity is highly dependent on sterics, and the O-bound enolate **2** becomes more favored as the hindrance of the ligand and the number of α substituents of the enolate increase.^5−8^ Finally, η^3^-bound enolates **3** (oxo-π-allylic enolates) could form upon dissociation of one of the Pd ancillary ligands (Figure 1a).^9,14^ One of the key steps of the reaction is the C–C bond-forming reductive elimination (**RE**), which could occur via several pathways depicted in Figure 1b. Pathway **A** is the direct reductive elimination between the aryl C(sp^2^) and the alkyl C(sp^3^) of the C-bound enolate **1**. **B** is the vinylogous reductive elimination from the O-bound enolate **2**,^15,16^ with the C–C bond forming between the aryl C(sp^2^) and the alkenyl C(sp^2^). Pathway **C** is similar to mechanism **A** yet occurring from **3** with the enolate bound in a η^3^ mode. Determining experimentally the actual reductive elimination mechanism is not trivial as pathways **A**, **B**, and **C**(14) could all take place starting from each one of the possible enolates **1–3** by tautomerization from the most stable to the most reactive intermediate prior to the **RE** step.

Despite the limited knowledge about the key C–C bond-forming step in this transformation, stereoselective variants were also developed. These allow the construction of benzylic stereocenters in the α-position with respect to a carbonyl moiety, supposedly via a stereodetermining **RE** step.^17−33^ However, in spite of these achievements, this research area still suffers many limitations.^34^ In this regard, the computational study of this reaction could greatly improve our understanding of its general mechanism by evaluation of the energetics of each possible pathway **A–C** (Figure 1b). Visualization and analysis of the diastereomeric transition structures (TSs) leading to different enantiomeric products could help in understanding the interactions responsible for the observed selectivity. This would improve the further development of catalytic systems for these transformations.^34−37^

Computational studies of enantioselective α-arylation reactions are rare.^38^ The first example was reported by Yamamoto and co-workers in 2011, where the authors describe Pd/Josiphos-catalyzed arylation of silyl ketene acetals in good yields and selectivities.^29^ The authors proposed a cyclic **TM** TS in which the Si atom of the silyl enol ether is attacked by an acetate ligand with concomitant formation of the O–Pd bond (O-bound enolate **B**, Figure 1b). Based on saturation of the Pd coordination sphere, the authors excluded the tautomerization from the O- to the C-bound enolate. Hence, they computed the TSs for the **RE** via the vinylogous mechanism **B**, which was found to proceed with an energy barrier of 27 kcal/mol. More recently, the Zhou group reported enantioselective Pd-catalyzed arylation of vinyl acetates^30^ and silyl ketene acetals^28,31^ using a BINOL-based monophosphine ligand. The authors found that the most favorable **TM** pathway involved coordination of the nucleophile C=C bond by a cationic Pd-complex, with subsequent outer sphere attack of an acetate anion at the Si atom. This would directly lead to the formation of a C-bound Pd-enolate. **RE** would then follow with low activation barrier (13–16 kcal/mol) via the general mechanism **A** (Figure 1b).^30,31^ Notably, based on the weak noncovalent interactions (NCIs) present at the TS level, the authors were able to design improved versions of their ligand, leading to the improvement of the catalytic performance. These studies provide insights into the reaction mechanism of the arylation of silyl ketene acetals to give arylated products bearing tertiary stereocenters. However, because of postreaction racemization, these reactions are run under mild conditions, which strongly differ from those of the more widespread enantioselective direct arylation of carbonyl compounds (*vide infra*). Na- or K-enolates are expected to undergo **TM** via a different mechanism with respect to the one showed with Si-enolates by Zhou et al. Moreover, the relative stability of the different tautomers of Pd-enolates is dependent on the number of α-substituents of the nucleophile.^5−8^ As α-disubstituted carbonyl compounds favor the O-bound tautomer **2**, one might hypothesize this preference to be translated at the TS level differently from previously studied systems.

In order to gain more insights into the reaction mechanism of the enantioselective Pd-catalyzed α-arylation of carbonyl compounds, we performed a DFT study of the [Pd((*R*)-**BINAP**)] catalyzed coupling between 2-methyltetralone **4a** and bromobenzene **5** to give **6a** (Figure 1c).^17^ The arylation of tetralones and indanones is a well-established transformation that is often used as a benchmark reaction for testing new catalytic systems.^34^ Therefore, experimental data are available for the validation of the resulting stereochemical model. For this purpose, the reaction enantioselectivity was also computed for a different ligand and substrate: (*R*)-**Difluorphos** and 2-methylindanone **4b** (Figure 1c).^20^

## Results and Discussion

### General Reaction Mechanism

**Figure 2:**
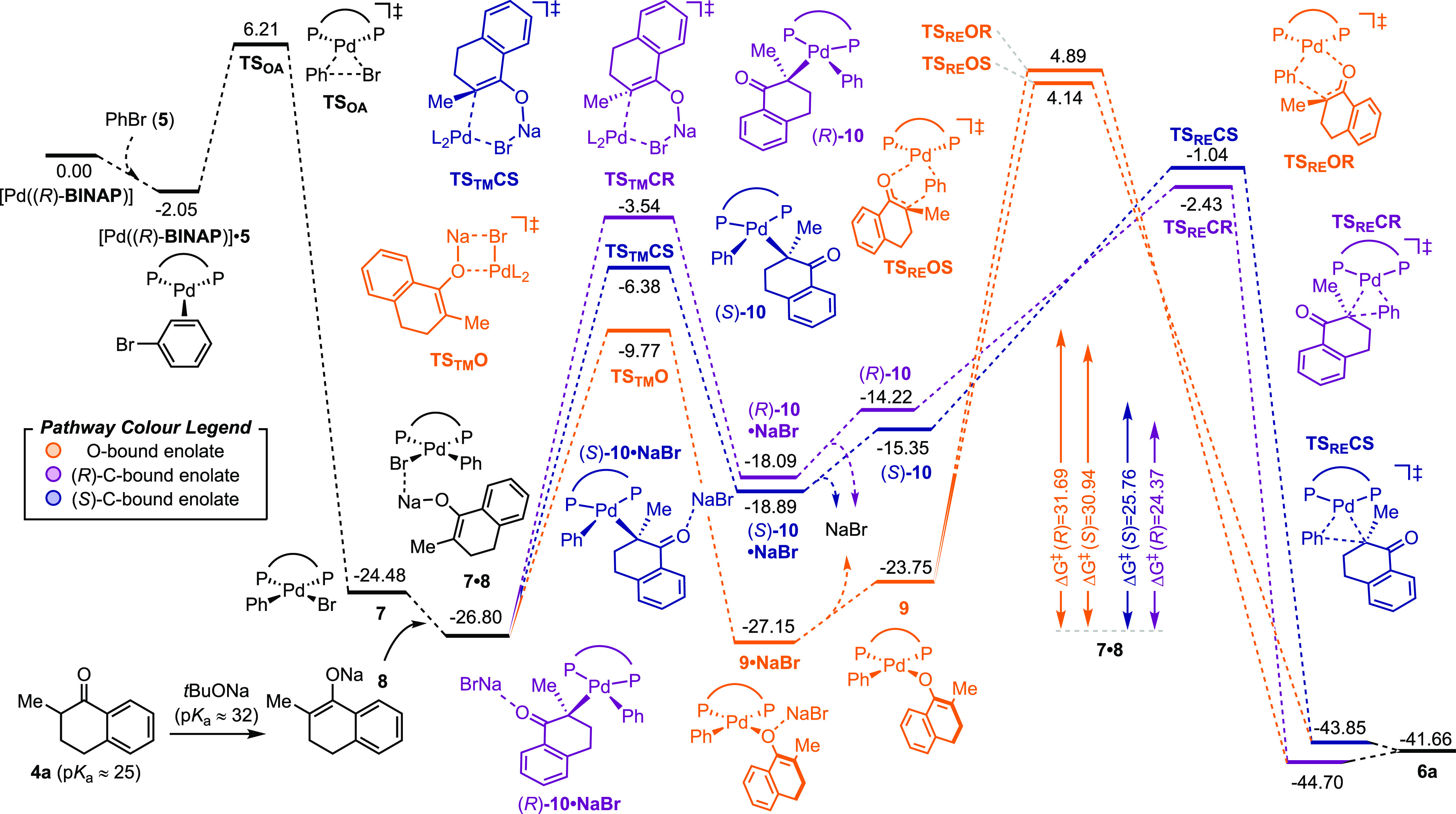
Reaction Gibbs free energy profile (kcal/mol) of the enantioselective α-phenylation of 2-methyltetralone **4a** catalyzed by [Pd((*R*)-**BINAP**)] at the [CPCM = toluene]PBE/SDD:6-311+G(d,p)//PBE/lanl2dz:6-31G(d) level of theory. Different reaction pathways are highlighted in different colors (see the pathway color legend).

**Figure 3:**
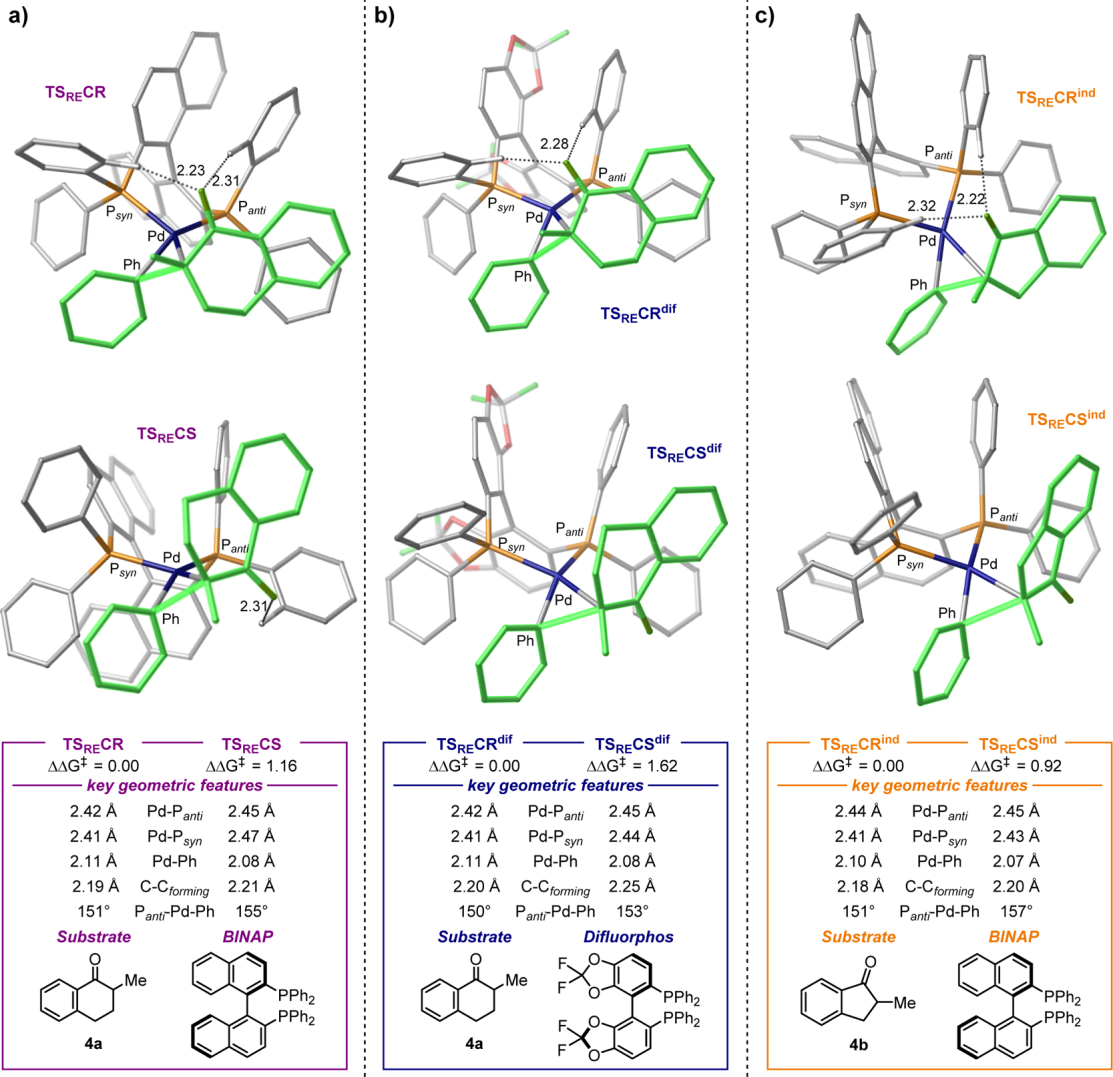
Computed ΔΔ*G*^*⧧*^ values in kcal/mol and geometric features for the reaction with different ligand/substrate combinations at the [CPCM = toluene]PBE-D3/SDD:6-311+G(d,p)//PBE/lanl2dz:6-31G(d) level of theory. (a) (*R*)-**BINAP**/**4a**, (b) (*R*)-**Difluorphos**/**4a**, and (c) (*R*)-**BINAP**/**4b**. The phenyl and enolate ligands undergoing C–C bond formation are highlighted in green. C–H···O NCIs are highlighted as black dotted lines and their values are reported in Å. All of the hydrogen atoms not involved in highlighted interactions are omitted for clarity.

Starting from Pd(0), π-coordination of bromobenzene **5** and subsequent **OA** into the C–Br bond lead to the irreversible formation of the intermediate **7** (Figure 2). The **OA** activation barrier is 8.26 kcal/mol. The subsequent **TM** step can occur by three different mechanisms: (i) bromide/enolate dissociative anion exchange, (ii) classical 4-membered cyclic transmetallation to give the O-bound Pd-enolate, and (iii) vinylogous 6-membered cyclic transmetallation to give the C-bound Pd-enolate (Figure 2). Because of the low polarity of toluene, the formation of a cationic complex upon bromide dissociation is highly unfavored (*ca*. 42 kcal/mol, see the Supporting Information). Thus, the **TM** must occur between **7** and the Na-enolate **8** via cyclic TSs, **TS**_**TM**_**O** or **TS**_**TM**_**C**.^39^ The latter one can exist in two diastereomeric forms (**TS**_**TM**_**CR** and **TS**_**TM**_**CS**, Figure 2), which at the end of the catalytic cycle lead to the formation of the two enantiomeric products (*R*)-**6a** and (*S*)-**6a**. Hence, evaluation of both the activation barrier Δ_TM_*G*^*⧧*^ and the reaction Gibbs free energy Δ_TM_*G* associated to the **TM** step is important. Depending on these values compared to the **RE** Δ*G*^*⧧*^, the formation of the Pd-substituted stereocenter can be stereodetermining. The O-bound Pd-enolate forms with Δ*G*^*⧧*^ = 17.03 kcal/mol via **TS**_**TM**_**O** to give the intermediate **9** after the loss of NaBr. Formation of the C-bound enolates via **TS**_**TM**_**CR** and **TS**_**TM**_**CS** is much slower, with the enolate eventually leading to (*S*)-**6a** being favored (Δ*G*^*⧧*^ = 23.26 and 20.42 kcal/mol, respectively). Additionally, the intermediate **9** was found to be energetically favored over the corresponding C-bound enolates (*R*)-**10** and (*S*)-**10** by *ca*. 8 kcal/mol. Upon transmetallation, η^3^-oxo-allyl Pd-enolates can also be easily accessed from (*R*)-**10** and (*S*)-**10** (Δ*G*^*⧧*^ = 5.9 and 6.1 kcal/mol, respectively, not shown, see the Supporting Information). These were found to be 3–7 kcal/mol lower in energy compared to enolates **10**. Nevertheless, they were still less favored than **9**. This is consistent with α-disubstituted Pd-enolates typically being observed in their O-bound form.^5^ A TS directly connecting enolate **9** and (*R*)-**10** was located (4.03 kcal/mol on the energy scale of Figure 2), although we were unable to find a corresponding structure between **9** and (*S*)-**10**. This excludes the chance for intramolecular tautomerization of **9** to **10**, as this is energetically demanding. Overall, these data suggest **9** to be the most stable Pd-enolate intermediate. The question that follows is whether it would also be the most reactive one in the next key step. TSs for the **RE** mechanisms depicted in Figure 1b were computed. Starting from **9**, mechanism **B** (Figure 1b) could occur via **TS**_**RE**_**OR** and **TS**_**RE**_**OS**, leading to (*R*)-**6** and (*S*)-**6**, respectively (orange path, Figure 2). The Δ*G*^*⧧*^ values associated to these TSs are 31.69 and 30.94 kcal/mol. This vinylogous mechanism is predicted to give low enantioselectivity toward (*S*)-**6a**, in contrast with the experimental evidence that (*R*)-**6a** is the major product when (*R*)-**BINAP** is used as the ligand. The TSs for mechanism **C** starting from oxo-allyl enolates show similarly high activation parameters (Δ*G*^*⧧*^ = 28.45 and 33.15 kcal/mol), yet correctly predicting (*R*)-**6a** as the kinetically favored product (see the Supporting Information). Finally, the lowest energy pathway was found to be via mechanism **A**. **RE** occurring from C-bound enolates (*R*)-**10** and (*S*)-**10** via **TS**_**RE**_**CR** and **TS**_**RE**_**CS** shows Δ*G*^*⧧*^ as low as 24.37 and 25.76 kcal/mol, respectively (Figures 2and 3). This suggests that despite α-disubstituted enolates bind to Pd preferentially *via* the O atom, this species is unproductive and needs conversion to the less-stable yet more reactive C-bound enolate **1** for the catalytic cycle to proceed.

### Reaction Enantioselectivity

The computed relative energy between the TSs **TS**_**RE**_**CR** and **TS**_**RE**_**CS** is ΔΔ*G*^*⧧*^ = 1.39 kcal/mol. This value is slightly reduced to 1.16 kcal/mol when considering NCIs using the PBE-D3 functional (see the Computational Methods section). Considering the irreversibility of the **RE** step (Δ*G ca*. −28 kcal/mol, Figure 2) and the reversible formation of (*R*)-**10** and (*S*)-**10**, the Curtin-Hammett principle can be applied.^40^ Therefore, the computed ΔΔ*G*^*⧧*^ value is in agreement with the enantioselectivity observed experimentally (66% *ee*, 1.18 kcal/mol).^17^ Analysis of the TSs **TS**_**RE**_**CR** and **TS**_**RE**_**CS** gives insights into the observed preference toward the product (*R*)-**6**. Previous work by Zhou and co-workers^30,31^ on BINOL-based monophosphine ligands showed that C–H···O contacts are key features for accessing high *ee* levels. Such NCIs are also present in both **TS**_**RE**_**CR** and **TS**_**RE**_**CS**, between the carbonyl O atom and ortho-protons of the PPh_2_ groups (Figure 3a). The more favored **TS**_**RE**_**CR** shows two C–H···O interactions (2.23 and 2.31 Å), while **TS**_**RE**_**CS** shows only one (2.31 Å, Figure 3a). Thus, these NCIs seem to be responsible for the observed selectivity at the first glance. Aiming at a validation of our observations, we computed **RE** TSs also for a different ligand and substrate. (*R*)-**Difluorphos** was shown to be a ligand of choice for this transformation, providing stereoselectivity typically higher than 90% *ee* for a range of substrates.^20^ In the case of the benchmark reaction in Figure 1c with the substrate **4a**, (*R*)-**Difluorphos** gave the product (*R*)-**6** in 90 or 85% *ee* (at 60 or 80 °C, respectively) corresponding to a ΔΔ*G*^*⧧*^ range of 1.76–1.95 kcal/mol. On the other hand, the reaction performance was shown to be generally lower when contracting the substrate ring size from tetralone to indanone derivatives.^20^ The *ee* for the reaction with (*R*)-**BINAP** and **4b** is 20% under catalytic conditions and 66% under stoichiometric conditions, corresponding to a ΔΔ*G*^*⧧*^ range of 0.28–1.11 kcal/mol. It should be noted that catalyst decomposition was found to occur under catalytic conditions depending on the ligand, resulting in substantial variability in the observed *ee*.^20^ Nevertheless, our stereochemical model should be able to predict the change in the reaction ΔΔ*G*^*⧧*^ at least qualitatively, if correct. The results obtained are shown in Figure 3, with the structures **TS**_**RE**_**CR**^**dif**^ and **TS**_**RE**_**CS**^**dif**^ corresponding to the TSs for the pair (*R*)-**Difluorphos**/**4a** and **TS**_**RE**_**CR**^**ind**^ and **TS**_**RE**_**CS**^**ind**^ for the pair (*R*)-**BINAP**/**4b**. Computations correctly describe the increase in ΔΔ*G*^*⧧*^ when changing from (*R*)-**BINAP** to (*R*)-**Difluorphos** (1.62 kcal/mol Figure 3b). When considering **TS**_**RE**_**CR**^**ind**^ and **TS**_**RE**_**CS**^**ind**^, a ΔΔ*G*^*⧧*^ value of 0.92 kcal/mol was computed (Figure 3c). This is in agreement with the typical decrement in selectivity associated with this substrate.

**Figure 4:**
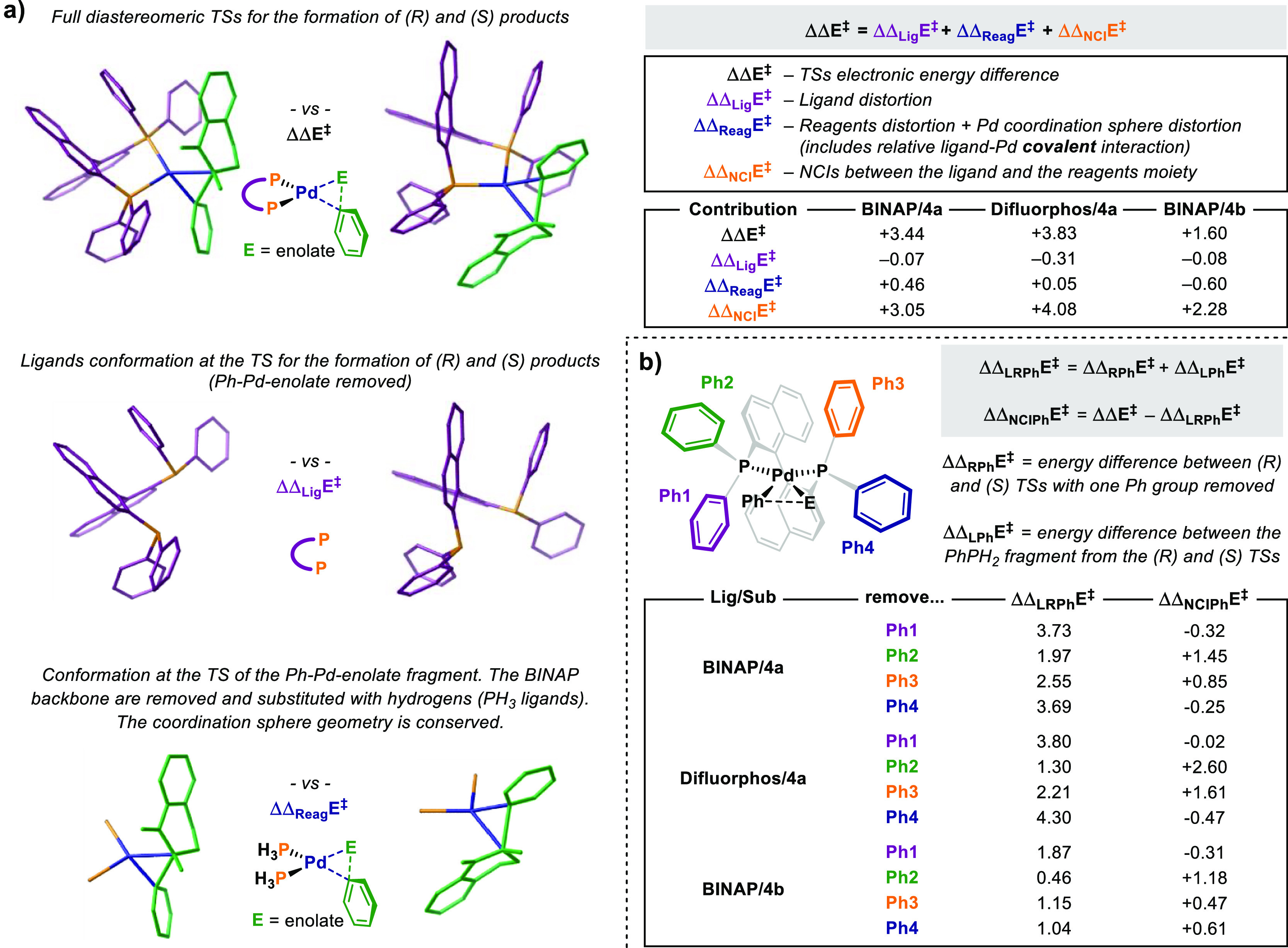
Energy decomposition analysis for the evaluation of the contributions affecting the reaction selectivity at the TS level. (a) General concept and evaluation of the NCI contribution given by the whole ligand. (b) Analysis of the NCI contributions given by each one of the ligand phenyl substituents.

Analysis of the geometry for these systems shows that the reaction selectivity and the C–H···O distances are not correlated. Despite (*R*)-**Difluorphos** gives the best selectivity, C–H···O contacts in **TS**_**RE**_**CR**^**dif**^ (2.28 and 2.28 Å) are longer than those in **TS**_**RE**_**CR** and **TS**_**RE**_**CR**^**ind**^ (2.23 and 2.31 Å, and 2.22 and 2.32 Å, Figure 3). Similarly, there is no trend with the NBO charges of the O and H atoms involved in the interaction (see the Supporting Information). Therefore, additional investigations were undertaken aiming at evaluating the factors affecting the stereochemical outcome. In order to gain more detailed insight into the mode of the interaction between the ligand and the substrate, we turned to an energy decomposition analysis similar to previous studies (Figure 4a).^41−44^

Several contributions are expected to affect the electronic energy difference between the two diastereomeric TSs (ΔΔ*E*^*⧧*^): (i) ligand-Pd bonds, (ii) [Pd(Ph)(enolate)] Pd–C breaking bonds, (iii) ligand distortion, (iv) [Ph-enolate] distortion (which includes the forming C–C bond), (v) distortion of the Pd coordination sphere, and (vi) NCIs between the ligand and the substrate/reagent and of these with the metal center.^45^ The relative contribution due to the NCIs between the ligand and the substrate can be evaluated by considering the TS fragmentation depicted in Figure 4a. ΔΔ_Lig_*E*^*⧧*^ is the electronic energy difference of the sole ligand at the TS and accounts for the relative ligand distortion. Replacing the ligand’s binaphthyl and phenyl groups with H atoms in the TS results in fragments where the relative [Ph-enolate] distortion, Pd coordination sphere, P–Pd bonds, and [Pd(Ph)(enolate)] Pd–C breaking bonds are conserved.^46^ Therefore, the relative energy of such fragments from the two TSs (ΔΔ_Reag_*E*^*⧧*^) would account for all of these contributions taken together. Subtracting ΔΔ_Lig_*E*^*⧧*^ and ΔΔ_Reag_*E*^*⧧*^ from ΔΔ*E*^*⧧*^ gives ΔΔ_NCI_*E*^*⧧*^, which accounts for the relative NCIs between the ligand and the [Pd(Ph)(enolate)]fragment. The values of the computed contributions for each one of the ligand/substrate combinations included in this study are reported in Figure 4. As expected, because of the rigidity of this class of bidentate ligands, ΔΔ_Lig_*E*^*⧧*^ was found to be small. Moreover, it was found to be always negative, that is, in favor of the minor (*S*)-product (−0.07 to −0.31 kcal/mol). The contribution due to the distortion of the [(PH_3_)_2_Pd(Ph) (enolate)] fragment (ΔΔ_Reag_*E*^*⧧*^) impacts the selectivity to a slightly bigger extent, even though in opposite directions depending on the substrate. ΔΔ_Reag_*E*^*⧧*^ is positive in the case of the tetralone derivative **4a** and negative for 2-methylindanone **4b**, suggesting a more favorable arrangement of the former substrate at the TS. The most important contribution to the computed ΔΔ*E*^*⧧*^ is ΔΔ_NCI_*E*^*⧧*^. This was computed to be positive and bigger in magnitude compared to ΔΔ_Lig_*E*^*⧧*^ and ΔΔ_Reag_*E*^*⧧*^ in all the cases, indicating that the selectivity observed is mainly due to attractive^47^ weak interactions between the ligand and the substrate at the TS. Such NCIs are maximized when (*R*)-**Difluorphos** is the ligand, providing ΔΔ_NCI_*E*^*⧧*^ = +4.08 kcal/mol. A value of +3.05 kcal/mol was computed for the (*R*)-**BINAP**/**4a** combination, which further decreased for (*R*)-**BINAP**/**4b** (+2.28 kcal/mol).

ΔΔ*E*^*⧧*^ and ΔΔ_NCI_*E*^*⧧*^ correlate, giving further support to the role of NCIs in the enantiodiscrimination process. Additional detail regarding such NCIs and the groups involved in the recognition event is desirable. This would provide the basis for a more rational design of improved ligands by harnessing NCIs. The effect of a specific group on the computed selectivity can be investigated by removing this from the (*R*) and (*S*)-TSs and by evaluating the change in ΔΔ*E*^*⧧*^ associated to this perturbation. Therefore, following the same approach described in Figure 4a, we performed an energy decomposition analysis of the TSs depicted in Figure 3 by systematically removing the Ph groups of the ligand ((*R*)-**BINAP** or (*R*)-**Difluorphos**). The results obtained are reported in Figure 4b. ΔΔ_LRPh_*E*^*⧧*^ is the relative distortion energy of the truncated TS and of the PhPH_2_^48^ residue summed. ΔΔ_NCIPh_*E*^*⧧*^ is the relative interaction energy due to NCIs engaging the removed Ph group and is calculated as ΔΔ_NCIPh_*E*^*⧧*^ = ΔΔ*E*^*⧧*^ – ΔΔ_LRPh_*E*^*⧧*^. The previously highlighted C–H···O interactions are present in the favored TS of all of the systems examined (*vide infra*). Therefore, it is not surprising that Ph2 and Ph3 are the groups contributing the most to the selectivity, as these are the groups involved in the C–H···O contacts highlighted before. Specifically, the interactions due to these groups account for ΔΔ_NCIPh_*E*^*⧧*^(Ph2) = 1.45, 2.60, and 1.18 kcal/mol and ΔΔ_NCIPh_*E*^*⧧*^(Ph3) = 0.85, 1.61, and 0.47 kcal/mol for (*R*)-**BINAP**/**4a**, (*R*)-**Difluorphos**/**4a**, and (*R*)-**BINAP**/**4b**, respectively (Figure 4b). However, as the geometric and electronic features of these interactions do not account for the trend observed across the three systems (*vide infra*), other effects should be considered to justify the higher ΔΔ_NCIPh_*E*^*⧧*^(Ph2) and ΔΔ_NCIPh_*E*^*⧧*^(Ph3) calculated for (*R*)-**Difluorphos**/**4a**. We reasoned that a destabilizing interaction involving Ph2 and Ph3 could be in place in the (*S*)-TSs.

**Figure 5:**
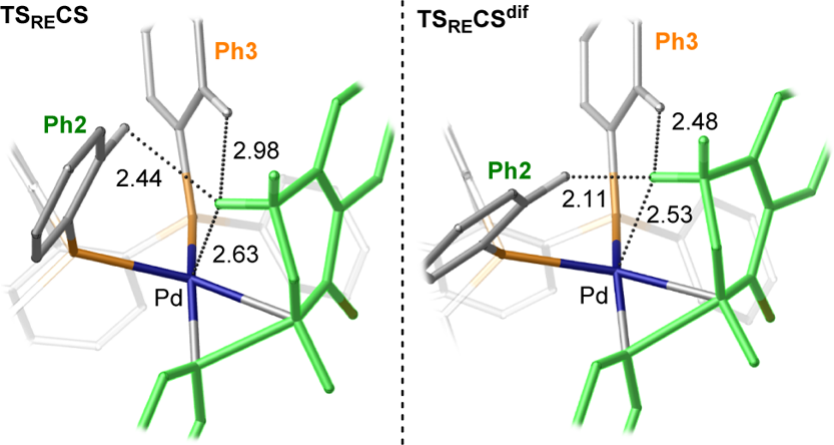
Detail of the structures **TS**_**RE**_**CS** and **TS**_**RE**_**CS**^**dif**^, showing steric repulsion between the γ-methylene group of **4a** and Ph2/Ph3 in the ligand. The phenyl and enolate portions undergoing C–C bond formation are highlighted in green. H···H distances are highlighted as black dotted lines and their values are reported in Å. Hydrogen atoms not involved in highlighted interactions are omitted for clarity.

In both **TS**_**RE**_**CS** and **TS**_**RE**_**CS**^**dif**^, the tetralone γ-methylene moiety is engaged in a CH···Pd interaction with the Pd occupied d_*z*^2^_ orbital (Figure 5), which is shorter in the case of (*R*)-**Difluorphos** (2.53 Å) than for (*R*)-**BINAP** (2.63 Å). This interaction causes the substrate to be in an area occupied by Ph2 and Ph3. However, in **TS**_**RE**_**CS**^**dif**^, the distance between the substrate CH_2_ group and Ph2 is much shorter than that in **TS**_**RE**_**CS**. For instance, the H···H distances between the methylene of **4a** and the ortho-proton of Ph2 are 2.11 and 2.44 Å in **TS**_**RE**_**CS**^**dif**^ and **TS**_**RE**_**CS**, respectively (Figure 5). Although the latter distance is about the sum of the van der Waals radii (2.40 Å), the former value is remarkably below this threshold, suggesting steric repulsion between these two moieties. Altogether, these data suggest that (*R*)-**Difluorphos** outperforms (*R*)-**BINAP** not only by stabilization of the favorite (*R*)-TS via NCIs but also by destabilization of the less-stable (*S*)-TS via steric repulsion. Additionally, as indanone **4b** is characterized by a contracted ring size, this substrate lacks the methylene moiety responsible for such destabilization, resulting in loss of selectivity when compared to tetralone **4a**.

## Conclusions

The Pd-catalyzed α-arylation of carbonyl compounds is a fundamental reaction in transition metal catalysis. Extensive mechanistic analyses disclosed the general reaction mechanism. However, only limited information was available regarding the actual mechanism governing the formation of the product C–C bond. It was hypothesized^5−8^ that this could proceed via three possible mechanisms: (i) **RE** via a classic three-membered cyclic TS from a C-bound enolate; (ii) vinylogous **RE** from an O-bound enolate via a five-membered cyclic TS; and (iii) **RE** from a η^3^-bound oxo-allyl Pd-enolate. To the best of our knowledge, there were no computational studies aiming at distinguishing between these. In this work, we have computed the reaction energy profile for the general mechanism initially hypothesized by Buchwald and Hartwig (Figure 1a). Computations show that the benchmark [Pd((*R*)-**BINAP**)]-catalyzed coupling between 2-methyltetralone and bromobenzene proceeds via facile **OA** followed by **TM** to give C-bound enolates. Formation of the O-bound enolate is also possible and more favored, but this is shown to be an unproductive off-cycle species. **RE** is computed to be the stereo- and rate-determining step of the reaction, which proceeds via direct C–C bond formation from the C-bound enolates. The reaction major product was correctly computed to have (*R*)-configuration when a (*R*)-ligand is used. This is due to stabilization of the (*R*)-TS by electrostatic C–H···O contacts and destabilization of the (*S*)-TS by specific steric repulsion. Even though the attractive NCIs are similar in strength across the different ligand–substrate combinations explored; (*R*)-**Difluorphos** optimizes repulsive interactions, leading to higher enantioselectivity. The formulation of such a detailed stereochemical model was possible thanks to the use of an energy decomposition analysis aimed at evaluating the contribution of each ligand’s Ph substituent to the reaction selectivity. This approach was previously reported by Wheeler in the context of chiral Brønsted acid-catalyzed reactions.^41,42^ However, to the best of our knowledge, this is the first time it is applied to the rationalization of an enantioselective reaction with biaryl phosphine ligands, for which a quadrant visual analysis is most often used. As the development of stereoselective versions of the α-arylation of carbonyl compounds still suffers limitations,^34^ we envision that this work will allow a more rational design of catalytic systems for this class of transformations in the future.

## Experimental Section

### Computational Methods

The selection of a suitable computational method capable of describing sensitive equilibria between Pd-enolate species is crucial for the success of this study. Therefore, preliminary work aiming at the identification of an appropriate functional was undertaken and is reported in the Supporting Information The results reported were obtained by geometry optimization at the BP86/Lanl2dz:6-31G(d) level of theory (Lanl2dz pseudopotential for Pd, Na, and Br atoms).^49,50^ Stationary points on the potential energy surface were determined to be minima (no vibrational modes with imaginary frequency) or TSs (TS, only one mode with imaginary vibrational frequency) by vibrational analysis at the same level. Finer single point energy (SPE) calculations were performed at the PBE/SDD:6-311+(d,p) level^51^ with the polarizable continuum model CPCM^52^ for toluene, which is the solvent used experimentally.^17,20^ Thermal corrections were calculated from the vibrational analysis at the BP86/Lanl2dz:6-31G(d) level of theory on the optimized geometries. We arbitrarily report in the text the results obtained with PBE, as these provide a better fit to the experimental data. However, other DFT methods including hybrid functionals were evaluated and found to reproduce the results obtained with the PBE functional (see the Supporting Information).

It is well-recognized that dispersion corrections are normally needed to account for NCIs. In this specific study, we found that functionals including long-range dispersion failed in reproducing the experimental distribution between the two Pd-enolate tautomeric forms (see the Supporting Information). We posit this to be due to an overestimation of the NCIs between the C-bound enolate and the Pd-complex. α-Disubstituted enolates are found in solution as O-bound tautomers **2** most likely because of steric repulsion, which seems to be underestimated in the case of long-range-corrected functionals. On the other hand, NCIs are likely to be involved in the molecular recognition process, leading to the enantiodiscrimination of the products.^53^ Indeed, we computed the diastereomeric TSs of the reaction stereodetermining step, *also* adding the Grimme long-range dispersion correction GD3^54^ to the PBE functional (PBE-D3/SDD:6-311+(d,p)). This provided a better fit with the experimental enantioselectivity trend for the catalyst/substrate combinations explored, suggesting the requirement for specific computational tools for the investigation of different aspects of this reaction.

## Supporting Information Available

Computational methods, coordinates of located structures, and corresponding thermochemical data (PDF)
