# Supplementary material for: Computational Analysis of Enantioselective Pd-Catalyzed α-Arylation of Ketones
Source: J Org Chem. 2020 Jul 31;85(17):11511–8. doi: 10.1021/acs.joc.0c01768 (PMC8009508; doi:10.1021/acs.joc.0c01768)
Supplement: Supplementary file 1 — jo0c01768_si_001.pdf [file jo0c01768_si_001.pdf]

Computational Analysis of an Enantioselective Pd-Catalyzed α-Arylation of Ketones

Manuel Orlandi,[a] Giulia Licini[a,b]*

[a]Department of Chemical Sciences, University of Padova, via Marzolo 1, 35131 Padova.
[b]CIRCC – Consorzio Interuniversitario per le Reattività Chimiche e la Catalisi, Padova Unit.

Supporting Information

General Information	S1
Evaluation of Functionals	S1
Distribution of Pd-enolate species	S2
Reaction energy profile with other functionals	S3
Dissociative pathway for the transmetallation step	S4
Enantioselectivity and energy decomposition analysis	S4
Energies	S9
Geometries	S11
References	S67

General Information

All computations were performed using the Gaussian 09[1] suite of programs at the C_3P facility of the University of Padova. Due to the relatively high rigidity of the system under exam, the conformational analyses were performed manually by relaxed scan of angles and dihedrals via the *scan* keyword. Optimizations and conformational analyses were performed at the BP86/6-31G(d) level of theory and lanl2dz was used for Pd, Br, and Na. For structures that gave tough convergence the keyword scf=xqc was used. Stationary points on the potential energy surface were determined to be minima (no vibrational modes with imaginary frequency) or transition structures (TS, only one mode with imaginary vibrational frequency) by vibrational analysis at the same level of theory. Thermal corrections were calculated from the vibrational analysis on the optimized geometries. SPE calculations were performed with basis set SDD:6-311+(d,p) and with polarizable continuum model CPCM for toluene as this solvent was the one used experimentally by the Buchwald and Hartwig groups (see main text). The functionals tested were BP86, M06, wB97XD, B3LYP, B3LYP-D3, PBE, TPSSh. These were chosen as they have a little amount of exact exchange, as recommended by Truhlar and coworkers for the computational treatment of transition metals.[2] Visualization of the computed structures was carried out using CYLView.[3]

Evaluation of Functionals

The choice of a computational method capable of describing sensitive equilibria between different Pd enolate species is crucial for this study. Experimental data regarding the relative stability of Pd enolate complexes are available in the literature thanks to extensive mechanistic studies by the Hartwig group.[4] Therefore, computation of some of these complexes in both the *C*- and *O*-enaolate forms and evaluation of their relative energies was undertaken with a set of functionals. This would provide an initial informative screening of the functionals able to describe the energetics of this class of complexes.

Complexes **S1-S3** were synthesized by Culkin and Hartwig.[4] It was observed by NMR that these complexes present the *C*-bouund to *O*-bound enolate tautomeric ratios reported in Table S1 (see the exp. a/b ratios and the corresponding exp. ΔG values). Geometries for these complexes were computed at the BP86/lanl2dz:6-31G(d) level of theory. Subsequent SPE calculation with basis set SDD:6-311+G(d,p) and functionals BP86, M06, wB97XD, B3LYP, B3LYP-D3, PBE, and TPSSh provided the Gibbs free energy values reported in Table 1 in kcal/mol. The values for all of the conformers are shown. As depicted in the table, letter **a** denotes *C*-enolate tautomers, while **b** denotes *O*-enolate tautomers.

Table S1.

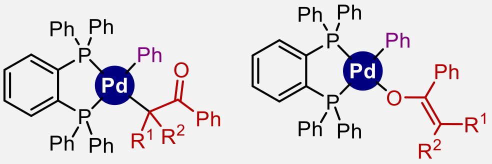

a, *C*-enolate   **b**, *O*-enolate

S1, R^1=H, R^2=H
S2, R^1=H, R^2=Me
S3, R^1=Me, R^2=Me

	exp. a/b	Conf.	ΔG (kcal/mol) BP86	M06	wB97XD	B3LYP	B3LYP-D3	PBE	PBE-D3	TPSSh
**S1**	>99:1	**a_1**	0.97	1.34	1.17	0.93	1.28	1.01	1.14	0.97
		**a_2**	1.35	2.78	3.09	1.31	3.87	1.75	3.23	1.47
		**a_3**	3.53	2.31	2.50	3.49	2.88	3.22	2.46	3.64
		**a_4**	4.80	3.71	3.87	4.80	4.23	4.54	3.86	4.81
		**a_5**	**0.00**	**0.00**	**0.00**	**0.00**	**0.00**	**0.00**	**0.00**	**0.00**
		**b_1**	8.48	13.87	12.38	7.23	11.07	9.29	11.60	8.17
		**b_2**	12.59	14.84	12.98	11.82	11.65	12.69	12.35	11.96
		**b_3**	11.02	12.71	11.39	10.07	9.68	11.23	10.77	10.31
		**b_4**	8.27	**11.70**	**10.05**	6.82	**8.44**	8.77	**9.49**	7.55
		**b_5**	9.51	12.91	11.22	8.05	9.65	10.14	11.04	8.98
		**b_6**	**7.74**	14.13	12.30	**6.48**	11.05	**8.75**	11.55	**7.50**
		**b_7**	9.66	14.89	13.08	8.90	11.88	10.33	12.18	9.25
		**b_8**	12.10	13.19	11.58	11.35	10.19	12.12	11.10	11.20
**S2**	>99:1	**a_1**	1.11	1.17	1.35	1.23	1.09	1.12	1.17	1.16
		**a_2**	1.20	0.61	0.47	1.46	0.81	0.87	0.29	0.88
		**a_3**	**0.00**	**0.00**	**0.00**	**0.00**	**0.00**	**0.00**	**0.00**	**0.00**
		**a_4**	4.53	6.13	5.60	4.45	6.27	4.83	6.04	4.72
		**a_5**	6.69	9.14	8.60	7.13	8.58	7.04	8.16	7.00
		**a_6**	7.26	8.04	7.58	7.74	8.29	7.44	7.87	7.11

		**a_7**	9.59	8.21	7.95	10.06	8.77	9.25	8.25	9.45
		**a_8**	5.64	6.55	5.88	5.96	5.87	5.77	5.84	5.70
		**b_1**	4.02	10.17	8.21	2.11	6.35	4.79	7.26	3.68
		**b_2**	7.31	12.18	9.98	6.43	8.40	7.62	8.70	6.93
		**b_3**	6.09	12.09	10.41	5.17	8.98	6.80	9.01	5.93
		**b_4**	**3.08**	**8.96**	**7.23**	**1.41**	**5.25**	**3.72**	**5.80**	**2.51**
		**b_5**	7.24	14.02	12.07	6.28	10.45	8.05	10.58	7.27
		**b_6**	6.11	9.43	8.01	5.16	6.39	6.27	6.64	5.31
**S3**	1:17	**a_1**	7.52	-1.54	0.18	10.09	2.90	6.45	2.47	7.16
		**a_2**	11.33	1.36	4.20	14.76	5.95	9.80	4.56	11.69
		**a_3**	**5.23**	**-4.81**	**-2.43**	**7.70**	**0.07**	**4.17**	**-0.16**	**5.21**
		**a_4**	10.31	2.48	4.68	12.53	7.14	9.64	6.96	10.37
		**b_1**	3.88	3.53	3.37	4.35	3.52	3.97	3.72	4.32
		**b_2**	4.84	4.34	3.92	5.71	4.16	4.63	3.83	5.08
		**b_3**	**0.00**	**0.00**	**0.00**	**0.00**	**0.00**	**0.00**	**0.00**	**0.00**
		**b_4**	4.00	1.00	1.23	4.79	1.51	3.39	1.25	3.67

The data reported in Table S1 show that all of the functionals tested correctly predict the *C*-enolates **S1a** and **S2a** to be favored over their corresponding *O*-enolates **S1b** and **S2b**. However, only BP86, B3LYP, PBE, and TPSSh correctly predict the inversion of the selectivity towards enolate **S3b**. B3LYP shows a qualitatively correct prediction yet with an exceedingly high energy difference. Popular functionals in the field such as M06 or wB97XD failed describing the correct relative stability of **S3a** and **S3b**. Moreover, addition of Grimme's long range dispersion correction GD3 to B3LYP and PBE (namely, B3LYP-D3 and PBE-D3) almost inverted the prediction towards the isomer **S3b**, in line with the data provided by wB97XD (which also incorporates long range dispersion correction). Thus, the incorporation of long range dispersion does not allow describing the steric effects responsible for the change of stability of the two tautomeric forms of Pd-enolates. In summary, this preliminary analysis shows that PBE/SDD:6-311+G(d,p)[CPCM=Tol]//BP86/lanl2dz:6-31G(d) is a good set-up for the computation of Pd enolates. This justifies the use this method for the studies regarding the reaction energy profile of the enantioselective Pd-catalyzed α-arylation of ketones hereby reported.

Distribution of Pd-enolate species

In Figure S1 the reaction energy profile for the formation of all of the possible enolate species is reported. From the profile, it is clear that the O-bound enolate is the most stable enolate species. Additionally, the C-bound enolate can isomerize leading to the formation of the η^3-oxo-allyl enolate **11**.

Figure S1

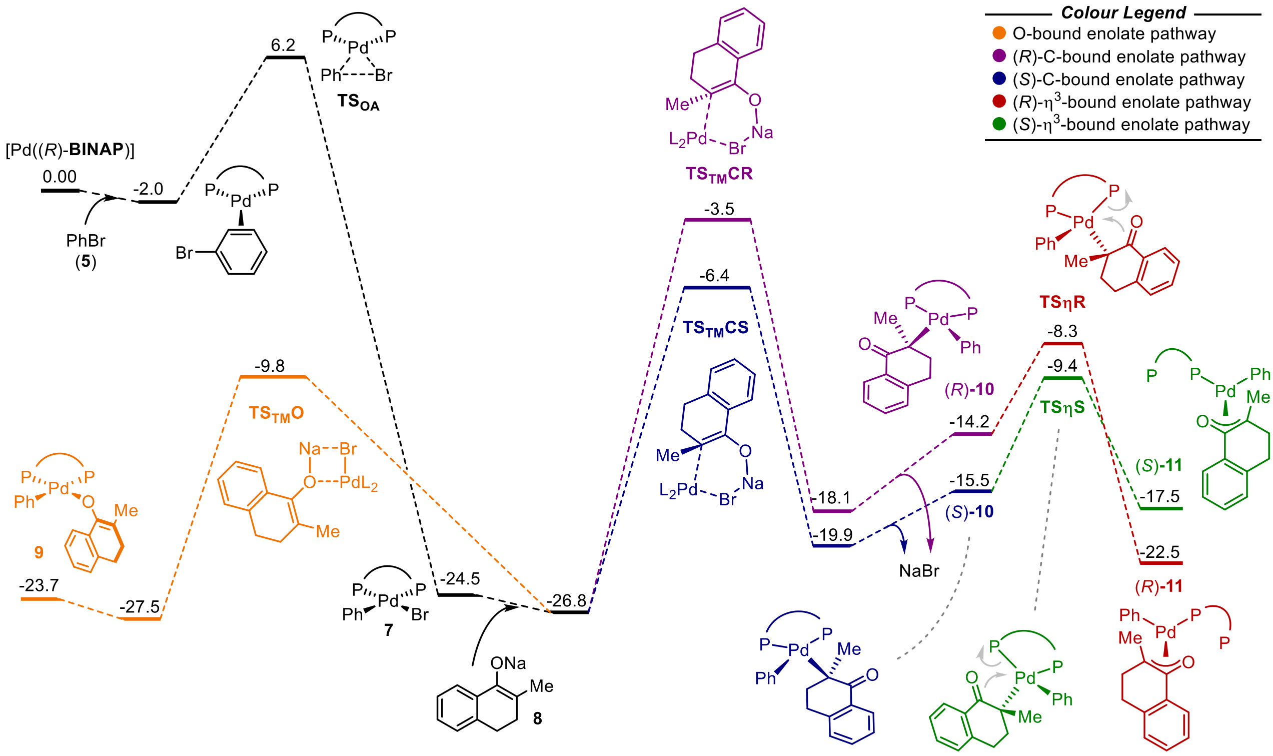

Reaction energy profile with other functionals

In Figure S2 and S3 are reported the energy profiles for the formation of the Pd enolates and for the whole reaction with BP86 and TPSSh (in parenthesis). These were found to be in good agreement with the one reported in the text (PBE functional).

Figure S2

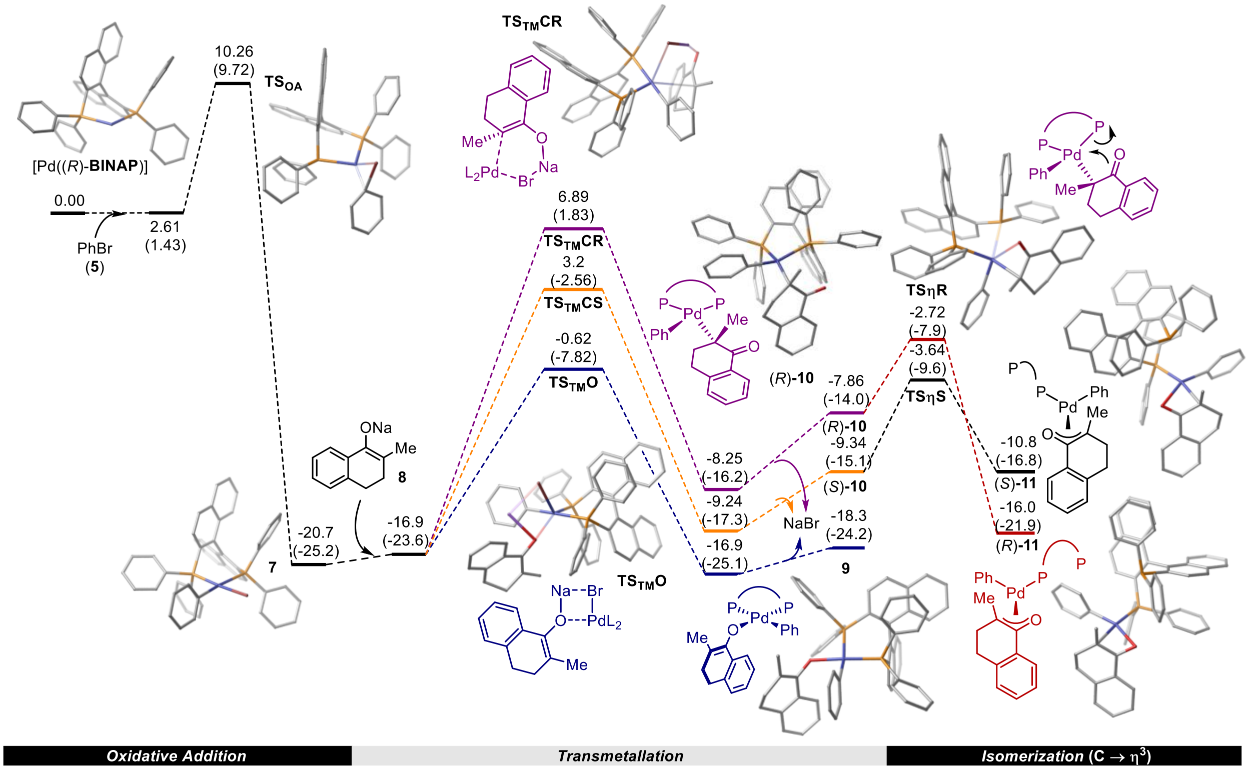

Figure S3

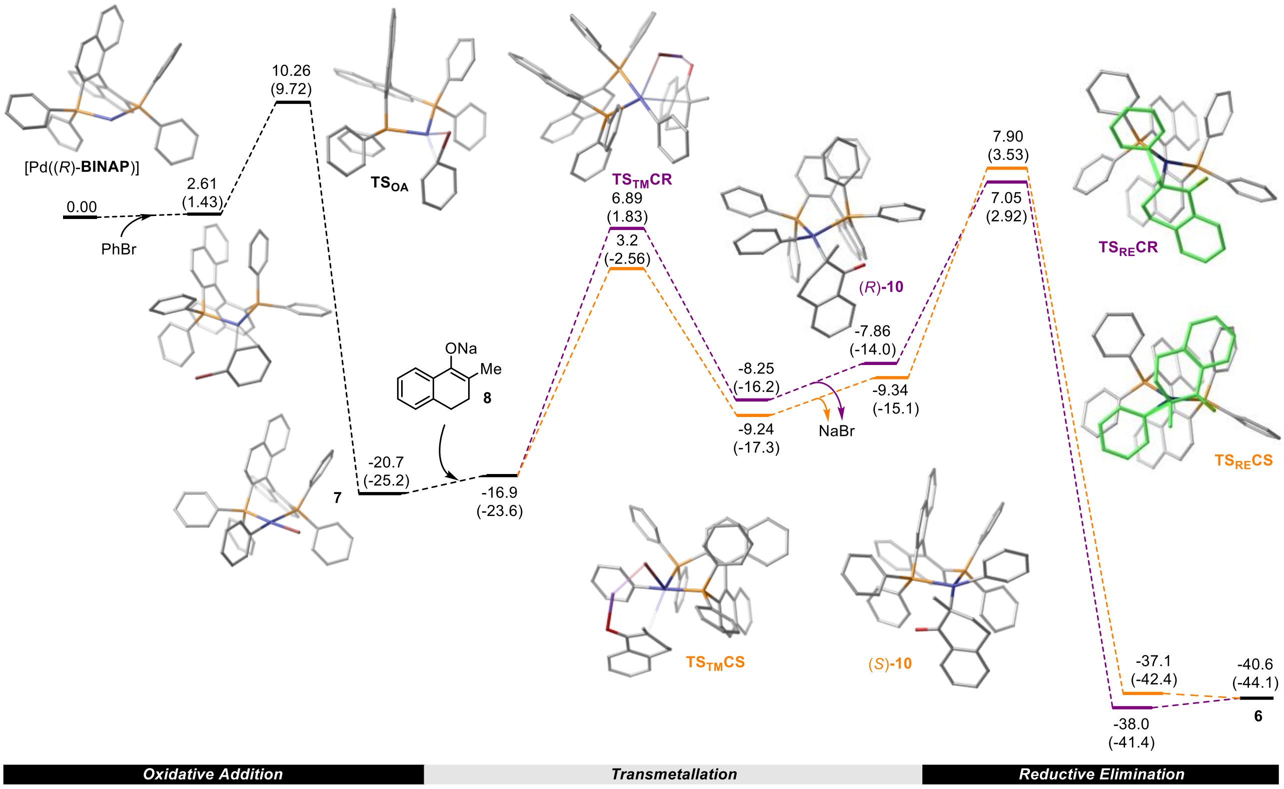

Dissociative pathway for the Transmetallation step

The transmetallation step leading to the formation of the Pd-enolate can occur by three different pathways:

I. Dissociative pathway: the bromide anion could dissociate leading to the formation of a cationic Pd intermediate **S4** before the enolate coordinates.

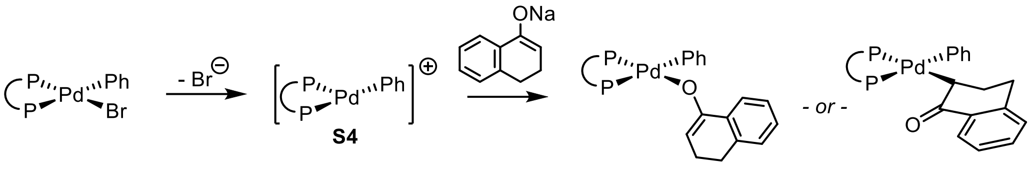

II. “Classic” 4 membered cyclic pathway: the Na-enolate undergoes a metathesis reaction leading to the contextual formation of a Pd-O bond and NaBr.

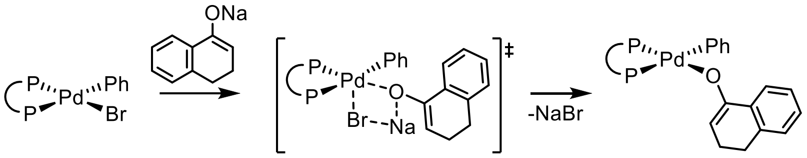

III. “Vinylogous” 6 membered cyclic pathway: the Na-enolate undergoes a metathesis reaction leading to the contextual formation of a Pd-C bond and NaBr.

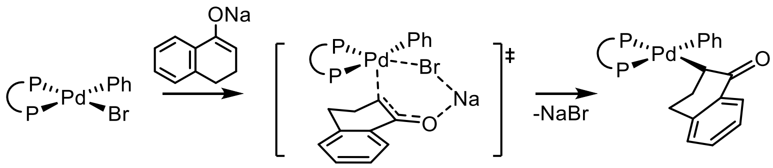

Pathways II and III are discussed in the manuscript. Pathway I was computed as well by evaluation of the dissociation energy of the bromide anion from **S4**. This energy was found to be 42.2 kcal/mol at the PBE/SDD:6-311+G(d,p) level of theory in toluene (CPCM), and 40.0 kcal/mol at the same level but with functional BP86. This could be ascribed to the low dielectric constant of toluene. Moreover, it cannot be excluded that the dissociation pathway could be operative in the case of less coordinating anions than bromide such as triflate [ref 20, 30, and 31 in the manuscript].

Enantioselectivity and energy decomposition analysis

TSs for the stereodetermining step (reductive elimination, **RE**) were found by relaxed scan of the distance between the two reacting C atoms from enolates **10**. Additional conformers were found by relaxed scan of the dihedral angle Pd-C-C-C(O) by keeping the C-C bond length fixed at 2.2 Å followed by re-optimization to TS. The low lying TS leading to the (*R*)-product was found to have two C-H···O contacts as explained in the manuscript. Several attempts were made to locate structures with similar interactions leading also to the (*S*)-products starting from geometries where the C-H···O distances were kept fixed and then re-optimized. However, our efforts were unfruitful as the re-optimization step always led to loss of the C-H···O contacts.

The conformational analysis was performed *ex novo* to locate TSs for the (*R*)-**BINAP**/**4b** combination (**TS$_{RE}$CR^{ind}** and **TS$_{RE}$CS^{ind}**). To locate TSs for the (*R*)-**Difluorphos**/**4a** system, we started from the structures of the(*R*)-**BINAP**/**4a** system and changed the ligand backbone before re-optimizing to TS (only the structures that had $\Delta\Delta G^{\ddagger} < 5$ kcal/mol were considered).

The $\Delta\Delta E^{\ddagger}$ and $\Delta\Delta G^{\ddagger}$ values for all of the systems with all the functionals used is reported in **Figure S4** together with key NBO charges. Together with the interatomic distances reported in the manuscript, these latter values show how the CH···O interactions are similar in strength across the different systems examined. This justifies the use of additional tools such as the energy decomposition analysis to detail the reasons for the observed enantioselectivity.

Figure S4

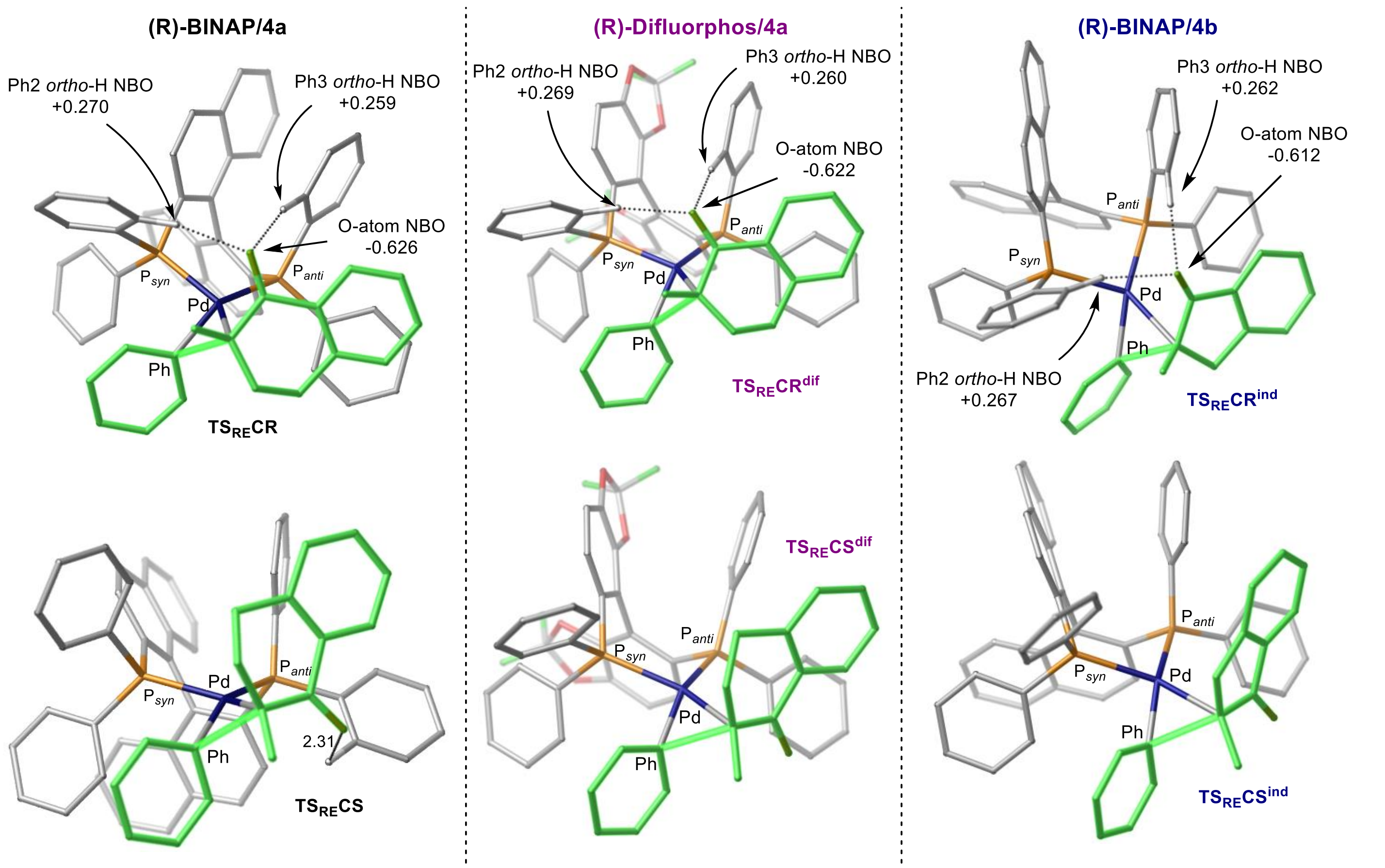

(R)-BINAP/4a

funtional	ΔΔE‡	ΔΔG‡
BP86	3.20	1.31
TPSSh	3.39	1.51
PBE	3.28	1.39
PBE-D3	3.05	1.16

Substrate ***BINAP***

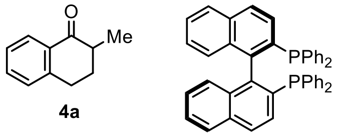

4a

(R)-Difluorphos/4a

funtional	ΔΔE‡	ΔΔG‡
BP86	2.89	1.37
TPSSh	3.09	1.57
PBE	3.06	1.54
PBE-D3	3.14	1.62

Substrate ***Difluorphos***

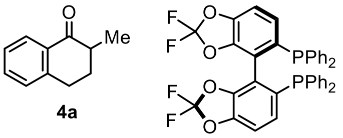

4a

(R)-BINAP/4b

funtional	ΔΔE‡	ΔΔG‡
BP86	2.50	1.16
TPSSh	1.90	0.57
PBE	2.26	0.92
PBE-D3	1.06	-0.27

Substrate ***BINAP***

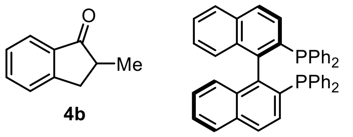

4b

The energy decomposition analysis was performed at the PBE-D3/6-311+G(d,p)//BP86/6-31G(d) level in the gas phase in order to optimize the description of the NCIs present in the TSs. The following relative contributions are supposed to be present in the complexes considered (also depicted in Figure S5):

a) Through bond interactions:
   - ligand-metal P-Pd bonds
   - Pd-C breaking bonds with both the Ph and the enolate ligands
b) Through space interactions:
   - Noncovalent interactions (NCIs) between the ligand and the substrate/reagent, and of these with the metal center.
c) Distortion:
   - ligand distortion
   - [Ph-enolate] fragment distortion (which includes the forming C-C bond)
   - distortion of the Pd-coordination sphere

Figure S5

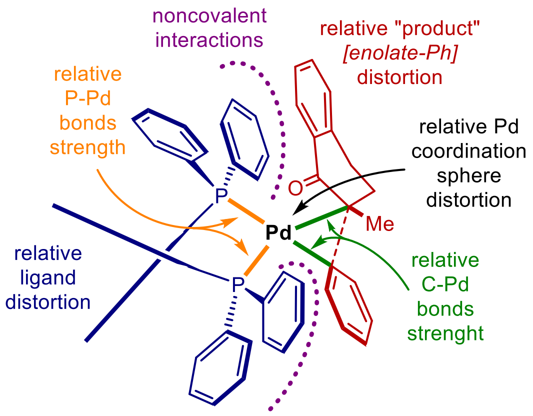

Evaluation of all of these contributions individually is not trivial. In fact, some of these contributions are intimately related and can be hardly separated. For instance, dissociation of the ligand from the metal center would also allow for the evaluation of the ligand-Pd bonds strength in principle. However, due to the significant distortion of the coordination sphere at the TS out of the square planar, this would result in a significant error. This error would originate from neglecting significant Pd orbitals rearrangement in the absence of the P atoms in the [Pd(Ph)(enolate)] fragment. Thus, the P-Pd bonds strength cannot be evaluated by simple dissociation without affecting the coordination sphere distortion.

On the other hand, evaluation of the relative contribution due to NCIs between the ligand and the substrate can be done by making a reasonable assumption as follow. As depicted in Figure S6, removing the ligand backbone (leaving therefore the P atoms) and replacing it with H atoms would result in a fragment where all of the contributions are conserved but two: the ligand distortion and the NCIs contributions. Here the only approximation is the assumption that the (relative) NCIs between the added H atoms and the rest of the fragment is negligible. The relative energy of such fragment in the (*R*)-TS and (*S*)-TS evaluated by SPE calculation in the gas phase at the PBE-D3/6-311+G(d,p)//BP86/6-31G(d) level is $\Delta\Delta_{Reag}E^{\ddagger}$. One of the two remaining contributions, namely the relative ligand distortion $\Delta\Delta_{Lig}E^{\ddagger}$, can be easily derived from the relative energy of the ligand conformation in the (*R*)-TS and (*S*)-TS (by removing reagents and the Pd atom from the TSs followed by SPE calculation). Subtraction of $\Delta\Delta_{Reag}E^{\ddagger}$ and $\Delta\Delta_{Lig}E^{\ddagger}$ from the "total" relative energy of the two TSs ($\Delta\Delta E^{\ddagger}$) gives the last contribution $\Delta\Delta_{NCI}E^{\ddagger}$, which accounts for the relative NCIs occurring between the ligand and the rest of the molecule.

NOTE. The ligand backbone was replaced with H atoms, the geometry of which was then optimized by keeping the rest of the molecule frozen. Therefore the position of the H atoms was optimized while conserving the geometry of the atoms deriving from the TS.

Figure S6

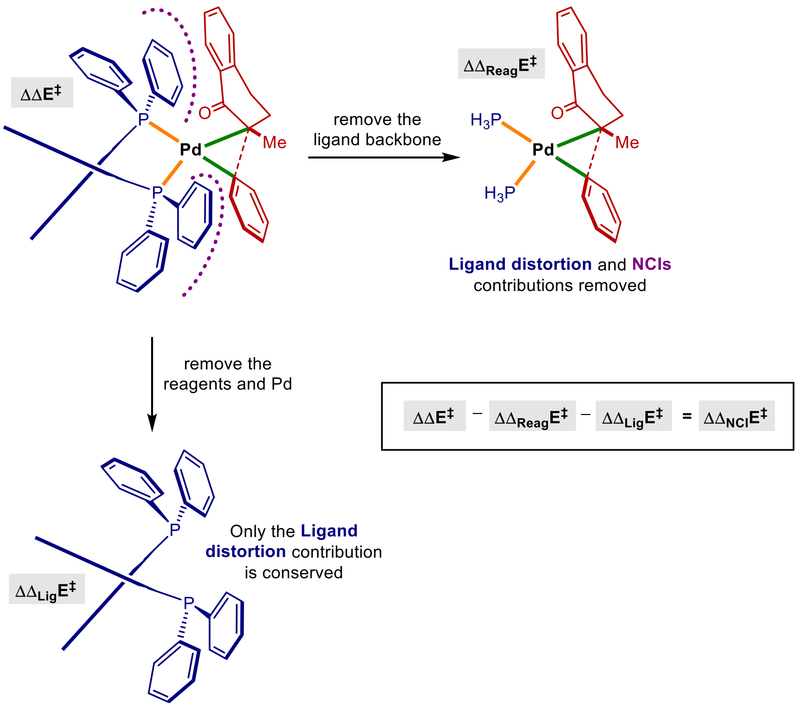

The energy decomposition analysis for the system (*R*)-**BINAP**/**4a** (**TS$_{RE}$CR** and **TS$_{RE}$CR**) is reported in Table S2.

Table S2

Fragment	E(S) (Hartree)	E(R) (Hartree)	Contribution value (kcal/mol)	contribution
Full TS	-3236.58015708	-3236.58564398	+3.44	$\Delta\Delta E^{\ddagger}$
Ligand	-2376.65658955	-2376.65648452	-0.07	$\Delta\Delta_{Lig}E^{\ddagger}$
[$(PH_3)_2$Pd(Ph)(enolate)]	-1545.84260205	-1545.84333266	+0.46	$\Delta\Delta_{Reag}E^{\ddagger}$
			+3.05	$\Delta\Delta_{NCI}E^{\ddagger}$

The energy decomposition analysis for the system (*R*)-**Difluorphos**/**4a** (**TS$_{RE}$CR^{dif}** and **TS$_{RE}$CR^{dif}**) is reported in Table S3.

Table S3

Fragment	E(S) (Hartree)	E(R) (Hartree)	Contribution value (kcal/mol)	contribution
Full TS	-3703.20653043	-3703.21262729	+3.83	$\Delta\Delta E^{\ddagger}$
Ligand	-2843.28473462	-2843.28424713	-0.31	$\Delta\Delta_{Lig}E^{\ddagger}$
[$(PH_3)_2$Pd(Ph)(enolate)]	-1544.33753901	-1544.33761868	+0.05	$\Delta\Delta_{Reag}E^{\ddagger}$
			+4.08	$\Delta\Delta_{NCI}E^{\ddagger}$

The energy decomposition analysis for the system (*R*)-**BINAP**/**4b** (**TS$_{RE}$CR^{ind}** and **TS$_{RE}$CR^{ind}**) is reported in Table S4.

Table S4

Fragment	E(S) (Hartree)	E(R) (Hartree)	Contribution value (kcal/mol)	contribution
Full TS	-3197.31219425	-3197.31474151	+1.60	$\Delta\Delta E^{\ddagger}$
Ligand	-2376.65766781	-2376.65753722	-0.08	$\Delta\Delta_{Lig}E^{\ddagger}$
[$(PH_3)_2$Pd(Ph)(enolate)]	-1506.57270501	-1506.57174428	-0.60	$\Delta\Delta_{Reag}E^{\ddagger}$
			+2.28	$\Delta\Delta_{NCI}E^{\ddagger}$

After evaluating the total NCIs contribution to the enantioselectivity, we sought to determine which groups of the ligand would contribute the most to the selectivity. This would help future ligand development for this transformation. Therefore, we applied the aforementioned energy decomposition analysis to each one of the ligand's Ph substituents as depicted in Figure S7.

Figure S7

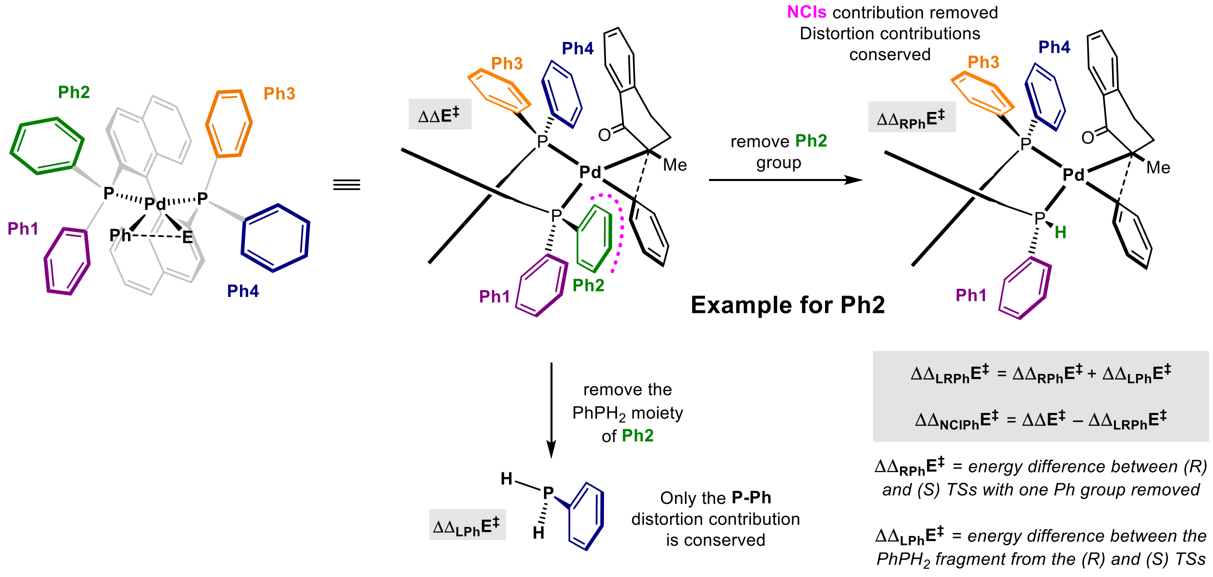

The energy decomposition analysis for the system (*R*)-**BINAP**/**4a** (**TS$_{RE}$CR** and **TS$_{RE}$CR**) is reported in Table S5.

Table S5

Fragment	E(S) (Hartree)		E(R) (Hartree)		ΔΔE‡ (kcal/mol)	$\Delta\Delta_{LRPh}E^‡$ (kcal/mol)	$\Delta\Delta_{NCIPh}E^‡$ (kcal/mol)
	Residual TS	$PhPH_2$	Residual TS	$PhPH_2$			
Full TS	-3236.58015708	-	-3236.58564398	-	+3.44	-	-
Ph1	-3005.74820817	-573.792272189	-3005.75415347	-573.792319991	-	+3.76	-0.32
Ph2	-3005.75233555	-573.792340315	-3005.75546993	-573.792389297	-	+2.00	+1.45
Ph3	-3005.74803946	-573.792212727	-3005.75209711	-573.792288461	-	+2.59	+0.85
Ph4	-3005.75371377	-573.79244003	-3005.75959889	-573.792434705	-	+3.69	-0.25

The energy decomposition analysis for the system (*R*)-**Difluorphos**/**4a** (**TS$_{RE}$CR^{dif}** and **TS$_{RE}$CR^{dif}**) is reported in Table S6.

Table S6

Fragment	E(S) (Hartree)		E(R) (Hartree)		ΔΔE‡ (kcal/mol)	$\Delta\Delta_{LRPh}E^‡$ (kcal/mol)	$\Delta\Delta_{NCIPh}E^‡$ (kcal/mol)
	Residual TS	$PhPH_2$	Residual TS	$PhPH_2$			
Full TS	-3703.20653043	-	-3703.21262729	-	+3.83	-	-
Ph1	-3472.37264914	-573.792338790	-3472.37871263	-573.792411127	-	+3.85	-0.02
Ph2	-3472.37891117	-573.792487927	-3472.38097552	-573.792382217	-	+1.23	+2.60
Ph3	-3472.37361579	-573.792311340	-3472.37713141	-573.792330011	-	+2.22	+1.61
Ph4	-3472.37839884	-573.792477201	-3472.38524459	-573.792483341	-	+4.30	-0.47

The energy decomposition analysis for the system (*R*)-**BINAP**/**4b** (**TS$_{RE}$CR^{ind}** and **TS$_{RE}$CR^{ind}**) is reported in Table S7.

Table S7

Fragment	E(S) (Hartree)		E(R) (Hartree)		ΔΔE‡ (kcal/mol)	$\Delta\Delta_{LRPh}E^‡$ (kcal/mol)	$\Delta\Delta_{NCIPh}E^‡$ (kcal/mol)
	Residual TS	$PhPH_2$	Residual TS	$PhPH_2$			
Full TS	-3197.31219425	-	-3197.31474151	-	+1.60	-	-
Ph1	-2966.480257	-573.7922588	-2966.483231	-573.7923314	-	1.91	-0.31
Ph2	-2966.483576	-573.7924551	-2966.484314	-573.7923869	-	0.42	1.18
Ph3	-2966.480192	-573.792229	-2966.482028	-573.792192	-	1.13	0.47
Ph4	-2966.486168	-573.792439	-2966.487819	-573.792368	-	0.99	0.61

Energies

In Table S8 are listed the energies and energy corrections for all of the structures computed with (*R*)-**BINAP** as ligand and 2-methyltetralone **4a** as substrate.

**Table S8**	**opt + freq**			**SPE**		
**structure**	**BP86/lanl2dz:6-31G(d)**			**SDD:6-311+G(d,p)[CPCM=Toluene]**		
	**E**	**Hcorr**	**Gcorr**	**BP86**	**PBE**	**TPSSh**
[((*R*)-**BINAP**)Pd]	-2505.50194150	0.645987	0.528996	-2507.19639233	-2504.57007027	-2507.14769252
PhBr (**5**)	-244.82573739	0.094689	0.056703	-245.10688894	-244.77687486	-245.09746355
[(**BINAP**)Pd-(PhBr)]_conf1	-2750.34604949	0.741903	0.607218	-2752.32133407	-2749.37052295	-2752.26508422
[(**BINAP**)Pd-(PhBr)]_conf2	-2750.34703676	0.741846	0.607086	-2752.32254868	-2749.37160323	-2752.26630520
**TS$_{OA}$**	-2750.33498279	0.740830	0.606365	-2752.30947726	-2749.35771736	-2752.25220046
[(BINAP)Pd(Br)(Ph)] (**7**)	-2750.37965399	0.743060	0.608177	-2752.36040903	-2749.40843212	-2752.30950866
**4a**-NaEnolate_conf1 (**8**)	-501.23140858	0.197984	0.147053	-663.48431931	-662.73173855	-663.52126523
**4a**-NaEnolate_conf2 (**8**)	-501.22969023	0.198015	0.146458	-	-	-
**7**+**8** complex_conf1	-3251.66077517	0.943755	0.777580	-3415.86269126	-3412.16501410	-3415.85233240
**7**+**8** complex_conf2	-3251.66177552	0.943587	0.777945	-3415.86407138	-3412.16658453	-3415.85359853
**TS$_{TM}$CR**	-3251.62783577	0.943156	0.781916	-3415.82811850	-3412.13348627	-3415.81500115
**TS$_{TM}$CS**	-3251.63620574	0.943464	0.784140	-3415.83547777	-3412.14024253	-3415.82343749
**TS$_{TM}$CO**_conf1	-3251.63959047	0.942858	0.782067	-3415.84025717	-3412.14357566	-3415.83055371
**TS$_{TM}$CO**_conf2	-3251.63477679	0.942809	0.782527	-3415.83573334	-3412.13949148	-3415.82675822
(*R*)-**10** + NaBr	-3251.64563317	0.944876	0.781988	-3415.85327888	-3412.15675314	-3415.84478280
(*S*)-**10** + NaBr	-3251.65272842	0.944761	0.783595	-3415.85567119	-3412.15962441	-3415.84728552
**9** + NaBr_conf1	-3251.66327289	0.944224	0.779260	-3415.86545614	-3412.16846200	-3415.85716428
**9** + NaBr_conf2	-3251.65721049	0.943132	0.780967	-3415.86222778	-3412.16534435	-3415.85379060
NaBr	-13.47239052	0.004384	-0.023108	-175.80581701	-175.64999057	-175.77845729
$[(BINAP)PdPh]^+$	-2736.94755408	0.740887	0.610339	-2738.72373639	-2735.80455526	-
Br^-	-13.26460136	0.002360	-0.016176	-13.55930755	-13.52294441	-13.52496010
**4a**-Enolate$^-$	-501.03317610	0.193434	0.146891	-501.23059130	-500.59713233	-
**TSηR**	-3238.12129570	0.937371	0.787577	-3240.01878538	-3236.47356608	-3240.03326027
**TSηS**	-3238.12241282	0.937070	0.785689	-3240.01917834	-3236.47360155	-3240.03489161
**9**_conf1	-3238.13745481	0.938088	0.783279	-3240.04136946	-3236.49420447	-3240.05684598
**9**_conf2	-3238.13800574	0.937988	0.783504	-3240.04145361	-3236.49417852	-3240.05708726
(*R*)-**10**_conf1	-3238.12431725	0.938035	0.786641	-3240.02611130	-3236.48054524	-3240.04107147
(*R*)-**10**_conf2	-3238.12673653	0.938066	0.786298	-3240.02664474	-3236.48177397	-3240.04223024
(*R*)-**10**_conf3	-3238.12465464	0.937919	0.781655	-3240.02418077	-3236.47783918	-3240.03959299
(*R*)-**10**_conf4	-3238.12003570	0.938327	0.786191	-3240.02406815	-3236.47834382	-3240.03962031
(*R*)-**10**_conf5	-3238.12673743	0.938065	0.786279	-3240.02664403	-3236.48177293	-3240.04222902
(*R*)-**10**_conf6	-3238.12654551	0.938513	0.788833	-3240.02734985	-3236.48292936	-3240.04388486
(*R*)-**10**_conf7	-3238.12081255	0.937985	0.786862	-3240.02458122	-3236.47893761	-3240.03996932
(*S*)-**10**_conf1	-3238.12343272	0.938097	0.785334	-3240.02460893	-3236.47844402	-3240.03981950
(*S*)-**10**_conf2	-3238.12350785	0.938362	0.787170	-3240.02460791	-3236.47844949	-3240.03993378
(*S*)-**10**_conf3	-3238.13051586	0.938474	0.786516	-3240.02906258	-3236.48381724	-3240.04443412
(*S*)-**10**_conf4	-3238.11941908	0.937750	0.783884	-3240.02403733	-3236.47824295	-3240.03946102
(*S*)-**11**_conf1	-3238.12545465	0.938041	0.784572	-3240.02322149	-3236.47709614	-3240.03894157
(*S*)-**11**_conf2	-3238.12761022	0.937593	0.779856	-3240.02802241	-3236.48066348	-3240.04374280
(*R*)-**11**_conf1	-3238.12569093	0.937942	0.785092	-3240.02305496	-3236.47765382	-3240.03890756
(*R*)-**11**_conf2	-3238.13681105	0.937925	0.781320	-3240.03719144	-3236.49003547	-3240.05278659
**TS$_{RE}$OR**	-3238.09791487	0.936073	0.785168	-3239.99667879	-3236.45020574	-3240.00649755
**TS$_{RE}$OS**	-3238.09675491	0.936011	0.783059	-3239.99582280	-3236.44929642	-3240.00535187
**TS$_{RE}$CR**_conf1	-3238.09168313	0.936142	0.783466	-3239.99213155	-3236.44682567	-3240.00396793
**TS$_{RE}$CR**_conf2	-3238.09565284	0.936367	0.783794	-3239.99316929	-3236.44907851	-3240.00545738

**TS$_{RE}$CR**_conf3	-3238.09746890	0.935477	0.784671	-3239.99390203	-3236.44948459	-3240.00601096
**TS$_{RE}$CR**_conf4	-3238.09289468	0.936323	0.783198	-3239.99283026	-3236.44743312	-3240.00460029
**TS$_{RE}$CR**_conf5	-3238.10048249	0.937079	0.789194	-3239.99874793	-3236.45515760	-3240.01169868
**TS$_{RE}$CR**_conf6	-3238.10331674	0.936476	0.786708	-3240.00264135	-3236.45860353	-3240.01539710
**TS$_{RE}$CR**_conf7	-3238.11237130	0.9370950	0.7891270	-3240.009982500	-3236.465836520	-3240.022637230
**TS$_{RE}$CS**_conf1	-3238.09172821	0.936107	0.782102	-3239.99221209	-3236.44671210	-3240.00408543
**TS$_{RE}$CS**_conf2	-3238.10408832	0.936005	0.789213	-3240.00190535	-3236.45786900	-3240.01495813
**TS$_{RE}$CS**_conf3	-3238.10426855	0.937258	0.788248	-3240.00220564	-3236.45825275	-3240.01535733
**TS$_{RE}$CS**_conf4	-3238.09276753	0.936105	0.782425	-3239.99414266	-3236.44833453	-3240.00576787
**TS$_{RE}$CS**_conf5	-3238.10149251	0.936727	0.784126	-3239.99772037	-3236.452032810	-3240.007911660
**TS$_{RE}$CS**_conf6	-3238.10034400	0.937155	0.784222	-3239.99615310	-3236.451128990	-3240.007708980
**TS$_{RE}$CS**_conf7	-3238.10628020	0.936831	0.786121	-3240.00488870	-3236.46061052	-3240.01722736
**TS$_{RE}$ηR**	-3238.10022532	0.936756	0.781269	-3239.99803828	-3236.45146989	-3240.01078112
**TS$_{RE}$ηS**	-3238.09200840	0.936646	0.782074	-3239.99101252	-3236.44478815	-3240.00428720
[(BINAP)Pd-((*R*)-**6a**)]_conf1	-3238.17347652	0.939233	0.784427	-3240.07166528	-3236.52480499	-3240.08554273
[(BINAP)Pd-((*R*)-**6a**)]_conf2	-3238.17942156	0.939487	0.785334	-3240.07605762	-3236.52940213	-3240.08919816
[(BINAP)Pd-((*R*)-**6a**)]_conf3	-3238.16472560	0.938906	0.785484	-3240.06135268	-3236.51650344	-3240.07620959
[(BINAP)Pd-((*S*)-**6a**)]_conf1	-3238.16363526	0.939298	0.781951	-3240.06119012	-3236.51497829	-3240.07503543
[(BINAP)Pd-((*S*)-**6a**)]_conf2	-3238.17304524	0.939398	0.785134	-3240.07052369	-3236.52424080	-3240.08397449
[(BINAP)Pd-((*S*)-**6a**)]_conf3	-3238.17686873	0.939537	0.784097	-3240.07399785	-3236.52680242	-3240.08701998
**6a**_conf1	-732.66738987	0.292024	0.234264	-732.85834200	-731.93240933	-732.91878552
**6a**_conf2	-732.66413829	0.291986	0.234065	-	-	-

In Table S9 are listed the energies and energy corrections for all of the structures computed with (*R*)-**Difluorphos** as ligand or 2-methylindanone **4a** as substrate. The conformational analysis was performed *ex novo* for the (*R*)-**BINAP/4a** system.

**Table S9**	**opt + freq**			**SPE**		
**structure**	**BP86/lanl2dz:6-31G(d)**			**SDD:6-311+G(d,p)[CPCM=Toluene]**		
	**E**	**Hcorr**	**Gcorr**	**BP86**	**PBE**	**TPSSh**
**TS$_{RE}$CR^{ind}**_conf1	-3198.80310377	0.907509	0.760890	-3200.69090459	-3197.20107118	-3200.69826895
**TS$_{RE}$CR^{ind}**_conf2	-3198.78400476	0.906871	0.757567	-	-	-
**TS$_{RE}$CR^{ind}**_conf3	-3198.78961603	0.907004	0.757580	-	-	-
**TS$_{RE}$CR^{ind}**_conf4	-3198.78340782	0.906886	0.755771	-	-	-
**TS$_{RE}$CR^{ind}**_conf5	-3198.79390566	0.906771	0.758511	-	-	-
**TS$_{RE}$CS^{ind}**_conf1	-3198.78598439	0.906946	0.756157	-	-	-
**TS$_{RE}$CS^{ind}**_conf2	-3198.79422339	0.907271	0.758923	-	-	-
**TS$_{RE}$CS^{ind}**_conf3	-3198.78746210	0.907580	0.759582	-	-	-
**TS$_{RE}$CS^{ind}**_conf4	-3198.79580853	0.907343	0.759473	-	-	-
**TS$_{RE}$CS^{ind}**_conf5	-3198.79778100	0.907091	0.758759	-3200.68691936	-3197.19747071	-3200.69523490
**TS$_{RE}$CS^{ind}**_conf6	-3198.78592200	0.906854	0.756755	-	-	-
**TS$_{RE}$CS^{ind}**_conf7	-3198.79615782	0.907238	0.757203	-3200.68333354	-3197.19329654	-3200.69025906
**TS$_{RE}$CS^{ind}**_conf8	-3198.79422314	0.907272	0.758922	-	-	-
**TS$_{RE}$CR^{dif}**_conf1	-3704.909292	0.845572	0.689633	-3706.980397	-3703.089259	-3706.930528
**TS$_{RE}$CR^{dif}**_conf2	-3704.920585	0.846548	0.693405	-3706.990216	-3703.099124	-3706.940459
**TS$_{RE}$CS^{dif}**_conf1	-3704.906895	0.846425	0.690118	-3706.974886	-3703.083369	-3706.924593
**TS$_{RE}$CS^{dif}**_conf2	-3704.912606	0.846437	0.690988	-3706.985615	-3703.094252	-3706.935538

Geometries

[((*R*)-**BINAP**)Pd]

Symbol	X	Y	Z
P	-1.90677	-0.7149	-0.732
C	-1.24384	-0.47354	1.01226
C	-3.17458	-2.06569	-0.53316
C	-2.96259	0.77199	-1.0509
C	-0.19141	0.42028	1.32047
C	-1.74579	-1.33587	2.0369
C	-2.73175	-3.38838	-0.76186
C	-4.53616	-1.84995	-0.22993
C	-3.65304	1.46484	-0.03337
C	-3.11319	1.19148	-2.38959
C	0.34748	1.44652	0.34981
C	0.3483	0.44553	2.66197
C	-1.23176	-1.32528	3.32086
H	-2.56035	-2.02508	1.79716
C	-3.61851	-4.47047	-0.66303
H	-1.68028	-3.55235	-1.03377
C	-5.4273	-2.93296	-0.14716
H	-4.90599	-0.83182	-0.06812
C	-4.48961	2.54622	-0.35289
H	-3.53013	1.159	1.01174
C	-3.95312	2.26968	-2.70811
H	-2.55954	0.6662	-3.17744
C	1.33691	1.17091	-0.61973
C	-0.15482	2.79812	0.48532
C	-0.17462	-0.44385	3.672
C	1.40026	1.337	3.04358
H	-1.64262	-1.99715	4.0847
C	-4.97154	-4.24448	-0.35722
H	-3.25638	-5.48962	-0.84101
H	-6.48364	-2.74851	0.08094
C	-4.64397	2.94749	-1.69007
H	-5.01732	3.08019	0.44562
H	-4.06123	2.58613	-3.75182
P	1.94334	-0.54478	-1.04828
C	1.75344	2.21577	-1.50874
C	0.31047	3.83724	-0.4034
C	-1.0989	3.16801	1.49501
C	0.36514	-0.41089	4.99216
H	1.81039	2.02143	2.29577
C	1.90166	1.34932	4.33675
H	-5.67012	-5.08661	-0.29391
H	-5.29491	3.79395	-1.93693
C	3.64601	-0.25278	-1.75736
C	2.37369	-1.44338	0.52227
C	1.2582	3.50094	-1.40896
H	2.48155	1.98494	-2.29203
C	-0.17771	5.16849	-0.25566
H	-1.46062	2.40349	2.18758
C	-1.5486	4.4746	1.62062
H	-0.04718	-1.09763	5.74159
C	1.38252	0.4689	5.32435
H	2.70582	2.04614	4.59954
C	4.57178	0.70969	-1.2924
C	4.03275	-1.10541	-2.81299
C	3.38628	-1.02316	1.41025
C	1.69504	-2.65154	0.78485
H	1.59904	4.27936	-2.10274
H	0.1894	5.93783	-0.94616
C	-1.08892	5.48847	0.73857
H	-2.26582	4.72623	2.41029
H	1.78647	0.48889	6.34283
C	5.85066	0.80495	-1.86254
H	4.28575	1.40638	-0.49612
C	5.31614	-1.01788	-3.37669
H	3.31163	-1.84011	-3.19256
C	3.7067	-1.79254	2.53839
H	3.93584	-0.09597	1.22041
C	2.01545	-3.42171	1.91377
H	0.90894	-2.96999	0.08818
H	-1.45296	6.51633	0.84662
C	6.228	-0.06202	-2.90239
H	6.55493	1.55994	-1.49383
H	5.5989	-1.69127	-4.19393
C	3.02324	-2.99361	2.79213
H	4.49216	-1.45191	3.22249
H	1.47871	-4.35811	2.10436
H	7.2272	0.01456	-3.34606
H	3.27856	-3.59459	3.67248
Pd	-0.04816	-1.18894	-1.95232

PhBr (**5**)

Symbol	X	Y	Z
C	0.809490	-1.225976	0.000002
C	2.215101	-1.214912	-0.000004
C	2.919052	0.000010	0.000001
C	2.215071	1.214924	0.000000
C	0.809475	1.225971	-0.000004
C	0.133728	-0.000016	0.000005
H	0.253964	-2.167824	0.000013
H	2.756674	-2.167440	-0.000011
H	4.014184	0.000037	0.000012
H	2.756652	2.167449	-0.000001
H	0.253900	2.167789	0.000009
Br	-1.847054	-0.000001	0.000000

[(**BINAP**)Pd-(PhBr)]_conf1

Symbol	X	Y	Z
P	-0.107803	1.816893	-0.14665
C	-1.643806	1.178110	-0.99581
C	0.259080	3.393502	-1.05802
C	-0.680840	2.394310	1.511965
C	-2.333751	0.042690	-0.51929
C	-2.040389	1.767685	-2.2365
C	1.016410	3.285955	-2.24835
C	-0.118929	4.677407	-0.6108
C	-1.914583	3.050698	1.715081
C	0.181186	2.194159	2.611067
C	-2.035715	-0.570189	0.82399
C	-3.418028	-0.511569	-1.29698
C	-3.079207	1.24973	-2.98738
H	-1.512319	2.654355	-2.59747
C	1.350426	4.428175	-2.99167
H	1.356307	2.298397	-2.58159
C	0.23507	5.82123	-1.34559
H	-0.685705	4.788412	0.319073
C	-2.266632	3.516588	2.991144
H	-2.603995	3.186477	0.874029
C	-0.17306	2.661861	3.88648
H	1.121246	1.649422	2.457712
C	-0.913183	-1.386314	1.065627
C	-2.969436	-0.305879	1.900965
C	-3.789328	0.097398	-2.55154
C	-4.14605	-1.667963	-0.87531
H	-3.36838	1.727839	-3.93142
C	0.961771	5.701018	-2.54087
H	1.930451	4.323598	-3.91571
H	-0.062488	6.81104	-0.9804
C	-1.394395	3.327993	4.076491
H	-3.228549	4.019793	3.140048
H	0.503218	2.497376	4.732873
P	0.471781	-1.664504	-0.14218
C	-0.670365	-1.870456	2.393443
C	-2.723193	-0.845863	3.216251
C	-4.14932	0.480584	1.718501
C	-4.855083	-0.460326	-3.31776
H	-3.878263	-2.143473	0.072509
C	-5.177969	-2.18821	-1.64312
H	1.232984	6.595246	-3.11343
H	-1.673497	3.690489	5.072387
C	1.076594	-3.37063	0.298645
C	-0.241336	-2.00529	-1.82353
C	-1.544302	-1.614054	3.430976
H	0.23178	-2.458597	2.587466
C	-3.651181	-0.596293	4.269204
H	-4.355147	0.908953	0.734031
C	-5.03814	0.702107	2.761675
H	-5.12167	0.018931	-4.26774
C	-5.540133	-1.581314	-2.87612
H	-5.719387	-3.075127	-1.29466
C	0.236191	-4.426563	0.719854
C	2.4518	-3.626753	0.12648

C	-1.193844	-3.013558	-2.08117
C	0.30094	-1.283462	-2.90701
H	-1.337472	-2.003871	4.435133
H	-3.441695	-1.018065	5.2599
C	-4.79304	0.158933	4.050326
H	-5.937571	1.303729	2.587443
H	-6.358239	-2.000225	-3.47273
C	0.762356	-5.705865	0.953843
H	-0.831312	-4.245594	0.887169
C	2.976838	-4.909543	0.352781
H	3.110347	-2.807169	-0.17868
C	-1.601456	-3.285164	-3.39503
H	-1.617488	-3.593131	-1.25502
C	-0.104179	-1.558652	-4.22313
H	1.049146	-0.507456	-2.69844
H	-5.501675	0.340144	4.866243
C	2.133438	-5.951852	0.766383
H	0.098782	-6.513437	1.284395
H	4.049266	-5.089168	0.216647
C	-1.05607	-2.56023	-4.4686
H	-2.347669	-4.065563	-3.58148
H	0.327133	-0.991341	-5.05586
H	2.542215	-6.951979	0.950085
H	-1.372659	-2.779287	-5.49483
Pd	1.762662	0.331243	-0.06629
C	3.910268	0.170153	0.193697
C	4.621472	-0.427986	-0.89449
C	3.614046	1.584175	0.172544
C	4.954567	0.338577	-2.00553
H	4.900789	-1.484854	-0.83982
C	4.024501	2.342204	-0.97525
H	3.402892	2.114128	1.107135
C	4.662077	1.731298	-2.04636
H	5.466288	-0.137436	-2.85003
H	3.857404	3.424851	-0.97144
H	4.977313	2.326082	-2.91103
Br	4.212086	-0.679198	2.035316

[(**BINAP**)Pd-(PhBr)]_conf2

Symbol	X	Y	Z
P	-0.961673	-1.052070	-0.5701
C	0.743008	-1.716724	-0.97171
C	-1.999278	-1.695009	-1.97282
C	-1.497635	-2.081791	0.869784
C	1.859011	-1.437718	-0.15404
C	0.933790	-2.434990	-2.19292
C	-2.060949	-0.902188	-3.14235
C	-2.756175	-2.884009	-1.92227
C	-1.135273	-3.438860	1.019101
C	-2.329307	-1.480758	1.8374
C	1.735268	-0.763329	1.188921
C	3.173548	-1.871360	-0.56975
C	2.184755	-2.864975	-2.59681
H	0.065519	-2.652289	-2.82103
C	-2.834692	-1.300252	-4.24195
H	-1.50064	0.041146	-3.17551
C	-3.545063	-3.273085	-3.018
H	-2.740965	-3.503526	-1.01995
C	-1.610931	-4.183292	2.109441
H	-0.476354	-3.911385	0.281629
C	-2.803484	-2.228509	2.926881
H	-2.598567	-0.423676	1.730896
C	1.563778	0.628274	1.335385
C	1.841188	-1.594457	2.370277
C	3.336533	-2.592871	-1.80912
C	4.345251	-1.603621	0.205336
H	2.300114	-3.423879	-3.53374
C	-3.582568	-2.488556	-4.18131
H	-2.865861	-0.67458	-5.14146
H	-4.135299	-4.194822	-2.95801
C	-2.448153	-3.579855	3.063001
H	-1.322689	-5.235214	2.217385
H	-3.450277	-1.751443	3.671675
P	1.188548	1.771579	-0.07779
C	1.411015	1.182511	2.64845
C	1.734634	-1.000969	3.681987
C	2.059406	-3.005642	2.298073
C	4.63737	-3.017271	-2.2121
H	4.242658	-1.053187	1.144832
C	5.59783	-2.028033	-0.2137
H	-4.199872	-2.795169	-5.03328
H	-2.816711	-4.162108	3.915304
C	1.856914	3.420671	0.476415
C	2.370604	1.422655	-1.46897
C	1.499525	0.399043	3.782084
H	1.220367	2.254767	2.754136
C	1.861571	-1.819522	4.842084
H	2.135881	-3.481241	1.31675
C	2.184171	-3.776299	3.445557
H	4.738561	-3.566088	-3.15647
C	5.749719	-2.744233	-1.43187
H	6.478413	-1.810215	0.401413
C	2.986287	3.60335	1.306381
C	1.213413	4.562239	-0.04715
C	3.773746	1.483653	-1.3311
C	1.820038	1.198108	-2.74714
H	1.389998	0.851738	4.775252
H	1.777364	-1.34469	5.827351
C	2.087652	-3.182822	4.731959
H	2.357489	-4.85479	3.356563
H	6.743913	-3.077184	-1.75055
C	3.453221	4.893754	1.60248
H	3.495075	2.735184	1.739837
C	1.688993	5.852467	0.236666
H	0.33185	4.426606	-0.68416
C	4.604863	1.314993	-2.44783
H	4.222618	1.669951	-0.35008
C	2.651875	1.032767	-3.86634
H	0.728492	1.153531	-2.84887
H	2.187535	-3.801779	5.630609
C	2.809249	6.021271	1.065716
H	4.326852	5.017881	2.252978
H	1.178772	6.726236	-0.18474
C	4.046109	1.092053	-3.71803
H	5.69301	1.35848	-2.32608
H	2.208893	0.858882	-4.85364
H	3.177809	7.027081	1.296788
H	4.698473	0.966163	-4.58969
Pd	-1.10179	1.313724	-0.42511
C	-4.133837	1.67744	0.396471
C	-4.140486	2.338117	1.612343
C	-3.252866	2.008703	-0.6763
C	-3.240202	3.427906	1.799772
H	-4.8383	2.049186	2.403082
C	-2.38294	3.150592	-0.48429
H	-3.478733	1.641199	-1.68235
C	-2.399894	3.838262	0.773816
H	-3.244147	3.959983	2.757764
H	-1.969028	3.658482	-1.36444
H	-1.749315	4.709118	0.911605
Br	-5.457065	0.229735	0.088979

TS$_{OA}$

Symbol	X	Y	Z
P	-0.249613	-1.74065	0.539824
C	-1.678864	-0.76158	1.24304
C	0.157761	-2.93592	1.908193
C	-1.022405	-2.83635	-0.73419
C	-2.266619	0.298159	0.520526
C	-2.105277	-1.01893	2.582265
C	1.191069	-2.57823	2.802627
C	-0.489594	-4.17848	2.080993
C	-2.362491	-3.27425	-0.66009
C	-0.204844	-3.28727	-1.79191
C	-1.919589	0.563532	-0.92026
C	-3.281130	1.111432	1.147298
C	-3.085418	-0.25499	3.189804
H	-1.648623	-1.84229	3.138462
C	1.553734	-3.42962	3.857579
H	1.724162	-1.63164	2.649119
C	-0.117034	-5.03535	3.129736
H	-1.280222	-4.48322	1.387307
C	-2.869618	-4.16396	-1.61998
H	-3.010102	-2.91282	0.146669
C	-0.715458	-4.17965	-2.74778
H	0.82746	-2.92426	-1.86825

C	-0.723537	1.194131	-1.31463
C	-2.869083	0.140976	-1.9308
C	-3.694386	0.829941	2.501247
C	-3.897418	2.211941	0.473898
H	-3.401816	-0.47939	4.215921
C	0.90052	-4.66224	4.022958
H	2.356183	-3.13444	4.543347
H	-0.62341	-6.00056	3.245646
C	-2.045678	-4.62137	-2.66192
H	-3.912429	-4.49511	-1.55703
H	-0.072299	-4.52217	-3.56622
P	0.676591	1.64821	-0.18365
C	-0.416567	1.304523	-2.71236
C	-2.560827	0.324156	-3.32881
C	-4.126993	-0.45393	-1.60291
C	-4.696482	1.639543	3.113645
H	-3.591305	2.442142	-0.55079
C	-4.867963	2.982662	1.097129
H	1.189446	-5.33381	4.839407
H	-2.444423	-5.31398	-3.41196
C	1.311871	3.256995	-0.87971
C	0.009204	2.314057	1.418472
C	-1.30439	0.892344	-3.68449
H	0.551572	1.716441	-3.01303
C	-3.503564	-0.07469	-4.32
H	-4.385723	-0.60713	-0.55179
C	-5.029298	-0.82524	-2.5907
H	-4.9983	1.40871	4.142583
C	-5.27638	2.695209	2.428001
H	-5.324989	3.820213	0.557949
C	0.595526	4.107119	-1.75167
C	2.580731	3.675953	-0.41865
C	-0.785571	3.479066	1.483344
C	0.428959	1.704351	2.617592
H	-1.045891	0.993748	-4.74546
H	-3.244429	0.072929	-5.37553
C	-4.720628	-0.63532	-3.96312
H	-5.989655	-1.2702	-2.3062
H	-6.046582	3.309334	2.908011
C	1.142717	5.333484	-2.16257
H	-0.393654	3.811701	-2.11674
C	3.120941	4.906255	-0.82359
H	3.144224	3.033311	0.267095
C	-1.165168	4.009741	2.724288
H	-1.098168	3.980923	0.561446
C	0.053367	2.239602	3.860455
H	1.057663	0.80706	2.560788
H	-5.439841	-0.93415	-4.73385
C	2.40629	5.736907	-1.70199
H	0.57302	5.978634	-2.84162
H	4.105489	5.214634	-0.45349
C	-0.744294	3.392993	3.915265
H	-1.788033	4.910488	2.761725
H	0.389465	1.756654	4.785106
H	2.83069	6.694428	-2.02427
H	-1.034865	3.815636	4.883914
Pd	1.718715	-0.50298	-0.084
C	3.80582	-0.37475	-0.35218
C	4.578953	0.806339	-0.41433
C	4.097811	-1.39056	0.592537
C	5.539953	1.031128	0.577892
H	4.397738	1.538992	-1.20548
C	5.053998	-1.1245	1.598924
H	3.630952	-2.3776	0.51436
C	5.769624	0.078787	1.596395
H	6.116718	1.963913	0.561463
H	5.269522	-1.89598	2.347738
H	6.53682	0.265024	2.355833
Br	3.202697	-1.2525	-2.36868

[(**BINAP**)Br-Pd-Ph] (**7**)

Symbol	X	Y	Z
P	0.500603	1.696942	0.337179
C	1.820804	0.593436	1.080082
C	0.385215	3.074612	1.572435
C	1.331833	2.463365	-1.12596
C	2.244308	-0.588472	0.439384
C	2.326189	0.900917	2.381026
C	-0.645752	3.016806	2.533997
C	1.287295	4.158431	1.598075
C	2.727250	2.678149	-1.16093
C	0.538357	2.887499	-2.21319
C	1.802092	-0.918099	-0.95911
C	3.154393	-1.484059	1.111417
C	3.221249	0.066306	3.026237
H	2.001877	1.821216	2.873396
C	-0.754371	4.006619	3.521896
H	-1.384435	2.209377	2.482225
C	1.170531	5.153663	2.581781
H	2.074517	4.233765	0.840589
C	3.317401	3.320115	-2.2606
H	3.354093	2.333947	-0.3306
C	1.135588	3.526822	-3.31108
H	-0.546972	2.739909	-2.1792
C	0.494347	-1.339984	-1.24874
C	2.756013	-0.771193	-2.03938
C	3.651711	-1.147257	2.423639
C	3.576541	-2.720603	0.530745
H	3.602962	0.331716	4.019661
C	0.154559	5.076771	3.548297
H	-1.565077	3.953042	4.256987
H	1.872215	5.995562	2.586184
C	2.522168	3.746486	-3.33687
H	4.401336	3.480252	-2.27837
H	0.509524	3.856227	-4.14788
P	-0.891418	-1.453621	-0.01848
C	0.09047	-1.525415	-2.61217
C	2.341165	-1.018449	-3.39943
C	4.115085	-0.389384	-1.81694
C	4.551756	-2.035599	3.083664
H	3.200004	-2.995903	-0.4588
C	4.447389	-3.567497	1.200944
H	0.062855	5.857511	4.311904
H	2.984581	4.246281	-4.19575
C	-1.80552	-2.984263	-0.54619
C	-0.30864	-2.019224	1.645859
C	0.985539	-1.373773	-3.65187
H	-0.946724	-1.799216	-2.83024
C	3.286042	-0.894675	-4.45947
H	4.450975	-0.186158	-0.79669
C	5.013862	-0.283083	-2.86921
H	4.92245	-1.760496	4.078546
C	4.945759	-3.222805	2.48702
H	4.755199	-4.510058	0.734342
C	-1.158085	-4.037577	-1.23242
C	-3.140917	-3.16307	-0.12543
C	0.314018	-3.271804	1.83684
C	-0.632138	-1.2323	2.769453
H	0.658635	-1.527642	-4.68717
H	2.94874	-1.087433	-5.485
C	4.601278	-0.539405	-4.20342
H	6.051987	0.00386	-2.66729
H	5.637022	-3.897877	3.003736
C	-1.836926	-5.236263	-1.5017
H	-0.122423	-3.921946	-1.56827
C	-3.813004	-4.365854	-0.39462
H	-3.66234	-2.360476	0.403263
C	0.624515	-3.714914	3.129851
H	0.546916	-3.907833	0.976813
C	-0.328744	-1.682961	4.064204
H	-1.130233	-0.267346	2.616151
H	5.320042	-0.451099	-5.02557
C	-3.166948	-5.403317	-1.08424
H	-1.32116	-6.040233	-2.03919
H	-4.852178	-4.482361	-0.06843
C	0.300704	-2.923833	4.245614
H	1.114964	-4.684919	3.266601
H	-0.588736	-1.06343	4.92973
H	-3.697078	-6.338502	-1.29727
H	0.534687	-3.279138	5.255543
Pd	-1.728379	0.698914	-0.08004
C	-3.646379	0.072222	-0.39245
C	-4.095771	-0.285665	-1.6765
C	-4.547659	0.030503	0.688943
C	-5.429176	-0.690324	-1.87558
H	-3.415001	-0.238888	-2.53448
C	-5.879227	-0.381449	0.488178
H	-4.222737	0.330282	1.691977
C	-6.322655	-0.746575	-0.79391

H	-5.767504	-0.961293	-2.88348
H	-6.57165	-0.407353	1.338987
H	-7.360492	-1.063396	-0.94991
Br	-2.709265	3.061677	-0.41517

2-Me-Tetralone-NaEnolate_conf1 (**8**)

Symbol	X	Y	Z
C	-0.932829	1.983189	0.416696
C	-1.588027	0.722239	-0.12562
C	0.489132	2.173175	-0.14169
H	-1.543441	2.870761	0.154414
H	-0.883322	1.983105	1.533165
C	-0.828156	-0.41684	-0.42157
C	-3.077895	0.742089	-0.3938
C	1.313568	0.906293	-0.02537
H	1.002450	3.018407	0.355582
H	0.405579	2.438058	-1.21745
O	-1.330480	-1.58172	-0.78078
C	0.661230	-0.33943	-0.21419
H	-3.745685	0.656869	0.511032
H	-3.351142	-0.07022	-1.0958
H	-3.397071	1.697377	-0.85602
C	2.700529	0.930586	0.196273
C	1.420681	-1.5261	-0.20111
H	3.202402	1.896197	0.343019
C	3.449507	-0.2596	0.22385
H	0.888929	-2.46674	-0.38197
C	2.806499	-1.49092	0.016422
H	4.530941	-0.22178	0.4011
H	3.385875	-2.4223	0.022715
Na	-2.576101	-1.57037	0.954277

2-Me-Tetralone-NaEnolate_conf2 (**8**)

Symbol	X	Y	Z
C	-1.430244	1.897445	0.425989
C	-1.897206	0.555565	-0.10011
C	-0.035862	2.297207	-0.08687
H	-2.153202	2.691083	0.146594
H	-1.428917	1.897211	1.545027
C	-1.005792	-0.44192	-0.48653
C	-3.373355	0.26708	-0.05541
C	0.950178	1.150671	-0.02519
H	0.352654	3.18035	0.454863
H	-0.134352	2.594794	-1.15261
O	-1.340561	-1.67884	-0.81527
C	0.468079	-0.14996	-0.34496
H	-3.791254	0.368958	0.972511
H	-3.563451	-0.75786	-0.416
H	-3.956222	0.971712	-0.68505
C	2.314994	1.338455	0.237275
C	1.40612	-1.20595	-0.47382
H	2.675292	2.344423	0.490112
C	3.230646	0.268402	0.160757
H	1.038647	-2.16085	-0.87698
C	2.776097	-1.00167	-0.22081
H	4.293003	0.439568	0.369104
H	3.486495	-1.82877	-0.3437
Na	-0.592376	-2.37835	1.076024

7+**8** complex_conf1

Symbol	X	Y	Z
P	-1.074469	-1.68812	0.289648
C	-2.155229	-0.89939	1.595181
C	0.098988	-2.72441	1.273702
C	-2.207336	-2.88591	-0.54478
C	-3.175283	0.006628	1.246051
C	-1.886984	-1.15958	2.973458
C	1.302894	-2.12341	1.703307
C	-0.146129	-4.07556	1.596908
C	-3.215126	-3.57068	0.170772
C	-2.035617	-3.14845	-1.92003
C	-3.536391	0.241726	-0.19367
C	-3.917775	0.684783	2.278942
C	-2.604873	-0.53156	3.976343
H	-1.097965	-1.86895	3.23786
C	2.236567	-2.84996	2.458114
H	1.550026	-1.08599	1.447271
C	0.792503	-4.8028	2.347547
H	-1.061788	-4.56669	1.25245
C	-4.024458	-4.51575	-0.47784
H	-3.371061	-3.36013	1.234923
C	-2.851474	-4.09188	-2.56423
H	-1.248288	-2.62816	-2.47475
C	-2.705796	0.971191	-1.06032
C	-4.762132	-0.33576	-0.70497
C	-3.624087	0.410249	3.665251
C	-4.93273	1.647174	1.983426
H	-2.385679	-0.75026	5.028418
C	1.981188	-4.19281	2.782364
H	3.161949	-2.34134	2.749017
H	0.593784	-5.85419	2.585572
C	-3.842619	-4.77894	-1.84573
H	-4.801994	-5.04326	0.086161
H	-2.7064	-4.29081	-3.63189
P	-1.039579	1.659229	-0.61402
C	-3.030672	1.058759	-2.45426
C	-5.099512	-0.18143	-2.0999
C	-5.669472	-1.06509	0.123797
C	-4.355244	1.089536	4.684674
H	-5.162536	1.870748	0.937258
C	-5.621588	2.296162	2.997758
H	2.7111	-4.76734	3.364358
H	-4.476991	-5.51623	-2.35074
C	-1.000394	3.284436	-1.51913
C	-0.957112	2.273196	1.125284
C	-4.192492	0.50635	-2.95523
H	-2.349699	1.583105	-3.13179
C	-6.31917	-0.72709	-2.5958
H	-5.426107	-1.21169	1.179259
C	-6.851508	-1.58238	-0.38758
H	-4.120783	0.86761	5.732727
C	-5.335368	2.014637	4.361634
H	-6.394757	3.030742	2.74592
C	-2.194102	3.979194	-1.82493
C	0.237301	3.914636	-1.77125
C	-1.834646	3.273119	1.599509
C	0.107044	1.840736	1.941948
H	-4.428097	0.594222	-4.02257
H	-6.556672	-0.5962	-3.65834
C	-7.185937	-1.4102	-1.75682
H	-7.531883	-2.13207	0.27236
H	-5.889394	2.530982	5.153388
C	-2.147506	5.26357	-2.38854
H	-3.166867	3.516116	-1.63154
C	0.276633	5.200347	-2.3337
H	1.173227	3.405554	-1.52924
C	-1.661471	3.808706	2.882602
H	-2.648518	3.641841	0.966648
C	0.28316	2.388923	3.222991
H	0.80975	1.08713	1.567698
H	-8.122691	-1.82273	-2.14746
C	-0.911748	5.87704	-2.64778
H	-3.083072	5.783783	-2.62344
H	1.247356	5.669198	-2.52801
C	-0.601526	3.369713	3.695246
H	-2.353472	4.57637	3.245772
H	1.122277	2.052251	3.840517
H	-0.876398	6.879053	-3.08989
H	-0.461617	3.800728	4.692953
Pd	0.291785	-0.1647	-1.09753
C	1.705891	0.978963	-2.03314
C	1.70607	1.184744	-3.42607
C	2.774507	1.4856	-1.2594
C	2.762777	1.883311	-4.0394
H	0.894387	0.784191	-4.04335
C	3.841002	2.17129	-1.88361
H	2.808155	1.341437	-0.17024
C	3.833512	2.376351	-3.27295
H	2.751221	2.033397	-5.12608
H	4.664437	2.543904	-1.26201
H	4.656118	2.915841	-3.75635
Br	1.531352	-2.24386	-2.15662
Na	3.73275	-1.07775	-0.701
O	3.869349	-0.31438	1.375406

C	5.069192	0.236518	1.466143
C	5.362323	1.454996	2.066934
C	6.223487	-0.53967	0.892027
C	6.788297	1.969393	2.123212
C	4.300974	2.21848	2.810686
C	7.463405	0.090723	0.589775
C	6.093553	-1.9276	0.637603
C	7.58532	1.574264	0.867536
H	6.789046	3.074104	2.218557
H	7.323166	1.594373	3.028533
H	4.541716	2.316755	3.892656
H	3.336425	1.690881	2.72101
H	4.171978	3.255431	2.432922
C	8.504755	-0.65931	0.022602
H	5.170786	-2.42926	0.960691
C	7.146723	-2.67045	0.074207
H	8.649931	1.864474	0.954623
H	7.169882	2.136451	0.00203
H	9.45397	-0.1596	-0.21249
C	8.355225	-2.03405	-0.24585
H	7.026313	-3.74728	-0.09761
H	9.183228	-2.60156	-0.68605

7+**8** complex_conf2

Symbol	X	Y	Z
P	0.913698	-1.5329	-0.71211
C	1.472234	-0.2601	-1.96201
C	-0.411801	-2.46037	-1.61149
C	2.343430	-2.69995	-0.61998
C	2.508442	0.646004	-1.66505
C	0.788995	-0.16387	-3.21215
C	-1.725239	-1.93801	-1.58153
C	-0.166792	-3.66174	-2.30783
C	3.114369	-3.01008	-1.76278
C	2.649927	-3.31966	0.609458
C	3.286428	0.530709	-0.38457
C	2.859053	1.671957	-2.61612
C	1.125425	0.80418	-4.14162
H	-0.015063	-0.86956	-3.43693
C	-2.769374	-2.59253	-2.25581
H	-1.964086	-1.02446	-1.02351
C	-1.214582	-4.3189	-2.974
H	0.838361	-4.09428	-2.32167
C	4.161633	-3.93993	-1.67839
H	2.896726	-2.52062	-2.71886
C	3.701972	-4.24583	0.688694
H	2.051026	-3.08793	1.495862
C	2.722967	0.854241	0.861431
C	4.645803	0.03262	-0.43946
C	2.154995	1.747889	-3.87387
C	3.873563	2.644921	-2.3539
H	0.590688	0.856921	-5.09756
C	-2.512831	-3.78508	-2.95436
H	-3.78032	-2.16592	-2.21089
H	-1.011236	-5.2556	-3.5057
C	4.455517	-4.56077	-0.45291
H	4.751931	-4.17561	-2.57106
H	3.928524	-4.72391	1.648206
P	0.960729	1.365178	1.156009
C	3.463384	0.607405	2.064384
C	5.393019	-0.14994	0.782043
C	5.291973	-0.29637	-1.67087
C	2.499842	2.766836	-4.81074
H	4.412357	2.605737	-1.40238
C	4.18101	3.62846	-3.28283
H	-3.327034	-4.302	-3.47513
H	5.274976	-5.28559	-0.38855
C	1.125447	2.659343	2.484144
C	0.264497	2.396745	-0.20368
C	4.75731	0.128322	2.025411
H	2.994351	0.813017	3.031553
C	6.738475	-0.61617	0.723938
H	4.740536	-0.18504	-2.60789
C	6.604645	-0.74629	-1.69579
H	1.955724	2.807987	-5.76196
C	3.493113	3.690288	-4.52552
H	4.962722	4.362696	-3.0578
C	2.299431	3.440138	2.598069
C	0.013484	2.979865	3.291424
C	0.93881	3.5499	-0.6616
C	-1.03361	2.113899	-0.67685
H	5.309597	-0.04263	2.95718
H	7.28819	-0.74467	1.664088
C	7.339997	-0.90398	-0.49145
H	7.07673	-0.98475	-2.65539
H	3.747976	4.469305	-5.25253
C	2.362283	4.503006	3.512111
H	3.173917	3.215865	1.978988
C	0.082016	4.046394	4.20192
H	-0.90764	2.397369	3.214505
C	0.333982	4.388862	-1.60633
H	1.936078	3.79358	-0.2812
C	-1.639825	2.965649	-1.61617
H	-1.589	1.245338	-0.30067
H	8.376018	-1.25874	-0.52484
C	1.254502	4.808032	4.318895
H	3.282135	5.093638	3.590229
H	-0.789906	4.275102	4.824349
C	-0.955909	4.097771	-2.08377
H	0.870963	5.27311	-1.96676
H	-2.656447	2.745676	-1.95851
H	1.305445	5.637218	5.033453
H	-1.431994	4.761293	-2.81457
Pd	-0.020414	-0.71076	1.420096
C	-1.047167	-0.14679	3.099814
C	-0.516833	-0.37901	4.384135
C	-2.348992	0.39026	2.978779
C	-1.276199	-0.08527	5.531241
H	0.48417	-0.8098	4.499307
C	-3.111468	0.669475	4.135643
H	-2.793947	0.604506	1.99438
C	-2.575705	0.437762	5.412186
H	-0.850241	-0.27554	6.524144
H	-4.120848	1.084772	4.02331
H	-3.164722	0.663729	6.308523
Br	-0.752764	-3.18272	2.012597
Na	-3.252145	-1.86034	1.561651
O	-4.063795	-0.33118	0.260366
C	-5.133724	-0.33986	-0.50965
C	-5.941584	-1.45477	-0.71866
C	-5.560221	0.956585	-1.13146
C	-7.210428	-1.35328	-1.54559
C	-5.687079	-2.72547	0.056397
C	-6.531874	0.987369	-2.16821
C	-5.031533	2.175755	-0.65845
C	-7.057312	-0.33453	-2.68766
H	-7.470737	-2.34169	-1.97532
H	-8.08664	-1.0621	-0.91664
H	-5.770609	-2.5842	1.163594
H	-4.684478	-3.16682	-0.14704
H	-6.419602	-3.51247	-0.20029
C	-6.944313	2.220323	-2.69817
H	-4.292974	2.133915	0.148856
C	-5.455161	3.404443	-1.19084
H	-8.012941	-0.18865	-3.22653
H	-6.331225	-0.74574	-3.423
H	-7.69503	2.232282	-3.49965
C	-6.414608	3.430937	-2.21608
H	-5.040864	4.341947	-0.79948
H	-6.755036	4.385418	-2.63495

$\mathbf{TS_{TM}CR}$

Symbol	X	Y	Z
C	2.877083	4.843097	0.176979
C	2.899086	5.171696	1.541866
C	2.324784	4.287625	2.466877
C	1.742292	3.079951	2.032233
C	1.740428	2.728661	0.666324
C	2.291965	3.639814	-0.26183
H	3.310428	5.527075	-0.56353
H	3.348433	6.112977	1.879569
H	2.323385	4.530334	3.5371
H	1.293614	2.422689	2.78253
H	2.272511	3.410455	-1.33437
Pd	1.017006	0.974842	-0.13601
C	4.203843	0.299382	0.737646

C	4.304335	-1.07243	0.31468
C	4.011059	0.576882	2.213695
C	5.000761	1.348979	-0.01374
O	4.718161	-1.43118	-0.84785
C	4.017308	-2.14772	1.324209
C	3.055635	-0.42041	2.875213
H	3.644719	1.607505	2.365495
H	5.005305	0.524721	2.718902
H	5.530358	0.914763	-0.88293
H	4.398312	2.217972	-0.33847
H	5.788463	1.761854	0.651165
C	3.4687	-1.84454	2.597817
C	4.365171	-3.48093	1.016329
H	2.967389	-0.23952	3.962947
H	2.050629	-0.25334	2.42312
C	3.297325	-2.87931	3.536357
H	4.784578	-3.67834	0.025118
C	4.186472	-4.50245	1.957888
H	2.889783	-2.63923	4.527108
C	3.655351	-4.20173	3.225487
H	4.469292	-5.5327	1.711424
H	3.528826	-4.99348	3.973427
P	-1.144903	1.662437	0.505437
C	-2.616581	0.802287	-0.27001
C	-1.37145	3.43766	0.030723
C	-1.604539	1.530381	2.295584
C	-2.866048	-0.57463	-0.0844
C	-3.504672	1.579158	-1.07463
C	-0.969475	3.802332	-1.27589
C	-1.869902	4.431349	0.896797
C	-2.8765	1.955775	2.743489
C	-0.716697	0.945394	3.217893
C	-1.999524	-1.42996	0.801377
C	-4.011109	-1.17892	-0.72476
C	-4.614608	1.014029	-1.67506
H	-3.306778	2.644745	-1.21386
C	-1.117906	5.123971	-1.71931
H	-0.504723	3.060889	-1.93825
C	-1.997811	5.758235	0.452323
H	-2.143198	4.182624	1.925961
C	-3.222189	1.849644	4.099027
H	-3.603604	2.358855	2.029949
C	-1.068699	0.830866	4.572319
H	0.242501	0.555134	2.862817
C	-0.683719	-1.78425	0.445717
C	-2.53568	-1.8753	2.071272
C	-4.897011	-0.37112	-1.52761
C	-4.299485	-2.57466	-0.61011
H	-5.288221	1.634474	-2.27851
C	-1.636321	6.105564	-0.85784
H	-0.800517	5.387463	-2.73412
H	-2.377912	6.521802	1.140485
C	-2.315008	1.296003	5.01792
H	-4.207796	2.19099	4.435122
H	-0.367989	0.369823	5.276911
P	0.149955	-1.13127	-1.0762
C	0.143365	-2.4747	1.389066
C	-1.715541	-2.66944	2.955754
C	-3.863551	-1.56281	2.498727
C	-6.028053	-0.97377	-2.15343
H	-3.630046	-3.20746	-0.02079
C	-5.405839	-3.13309	-1.23346
H	-1.739229	7.141138	-1.20117
H	-2.588518	1.210285	6.075425
C	1.253741	-2.47467	-1.76395
C	-1.088002	-1.19254	-2.46155
C	-0.362862	-2.92362	2.593978
H	1.203572	-2.63321	1.170316
C	-2.258591	-3.14735	4.184264
H	-4.495977	-0.94109	1.860044
C	-4.363181	-2.04073	3.70218
H	-6.690505	-0.34125	-2.75657
C	-6.283526	-2.32819	-2.00887
H	-5.603608	-4.20576	-1.12817
C	1.451855	-3.76708	-1.23995
C	1.852291	-2.15065	-3.00891
C	-1.693997	-2.42245	-2.80351
C	-1.310661	-0.06319	-3.27101
H	0.289144	-3.46747	3.287478
H	-1.61949	-3.75558	4.835883
C	-3.561059	-2.84772	4.551808
H	-5.387722	-1.79038	3.999553
H	-7.155558	-2.78087	-2.49384
C	2.255958	-4.69682	-1.92407
H	0.96733	-4.07445	-0.31001
C	2.65564	-3.08095	-3.68415
H	1.6392	-1.17695	-3.47688
C	-2.527796	-2.51141	-3.92574
H	-1.503207	-3.31553	-2.19801
C	-2.142171	-0.15939	-4.39994
H	-0.795023	0.874932	-3.0447
H	-3.969577	-3.22304	5.496737
C	2.868851	-4.35834	-3.13779
H	2.394884	-5.69669	-1.49772
H	3.096887	-2.81208	-4.65129
C	-2.753939	-1.37843	-4.72705
H	-2.997834	-3.46846	-4.17786
H	-2.296877	0.723641	-5.02986
H	3.494788	-5.08741	-3.66391
H	-3.39779	-1.45203	-5.61095
Br	1.866242	1.711341	-3.34028
Na	3.636181	-0.07609	-2.19001

TS$_{TM}$CS

Symbol	X	Y	Z
C	-3.940890	0.400891	3.458108
C	-4.652557	1.522296	3.001131
C	-4.319373	2.077604	1.756584
C	-3.294116	1.516419	0.971284
C	-2.577943	0.379451	1.403033
C	-2.906642	-0.15306	2.675495
H	-4.166168	-0.03822	4.438522
H	-5.450470	1.961577	3.611135
H	-4.864569	2.949528	1.37592
H	-3.084532	1.972915	0.000728
H	-2.349530	-1.01337	3.065866
Pd	-1.060927	-0.52989	0.30213
P	0.406992	0.965507	1.436076
C	2.266695	0.708566	1.403564
C	0.030803	0.954451	3.248911
C	0.301836	2.737777	0.915345
C	2.99688	0.709959	0.198789
C	2.958601	0.528126	2.641994
C	0.105781	-0.28899	3.919571
C	-0.385523	2.093596	3.965407
C	1.064695	3.735888	1.56388
C	-0.52139	3.099243	-0.16704
C	2.36686	0.962411	-1.14296
C	4.428659	0.500118	0.232014
C	4.328738	0.354377	2.68844
H	2.395784	0.538947	3.577515
C	-0.184363	-0.37731	5.28823
H	0.370318	-1.19439	3.361747
C	-0.692608	1.996332	5.333249
H	-0.486396	3.057509	3.458676
C	0.9658	5.075035	1.158176
H	1.737192	3.466216	2.38606
C	-0.61213	4.438793	-0.57782
H	-1.076892	2.316722	-0.6928
C	1.550783	0.013134	-1.78837
C	2.673209	2.212815	-1.80843
C	5.100946	0.32514	1.496013
C	5.222262	0.435292	-0.95637
H	4.833566	0.231852	3.654409
C	-0.582442	0.767194	6.000351
H	-0.117549	-1.34789	5.792118
H	-1.021979	2.889891	5.875498
C	0.12221	5.429519	0.091271
H	1.554051	5.842795	1.673607
H	-1.259994	4.702921	-1.4206
P	0.842654	-1.4838	-0.9391
C	1.038102	0.305047	-3.09322
C	2.180862	2.457154	-3.14276
C	3.458168	3.236803	-1.19263
C	6.512364	0.123202	1.526087
H	4.735279	0.54729	-1.92876
C	6.593099	0.228924	-0.89686
H	-0.818825	0.696595	7.068096
H	0.048264	6.47632	-0.22421

C	0.669969	-2.77575	-2.27068
C	2.215464	-2.32195	-0.00802
C	1.359712	1.472473	-3.75813
H	0.379422	-0.41812	-3.58113
C	2.515311	3.672252	-3.80991
H	3.818881	3.088226	-0.17161
C	3.760708	4.412979	-1.86386
H	7.001253	-0.00227	2.499751
C	7.250064	0.077913	0.353997
H	7.174296	0.182866	-1.82472
C	1.4322	-2.80454	-3.45951
C	-0.16186	-3.8741	-1.96261
C	3.473836	-2.53674	-0.60998
C	1.943896	-2.89312	1.25023
H	0.971952	1.655943	-4.76764
H	2.136527	3.831495	-4.82686
C	3.295911	4.633513	-3.18777
H	4.362927	5.180537	-1.36507
H	8.333953	-0.07873	0.387804
C	1.334222	-3.89469	-4.33881
H	2.108125	-1.97884	-3.70495
C	-0.246801	-4.96644	-2.84046
H	-0.726521	-3.87999	-1.02246
C	4.454375	-3.2897	0.04977
H	3.691074	-2.11604	-1.59773
C	2.927094	-3.65664	1.902888
H	0.946737	-2.7834	1.693039
H	3.547713	5.563895	-3.70884
C	0.492046	-4.97633	-4.03414
H	1.925931	-3.89929	-5.26154
H	-0.894802	-5.81226	-2.58481
C	4.182725	-3.85217	1.309094
H	5.431553	-3.43923	-0.4228
H	2.698862	-4.10759	2.875254
H	0.419371	-5.82701	-4.72121
H	4.947255	-4.44945	1.819191
Br	-1.663004	-3.39423	1.698315
C	-3.25884	-1.40884	-1.76309
C	-4.369221	-0.61447	-1.30027
C	-2.531186	-0.92105	-3.00469
C	-3.407789	-2.91529	-1.61634
O	-5.235558	-1.04977	-0.46104
C	-4.580898	0.738677	-1.90637
C	-2.307889	0.59407	-2.9766
H	-1.57002	-1.4531	-3.12053
H	-3.133346	-1.18425	-3.90721
H	-4.479781	-3.19021	-1.54708
H	-2.894581	-3.34257	-0.73099
H	-2.97549	-3.43058	-2.49254
C	-3.60354	1.331278	-2.74725
C	-5.792248	1.420082	-1.65751
H	-1.813373	0.955022	-3.89718
H	-1.610104	0.795281	-2.12821
C	-3.860791	2.593253	-3.31551
H	-6.522507	0.933146	-1.0036
C	-6.038557	2.671584	-2.2336
H	-3.108013	3.047173	-3.97317
C	-5.069039	3.262482	-3.06557
H	-6.985051	3.189691	-2.0394
H	-5.258559	4.239589	-3.52551
Na	-4.073744	-2.00832	1.148928

TS$_{TM}$CO_conf1

Symbol	X	Y	Z
P	-0.456265	-1.65869	0.167737
C	-1.805277	-1.28408	1.423456
C	0.658884	-2.87391	1.013417
C	-1.398326	-2.73395	-1.0168
C	-2.943526	-0.53257	1.058631
C	-1.656741	-1.71516	2.775262
C	1.470483	-2.43068	2.082067
C	0.795129	-4.20527	0.565571
C	-2.242463	-3.74627	-0.50199
C	-1.257311	-2.59798	-2.41033
C	-3.194433	-0.16908	-0.37984
C	-3.917678	-0.17659	2.061177
C	-2.595745	-1.40064	3.742868
H	-0.789312	-2.32269	3.048617
C	2.347994	-3.31455	2.727037
H	1.428844	-1.38726	2.402114
C	1.691065	-5.0815	1.199624
H	0.206702	-4.56219	-0.28356
C	-2.916184	-4.61708	-1.3694
H	-2.367605	-3.85694	0.581206
C	-1.941094	-3.47032	-3.27485
H	-0.591874	-1.83099	-2.81669
C	-2.403662	0.772435	-1.0622
C	-4.242143	-0.86621	-1.09953
C	-3.739594	-0.61911	3.423214
C	-5.067928	0.616804	1.75887
H	-2.465609	-1.75795	4.771858
C	2.460628	-4.64366	2.288225
H	2.965243	-2.95133	3.555877
H	1.783899	-6.11147	0.83665
C	-2.765401	-4.48127	-2.76044
H	-3.56283	-5.40051	-0.95789
H	-1.816881	-3.35664	-4.35762
P	-1.030801	1.738755	-0.2761
C	-2.536208	0.91784	-2.4817
C	-4.432021	-0.61444	-2.50828
C	-5.116523	-1.80513	-0.47025
C	-4.712741	-0.27415	4.407718
H	-5.209891	0.973108	0.734303
C	-5.995934	0.937525	2.738655
H	3.156792	-5.33019	2.783506
H	-3.293038	-5.16214	-3.43831
C	-1.174894	3.436629	-1.03664
C	-1.554491	2.199925	1.447971
C	-3.526735	0.256184	-3.17942
H	-1.842277	1.569646	-3.0214
C	-5.494438	-1.25914	-3.20531
H	-4.974524	-2.04042	0.587228
C	-6.146652	-2.41355	-1.17424
H	-4.562463	-0.6238	5.436379
C	-5.822797	0.486117	4.07537
H	-6.869939	1.546145	2.480346
C	-2.325908	3.890779	-1.71959
C	-0.141445	4.361828	-0.76795
C	-2.64118	3.075579	1.667642
C	-0.801305	1.755471	2.549227
H	-3.623709	0.394466	-4.26291
H	-5.621068	-1.04863	-4.27418
C	-6.346986	-2.13552	-2.55158
H	-6.808088	-3.1203	-0.66064
H	-6.565024	0.74341	4.839312
C	-2.42323	5.225004	-2.14874
H	-3.157761	3.208439	-1.91692
C	-0.245692	5.694382	-1.19278
H	0.750531	4.040477	-0.22348
C	-2.967317	3.483087	2.968834
H	-3.225356	3.453453	0.822103
C	-1.125516	2.168443	3.850806
H	0.042282	1.081955	2.375761
H	-7.164356	-2.62136	-3.09602
C	-1.382351	6.129879	-1.89214
H	-3.322974	5.554247	-2.6811
H	0.572133	6.391083	-0.9786
C	-2.209232	3.034172	4.063178
H	-3.815052	4.15905	3.126848
H	-0.525391	1.814155	4.696111
H	-1.459232	7.169647	-2.2296
H	-2.461733	3.36267	5.077615
Pd	0.844488	0.390693	-0.51667
C	1.991068	1.986474	-1.10585
C	1.942628	2.440325	-2.44419
C	2.925418	2.575511	-0.22645
C	2.813029	3.453393	-2.8908
H	1.245042	1.979364	-3.1501
C	3.797185	3.590506	-0.679
H	3.011139	2.230483	0.809398
C	3.742411	4.034999	-2.01041
H	2.761219	3.784782	-3.93521
H	4.517017	4.03135	0.022327
H	4.416024	4.826489	-2.359
Br	1.812031	-0.99963	-2.95235
Na	3.905995	0.11113	-1.52074
O	2.939569	-0.51731	0.481254
C	4.007843	-0.23756	1.217577
C	4.051965	0.457953	2.420207

C	5.313332	-0.79333	0.701581
C	5.371174	0.65824	3.144685
C	2.804302	0.938465	3.103893
C	6.562316	-0.27561	1.145451
C	5.309553	-1.85436	-0.23793
C	6.539532	0.846202	2.161902
H	5.303563	1.54403	3.806916
H	5.596384	-0.20168	3.817809
H	2.594256	0.376077	4.040801
H	1.941454	0.814118	2.429729
H	2.864461	2.006885	3.398995
C	7.755179	-0.80044	0.623407
H	4.344864	-2.29354	-0.52271
C	6.512745	-2.36994	-0.75179
H	7.507919	0.907193	2.693242
H	6.399951	1.814889	1.634581
H	8.713432	-0.39127	0.969318
C	7.740385	-1.83794	-0.32742
H	6.486676	-3.19886	-1.46901
H	8.683255	-2.23618	-0.71945

TS$_{TM}$CO_conf2

Symbol	X	Y	Z
P	-0.460478	-1.68241	0.180987
C	-1.647913	-1.13677	1.53306
C	0.640688	-2.92078	1.004742
C	-1.603794	-2.73909	-0.8308
C	-2.742233	-0.29439	1.240268
C	-1.417616	-1.53832	2.882684
C	1.574395	-2.47029	1.965882
C	0.637486	-4.28459	0.645793
C	-2.454089	-3.65725	-0.17014
C	-1.626471	-2.66663	-2.23571
C	-3.095014	0.044131	-0.18279
C	-3.581957	0.187152	2.310186
C	-2.231377	-1.10619	3.915734
H	-0.585411	-2.2126	3.103659
C	2.44092	-3.37505	2.595783
H	1.638427	-1.40714	2.213597
C	1.522245	-5.18442	1.263446
H	-0.051372	-4.64828	-0.12143
C	-3.298024	-4.49845	-0.90816
H	-2.451532	-3.71792	0.923974
C	-2.480263	-3.50863	-2.96952
H	-0.957165	-1.97438	-2.75477
C	-2.292367	0.883641	-0.97458
C	-4.270478	-0.56828	-0.77046
C	-3.320325	-0.22635	3.667945
C	-4.673702	1.081282	2.080513
H	-2.040746	-1.44169	4.942592
C	2.416621	-4.73572	2.246759
H	3.15131	-3.00702	3.343989
H	1.506998	-6.24059	0.971107
C	-3.312716	-4.42535	-2.31204
H	-3.948236	-5.2093	-0.38543
H	-2.482449	-3.44584	-4.06342
P	-0.750152	1.713109	-0.37238
C	-2.559505	1.011856	-2.37654
C	-4.580391	-0.33365	-2.16069
C	-5.156802	-1.40533	-0.02519
C	-4.158037	0.246746	4.721193
H	-4.87792	1.416018	1.059258
C	-5.469213	1.526045	3.12622
H	3.102162	-5.44052	2.731325
H	-3.974609	-5.08218	-2.88821
C	-0.797331	3.408389	-1.14323
C	-1.012757	2.240696	1.39379
C	-3.674954	0.433509	-2.94798
H	-1.868907	1.584184	-3.00363
C	-5.763603	-0.89174	-2.72543
H	-4.926806	-1.62789	1.01944
C	-6.305296	-1.93072	-0.60136
H	-3.945832	-0.0823	5.745711
C	-5.21522	1.10384	4.459522
H	-6.300686	2.210282	2.922672
C	-1.957553	3.979275	-1.71154
C	0.35372	4.215231	-0.99955
C	-1.959735	3.240616	1.709108
C	-0.202716	1.71979	2.419619
H	-3.872929	0.558333	-4.01925
H	-5.980748	-0.69568	-3.78242
C	-6.620396	-1.6678	-1.96007
H	-6.971684	-2.56011	-0.00092
H	-5.853362	1.459439	5.276222
C	-1.954578	5.314822	-2.14885
H	-2.873164	3.388731	-1.81204
C	0.349709	5.549383	-1.43037
H	1.259681	3.797439	-0.55095
C	-2.094769	3.69684	3.028047
H	-2.582653	3.677494	0.921978
C	-0.337234	2.1798	3.739257
H	0.534277	0.94823	2.180834
H	-7.530868	-2.08695	-2.40269
C	-0.801939	6.102642	-2.01316
H	-2.863694	5.73773	-2.59152
H	1.255586	6.154416	-1.31468
C	-1.282603	3.170407	4.046149
H	-2.835487	4.470493	3.258821
H	0.30341	1.760997	4.52296
H	-0.801989	7.143505	-2.35583
H	-1.385628	3.534379	5.074547
Pd	0.930078	0.189507	-0.81152
C	2.063969	1.613144	-1.77356
C	1.700069	1.989886	-3.08727
C	3.268744	2.129575	-1.24275
C	2.517692	2.843627	-3.84877
H	0.787997	1.585369	-3.53625
C	4.092486	2.982097	-2.01395
H	3.58124	1.885333	-0.22249
C	3.720543	3.342378	-3.31817
H	2.212643	3.110949	-4.86781
H	5.020222	3.369523	-1.57408
H	4.356379	4.007765	-3.9136
Br	1.462854	-1.42694	-3.309
Na	3.793104	-0.48188	-2.13283
O	2.92376	-0.95024	-0.072
C	4.115061	-0.84789	0.503143
C	5.228675	-1.56246	0.057813
C	4.289063	0.040744	1.70422
C	6.586645	-1.3736	0.716397
C	5.124845	-2.60633	-1.03281
C	5.436336	-0.06884	2.538282
C	3.300107	0.987259	2.040923
C	6.45731	-1.14447	2.230274
H	7.219492	-2.26406	0.534066
H	7.140747	-0.51715	0.266289
H	5.673473	-2.34687	-1.97259
H	4.071726	-2.82866	-1.28481
H	5.584816	-3.56125	-0.70781
C	5.566115	0.774576	3.6537
H	2.418837	1.056811	1.392202
C	3.440134	1.830047	3.155441
H	7.435071	-0.89218	2.68282
H	6.123548	-2.09466	2.70045
H	6.455744	0.680195	4.290092
C	4.580382	1.727716	3.966323
H	2.660119	2.565925	3.383645
H	4.705755	2.382617	4.836463

(*R*)-**10** + NaBr

Symbol	X	Y	Z
C	0.234781	-4.91867	-2.74344
C	1.084727	-5.79123	-2.0446
C	1.564603	-5.41061	-0.78264
C	1.187312	-4.17637	-0.21769
C	0.313665	-3.2984	-0.89457
C	-0.138893	-3.68703	-2.17567
H	-0.140577	-5.19188	-3.73722
H	1.374562	-6.75333	-2.48275
H	2.238642	-6.07411	-0.22599
H	1.593747	-3.91321	0.763969
H	-0.790687	-3.02207	-2.75509
Pd	-0.316435	-1.4411	-0.22238
C	-2.191228	-2.52661	0.456588
C	-2.814619	-1.60503	1.434301
C	-1.894033	-3.878	1.137589

C	-3.046454	-2.6825	-0.80328
O	-3.739451	-0.80797	1.118679
C	-2.431342	-1.689	2.88387
C	-1.016408	-3.72161	2.38594
H	-1.417601	-4.57157	0.427747
H	-2.862856	-4.34183	1.434876
H	-4.058544	-3.06601	-0.54445
H	-3.184296	-1.72721	-1.3397
H	-2.593547	-3.40589	-1.49974
C	-1.594912	-2.72805	3.364519
C	-2.982438	-0.75411	3.790032
H	-0.848637	-4.6963	2.8819
H	-0.022851	-3.35438	2.056231
C	-1.324734	-2.79953	4.744368
H	-3.635986	0.027034	3.389208
C	-2.692579	-0.82847	5.15711
H	-0.693229	-3.61344	5.122436
C	-1.861795	-1.85892	5.636212
H	-3.121452	-0.09738	5.851721
H	-1.645119	-1.93767	6.70802
P	1.912317	-0.70833	-0.99296
C	2.406693	1.084432	-1.31295
C	2.360223	-1.54638	-2.58509
C	3.224117	-1.2111	0.211867
C	2.279182	2.063563	-0.30872
C	2.922536	1.464804	-2.59015
C	1.65102	-1.19319	-3.75729
C	3.307297	-2.58764	-2.65513
C	4.595391	-1.05882	-0.09186
C	2.850879	-1.70605	1.476605
C	1.860486	1.703862	1.084412
C	2.60328	3.440485	-0.59762
C	3.273638	2.773498	-2.86988
H	3.056188	0.706632	-3.36459
C	1.913485	-1.83856	-4.97408
H	0.897787	-0.39828	-3.72355
C	3.555919	-3.24217	-3.87296
H	3.842702	-2.90585	-1.75669
C	5.572047	-1.42978	0.844264
H	4.900168	-0.65171	-1.06246
C	3.830294	-2.06756	2.415591
H	1.785086	-1.78628	1.722785
C	0.556135	1.288154	1.390109
C	2.859715	1.758562	2.134718
C	3.109246	3.799087	-1.89969
C	2.413318	4.486336	0.359932
H	3.678532	3.032067	-3.85583
C	2.869325	-2.866	-5.0364
H	1.362876	-1.54126	-5.87377
H	4.291245	-4.05376	-3.90553
C	5.191038	-1.93874	2.097597
H	6.633229	-1.31514	0.596023
H	3.5271	-2.44538	3.398152
P	-0.823158	0.939387	0.196265
C	0.250094	0.851578	2.72287
C	2.498792	1.40465	3.486069
C	4.212309	2.148112	1.888748
C	3.42551	5.1611	-2.1823
H	2.01383	4.237471	1.347428
C	2.717492	5.804303	0.051025
H	3.06945	-3.37595	-5.9853
H	5.955638	-2.2237	2.828881
C	-2.283414	1.90894	0.858392
C	-0.706543	1.844743	-1.41616
C	1.180033	0.931754	3.740423
H	-0.736528	0.43061	2.939018
C	3.469364	1.498084	4.525573
H	4.521177	2.390864	0.868989
C	5.137383	2.225049	2.920958
H	3.814784	5.412934	-3.17615
C	3.235318	6.147693	-1.22773
H	2.557197	6.587727	0.800001
C	-2.324548	2.574996	2.099774
C	-3.399742	2.030417	-0.00527
C	-0.557443	3.244615	-1.5114
C	-1.014207	1.10546	-2.57832
H	0.910364	0.609425	4.753079
H	3.169003	1.232205	5.546351
C	4.76545	1.908299	4.253864
H	6.166356	2.531696	2.702416
H	3.477298	7.191463	-1.45637
C	-3.459016	3.319363	2.474214
H	-1.46828	2.540112	2.779128
C	-4.530465	2.774553	0.365435
H	-3.378602	1.572201	-1.00216
C	-0.673024	3.883035	-2.75409
H	-0.36117	3.839077	-0.61346
C	-1.153332	1.751297	-3.81941
H	-1.165482	0.020672	-2.49561
H	5.503653	1.979668	5.060341
C	-4.565649	3.414348	1.618787
H	-3.463567	3.835675	3.441312
H	-5.378781	2.83713	-0.33135
C	-0.974943	3.139804	-3.90901
H	-0.543641	4.968911	-2.8177
H	-1.417039	1.169477	-4.70966
H	-5.445134	3.998266	1.912041
H	-1.088496	3.647131	-4.87337
Br	-7.08054	0.986542	-1.90318
Na	-5.457035	-0.08586	-0.02434

(*S*)-**10** + NaBr

Symbol	X	Y	Z
C	4.083407	-0.94	-2.72197
C	4.602565	0.27934	-3.18961
C	4.121347	1.478594	-2.64349
C	3.143453	1.463083	-1.62915
C	2.616188	0.249546	-1.14222
C	3.098474	-0.95055	-1.71384
H	4.410204	-1.89035	-3.16428
H	5.363377	0.293792	-3.97884
H	4.514060	2.440478	-2.99578
H	2.817805	2.420024	-1.20769
H	2.674168	-1.91978	-1.41349
Pd	1.141411	0.107467	0.301331
P	-0.455148	-0.11995	-1.60832
C	-2.22044	-0.75976	-1.40909
C	0.2078	-1.23519	-2.93126
C	-0.753838	1.499914	-2.4491
C	-3.149557	-0.1067	-0.57623
C	-2.625357	-1.9389	-2.10708
C	0.511072	-2.57022	-2.57564
C	0.476306	-0.8026	-4.24572
C	-1.627161	1.607713	-3.55488
C	-0.104322	2.654451	-1.9711
C	-2.798868	1.178604	0.108374
C	-4.472397	-0.6551	-0.39764
C	-3.902241	-2.45529	-1.97672
H	-1.916718	-2.44216	-2.76813
C	1.040578	-3.46004	-3.52125
H	0.365325	-2.92288	-1.54867
C	1.011579	-1.69546	-5.18909
H	0.283702	0.233965	-4.53681
C	-1.818916	2.845767	-4.1855
H	-2.159525	0.721983	-3.91936
C	-0.304272	3.894138	-2.59911
H	0.548403	2.569125	-1.09591
C	-1.854783	1.224083	1.14415
C	-3.423659	2.405097	-0.34895
C	-4.854981	-1.84544	-1.11623
C	-5.426483	-0.07526	0.496728
H	-4.187133	-3.35524	-2.535
C	1.287051	-3.02527	-4.83396
H	1.289496	-4.47912	-3.20761
H	1.218721	-1.34212	-6.20577
C	-1.154533	3.989947	-3.71125
H	-2.493954	2.918401	-5.04571
H	0.205509	4.785569	-2.21686
P	-0.822825	-0.18512	1.784935
C	-1.45439	2.497973	1.67196
C	-3.067478	3.661817	0.263256
C	-4.392364	2.428548	-1.39866
C	-6.164201	-2.38389	-0.94034
H	-5.147717	0.816746	1.065458
C	-6.688187	-0.62975	0.656737
H	1.707941	-3.71669	-5.57286
H	-1.308676	4.956502	-4.20404
C	-1.069996	-0.08492	3.645449
C	-1.54587	-1.87079	1.555575

C	-2.052884	3.672918	1.262392
H	-0.657928	2.539152	2.421414
C	-3.712382	4.861494	-0.15581
H	-4.657546	1.494215	-1.89961
C	-5.0047	3.612898	-1.78587
H	-6.438337	-3.28624	-1.5001
C	-7.06696	-1.78995	-0.07257
H	-7.39917	-0.16902	1.351666
C	-1.813693	0.911358	4.313711
C	-0.517831	-1.13536	4.41821
C	-2.834176	-2.23293	2.006831
C	-0.664541	-2.87566	1.103285
H	-1.744381	4.630556	1.69908
H	-3.430301	5.804052	0.328932
C	-4.672139	4.840838	-1.15619
H	-5.750273	3.59852	-2.58872
H	-8.068447	-2.21458	0.058516
C	-1.964311	0.880097	5.711105
H	-2.299523	1.712611	3.750467
C	-0.664975	-1.16112	5.811801
H	0.001719	-1.96295	3.924337
C	-3.239407	-3.57426	1.966745
H	-3.51807	-1.4714	2.396141
C	-1.066327	-4.22241	1.083914
H	0.35581	-2.6241	0.785466
H	-5.165642	5.767968	-1.4682
C	-1.381796	-0.14651	6.467397
H	-2.550809	1.663885	6.204257
H	-0.224959	-1.98551	6.383858
C	-2.356927	-4.5704	1.508383
H	-4.245891	-3.84381	2.30619
H	-0.346748	-4.97769	0.748198
H	-1.497533	-0.16612	7.556725
H	-2.675255	-5.61923	1.497355
Br	2.797686	-4.48757	0.052645
C	2.685516	0.314118	1.927798
C	4.027612	0.618444	1.365925
C	2.218003	1.451607	2.85854
C	2.667393	-1.05569	2.626441
O	4.858815	-0.28594	1.077712
C	4.465433	2.047734	1.235005
C	2.247401	2.819364	2.163914
H	1.208844	1.237772	3.250176
H	2.888444	1.490796	3.74829
H	3.580289	-1.1973	3.243316
H	2.590895	-1.92072	1.941809
H	1.800668	-1.11776	3.30161
C	3.625921	3.121279	1.626743
C	5.762286	2.317357	0.744607
H	1.920475	3.625481	2.848768
H	1.520849	2.793943	1.320858
C	4.103053	4.440585	1.506729
H	6.385926	1.465309	0.457504
C	6.223804	3.631601	0.62916
H	3.459381	5.272433	1.820176
C	5.388878	4.699045	1.010695
H	7.231133	3.830283	0.245979
H	5.745703	5.732672	0.931597
Na	4.482199	-2.30059	0.24166

9 + NaBr_conf1

Symbol	X	Y	Z
P	0.430866	-1.46524	-0.89986
C	2.286943	-1.60064	-1.09978
C	-0.265196	-2.27695	-2.40883
C	0.075219	-2.63489	0.481175
C	3.145979	-0.98138	-0.17094
C	2.848416	-2.31686	-2.20162
C	0.118148	-1.83223	-3.69625
C	-1.283866	-3.24765	-2.3027
C	0.395219	-4.00868	0.384144
C	-0.486908	-2.1389	1.673602
C	2.644078	-0.40129	1.122904
C	4.572924	-1.00154	-0.39426
C	4.214823	-2.37316	-2.40892
H	2.185132	-2.84338	-2.89248
C	-0.465411	-2.38531	-4.84595
H	0.88091	-1.05245	-3.80392
C	-1.876834	-3.78675	-3.45581
H	-1.616705	-3.58079	-1.31601
C	0.123088	-4.86966	1.45726
H	0.845645	-4.40603	-0.53269
C	-0.753919	-3.00094	2.749757
H	-0.696819	-1.0684	1.790127
C	1.979851	0.833905	1.221627
C	2.866091	-1.18007	2.324949
C	5.112525	-1.69889	-1.53624
C	5.489386	-0.33732	0.479662
H	4.619097	-2.94001	-3.25651
C	-1.462924	-3.36832	-4.72951
H	-0.143583	-2.04114	-5.83556
H	-2.666294	-4.5395	-3.35246
C	-0.454724	-4.36784	2.637707
H	0.367009	-5.93527	1.372402
H	-1.194493	-2.57244	3.658197
P	1.294358	1.787873	-0.20609
C	1.505913	1.293458	2.493914
C	2.398314	-0.68391	3.597496
C	3.51483	-2.45405	2.305176
C	6.522147	-1.70846	-1.75439
H	5.099119	0.193683	1.353115
C	6.855229	-0.35567	0.2356
H	-1.921204	-3.79725	-5.62763
H	-0.664035	-5.04543	3.47334
C	1.46856	3.564987	0.289983
C	2.403956	1.770552	-1.68405
C	1.708306	0.559338	3.644909
H	0.953723	2.235432	2.555041
C	2.61141	-1.45256	4.778946
H	3.858018	-2.86713	1.353289
C	3.705915	-3.17968	3.472293
H	6.913119	-2.24749	-2.62588
C	7.380537	-1.04937	-0.88847
H	7.535727	0.164753	0.918811
C	2.52473	3.994186	1.124574
C	0.615707	4.526577	-0.29024
C	3.777257	2.078374	-1.5954
C	1.813157	1.597738	-2.95243
H	1.302216	0.914657	4.597626
H	2.238144	-1.0578	5.730747
C	3.257577	-2.67705	4.722542
H	4.203038	-4.15513	3.425961
H	8.461619	-1.06241	-1.06607
C	2.714536	5.360906	1.379731
H	3.1914	3.260968	1.590861
C	0.814458	5.892685	-0.03661
H	-0.217461	4.205566	-0.92215
C	4.549838	2.190768	-2.76008
H	4.246212	2.229618	-0.61777
C	2.587432	1.723105	-4.11785
H	0.740996	1.372809	-3.01424
H	3.413006	-3.26259	5.635404
C	1.860913	6.312954	0.799024
H	3.53115	5.679871	2.037157
H	0.138191	6.627977	-0.48615
C	3.956721	2.016894	-4.02237
H	5.618372	2.418324	-2.68054
H	2.117369	1.595475	-5.09957
H	2.008827	7.379467	1.002434
H	4.562173	2.115439	-4.93033
Pd	-0.704649	0.716718	-0.52481
C	-1.847227	2.342952	-0.01222
C	-1.969746	2.704262	1.348303
C	-2.624726	3.033289	-0.96654
C	-2.870609	3.715071	1.747464
H	-1.396584	2.186251	2.129772
C	-3.502295	4.060314	-0.5679
H	-2.554382	2.778221	-2.02816
C	-3.632952	4.401014	0.789188
H	-2.963467	3.95342	2.813561
H	-4.092903	4.589385	-1.32657
H	-4.323528	5.194859	1.096206
Br	-2.271774	0.154036	4.244724
Na	-3.255024	0.24124	1.650576
O	-2.499191	-0.44656	-0.47119
C	-3.599898	-0.34787	-1.25147
C	-3.691733	0.122855	-2.54221
C	-4.831422	-0.90007	-0.60177
C	-5.048276	0.142725	-3.23117

C	-2.514386	0.521649	-3.38703
C	-6.121426	-0.5299	-1.07264
C	-4.73652	-1.81366	0.476708
C	-6.189185	0.42908	-2.24049
H	-5.048366	0.916504	-4.02368
H	-5.240105	-0.82295	-3.7522
H	-2.33412	-0.2174	-4.19477
H	-1.581478	0.586375	-2.79268
H	-2.676443	1.499144	-3.88712
C	-7.263976	-1.0463	-0.44306
H	-3.745359	-2.16465	0.790507
C	-5.890646	-2.32212	1.098786
H	-7.176272	0.369668	-2.73586
H	-6.075338	1.468998	-1.86601
H	-8.254311	-0.74973	-0.81208
C	-7.159341	-1.93226	0.644991
H	-5.792296	-3.02946	1.930024
H	-8.06422	-2.32376	1.123526

9 + NaBr_conf2

Symbol	X	Y	Z
P	0.137637	0.825291	-1.31463
C	1.938596	1.353792	-1.34227
C	-0.716791	2.270005	-2.10032
C	0.051493	-0.51144	-2.58097
C	2.978638	0.497583	-0.92802
C	2.253845	2.691019	-1.73779
C	-0.959831	3.413106	-1.30638
C	-1.095893	2.285656	-3.45885
C	0.804696	-0.45197	-3.77594
C	-0.828493	-1.5934	-2.37558
C	2.761376	-0.94888	-0.57926
C	4.335748	0.996047	-0.86725
C	3.550617	3.169874	-1.71646
H	1.451714	3.350768	-2.07591
C	-1.527183	4.564935	-1.87211
H	-0.709193	3.403281	-0.24065
C	-1.677648	3.434705	-4.0182
H	-0.938733	1.40011	-4.08191
C	0.65936	-1.4549	-4.74648
H	1.502729	0.376111	-3.94519
C	-0.969895	-2.60083	-3.34338
H	-1.415193	-1.64872	-1.45321
C	2.161751	-1.3567	0.625437
C	3.251574	-1.95317	-1.50032
C	4.623053	2.350468	-1.27145
C	5.42506	0.197566	-0.39452
H	3.761988	4.196425	-2.03956
C	-1.883908	4.579359	-3.23104
H	-1.708704	5.4416	-1.24142
H	-1.969977	3.431903	-5.07436
C	-0.226953	-2.52573	-4.53221
H	1.245098	-1.40098	-5.67143
H	-1.667784	-3.42462	-3.14119
P	1.219993	-0.2376	1.756481
C	2.026956	-2.75333	0.919306
C	3.113017	-3.35329	-1.17773
C	3.877587	-1.6149	-2.74
C	5.962573	2.83762	-1.21262
H	5.232851	-0.82978	-0.07286
C	6.716199	0.702861	-0.33731
H	-2.331139	5.477345	-3.67241
H	-0.333842	-3.30632	-5.29404
C	1.480746	-0.94574	3.451235
C	2.046212	1.399733	1.996905
C	2.491083	-3.7191	0.048486
H	1.553085	-3.06043	1.855908
C	3.602228	-4.33994	-2.08299
H	3.984934	-0.56194	-3.01285
C	4.347296	-2.5978	-3.59925
H	6.157936	3.868553	-1.53163
C	6.993551	2.03245	-0.75549
H	7.530259	0.068215	0.030494
C	2.684974	-1.59395	3.806775
C	0.519996	-0.68816	4.450228
C	3.420008	1.508542	2.297466
C	1.234541	2.551608	2.038327
H	2.37944	-4.78137	0.295149
H	3.480939	-5.39702	-1.81899
C	4.212606	-3.97313	-3.27171
H	4.823242	-2.30893	-4.54292
H	8.018686	2.416716	-0.71294
C	2.911811	-1.98832	5.13423
H	3.444745	-1.80466	3.04634
C	0.754578	-1.07755	5.777814
H	-0.420526	-0.19787	4.183408
C	3.97492	2.757473	2.610698
H	4.059998	0.620558	2.282249
C	1.792189	3.798845	2.363808
H	0.16254	2.464568	1.822094
H	4.585061	-4.73896	-3.96075
C	1.948056	-1.7309	6.12278
H	3.84586	-2.4991	5.394215
H	-0.0055	-0.87729	6.540936
C	3.162605	3.903858	2.646543
H	5.045812	2.834143	2.828455
H	1.1504	4.68626	2.395759
H	2.126516	-2.04165	7.158111
H	3.599338	4.876684	2.899169
Pd	-0.855175	-0.04113	0.791309
C	-1.748807	-1.28988	2.173591
C	-1.482791	-2.67091	2.037684
C	-2.807646	-0.8931	3.021271
C	-2.262252	-3.63232	2.717075
H	-0.693509	-3.02214	1.363333
C	-3.568696	-1.85172	3.719669
H	-3.072095	0.164687	3.127084
C	-3.303048	-3.22448	3.566109
H	-2.06393	-4.6972	2.554181
H	-4.38431	-1.51897	4.373739
H	-3.910598	-3.96927	4.091732
Br	-3.635317	-4.41526	-1.24287
Na	-3.653094	-2.11343	0.250969
O	-2.878226	0.02178	0.049356
C	-3.637331	1.088956	-0.29047
C	-4.60192	0.960494	-1.27016
C	-3.553922	2.351436	0.499804
C	-5.656628	2.036784	-1.44658
C	-4.769084	-0.30925	-2.06303
C	-4.298712	3.497517	0.094631
C	-2.758476	2.456848	1.662768
C	-5.086011	3.437892	-1.1961
H	-6.077514	1.984984	-2.46905
H	-6.515263	1.85244	-0.76014
H	-5.532278	-0.99567	-1.6298
H	-3.827268	-0.8781	-2.15116
H	-5.123391	-0.0933	-3.08891
C	-4.233772	4.676645	0.853001
H	-2.163887	1.589513	1.97653
C	-2.690617	3.645951	2.40808
H	-5.885458	4.202612	-1.19724
H	-4.397356	3.689558	-2.03077
H	-4.820854	5.545544	0.52799
C	-3.436781	4.764184	2.008772
H	-2.066337	3.689348	3.308685
H	-3.407723	5.692441	2.590883

[(**BINAP**)PdPh]$^{+}$

Symbol	X	Y	Z
P	0.657184	1.795364	0.260005
C	1.669839	0.538681	1.172848
C	0.574929	3.276743	1.360054
C	1.631209	2.347338	-1.19634
C	1.884306	-0.74425	0.630754
C	2.102791	0.835638	2.499538
C	-0.547988	3.408998	2.208747
C	1.552342	4.295723	1.349345
C	3.042022	2.392312	-1.18946
C	0.925481	2.803383	-2.33044
C	1.549581	-1.03974	-0.80649
C	2.51844	-1.75867	1.433797
C	2.729443	-0.12498	3.275053
H	1.939119	1.838371	2.904864
C	-0.684813	4.529093	3.042771
H	-1.321761	2.629034	2.210887
C	1.411599	5.415427	2.183627

H	2.415499	4.221988	0.679642
C	3.73282	2.900195	-2.30055
H	3.59872	2.024878	-0.32021
C	1.620465	3.312025	-3.43798
H	-0.171171	2.761662	-2.34174
C	0.231713	-1.22563	-1.26723
C	2.638301	-1.08964	-1.7591
C	2.942168	-1.44062	2.777958
C	2.737975	-3.08614	0.95183
H	3.067742	0.121601	4.288286
C	0.296681	5.533408	3.030947
H	-1.56066	4.621197	3.69394
H	2.173469	6.202152	2.166355
C	3.02408	3.362935	-3.42212
H	4.827511	2.932209	-2.29079
H	1.065819	3.666866	-4.31322
P	-1.27172	-1.12278	-0.20424
C	-0.031146	-1.35386	-2.67127
C	2.3556	-1.27524	-3.16341
C	4.006349	-0.95979	-1.36435
C	3.567519	-2.44811	3.571362
H	2.421273	-3.34185	-0.06383
C	3.346676	-4.04357	1.749842
H	0.189008	6.412358	3.675383
H	3.56714	3.759924	-4.28649
C	-2.449015	-2.36074	-0.9046
C	-1.001652	-1.75794	1.502625
C	1.000579	-1.37956	-3.58797
H	-1.063869	-1.44622	-3.02102
C	3.429612	-1.33983	-4.09766
H	4.247361	-0.81444	-0.30837
C	5.030803	-1.03281	-2.2975
H	3.888331	-2.19145	4.587712
C	3.768112	-3.72434	3.069912
H	3.508264	-5.05417	1.359327
C	-1.964576	-3.53022	-1.539
C	-3.832477	-2.233	-0.65866
C	-0.727126	-3.12801	1.712356
C	-1.180405	-0.90606	2.610833
H	0.783361	-1.488	-4.65684
H	3.194106	-1.48406	-5.15841
C	4.745198	-1.22648	-3.67532
H	6.071216	-0.9424	-1.9672
H	4.252606	-4.48875	3.686598
C	-2.855834	-4.54139	-1.92927
H	-0.895285	-3.65151	-1.73824
C	-4.715837	-3.25048	-1.04965
H	-4.22076	-1.33717	-0.16661
C	-0.611952	-3.62462	3.017953
H	-0.616591	-3.80973	0.863248
C	-1.071424	-1.41062	3.915319
H	-1.406867	0.152923	2.438947
H	5.564118	-1.28441	-4.40006
C	-4.231995	-4.40369	-1.68774
H	-2.469172	-5.43803	-2.42541
H	-5.787757	-3.13542	-0.8576
C	-0.785507	-2.76972	4.119514
H	-0.39436	-4.68629	3.174093
H	-1.214974	-0.7419	4.770606
H	-4.925317	-5.19295	-1.99711
H	-0.70626	-3.16637	5.137284
Pd	-1.645636	1.120268	-0.1697
C	-3.626697	1.038771	-0.451
C	-4.117882	1.168723	-1.7665
C	-4.470537	1.270107	0.655824
C	-5.433925	1.624298	-1.96967
H	-3.485794	0.93199	-2.63027
C	-5.786951	1.72064	0.438352
H	-4.114053	1.112987	1.680723
C	-6.265426	1.897933	-0.87047
H	-5.810217	1.752278	-2.99108
H	-6.438691	1.920774	1.29651
H	-7.294636	2.234854	-1.03468

2-Me-Tetralone-Enolate$^-$

Symbol	X	Y	Z
C	-1.58777	1.5557	0.320977
C	-1.985087	0.124288	0.043958
C	-0.265908	1.936054	-0.37206
H	-2.380793	2.254307	-0.03225
H	-1.489761	1.777878	1.419321
C	-1.032626	-0.90178	-0.12441
C	-3.435312	-0.24919	0.171013
C	0.811782	0.900098	-0.12582
H	0.085011	2.943196	-0.06237
H	-0.459546	1.982575	-1.46668
O	-1.269764	-2.15669	-0.26824
C	0.416519	-0.46262	-0.06866
H	-3.858079	-0.05686	1.189101
H	-3.526995	-1.33147	-0.03396
H	-4.100601	0.306022	-0.53327
C	2.171769	1.237933	-0.02111
C	1.414284	-1.45203	0.065744
H	2.465966	2.297605	-0.06695
C	3.158103	0.242898	0.132856
H	1.057651	-2.48932	0.081683
C	2.770294	-1.1082	0.169575
H	4.21666	0.524077	0.218369
H	3.532301	-1.89342	0.280437

TSηR

Symbol	X	Y	Z
P	-1.184426	-1.32998	0.777224
C	-1.927488	0.252672	1.452682
C	-1.105804	-2.42601	2.279562
C	-2.607477	-2.02396	-0.18796
C	-2.232682	1.32072	0.585488
C	-2.178042	0.388488	2.851954
C	-0.08204	-2.17351	3.225512
C	-1.97651	-3.51546	2.493904
C	-3.913915	-2.00779	0.354004
C	-2.400249	-2.58737	-1.46281
C	-2.024932	1.179755	-0.89636
C	-2.807533	2.531477	1.116245
C	-2.726879	1.546123	3.374876
H	-1.940434	-0.44127	3.522728
C	0.033621	-2.96983	4.375278
H	0.650718	-1.37619	3.045213
C	-1.842321	-4.32098	3.638281
H	-2.761831	-3.74279	1.767184
C	-4.984648	-2.57087	-0.3555
H	-4.092224	-1.55072	1.334217
C	-3.477811	-3.14346	-2.1724
H	-1.398849	-2.59092	-1.90079
C	-0.739246	1.187889	-1.46595
C	-3.186285	0.993873	-1.74402
C	-3.056787	2.643837	2.533104
C	-3.142325	3.64527	0.284997
H	-2.919196	1.624935	4.451923
C	-0.846264	-4.04553	4.586784
H	0.828602	-2.75618	5.098703
H	-2.523787	-5.16707	3.784298
C	-4.766989	-3.14439	-1.61989
H	-5.991498	-2.55509	0.077385
H	-3.301507	-3.57799	-3.16252
P	0.870434	1.391043	-0.54192
C	-0.594989	0.876483	-2.85897
C	-3.011966	0.770069	-3.15863
C	-4.52216	1.035205	-1.23851
C	-3.629169	3.842604	3.052706
H	-2.952905	3.577823	-0.79031
C	-3.701475	4.7966	0.819481
H	-0.746666	-4.67211	5.480428
H	-5.604363	-3.58185	-2.17543
C	1.810891	2.475491	-1.74484
C	0.637094	2.645858	0.815397
C	-1.689282	0.679039	-3.67744
H	0.409933	0.819142	-3.28935
C	-4.154159	0.634615	-3.99996
H	-4.681254	1.178411	-0.16678
C	-5.616088	0.907205	-2.08352
H	-3.811072	3.910126	4.132203
C	-3.949556	4.899623	2.21544
H	-3.953256	5.634983	0.16012
C	1.195552	3.370726	-2.65124
C	3.218583	2.462979	-1.66682

C	0.180754	3.962458	0.587171
C	1.079656	2.285871	2.106008
H	-1.548409	0.462091	-4.74343
H	-3.996114	0.470096	-5.0729
C	-5.436334	0.710773	-3.4779
H	-6.628831	0.953635	-1.66741
H	-4.392095	5.81429	2.625691
C	1.969691	4.216762	-3.46018
H	0.104564	3.401351	-2.73842
C	3.992315	3.314136	-2.47308
H	3.71629	1.790475	-0.96315
C	0.144572	4.893182	1.634567
H	-0.137085	4.268639	-0.41475
C	1.043462	3.223767	3.152744
H	1.489214	1.283465	2.281255
H	-6.307965	0.612393	-4.13464
C	3.37178	4.191494	-3.37463
H	1.472531	4.90092	-4.15796
H	5.084236	3.28119	-2.3903
C	0.573506	4.524885	2.921582
H	-0.219504	5.909263	1.445173
H	1.393926	2.932894	4.149865
H	3.974512	4.85286	-4.0076
H	0.549718	5.25647	3.737691
Pd	1.153266	-1.33256	0.0761
C	0.951455	-2.9938	-1.06222
C	0.501806	-4.23604	-0.56868
C	1.211307	-2.8722	-2.44621
C	0.313622	-5.32876	-1.4369
H	0.278711	-4.35802	0.497568
C	1.019696	-3.96628	-3.31242
H	1.559437	-1.91849	-2.85929
C	0.571098	-5.19954	-2.81105
H	-0.042054	-6.28414	-1.03115
H	1.226776	-3.84898	-4.38374
H	0.424687	-6.05119	-3.48547
C	4.152886	-2.51087	-0.58837
C	3.190954	-2.16234	0.544682
C	4.598757	-1.28897	-1.40464
H	3.693074	-3.26393	-1.25247
H	5.054275	-2.99237	-0.14513
C	3.406315	-0.87316	1.222309
C	2.951815	-3.34073	1.485743
C	5.163846	-0.22305	-0.49731
H	5.333628	-1.57467	-2.17953
H	3.712551	-0.87575	-1.93351
O	2.721895	-0.53553	2.239168
C	4.512437	0.019017	0.740916
H	3.915184	-3.62854	1.961038
H	2.240363	-3.0965	2.287788
H	2.581118	-4.22149	0.936348
C	6.271736	0.569301	-0.84938
C	4.963114	1.05831	1.582651
H	6.782201	0.381531	-1.80231
C	6.723691	1.593328	-0.00028
H	4.433939	1.220432	2.526958
C	6.062209	1.844031	1.21585
H	7.59483	2.194405	-0.28643
H	6.409456	2.646636	1.876246

TSηS

Symbol	X	Y	Z
P	-0.217294	1.290797	0.785539
C	1.256081	0.589862	1.712603
C	-1.483447	1.559753	2.124362
C	0.41133	2.997079	0.418537
C	2.463019	0.298072	1.046134
C	1.162753	0.394323	3.124649
C	-2.180452	0.420976	2.596855
C	-1.857865	2.830573	2.609127
C	1.012958	3.767438	1.44148
C	0.314814	3.536961	-0.87894
C	2.622598	0.497757	-0.43751
C	3.599635	-0.16479	1.806981
C	2.242313	-0.05659	3.86238
H	0.221244	0.617404	3.633758
C	-3.201469	0.550398	3.548825
H	-1.937226	-0.57283	2.201487
C	-2.889827	2.957366	3.556071
H	-1.352875	3.728709	2.24186
C	1.473698	5.065206	1.176778
H	1.124551	3.348039	2.447937
C	0.788945	4.833009	-1.14224
H	-0.132257	2.944461	-1.68143
C	2.088627	-0.4158	-1.36691
C	3.371613	1.645223	-0.90546
C	3.485648	-0.34208	3.234515
C	4.855489	-0.46411	1.193106
H	2.150174	-0.19076	4.947159
C	-3.559803	1.82092	4.031917
H	-3.736503	-0.34339	3.886862
H	-3.170106	3.953977	3.916378
C	1.358551	5.601988	-0.1168
H	1.930544	5.654249	1.98047
H	0.708012	5.239064	-2.15633
P	0.962705	-1.81894	-0.92445
C	2.188858	-0.11788	-2.76753
C	3.526269	1.871515	-2.32249
C	3.989045	2.575066	-0.01346
C	4.614468	-0.79457	3.979753
H	4.95728	-0.3431	0.110874
C	5.936936	-0.8989	1.945249
H	-4.367338	1.923106	4.765681
H	1.721538	6.614998	-0.32487
C	1.351107	-3.13792	-2.18759
C	1.668304	-2.71781	0.547801
C	2.890372	0.977329	-3.23032
H	1.70708	-0.78656	-3.48796
C	4.295589	2.97911	-2.78253
H	3.868467	2.436775	1.064156
C	4.736689	3.642568	-0.49103
H	4.507964	-0.92327	5.063767
C	5.819791	-1.06584	3.351871
H	6.890106	-1.11656	1.450152
C	2.535481	-3.2386	-2.95078
C	0.389134	-4.1689	-2.29671
C	2.991167	-3.20979	0.59073
C	0.781835	-3.04139	1.596562
H	2.97021	1.16662	-4.30799
H	4.401761	3.128199	-3.86416
C	4.89877	3.848542	-1.88644
H	5.20322	4.335729	0.217967
H	6.68132	-1.40979	3.93521
C	2.739447	-4.32651	-3.81526
H	3.30604	-2.46393	-2.87541
C	0.599542	-5.25996	-3.15401
H	-0.522291	-4.11726	-1.68898
C	3.425813	-3.98792	1.672633
H	3.682954	-2.99257	-0.23021
C	1.219581	-3.82685	2.677073
H	-0.260015	-2.70724	1.540886
H	5.49283	4.694374	-2.25035
C	1.772398	-5.33907	-3.92219
H	3.6638	-4.38461	-4.40218
H	-0.158234	-6.04891	-3.22272
C	2.540424	-4.29729	2.719886
H	4.457725	-4.35619	1.697143
H	0.520075	-4.07664	3.483423
H	1.934402	-6.18696	-4.59741
H	2.879937	-4.91228	3.56147
Pd	-1.629637	-0.0312	-0.71297
C	-2.28585	1.586536	-1.72047
C	-2.980616	2.654518	-1.11207
C	-2.00617	1.668817	-3.10338
C	-3.390602	3.769071	-1.86871
H	-3.190805	2.636778	-0.03622
C	-2.408818	2.789473	-3.85543
H	-1.460264	0.860947	-3.60556
C	-3.10764	3.842311	-3.24196
H	-3.93012	4.586456	-1.374
H	-2.174575	2.833574	-4.9266
H	-3.426188	4.711932	-3.82833
C	-4.585308	-0.61425	-2.01028
C	-3.238929	-1.22936	-1.63432
C	-5.340852	0.016278	-0.82974
H	-4.442363	0.140853	-2.80434
H	-5.211805	-1.42062	-2.45551
C	-3.150909	-1.8439	-0.3025
C	-2.568597	-1.9368	-2.80316

C	-5.425749	-0.9198	0.351676
H	-6.351423	0.337728	-1.14349
H	-4.797228	0.931754	-0.52274
O	-2.087504	-2.44478	0.078415
C	-4.325195	-1.7699	0.626264
H	-3.241063	-2.7277	-3.20281
H	-1.617321	-2.40658	-2.51856
H	-2.378753	-1.23122	-3.63226
C	-6.533688	-0.9439	1.218066
C	-4.350883	-2.61779	1.754064
H	-7.388205	-0.28748	1.010859
C	-6.558107	-1.79324	2.335567
H	-3.486371	-3.26648	1.929951
C	-5.462717	-2.63417	2.605715
H	-7.435893	-1.8052	2.992358
H	-5.483483	-3.30617	3.471566

9_conf1

Symbol	X	Y	Z
C	3.935852	3.251451	-1.49446
C	4.537305	3.738677	-0.32238
C	4.132299	3.232897	0.92401
C	3.118246	2.25791	0.998038
C	2.50881	1.767263	-0.17517
C	2.9326	2.266025	-1.42195
H	4.246672	3.6376	-2.4735
H	5.320744	4.503533	-0.37953
H	4.607268	3.591761	1.845484
H	2.814663	1.878656	1.981078
H	2.486615	1.887261	-2.34859
O	2.55388	-1.16529	-0.45984
C	3.811803	-0.95444	-0.88873
C	4.149125	-0.89562	-2.22454
C	4.886468	-0.92359	0.144015
C	5.610882	-0.83513	-2.63078
C	6.205331	-0.52452	-0.20177
C	6.455775	-0.0456	-1.61645
H	5.702918	-0.36842	-3.63144
H	6.023921	-1.86441	-2.74102
H	7.532365	-0.10527	-1.8649
H	6.166644	1.024509	-1.68087
C	7.209948	-0.53501	0.777861
H	8.226632	-0.22385	0.503387
C	4.614871	-1.32087	1.468705
H	3.596879	-1.63975	1.710518
C	5.627385	-1.32618	2.439999
H	5.400179	-1.64692	3.464234
C	6.930986	-0.93347	2.097565
H	7.729639	-0.94095	2.848931
C	3.124323	-0.9988	-3.32055
H	3.188136	-1.97132	-3.8559
H	2.100684	-0.90328	-2.92252
H	3.279779	-0.21816	-4.09316
P	-0.665675	1.780177	-0.0355
C	-1.736313	0.960696	-1.31267
C	-0.580301	3.54343	-0.62791
C	-1.578512	2.067577	1.551482
C	-2.670055	-0.05925	-1.06038
C	-1.380095	1.277688	-2.66553
C	-1.628825	4.12224	-1.38
C	0.483703	4.365446	-0.199
C	-2.80448	2.764882	1.604654
C	-0.93676	1.71167	2.75404
C	-3.322136	-0.71827	-2.17395
C	-3.015031	-0.502	0.33423
C	-1.983554	0.648072	-3.73512
H	-0.614743	2.037453	-2.85119
C	-1.60425	5.486917	-1.70624
H	-2.46588	3.505674	-1.72395
C	0.499931	5.731465	-0.52388
H	1.306554	3.93983	0.381606
C	-3.384275	3.078922	2.841407
H	-3.304535	3.069174	0.679683
C	-1.513537	2.036875	3.992552
H	0.023445	1.185655	2.706099
C	-2.978269	-0.34912	-3.52637
C	-4.312386	-1.73317	-1.99375
C	-2.124063	-1.27908	1.100871
C	-4.307337	-0.15164	0.872966
H	-1.69927	0.910873	-4.76106
C	-0.53877	6.295949	-1.27967
H	-2.422712	5.916369	-2.29538
H	1.338945	6.350692	-0.18825
C	-2.738808	2.718908	4.03731
H	-4.341148	3.611539	2.871387
H	-1.002048	1.75794	4.920622
C	-3.629751	-0.98895	-4.62079
H	-4.590389	-2.03127	-0.97945
C	-4.933911	-2.33434	-3.07941
P	-0.387777	-1.63819	0.503153
C	-2.525081	-1.728	2.397374
C	-4.686882	-0.61683	2.185212
C	-5.235571	0.667691	0.157496
H	-0.519238	7.361118	-1.53668
H	-3.190082	2.976054	5.002309
H	-3.35093	-0.69343	-5.63941
C	-4.593467	-1.96212	-4.4066
H	-5.694123	-3.10496	-2.9091
C	0.362727	-2.547	1.935173
C	-0.599339	-2.97877	-0.75283
C	-3.767631	-1.41605	2.919017
H	-1.833662	-2.33529	2.987413
C	-5.963142	-0.2629	2.715057
H	-4.962554	1.034361	-0.83633
C	-6.467291	1.000533	0.70232
H	-5.089403	-2.4455	-5.25553
C	1.135769	-1.81702	2.862571
C	0.186166	-3.93315	2.133552
C	-1.771009	-3.75765	-0.8519
C	0.500612	-3.25722	-1.59393
H	-4.054034	-1.77949	3.913455
H	-6.234622	-0.62885	3.712652
C	-6.839753	0.529582	1.990867
H	-7.160474	1.630246	0.133263
C	1.698326	-2.45184	3.981205
H	1.317471	-0.75065	2.683041
C	0.757051	-4.56849	3.247808
H	-0.394315	-4.51722	1.411427
C	-1.845229	-4.80167	-1.78822
H	-2.625569	-3.54709	-0.20017
C	0.417963	-4.30444	-2.52459
H	1.411443	-2.6518	-1.49129
H	-7.81735	0.795604	2.408129
C	1.508784	-3.82948	4.176279
H	2.29941	-1.87152	4.690105
H	0.618122	-5.64673	3.386678
C	-0.751783	-5.07627	-2.62535
H	-2.761119	-5.39889	-1.86356
H	1.274248	-4.5163	-3.17496
H	1.956359	-4.32898	5.04289
H	-0.812156	-5.89163	-3.35553
Pd	1.103667	0.299556	-0.02783

9_conf2

Symbol	X	Y	Z
C	4.096924	3.367461	-0.86021
C	4.407346	4.054951	0.324229
C	3.742958	3.714342	1.514173
C	2.770825	2.694732	1.515555
C	2.45388	2.001199	0.328321
C	3.122193	2.351527	-0.8612
H	4.613133	3.620932	-1.79454
H	5.163046	4.849378	0.321317
H	3.977402	4.242383	2.447214
H	2.261546	2.445421	2.454249
H	2.905794	1.821942	-1.79497
O	2.667664	-0.88546	0.840802
C	3.956264	-0.7397	0.478879
C	4.945373	-0.43738	1.389569
C	4.318942	-0.96558	-0.95298
C	6.386805	-0.314	0.934412
C	5.675126	-1.19541	-1.318
C	6.71028	-1.2807	-0.21503
H	7.071408	-0.50527	1.784093
H	6.588822	0.732359	0.611229
H	7.72412	-1.09677	-0.61796

H	6.711631	-2.31624	0.189386
C	6.0094	-1.39951	-2.66563
H	7.059934	-1.57605	-2.93193
C	3.337616	-0.97294	-1.9682
H	2.290721	-0.80417	-1.68799
C	3.683592	-1.1772	-3.31337
H	2.901844	-1.17424	-4.08258
C	5.024464	-1.3885	-3.66936
H	5.30459	-1.54757	-4.71736
C	4.641091	-0.13698	2.829923
H	4.934356	0.90022	3.094611
H	3.567085	-0.2628	3.036063
H	5.209477	-0.80556	3.509636
P	-0.670938	1.765033	-0.24957
C	-1.405337	0.657391	-1.54493
C	-0.52796	3.404976	-1.1163
C	-1.917675	2.232719	1.041019
C	-2.332389	-0.37116	-1.30242
C	-0.78152	0.763205	-2.83209
C	-1.38163	3.762228	-2.1856
C	0.365538	4.371367	-0.60595
C	-3.115279	2.908473	0.721605
C	-1.580621	2.033584	2.394505
C	-2.713333	-1.25348	-2.38714
C	-2.943908	-0.60021	0.052412
C	-1.12126	-0.083	-3.86794
H	-0.023006	1.53496	-2.99517
C	-1.329208	5.050455	-2.74042
H	-2.084253	3.030668	-2.59826
C	0.409836	5.659735	-1.16209
H	1.035423	4.116605	0.219968
C	-3.965836	3.35718	1.741267
H	-3.379657	3.095328	-0.32416
C	-2.429429	2.492099	3.414766
H	-0.642619	1.520654	2.636596
C	-2.10314	-1.09806	-3.68599
C	-3.687799	-2.288	-2.23117
C	-2.207599	-1.18113	1.105303
C	-4.331766	-0.2576	0.251816
H	-0.635963	0.020365	-4.84582
C	-0.431779	6.002729	-2.23133
H	-1.993687	5.30715	-3.57345
H	1.116504	6.392847	-0.75816
C	-3.623259	3.153602	3.089264
H	-4.896648	3.873632	1.482134
H	-2.153796	2.333258	4.463328
C	-2.48568	-1.95984	-4.7548
H	-4.166757	-2.42666	-1.25823
C	-4.044965	-3.10762	-3.29293
P	-0.366969	-1.48016	0.963039
C	-2.860192	-1.44886	2.348432
C	-4.965431	-0.53889	1.517486
C	-5.114384	0.377328	-0.76257
H	-0.389182	7.007208	-2.66744
H	-4.285528	3.516012	3.88366
H	-2.006097	-1.8244	-5.73167
C	-3.44118	-2.94663	-4.56783
H	-4.800141	-3.88762	-3.14403
C	0.102236	-2.09568	2.648138
C	-0.20772	-3.01261	-0.05906
C	-4.196188	-1.15015	2.545259
H	-2.289125	-1.90413	3.161774
C	-6.336107	-0.19446	1.710454
H	-4.649395	0.604274	-1.72639
C	-6.443745	0.707388	-0.54231
H	-3.729148	-3.60198	-5.39721
C	0.722121	-1.20193	3.545495
C	-0.127929	-3.4274	3.05594
C	-1.293278	-3.86586	-0.34511
C	1.087891	-3.35604	-0.50445
H	-4.674562	-1.37493	3.506361
H	-6.800095	-0.41957	2.678362
C	-7.065219	0.416694	0.702648
H	-7.020809	1.194754	-1.33627
C	1.080765	-1.6207	4.836437
H	0.949136	-0.18342	3.209999
C	0.234588	-3.84589	4.34577
H	-0.583479	-4.13947	2.359008
C	-1.087232	-5.04483	-1.07997
H	-2.298967	-3.61012	0.004229
C	1.285132	-4.5363	-1.23661
H	1.926654	-2.69353	-0.25781
H	-8.117236	0.678108	0.862315
C	0.834827	-2.94293	5.239439
H	1.568429	-0.91731	5.520581
H	0.053824	-4.88309	4.65042
C	0.200447	-5.38089	-1.52781
H	-1.937589	-5.69904	-1.30371
H	2.292646	-4.79078	-1.58383
H	1.121991	-3.27397	6.24396
H	0.357901	-6.30063	-2.10302
Pd	1.109537	0.471467	0.43658

(*R*)-**10**_conf1

Symbol	X	Y	Z
C	-4.195346	-1.3196	-2.77937
C	-4.77823	-2.39833	-2.09543
C	-4.409382	-2.64611	-0.76402
C	-3.470136	-1.8217	-0.11683
C	-2.868398	-0.74352	-0.79297
C	-3.246218	-0.50361	-2.13472
H	-4.479684	-1.10478	-3.81771
H	-5.51952	-3.03432	-2.59338
H	-4.860903	-3.4821	-0.2155
H	-3.230172	-2.01195	0.935852
H	-2.800424	0.328505	-2.69652
C	-2.723915	1.704851	1.029337
C	-3.691952	0.724775	1.62408
C	-3.408491	2.842447	0.254826
C	-1.828633	2.27422	2.140884
O	-3.36366	-0.03902	2.559815
C	-5.121316	0.733734	1.151894
C	-4.542797	2.386623	-0.67144
H	-2.66159	3.422315	-0.31324
H	-3.834779	3.554008	1.000982
H	-2.458061	2.765866	2.915159
H	-1.247645	1.490763	2.651797
H	-1.144535	3.041413	1.741209
C	-5.553474	1.54921	0.07653
C	-6.056281	-0.06262	1.847754
H	-5.039528	3.26033	-1.1352
H	-4.119837	1.782472	-1.49744
C	-6.916795	1.544707	-0.27537
H	-5.682555	-0.67471	2.675033
C	-7.406916	-0.05898	1.487709
H	-7.256087	2.17823	-1.1049
C	-7.839034	0.749611	0.419607
H	-8.125911	-0.67957	2.035108
H	-8.897408	0.764806	0.133185
P	0.202129	-1.56475	-0.27864
C	1.966956	-1.46482	-0.91738
C	-0.46547	-3.0298	-1.20687
C	0.360543	-2.19446	1.45126
C	2.944772	-0.63785	-0.32585
C	2.287277	-2.15231	-2.13093
C	-0.92396	-2.82898	-2.52813
C	-0.537246	-4.32495	-0.65421
C	1.345954	-3.14146	1.81073
C	-0.573796	-1.7663	2.418062
C	2.730597	0.035517	0.999481
C	4.227093	-0.46438	-0.97073
C	3.520218	-2.02113	-2.7411
H	1.542493	-2.81112	-2.5832
C	-1.416286	-3.90136	-3.28587
H	-0.90867	-1.82415	-2.96129
C	-1.037528	-5.39607	-1.41265
H	-0.21004	-4.50169	0.374689
C	1.389554	-3.65665	3.114926
H	2.081764	-3.47197	1.069182
C	-0.525043	-2.28701	3.721992
H	-1.349086	-1.02948	2.164731
C	1.880309	1.143075	1.161769
C	3.448903	-0.48755	2.145816
C	4.51744	-1.16687	-2.19618
C	5.231522	0.411159	-0.45049
H	3.738213	-2.5756	-3.66209
C	-1.472939	-5.18998	-2.73025
H	-1.771977	-3.72407	-4.30678
H	-1.088093	-6.3956	-0.96564

C	0.452665	-3.231	4.072243
H	2.16287	-4.38446	3.384831
H	-1.261374	-1.94646	4.457957
P	0.726676	1.810305	-0.12995
C	1.688852	1.695983	2.472361
C	3.266136	0.113221	3.444028
C	4.343943	-1.59806	2.050098
C	5.782226	-0.98878	-2.83021
H	5.027758	0.964487	0.470795
C	6.449495	0.57311	-1.09553
H	-1.865855	-6.02671	-3.31892
H	0.489586	-3.63436	5.090854
C	0.845042	3.66403	0.078975
C	1.505536	1.742896	-1.8152
C	2.362489	1.205203	3.572203
H	0.990271	2.526367	2.605625
C	3.979312	-0.39653	4.56802
H	4.493584	-2.07929	1.080134
C	5.02778	-2.07148	3.161598
H	5.983701	-1.53804	-3.758
C	6.734272	-0.13587	-2.29379
H	7.199219	1.253086	-0.67535
C	1.86658	4.33279	0.787031
C	-0.103163	4.436842	-0.6275
C	2.763739	2.318025	-2.09161
C	0.728331	1.24613	-2.88105
H	2.201884	1.652283	4.560599
H	3.822441	0.077535	5.544483
C	4.849599	-1.46717	4.433935
H	5.710975	-2.92165	3.054444
H	7.701933	-0.00427	-2.79072
C	1.916925	5.73684	0.81015
H	2.628789	3.761532	1.326333
C	-0.048872	5.838008	-0.60776
H	-0.882486	3.933247	-1.21046
C	3.242724	2.372606	-3.40799
H	3.369079	2.730022	-1.27745
C	1.204534	1.311395	-4.20099
H	-0.256871	0.813961	-2.6605
H	5.394483	-1.84979	5.304115
C	0.958618	6.493701	0.118839
H	2.715137	6.238104	1.369737
H	-0.797366	6.417318	-1.1599
C	2.463022	1.872723	-4.46562
H	4.226593	2.810841	-3.60858
H	0.588747	0.925581	-5.02146
H	0.999339	7.588408	0.141179
H	2.836105	1.926409	-5.49474
Pd	-1.30229	0.416048	-0.11255

(*R*)-**10**_conf2

Symbol	X	Y	Z
C	3.934351	3.340039	-2.42374
C	4.502021	3.98861	-1.31514
C	4.232774	3.502326	-0.02652
C	3.402207	2.37993	0.153669
C	2.830995	1.707456	-0.94908
C	3.115501	2.209599	-2.24011
H	4.133889	3.7059	-3.43915
H	5.146876	4.864132	-1.4548
H	4.664289	4.000329	0.850742
H	3.191555	2.043889	1.17656
H	2.702287	1.712262	-3.12777
Pd	1.450294	0.189388	-0.90902
C	3.05297	-1.18908	-1.64999
C	2.702358	-2.39328	-0.83026
C	4.52217	-0.77589	-1.4393
C	2.765577	-1.38595	-3.14058
O	1.831663	-3.22191	-1.18369
C	3.466234	-2.65587	0.445565
C	4.956426	-0.64991	0.025403
H	4.735958	0.16655	-1.97151
H	5.151729	-1.5564	-1.92658
H	3.553043	-2.02441	-3.59926
H	1.804772	-1.89452	-3.29725
H	2.772461	-0.42647	-3.68836
C	4.55382	-1.84443	0.855349
C	3.113768	-3.78867	1.213691
H	6.051562	-0.49999	0.088972
H	4.499371	0.26261	0.452588
C	5.239796	-2.17341	2.042649
H	2.299922	-4.42027	0.843091
C	3.799662	-4.10234	2.390657
H	6.088924	-1.55246	2.356041
C	4.86703	-3.28567	2.810536
H	3.516463	-4.98422	2.977006
H	5.420469	-3.52857	3.725525
P	-0.011477	1.607998	0.530602
C	-1.889287	1.640168	0.452971
C	0.387863	3.423392	0.474812
C	0.355593	1.114863	2.27507
C	-2.667583	0.484381	0.66442
C	-2.540149	2.856379	0.074391
C	0.492662	4.03261	-0.79558
C	0.579762	4.214103	1.625451
C	-0.41784	1.564513	3.368135
C	1.469099	0.282816	2.51765
C	-2.048255	-0.79769	1.133047
C	-4.0975	0.534018	0.4657
C	-3.911801	2.926949	-0.08375
H	-1.943049	3.757801	-0.08141
C	0.766352	5.402711	-0.91065
H	0.372532	3.42627	-1.6989
C	0.860181	5.585681	1.507911
H	0.523161	3.759198	2.618929
C	-0.066766	1.20496	4.678307
H	-1.304177	2.184277	3.192534
C	1.816565	-0.07758	3.829443
H	2.050833	-0.09405	1.665694
C	-1.210353	-1.56885	0.311482
C	-2.307179	-1.22814	2.493753
C	-4.728285	1.776463	0.093686
C	-4.926129	-0.62432	0.597328
H	-4.383701	3.878494	-0.3576
C	0.951065	6.184084	0.242284
H	0.850501	5.856884	-1.9041
H	1.012172	6.185124	2.4129
C	1.052176	0.387366	4.911122
H	-0.677315	1.554634	5.518228
H	2.679417	-0.73148	3.996436
P	-0.596619	-1.14402	-1.3895
C	-0.551027	-2.72331	0.85407
C	-1.690288	-2.4328	2.992408
C	-3.153906	-0.49884	3.384849
C	-6.140868	1.820192	-0.09837
H	-4.462887	-1.57957	0.860617
C	-6.296573	-0.55376	0.391391
H	1.17464	7.253169	0.152557
H	1.3194	0.104519	5.935667
C	-0.935453	-2.68905	-2.38897
C	-1.763168	-0.04713	-2.33544
C	-0.79646	-3.14697	2.144701
H	0.178242	-3.25761	0.232022
C	-1.962659	-2.86871	4.321892
H	-3.617821	0.429202	3.040578
C	-3.404109	-0.9502	4.673887
H	-6.601715	2.776621	-0.37384
C	-6.914652	0.679465	0.048059
H	-6.907849	-1.45726	0.495278
C	-1.562495	-3.8468	-1.88785
C	-0.622646	-2.63465	-3.76628
C	-3.119617	-0.37232	-2.5497
C	-1.204802	1.046664	-3.02933
H	-0.285312	-4.03433	2.536959
H	-1.483249	-3.78907	4.676952
C	-2.809622	-2.14812	5.150003
H	-4.065726	-0.3732	5.33006
H	-7.998734	0.723495	-0.10517
C	-1.838516	-4.93342	-2.73545
H	-1.842387	-3.91174	-0.83256
C	-0.895966	-3.72026	-4.60904
H	-0.176083	-1.72871	-4.19051
C	-3.905666	0.401957	-3.41476
H	-3.561011	-1.23885	-2.04659
C	-1.989567	1.813408	-3.90742
H	-0.14129	1.279976	-2.88227
H	-3.015314	-2.49535	6.168746
C	-1.500348	-4.87952	-4.09497
H	-2.324163	-5.82555	-2.32318

H	-0.638053	-3.65707	-5.67231
C	-3.342651	1.494941	-4.09699
H	-4.960579	0.145504	-3.56253
H	-1.539734	2.655395	-4.44616
H	-1.712827	-5.73014	-4.75229
H	-3.957647	2.090053	-4.78177

(*R*)-**10**_conf3

Symbol	X	Y	Z
C	-0.817781	-3.73814	3.045228
C	-1.837036	-3.55931	3.994525
C	-2.776626	-2.53284	3.805943
C	-2.696745	-1.68894	2.681448
C	-1.684086	-1.86834	1.713452
C	-0.747378	-2.90652	1.910017
H	-0.072039	-4.5321	3.180086
H	-1.898261	-4.21265	4.872942
H	-3.576848	-2.37721	4.540346
H	-3.425933	-0.8773	2.575165
H	0.051573	-3.06519	1.175041
Pd	-1.63383	-0.78336	0.008071
C	-2.56702	-2.4497	-1.1554
C	-3.055646	-1.20915	-1.735
C	-3.592079	-3.41125	-0.57447
C	-1.35152	-3.11593	-1.7785
O	-2.206338	-0.34355	-2.19879
C	-4.519233	-0.92365	-1.76765
C	-4.852287	-2.71843	-0.02626
H	-3.12453	-4.01554	0.224337
H	-3.880725	-4.1248	-1.3792
H	-1.688013	-3.86825	-2.52558
H	-0.71075	-2.38249	-2.29154
H	-0.748518	-3.65458	-1.02642
C	-5.41738	-1.70102	-0.9918
C	-5.009794	0.081698	-2.62636
H	-5.620561	-3.46826	0.238469
H	-4.576202	-2.20392	0.915926
C	-6.796325	-1.45143	-1.10394
H	-4.28929	0.65544	-3.21868
C	-6.386966	0.315492	-2.72878
H	-7.497481	-2.04675	-0.50558
C	-7.282236	-0.45396	-1.96525
H	-6.764425	1.091091	-3.40488
H	-8.361803	-0.2802	-2.04317
P	-0.746828	1.237817	0.949412
C	1.072879	1.681689	1.116489
C	-1.381352	1.422818	2.684705
C	-1.422644	2.760637	0.124507
C	1.993862	1.493762	0.067948
C	1.506014	2.3251	2.319954
C	-0.811882	0.653333	3.724996
C	-2.50272	2.228427	2.976686
C	-1.111675	4.048317	0.619901
C	-2.272052	2.634944	-0.99187
C	1.629078	0.900907	-1.26648
C	3.346344	1.986275	0.211802
C	2.806068	2.765147	2.483265
H	0.793442	2.470566	3.135607
C	-1.317885	0.728548	5.030556
H	0.035417	-0.00736	3.514152
C	-3.021043	2.285151	4.281608
H	-2.973565	2.819654	2.185448
C	-1.655991	5.189988	0.014977
H	-0.444	4.159312	1.481794
C	-2.813194	3.784353	-1.59421
H	-2.482795	1.642049	-1.41023
C	1.812246	-0.46027	-1.57027
C	1.212412	1.82128	-2.30485
C	3.7608	2.614831	1.441659
C	4.305347	1.888956	-0.84377
H	3.108501	3.250953	3.419071
C	-2.424468	1.545661	5.314284
H	-0.855673	0.130332	5.82349
H	-3.891464	2.918399	4.48815
C	-2.512083	5.059079	-1.0931
H	-1.412051	6.183111	0.409841
H	-3.470175	3.676035	-2.46435
P	2.183411	-1.85354	-0.39
C	1.483944	-0.92264	-2.8899
C	0.911428	1.32721	-3.62603
C	1.12055	3.230053	-2.08506
C	5.09905	3.088333	1.577
H	4.002902	1.435679	-1.79178
C	5.597373	2.370487	-0.68826
H	-2.824552	1.597919	6.333195
H	-2.938498	5.952055	-1.56478
C	3.364364	-2.90029	-1.37874
C	3.313665	-1.29775	0.982058
C	1.047551	-0.06586	-3.87916
H	1.594787	-1.98714	-3.12075
C	0.51244	2.236934	-4.64747
H	1.350882	3.631125	-1.09454
C	0.740041	4.093952	-3.10245
H	5.394915	3.557085	2.523406
C	6.004105	2.969928	0.534096
H	6.309944	2.290441	-1.51702
C	4.274644	-2.41399	-2.34476
C	3.358051	-4.28443	-1.09714
C	4.699292	-1.0686	0.834924
C	2.754722	-1.26537	2.278918
H	0.810347	-0.45222	-4.87776
H	0.280303	1.836228	-5.6418
C	0.426904	3.59761	-4.39538
H	0.676009	5.168903	-2.90168
H	7.028046	3.343978	0.645827
C	5.157336	-3.28669	-3.00053
H	4.281752	-1.34763	-2.59639
C	4.247169	-5.15633	-1.74565
H	2.645834	-4.67947	-0.36258
C	5.497792	-0.78734	1.953364
H	5.161208	-1.12537	-0.15563
C	3.554314	-0.98085	3.397818
H	1.689259	-1.49005	2.409858
H	0.122785	4.289219	-5.18914
C	5.149131	-4.65927	-2.69984
H	5.85531	-2.89227	-3.74835
H	4.227368	-6.22698	-1.51195
C	4.927439	-0.7399	3.236731
H	6.571075	-0.60905	1.822058
H	3.104796	-0.96323	4.397327
H	5.838812	-5.33898	-3.21302
H	5.555669	-0.52814	4.109719

(*R*)-**10**_conf4

Symbol	X	Y	Z
C	4.587714	2.766432	-0.55203
C	4.916075	3.38705	0.663174
C	4.2692	2.978205	1.842279
C	3.301743	1.957495	1.803957
C	2.955175	1.34021	0.583358
C	3.610407	1.753812	-0.5934
H	5.092827	3.066292	-1.47855
H	5.678975	4.173842	0.695331
H	4.525378	3.445324	2.801532
H	2.83163	1.634489	2.74057
H	3.375865	1.27941	-1.55305
Pd	1.424736	-0.00139	0.570333
C	2.989267	-1.52786	1.091584
C	4.38816	-1.05548	0.784367
C	2.660524	-2.75945	0.226467
C	2.865839	-1.7842	2.594871
O	5.137815	-0.58841	1.659032
C	4.932343	-1.25835	-0.61031
C	2.820933	-2.46666	-1.27415
H	1.640202	-3.13002	0.436452
H	3.348901	-3.59298	0.500793
H	3.637555	-2.51258	2.924727
H	3.046308	-0.86304	3.172595
H	1.881025	-2.19028	2.862765
C	4.207105	-1.96256	-1.6002
C	6.226048	-0.77665	-0.90091
H	2.581161	-3.35705	-1.88707
H	2.087963	-1.6716	-1.54453
C	4.793123	-2.16497	-2.86548
H	6.755623	-0.24702	-0.10224
C	6.794935	-0.97308	-2.16323

H	4.238254	-2.72433	-3.63032
C	6.074906	-1.67274	-3.15058
H	7.7983	-0.58943	-2.38247
H	6.517765	-1.84334	-4.13925
P	-0.741754	-1.49304	0.934245
C	-2.423638	-0.64134	1.008777
C	-0.601412	-2.22934	2.634782
C	-1.241564	-2.95827	-0.10532
C	-2.924685	0.036066	-0.11772
C	-3.225656	-0.71871	2.18845
C	-0.512793	-1.35522	3.744502
C	-0.437194	-3.61343	2.854028
C	-2.329524	-3.77942	0.27142
C	-0.571077	-3.22883	-1.31298
C	-2.158109	0.072476	-1.4067
C	-4.214317	0.682297	-0.05614
C	-4.473804	-0.12593	2.255658
H	-2.851524	-1.26372	3.058567
C	-0.295066	-1.85428	5.036187
H	-0.618373	-0.27512	3.595534
C	-0.204958	-4.10957	4.148286
H	-0.479278	-4.311	2.012291
C	-2.70736	-4.8693	-0.52566
H	-2.88058	-3.567	1.1943
C	-0.954818	-4.31755	-2.11442
H	0.245898	-2.57729	-1.63411
C	-0.991882	0.83986	-1.54788
C	-2.637782	-0.71664	-2.52315
C	-4.999331	0.598396	1.150935
C	-4.745647	1.4342	-1.15021
H	-5.071456	-0.20745	3.171855
C	-0.13693	-3.23533	5.242651
H	-0.235615	-1.16034	5.882183
H	-0.073069	-5.18744	4.29557
C	-2.016331	-5.14402	-1.71872
H	-3.546595	-5.50296	-0.21645
H	-0.423428	-4.51325	-3.05217
P	-0.097867	1.73245	-0.19343
C	-0.250168	0.778326	-2.77619
C	-1.910895	-0.70615	-3.76987
C	-3.814418	-1.52273	-2.44359
C	-6.271217	1.24101	1.210691
H	-4.15999	1.518027	-2.07034
C	-5.982404	2.056796	-1.05897
H	0.046188	-3.6246	6.250226
H	-2.313139	-5.99548	-2.34145
C	0.390187	3.332488	-1.01414
C	-1.229903	2.461338	1.082622
C	-0.703103	0.045002	-3.85447
H	0.687031	1.337662	-2.86211
C	-2.399804	-1.45711	-4.87801
H	-4.365404	-1.56838	-1.5007
C	-4.263372	-2.24763	-3.53898
H	-6.854318	1.161697	2.136184
C	-6.75761	1.957388	0.128463
H	-6.365746	2.629446	-1.91096
C	-0.283511	3.844354	-2.14656
C	1.376807	4.128004	-0.39173
C	-2.327174	3.291255	0.771067
C	-0.83792	2.317405	2.429631
H	-0.131025	0.030352	-4.79007
H	-1.836269	-1.42852	-5.81857
C	-3.558951	-2.21062	-4.77126
H	-5.170151	-2.85621	-3.44974
H	-7.735014	2.449277	0.185488
C	0.037516	5.113051	-2.65475
H	-1.061501	3.253393	-2.64068
C	1.688838	5.398332	-0.90071
H	1.905767	3.758004	0.4913
C	-3.036762	3.936963	1.793439
H	-2.623093	3.442198	-0.27202
C	-1.541349	2.976328	3.45182
H	0.04042	1.701964	2.661288
H	-3.927366	-2.78283	-5.62999
C	1.026509	5.893138	-2.03464
H	-0.492596	5.491333	-3.53653
H	2.465043	5.994382	-0.40871
C	-2.645249	3.782378	3.135001
H	-3.894468	4.569948	1.540234
H	-1.220338	2.864479	4.493779
H	1.277623	6.882675	-2.43335
H	-3.195393	4.298998	3.929667

(*R*)-**10**_conf5

Symbol	X	Y	Z
C	3.934168	3.341271	-2.4231
C	4.501791	3.989665	-1.31437
C	4.232592	3.503091	-0.02585
C	3.402129	2.38059	0.15412
C	2.83101	1.708254	-0.94877
C	3.115434	2.210708	-2.23969
H	4.133655	3.707349	-3.43844
H	5.146566	4.865275	-1.45384
H	4.664046	4.000976	0.851511
H	3.19149	2.044366	1.176951
H	2.702251	1.713522	-3.12744
Pd	1.450585	0.189974	-0.90903
C	3.053378	-1.18819	-1.64987
C	2.702467	-2.39297	-0.83099
C	4.52243	-0.77499	-1.43799
C	2.766994	-1.38456	-3.14074
O	1.831989	-3.22141	-1.18533
C	3.465901	-2.65638	0.444933
C	4.955509	-0.64954	0.02711
H	4.736512	0.167699	-1.96965
H	5.15245	-1.55523	-1.92508
H	3.554743	-2.02291	-3.59907
H	1.806271	-1.89303	-3.29826
H	2.774243	-0.42491	-3.68822
C	4.552904	-1.8448	0.855977
C	3.113538	-3.79	1.211897
H	6.050514	-0.49907	0.091626
H	4.497577	0.262473	0.454436
C	5.238317	-2.17442	2.043425
H	2.300163	-4.42163	0.840318
C	3.798936	-4.10436	2.388965
H	6.086962	-1.55332	2.357806
C	4.865643	-3.28751	2.810163
H	3.515852	-4.9869	2.974383
H	5.41866	-3.53089	3.725281
P	-0.011619	1.607871	0.531141
C	-1.889406	1.639904	0.453546
C	0.387656	3.423304	0.476061
C	0.355475	1.114102	2.275407
C	-2.667586	0.483954	0.664561
C	-2.540355	2.856223	0.07547
C	0.492689	4.032977	-0.79409
C	0.57918	4.21364	1.627024
C	-0.418026	1.563195	3.36865
C	1.46909	0.282104	2.517633
C	-2.048149	-0.79829	1.132606
C	-4.097515	0.533589	0.465919
C	-3.912024	2.926776	-0.08253
H	-1.943293	3.757719	-0.08006
C	0.766227	5.403155	-0.90862
H	0.372952	3.426914	-1.69765
C	0.859465	5.585294	1.510037
H	0.522394	3.758384	2.620337
C	-0.066911	1.203132	4.67867
H	-1.304439	2.182916	3.193292
C	1.816589	-0.07881	3.829279
H	2.050859	-0.09432	1.665504
C	-1.21023	-1.56906	0.310672
C	-2.307011	-1.22936	2.493129
C	-4.728406	1.776157	0.094482
C	-4.926075	-0.62486	0.597059
H	-4.384007	3.878392	-0.35599
C	0.950559	6.184153	0.244637
H	0.850566	5.857681	-1.90189
H	1.011154	6.184431	2.415279
C	1.052127	0.385572	4.911145
H	-0.677507	1.552371	5.518739
H	2.679514	-0.73269	3.996006
P	-0.596368	-1.14343	-1.39007
C	-0.550888	-2.72377	0.852724
C	-1.690036	-2.4342	2.991242
C	-3.153748	-0.5005	3.384575
C	-6.141006	1.819883	-0.09746
H	-4.462762	-1.5802	0.859895

C	-6.296534	-0.55429	0.391234
H	1.174009	7.253299	0.155328
H	1.31937	0.102318	5.935573
C	-0.934957	-2.68802	-2.39026
C	-1.763074	-0.04629	-2.33553
C	-0.796251	-3.148	2.143181
H	0.178369	-3.2578	0.230426
C	-1.962289	-2.87067	4.320569
H	-3.617745	0.427654	3.040714
C	-3.403851	-0.95241	4.673438
H	-6.60192	2.77641	-0.37248
C	-6.914715	0.679047	0.048496
H	-6.907747	-1.45788	0.494754
C	-1.562418	-3.84586	-1.88992
C	-0.621561	-2.63308	-3.76742
C	-3.119476	-0.37167	-2.54985
C	-1.205004	1.047975	-3.02888
H	-0.285055	-4.03551	2.535037
H	-1.482787	-3.79113	4.675225
C	-2.809248	-2.15048	5.149029
H	-4.065488	-0.37574	5.32988
H	-7.998809	0.723076	-0.10465
C	-1.838315	-4.93205	-2.73812
H	-1.842715	-3.91124	-0.83476
C	-0.894756	-3.71824	-4.61078
H	-0.174589	-1.72704	-4.19104
C	-3.90577	0.402898	-3.41443
H	-3.560633	-1.23855	-2.04715
C	-1.990014	1.815023	-3.90649
H	-0.141531	1.281444	-2.88182
H	-3.014847	-2.49814	6.167645
C	-1.4996	-4.87761	-4.09748
H	-2.324301	-5.82426	-2.32642
H	-0.636389	-3.65464	-5.67392
C	-3.343045	1.496371	-4.09612
H	-4.960642	0.146296	-3.56222
H	-1.540408	2.657408	-4.44479
H	-1.711995	-5.72789	-4.75526
H	-3.958221	2.091731	-4.78051

(*R*)-**10**_conf6

Symbol	X	Y	Z
C	3.656333	4.1709	-1.27997
C	4.053209	4.67633	-0.03104
C	3.756159	3.942442	1.127004
C	3.080445	2.709056	1.036067
C	2.686949	2.176916	-0.21125
C	2.977902	2.941728	-1.36366
H	3.872393	4.7337	-2.19657
H	4.584661	5.632875	0.038006
H	4.053696	4.323302	2.112493
H	2.872282	2.164254	1.963279
H	2.67186	2.579316	-2.35257
Pd	1.636251	0.416193	-0.48794
C	3.671242	-0.48777	-0.76101
C	3.420568	-1.96461	-0.81288
C	4.608143	-0.17325	0.421707
C	4.235124	0.000113	-2.09836
O	3.346467	-2.58927	-1.88987
C	3.368843	-2.73568	0.484955
C	4.037626	-0.65625	1.761664
H	4.823922	0.905729	0.470726
H	5.581802	-0.68566	0.243734
H	5.168909	-0.55034	-2.34008
H	3.535231	-0.1912	-2.92878
H	4.471466	1.074998	-2.06443
C	3.683778	-2.12557	1.725348
C	3.057565	-4.11245	0.438111
H	4.739278	-0.45366	2.593674
H	3.116059	-0.07124	1.96881
C	3.662789	-2.91087	2.894484
H	2.845265	-4.55352	-0.54094
C	3.026806	-4.8781	1.609596
H	3.922901	-2.44633	3.854495
C	3.331608	-4.27364	2.843862
H	2.776104	-5.94413	1.564394
H	3.327809	-4.86903	3.764867
P	-0.357819	1.826609	0.032474
C	-2.146411	1.374207	-0.35656
C	-0.182649	3.497663	-0.75402
C	-0.491361	2.175641	1.84656
C	-2.698235	0.169604	0.118586
C	-2.958839	2.245203	-1.14664
C	-0.281858	3.599516	-2.16171
C	0.155981	4.649138	-0.01563
C	-1.434752	3.097581	2.352223
C	0.33052	1.470068	2.746067
C	-1.947628	-0.71713	1.066427
C	-4.041365	-0.20559	-0.25694
C	-4.260972	1.918603	-1.48085
H	-2.551052	3.200592	-1.4842
C	-0.091783	4.830777	-2.80539
H	-0.521371	2.71229	-2.75874
C	0.361917	5.877151	-0.66553
H	0.282118	4.588199	1.068607
C	-1.525738	3.330479	3.732369
H	-2.097673	3.63503	1.664779
C	0.231728	1.697743	4.128117
H	1.032782	0.727913	2.348498
C	-0.838107	-1.47613	0.668785
C	-2.395763	-0.77323	2.445444
C	-4.833416	0.68376	-1.07045
C	-4.621111	-1.45454	0.129685
H	-4.864685	2.614413	-2.07637
C	0.229459	5.976064	-2.05816
H	-0.186876	4.891918	-3.89564
H	0.632929	6.758757	-0.07399
C	-0.689601	2.634092	4.621945
H	-2.255941	4.052926	4.114524
H	0.873519	1.138958	4.818084
P	0.04985	-1.42746	-0.96266
C	-0.134283	-2.26608	1.639817
C	-1.711833	-1.62659	3.386355
C	-3.500837	-0.00517	2.925029
C	-6.158794	0.307853	-1.44077
H	-4.030872	-2.14838	0.735082
C	-5.908463	-1.79853	-0.25717
H	0.386617	6.936725	-2.5614
H	-0.765498	2.814458	5.700167
C	0.135745	-3.23486	-1.42856
C	-1.017115	-0.91645	-2.39474
C	-0.568553	-2.35385	2.947152
H	0.760807	-2.81876	1.335255
C	-2.177075	-1.71083	4.730861
H	-4.015166	0.673672	2.239939
C	-3.928847	-0.10494	4.242014
H	-6.747092	1.000992	-2.05438
C	-6.690317	-0.90903	-1.04359
H	-6.326638	-2.7644	0.047636
C	-0.572569	-4.25223	-0.75367
C	0.846373	-3.56268	-2.6047
C	-2.242424	-1.53907	-2.71167
C	-0.461826	0.008569	-3.30363
H	-0.023281	-2.98138	3.661975
H	-1.646968	-2.37297	5.426329
C	-3.2708	-0.97106	5.154049
H	-4.781571	0.494411	4.580315
H	-7.708115	-1.18954	-1.33678
C	-0.55724	-5.57117	-1.23642
H	-1.143999	-4.02166	0.150424
C	0.858158	-4.88145	-3.08283
H	1.401242	-2.7888	-3.14065
C	-2.917168	-1.20972	-3.89575
H	-2.666377	-2.28927	-2.03659
C	-1.129645	0.322626	-4.50013
H	0.50802	0.465661	-3.06613
H	-3.621956	-1.0458	6.18934
C	0.160386	-5.89057	-2.39967
H	-1.114454	-6.34812	-0.70011
H	1.423992	-5.11877	-3.9905
C	-2.36242	-0.27997	-4.793
H	-3.875931	-1.68871	-4.12318
H	-0.680014	1.031543	-5.20506
H	0.172815	-6.92054	-2.77435
H	-2.886023	-0.03712	-5.72467

(*R*)-**10**_conf7

Symbol	X	Y	Z
C	4.347021	-2.57181	1.166101
C	4.447658	-3.62793	0.246812
C	3.6808	-3.59009	-0.92822
C	2.817893	-2.50781	-1.18038
C	2.693786	-1.45036	-0.25701
C	3.479939	-1.4929	0.913954
H	4.951944	-2.57605	2.081143
H	5.126503	-4.46738	0.43922
H	3.760343	-4.3993	-1.66517
H	2.260994	-2.4946	-2.12276
H	3.436182	-0.67264	1.640432
Pd	1.345518	0.09496	-0.4442
C	2.812106	1.059343	-1.80604
C	4.175693	0.419864	-1.76788
C	2.945548	2.542505	-1.42569
C	2.16809	0.853053	-3.18006
O	4.544341	-0.41488	-2.61134
C	5.15278	0.900294	-0.72142
C	3.546967	2.729577	-0.0262
H	1.972207	3.054551	-1.50137
H	3.613881	3.041829	-2.16595
H	2.785986	1.328083	-3.97291
H	2.106409	-0.2175	-3.43484
H	1.158881	1.296151	-3.23197
C	4.873587	2.013943	0.107852
C	6.396799	0.243396	-0.61745
H	3.670204	3.803194	0.21726
H	2.830384	2.307938	0.714347
C	5.847249	2.436493	1.034324
H	6.576207	-0.60338	-1.28781
C	7.351813	0.665451	0.312537
H	5.64098	3.309109	1.668046
C	7.074978	1.767919	1.143326
H	8.312704	0.143787	0.392497
H	7.822325	2.11368	1.867596
P	-0.300001	-1.51831	0.520202
C	-1.943157	-1.06878	1.327433
C	0.486292	-2.61851	1.788826
C	-0.867662	-2.66068	-0.82079
C	-2.895149	-0.28903	0.642199
C	-2.223734	-1.4822	2.666911
C	0.880632	-2.04737	3.021824
C	0.793087	-3.97102	1.541411
C	-1.711206	-3.76093	-0.54911
C	-0.477914	-2.40402	-2.1503
C	-2.727589	0.079878	-0.80243
C	-4.102742	0.132568	1.31471
C	-3.390061	-1.11534	3.312991
H	-1.506471	-2.11537	3.193528
C	1.521249	-2.82304	3.998092
H	0.673423	-0.99041	3.224698
C	1.451901	-4.74047	2.514797
H	0.536421	-4.42433	0.580393
C	-2.128539	-4.60507	-1.58888
H	-2.040666	-3.95753	0.47744
C	-0.90554	-3.24439	-3.19055
H	0.154859	-1.53227	-2.35906
C	-1.791738	1.030982	-1.23802
C	-3.583464	-0.57681	-1.77302
C	-4.350691	-0.28624	2.672401
C	-5.068228	0.984952	0.691946
H	-3.581839	-1.46085	4.336289
C	1.807984	-4.1752	3.747558
H	1.806993	-2.36588	4.952389
H	1.689791	-5.78836	2.300409
C	-1.72361	-4.34983	-2.91034
H	-2.776484	-5.46084	-1.36747
H	-0.596615	-3.03372	-4.22033
P	-0.438369	1.811657	-0.23369
C	-1.674381	1.310915	-2.64128
C	-3.482976	-0.2328	-3.17015
C	-4.540212	-1.57081	-1.40155
C	-5.540062	0.137118	3.335993
H	-4.894185	1.324149	-0.33328
C	-6.210824	1.390254	1.367247
H	2.317982	-4.78014	4.505671
H	-2.053876	-5.0088	-3.72127
C	-0.575306	3.627979	-0.68616
C	-0.890275	2.030087	1.554774
C	-2.501712	0.717178	-3.57271
H	-0.916753	2.024344	-2.98078
C	-4.349043	-0.85286	-4.11686
H	-4.617149	-1.87061	-0.3534
C	-5.369153	-2.16033	-2.34633
H	-5.711297	-0.19796	4.366234
C	-6.456039	0.959869	2.699556
H	-6.931739	2.046941	0.867386
C	-1.621828	4.192264	-1.44603
C	0.394447	4.494975	-0.13136
C	-2.068288	2.679682	1.980006
C	0.091489	1.693684	2.510198
H	-2.402797	0.966099	-4.63612
H	-4.257624	-0.57005	-5.17258
C	-5.282566	-1.79602	-3.71555
H	-6.095443	-2.91771	-2.03078
H	-7.365159	1.28176	3.219574
C	-1.678331	5.57925	-1.66777
H	-2.404588	3.55472	-1.86794
C	0.334408	5.878345	-0.35071
H	1.195539	4.087209	0.494708
C	-2.271022	2.957066	3.339457
H	-2.827666	2.975223	1.248599
C	-0.105827	1.988173	3.870276
H	1.015466	1.206058	2.171554
H	-5.945651	-2.2656	-4.45063
C	-0.699716	6.426278	-1.12752
H	-2.499532	5.995231	-2.26284
H	1.099052	6.529	0.088306
C	-1.289784	2.615652	4.286791
H	-3.196188	3.449262	3.659006
H	0.669908	1.731716	4.600973
H	-0.745328	7.506704	-1.3038
H	-1.446475	2.847071	5.346483

(*S*)-**10**_conf1

Symbol	X	Y	Z
P	-0.31292	-1.57577	0.655893
C	-2.077743	-1.1005	1.078854
C	0.321411	-2.29121	2.254154
C	-0.598042	-3.11903	-0.35134
C	-2.889116	-0.44137	0.134083
C	-2.602882	-1.37693	2.377845
C	1.236461	-1.53062	3.012808
C	-0.060593	-3.56687	2.725781
C	-1.790016	-3.87039	-0.23361
C	0.426278	-3.59732	-1.19556
C	-2.368847	-0.14874	-1.24496
C	-4.234211	-0.05781	0.481924
C	-3.896852	-1.02764	2.722996
H	-1.970129	-1.8809	3.113578
C	1.739469	-2.02108	4.22909
H	1.600315	-0.56913	2.624918
C	0.441521	-4.0547	3.942393
H	-0.745913	-4.18497	2.135857
C	-1.949274	-5.07342	-0.93917
H	-2.598618	-3.51835	0.415985
C	0.259118	-4.79865	-1.90309
H	1.354876	-3.03325	-1.30447
C	-1.413364	0.856782	-1.45882
C	-2.826929	-0.94794	-2.36144
C	-4.745675	-0.36196	1.796374
C	-5.089673	0.636162	-0.42911
H	-4.280498	-1.25788	3.72453
C	1.338224	-3.28118	4.699269
H	2.464025	-1.42045	4.789201
H	0.137708	-5.0474	4.294387
C	-0.924776	-5.54124	-1.77746
H	-2.87925	-5.64399	-0.83263
H	1.066887	-5.15023	-2.5547
P	-0.629648	1.888402	-0.12154
C	-0.815828	1.00101	-2.75407
C	-2.255563	-0.7401	-3.67087
C	-3.842104	-1.94411	-2.22699
C	-6.078411	0.016415	2.13562
H	-4.712041	0.882968	-1.42594
C	-6.380321	0.995037	-0.06781
H	1.735587	-3.66925	5.644252
H	-1.050642	-6.47916	-2.33049

C	-0.601569	3.587467	-0.91656
C	-1.847047	2.288228	1.230286
C	-1.225592	0.231101	-3.82499
H	-0.021744	1.741227	-2.89555
C	-2.722041	-1.51237	-4.77434
H	-4.283803	-2.12319	-1.24321
C	-4.281164	-2.67493	-3.3221
H	-6.452296	-0.22586	3.137839
C	-6.883902	0.680277	1.223652
H	-7.017893	1.524559	-0.78478
C	-1.40663	3.938019	-2.02468
C	0.168308	4.604197	-0.307
C	-3.001648	3.071036	1.009646
C	-1.506836	1.92661	2.550124
H	-0.761222	0.363742	-4.80966
H	-2.273477	-1.33957	-5.76012
C	-3.720089	-2.46017	-4.60869
H	-5.066457	-3.42775	-3.191
H	-7.906349	0.965618	1.495382
C	-1.418176	5.252262	-2.52041
H	-2.031095	3.182393	-2.5107
C	0.153923	5.917462	-0.80158
H	0.779612	4.377348	0.570677
C	-3.804175	3.469768	2.08735
H	-3.266166	3.381745	-0.0064
C	-2.307723	2.334599	3.629629
H	-0.605111	1.329943	2.729585
H	-4.073511	-3.04569	-5.46466
C	-0.634845	6.246482	-1.91477
H	-2.0494	5.496374	-3.38268
H	0.763851	6.684259	-0.31093
C	-3.455872	3.107445	3.400163
H	-4.700572	4.072107	1.90228
H	-2.027121	2.050968	4.650194
H	-0.643387	7.270811	-2.30368
H	-4.077315	3.433021	4.242177
C	2.686189	-1.0328	-0.45358
C	3.415009	-1.93934	0.338328
C	2.789138	-1.1408	-1.86132
C	4.207735	-2.94046	-0.25437
H	3.39425	-1.85344	1.429923
C	3.598851	-2.12877	-2.45466
H	2.215576	-0.46935	-2.51178
C	4.306138	-3.03888	-1.65154
H	4.759297	-3.64007	0.386057
H	3.666972	-2.18743	-3.54845
H	4.935066	-3.8106	-2.11082
C	3.209243	2.763382	-0.33057
C	2.866643	1.758303	0.780494
C	4.035384	2.153314	-1.47049
H	2.291977	3.217408	-0.74139
H	3.794197	3.600151	0.119851
C	4.047409	0.933111	1.218094
C	2.259555	2.435589	2.020187
C	5.28614	1.489048	-0.94699
H	4.295173	2.924239	-2.2208
H	3.417369	1.392953	-1.98891
O	4.07316	0.362484	2.329627
C	5.261272	0.875669	0.330381
H	2.950014	3.225475	2.391891
H	2.13331	1.714505	2.843082
H	1.286818	2.910201	1.820671
C	6.476594	1.450797	-1.69774
C	6.418545	0.236307	0.824069
H	6.501598	1.931531	-2.68417
C	7.622729	0.814725	-1.19971
H	6.357507	-0.224	1.815576
C	7.593657	0.202333	0.067365
H	8.542217	0.800916	-1.79706
H	8.488341	-0.29547	0.459101
Pd	1.284135	0.333996	0.200482

(*S*)-**10**_conf2

Symbol	X	Y	Z
P	-0.301144	-1.58827	0.65325
C	-2.085728	-1.14634	1.040059
C	0.324866	-2.28244	2.262923
C	-0.515111	-3.15571	-0.33632
C	-2.887472	-0.46977	0.098195
C	-2.634613	-1.46386	2.320034
C	1.239844	-1.52173	3.020923
C	-0.062097	-3.55637	2.73652
C	-1.757943	-3.82089	-0.42273
C	0.613366	-3.74153	-0.95201
C	-2.354483	-0.14677	-1.269
C	-4.238931	-0.09823	0.436148
C	-3.935979	-1.13141	2.652632
H	-2.014007	-1.98654	3.052868
C	1.737977	-2.01071	4.240094
H	1.60691	-0.56245	2.630252
C	0.434478	-4.04198	3.955997
H	-0.747836	-4.17232	2.144437
C	-1.873018	-5.03573	-1.1175
H	-2.641595	-3.39331	0.0607
C	0.491646	-4.95476	-1.64661
H	1.590201	-3.25982	-0.88325
C	-1.401201	0.865958	-1.4513
C	-2.797977	-0.92308	-2.40801
C	-4.77121	-0.44024	1.732695
C	-5.080786	0.620861	-0.46808
H	-4.335473	-1.39387	3.639929
C	1.33148	-3.26817	4.712693
H	2.46226	-1.41088	4.801503
H	0.126842	-5.03251	4.310687
C	-0.749133	-5.60524	-1.73534
H	-2.846983	-5.53549	-1.17354
H	1.380103	-5.38839	-2.11903
P	-0.644876	1.885861	-0.0901
C	-0.783707	1.035736	-2.73431
C	-2.19898	-0.69746	-3.70214
C	-3.827207	-1.90971	-2.31362
C	-6.109788	-0.07336	2.061361
H	-4.688029	0.896464	-1.45142
C	-6.377343	0.968583	-0.11718
H	1.725095	-3.65439	5.659998
H	-0.839665	-6.55218	-2.27988
C	-0.647254	3.597204	-0.85882
C	-1.864676	2.237161	1.271842
C	-1.168319	0.278018	-3.82282
H	0.006412	1.784584	-2.85067
C	-2.643012	-1.4526	-4.82653
H	-4.301153	-2.0935	-1.34574
C	-4.246574	-2.62106	-3.42925
H	-6.499108	-0.34476	3.050122
C	-6.901401	0.615933	1.156201
H	-7.003679	1.518654	-0.82853
C	-1.459494	3.946436	-1.96224
C	0.104324	4.620317	-0.23721
C	-3.015556	3.033323	1.081716
C	-1.527389	1.821295	2.576546
H	-0.686104	0.42603	-4.79662
H	-2.171055	-1.26808	-5.79914
C	-3.65048	-2.39618	-4.69811
H	-5.044519	-3.36524	-3.32832
H	-7.928445	0.892451	1.419537
C	-1.497338	5.266636	-2.4405
H	-2.068935	3.185112	-2.45833
C	0.063674	5.93942	-0.71447
H	0.722883	4.393553	0.635344
C	-3.816184	3.393787	2.174404
H	-3.278001	3.385758	0.078911
C	-2.326457	2.190448	3.671059
H	-0.630418	1.209948	2.730541
H	-3.986733	-2.96785	-5.57016
C	-0.733178	6.267872	-1.82207
H	-2.133832	5.509706	-3.29919
H	0.659784	6.71127	-0.21485
C	-3.469942	2.978814	3.471978
H	-4.709156	4.007959	2.013078
H	-2.048234	1.864776	4.679631
H	-0.762222	7.29694	-2.19724
H	-4.08957	3.274785	4.326175
Pd	1.273038	0.346802	0.218495
C	2.676582	-1.01705	-0.4402
C	3.464626	-1.87606	0.348024
C	2.719868	-1.16907	-1.84676
C	4.26685	-2.86865	-0.24749
H	3.481276	-1.75909	1.436968
C	3.535936	-2.1489	-2.44349

H	2.095081	-0.53877	-2.49131
C	4.309576	-3.00756	-1.64441
H	4.867661	-3.52985	0.38947
H	3.556595	-2.24357	-3.53658
H	4.944546	-3.77238	-2.10698
C	3.149975	2.790308	-0.34098
C	2.842128	1.791293	0.78524
C	3.953187	2.171365	-1.49286
H	2.219627	3.23277	-0.73436
H	3.739247	3.636019	0.086416
C	4.04282	0.987885	1.206738
C	2.245449	2.464237	2.032453
C	5.227713	1.538415	-0.98836
H	4.180351	2.930519	-2.26532
H	3.332681	1.389572	-1.97658
O	4.095941	0.421942	2.319967
C	5.241314	0.944441	0.29832
H	2.153992	1.745579	2.862058
H	1.257746	2.915337	1.852652
H	2.925691	3.271999	2.38404
C	6.402931	1.512203	-1.76311
C	6.421724	0.337894	0.778447
H	6.39786	1.977058	-2.75741
C	7.57221	0.908699	-1.27854
H	6.390796	-0.10755	1.778134
C	7.581997	0.31702	-0.00134
H	8.479527	0.904484	-1.89435
H	8.49514	-0.15455	0.380125

(*S*)-**10**_conf3

Symbol	X	Y	Z
P	0.026266	1.418683	0.756588
C	1.346023	0.503838	1.720064
C	-1.166703	1.902286	2.101711
C	0.910226	3.005161	0.364608
C	2.468203	-0.02706	1.056216
C	1.210563	0.323064	3.129587
C	-2.214998	1.00058	2.395539
C	-1.092893	3.114444	2.82047
C	1.822447	3.571567	1.285089
C	0.662928	3.672441	-0.85256
C	2.629416	0.181252	-0.42195
C	3.468149	-0.74814	1.800694
C	2.168415	-0.35789	3.860458
H	0.33683	0.734749	3.641775
C	-3.154497	1.292736	3.396473
H	-2.315878	0.07742	1.811623
C	-2.038527	3.40866	3.817129
H	-0.30389	3.838924	2.595541
C	2.452631	4.792836	1.0035
H	2.046353	3.050736	2.222959
C	1.302681	4.89111	-1.13284
H	-0.031385	3.24161	-1.57905
C	1.790232	-0.47132	-1.33832
C	3.621711	1.122136	-0.90014
C	3.314961	-0.91166	3.226625
C	4.613328	-1.33085	1.174442
H	2.049264	-0.4801	4.943965
C	-3.067259	2.499203	4.110874
H	-3.963131	0.581858	3.597067
H	-1.971441	4.358117	4.360825
C	2.191417	5.457086	-0.20638
H	3.154705	5.221332	1.727997
H	1.100315	5.397064	-2.08337
P	0.404563	-1.6565	-0.93012
C	1.848078	-0.10752	-2.72489
C	3.712202	1.408307	-2.31182
C	4.53652	1.785915	-0.02651
C	4.307747	-1.62575	3.960529
H	4.736634	-1.22211	0.092741
C	5.560081	-2.02261	1.91566
H	-3.805048	2.734955	4.886317
H	2.686863	6.409166	-0.4285
C	0.731313	-2.99971	-2.20721
C	0.803791	-2.65685	0.584652
C	2.779933	0.795474	-3.19694
H	1.148851	-0.57496	-3.42609
C	4.715386	2.3013	-2.78854
H	4.472997	1.602272	1.049146
C	5.506466	2.647282	-0.52081
H	4.177595	-1.73893	5.043647
C	5.410918	-2.17014	3.321867
H	6.430798	-2.45902	1.413378
C	1.998095	-3.16696	-2.81505
C	-0.276208	-3.95363	-2.47326
C	1.923411	-3.5139	0.656385
C	-0.134593	-2.66806	1.636226
H	2.815691	1.044418	-4.26451
H	4.768541	2.500493	-3.86581
C	5.604597	2.905913	-1.91336
H	6.201089	3.134968	0.172367
H	6.167353	-2.71639	3.896403
C	2.240641	-4.24303	-3.68413
H	2.805423	-2.45548	-2.61728
C	-0.026118	-5.02739	-3.34341
H	-1.251834	-3.84727	-1.98544
C	2.110203	-4.34604	1.768677
H	2.644545	-3.54341	-0.16673
C	0.050133	-3.50798	2.746741
H	-1.022357	-2.03055	1.572525
H	6.375623	3.586689	-2.2911
C	1.227613	-5.17558	-3.956
H	3.229125	-4.34982	-4.14617
H	-0.823607	-5.75343	-3.53968
C	1.172465	-4.347	2.815491
H	2.987645	-5.0007	1.81433
H	-0.691428	-3.50974	3.553557
H	1.417072	-6.0138	-4.63644
H	1.314113	-5.0074	3.678762
Pd	-1.369035	0.166412	-0.85823
C	-2.203307	1.935291	-1.47917
C	-2.775606	2.957667	-0.69179
C	-2.033297	2.187839	-2.86232
C	-3.167787	4.183659	-1.26429
H	-2.910044	2.815586	0.385764
C	-2.413044	3.417605	-3.43325
H	-1.593384	1.420642	-3.51152
C	-2.986832	4.421486	-2.6352
H	-3.612565	4.957477	-0.626
H	-2.262878	3.584855	-4.50744
H	-3.29014	5.377058	-3.07864
C	-4.435442	-0.09936	-1.9245
C	-3.117546	-0.88395	-1.76216
C	-5.06112	0.411761	-0.6219
H	-4.289552	0.74619	-2.61831
H	-5.162333	-0.78355	-2.42102
C	-3.228371	-1.92836	-0.6994
C	-2.669144	-1.40899	-3.12774
C	-5.131302	-0.64731	0.44849
H	-6.069194	0.82636	-0.81572
H	-4.449645	1.252565	-0.24326
O	-2.575459	-2.99837	-0.70767
C	-4.214022	-1.72692	0.428411
H	-3.45307	-2.0648	-3.56679
H	-1.739683	-1.99208	-3.08684
H	-2.523816	-0.5698	-3.83181
C	-6.07334	-0.57486	1.494664
C	-4.25276	-2.69893	1.454253
H	-6.796846	0.250389	1.502724
C	-6.101536	-1.53978	2.51219
H	-3.540057	-3.52767	1.390853
C	-5.181127	-2.60576	2.496022
H	-6.848535	-1.46872	3.31195
H	-5.202091	-3.36489	3.286549

(*S*)-**10**_conf4

Symbol	X	Y	Z
P	0.66407	-1.75006	-0.1781
C	2.279062	-1.16794	-0.96545
C	0.349834	-3.33093	-1.1085
C	1.257533	-2.28771	1.489947
C	3.000423	-0.07729	-0.43986
C	2.772723	-1.82296	-2.13614
C	-0.100795	-3.22235	-2.44624
C	0.462274	-4.61557	-0.53852
C	2.385195	-3.12258	1.655906

C	0.570454	-1.82347	2.629279
C	2.598584	0.60047	0.836731
C	4.187423	0.394697	-1.11752
C	3.927928	-1.40749	-2.7724
H	2.237549	-2.68779	-2.53364
C	-0.391874	-4.36736	-3.20232
H	-0.214118	-2.23065	-2.90055
C	0.14969	-5.76063	-1.29108
H	0.787819	-4.72674	0.500302
C	2.794312	-3.50874	2.941105
H	2.946777	-3.46547	0.779424
C	0.987308	-2.20416	3.914278
H	-0.2904	-1.15717	2.490996
C	1.449168	1.401447	0.925438
C	3.434494	0.419215	2.008138
C	4.659251	-0.28492	-2.29857
C	4.913922	1.544355	-0.67404
H	4.288417	-1.94287	-3.6593
C	-0.270023	-5.64227	-2.62444
H	-0.729083	-4.2617	-4.23959
H	0.236487	-6.74972	-0.82734
C	2.094004	-3.05302	4.071281
H	3.667526	-4.16002	3.060651
H	0.443753	-1.837	4.791625
P	0.139196	1.603011	-0.36487
C	1.080524	1.977773	2.188263
C	3.076884	1.062983	3.248849
C	4.616263	-0.38376	1.993634
C	5.832878	0.1819	-2.96136
H	4.562953	2.083452	0.210274
C	6.045356	1.983147	-1.34685
H	-0.510194	-6.53714	-3.20918
H	2.418463	-3.35306	5.074
C	-0.211492	3.430209	-0.31958
C	0.792748	1.52821	-2.09978
C	1.874873	1.825784	3.30602
H	0.14994	2.549927	2.262102
C	3.917262	0.915539	4.390027
H	4.893838	-0.90631	1.074572
C	5.416437	-0.50833	3.121444
H	6.178097	-0.35526	-3.85315
C	6.517339	1.293886	-2.49698
H	6.580848	2.868695	-0.98658
C	0.691441	4.378289	0.209806
C	-1.374856	3.890185	-0.97224
C	1.867498	2.317013	-2.56068
C	0.047174	0.772709	-3.02888
H	1.583349	2.290853	4.255544
H	3.627764	1.419466	5.320178
C	5.073069	0.151654	4.330149
H	6.320247	-1.12647	3.077528
H	7.416254	1.646239	-3.01498
C	0.424445	5.752547	0.103289
H	1.606179	4.047184	0.711668
C	-1.636729	5.264091	-1.08022
H	-2.082861	3.170782	-1.39137
C	2.206302	2.322835	-3.92139
H	2.437081	2.934106	-1.85808
C	0.37947	0.790493	-4.39379
H	-0.807132	0.184293	-2.66843
H	5.714296	0.048504	5.21261
C	-0.740522	6.199488	-0.5398
H	1.134181	6.474538	0.52341
H	-2.549712	5.601958	-1.58292
C	1.4631	1.561855	-4.84071
H	3.05015	2.930652	-4.26621
H	-0.213983	0.206722	-5.10686
H	-0.94829	7.272464	-0.62007
H	1.724669	1.579131	-5.90482
Pd	-1.390923	-0.2006	0.161281
C	-2.718164	1.145579	0.960148
C	-2.571168	1.418267	2.33775
C	-3.665557	1.891549	0.231487
C	-3.324278	2.430943	2.959259
H	-1.881422	0.82336	2.948099
C	-4.427069	2.898836	0.854928
H	-3.845679	1.676467	-0.82859
C	-4.254304	3.17866	2.218376
H	-3.193338	2.620814	4.032148
H	-5.167293	3.456079	0.267298
H	-4.849083	3.961178	2.703967
C	-3.208585	-2.3361	-0.94276
C	-2.920105	-1.8236	0.480532
C	-3.919971	-1.29162	-1.81532
H	-2.280181	-2.67063	-1.4344
H	-3.857228	-3.24079	-0.87223
C	-4.166003	-1.28048	1.130487
C	-2.343769	-2.92497	1.378675
C	-5.208102	-0.83695	-1.16991
H	-4.116165	-1.68361	-2.83207
H	-3.236489	-0.41917	-1.92847
O	-4.327905	-1.28795	2.36394
C	-5.299427	-0.81736	0.243961
H	-3.070799	-3.76312	1.455761
H	-2.173556	-2.56267	2.403385
H	-1.407952	-3.3476	0.981396
C	-6.318681	-0.42608	-1.93261
C	-6.495495	-0.39662	0.86162
H	-6.25439	-0.45022	-3.02849
C	-7.501577	-0.00104	-1.31017
H	-6.522854	-0.40017	1.956178
C	-7.590562	0.013724	0.094759
H	-8.358386	0.309374	-1.9203
H	-8.514557	0.343624	0.584034

(*R*)-**11**_conf1

Symbol	X	Y	Z
P	0.980204	-1.60195	-0.69556
C	1.860704	-0.13461	-1.47591
C	0.931007	-2.87318	-2.06195
C	2.266042	-2.28257	0.449002
C	2.295892	0.968016	-0.70928
C	2.058663	-0.12326	-2.89115
C	0.039707	-2.65916	-3.14237
C	1.67889	-4.0692	-2.03015
C	3.590846	-2.46689	-0.01129
C	1.9381	-2.65484	1.767939
C	2.173205	1.000379	0.790189
C	2.956041	2.074663	-1.36462
C	2.676783	0.935977	-3.52967
H	1.728779	-0.97813	-3.48596
C	-0.061509	-3.59789	-4.18017
H	-0.601203	-1.76951	-3.15156
C	1.557235	-5.01561	-3.06263
H	2.35741	-4.27093	-1.19673
C	4.560086	-3.03806	0.825332
H	3.86001	-2.16395	-1.02965
C	2.916462	-3.21753	2.604877
H	0.923667	-2.50131	2.142562
C	0.958739	1.290634	1.441364
C	3.371462	0.766931	1.573674
C	3.145685	2.057962	-2.79425
C	3.441338	3.208141	-0.64063
H	2.824218	0.910852	-4.61635
C	0.697402	-4.78026	-4.14526
H	-0.750364	-3.40851	-5.01142
H	2.143635	-5.94053	-3.01551
C	4.223052	-3.4172	2.135903
H	5.582254	-3.17849	0.45595
H	2.648551	-3.49917	3.629132
P	-0.670666	1.609116	0.592236
C	0.902976	1.187859	2.871999
C	3.297131	0.757226	3.014345
C	4.655153	0.567425	0.977629
C	3.806991	3.148981	-3.43195
H	3.299416	3.243885	0.442956
C	4.088648	4.250767	-1.28708
H	0.609435	-5.51732	-4.95153
H	4.982237	-3.85932	2.791081
C	-1.480667	2.821221	1.757667
C	-0.339157	2.734573	-0.85552
C	2.026651	0.932169	3.631666
H	-0.053911	1.345687	3.378453
C	4.482379	0.578983	3.785213
H	4.73916	0.550658	-0.11211
C	5.79574	0.412469	1.754006
H	3.940018	3.115517	-4.52018
C	4.276161	4.224888	-2.69541
H	4.456009	5.104355	-0.70615

C	-0.804404	3.778953	2.549599
C	-2.891739	2.798048	1.790802
C	0.156299	4.050613	-0.73168
C	-0.710514	2.259236	-2.13189
H	1.953499	0.881785	4.725043
H	4.398262	0.577871	4.878954
C	5.715287	0.420153	3.171032
H	6.767565	0.277831	1.265487
H	4.788126	5.055258	-3.19465
C	-1.521219	4.682275	3.349375
H	0.290757	3.807428	2.558166
C	-3.608816	3.708981	2.583832
H	-3.434342	2.063946	1.185264
C	0.294437	4.867987	-1.86298
H	0.424156	4.447505	0.252868
C	-0.567231	3.080597	-3.26318
H	-1.133578	1.252035	-2.23424
H	6.622388	0.295973	3.773143
C	-2.926252	4.651689	3.367473
H	-0.979328	5.414804	3.959272
H	-4.704	3.672798	2.588171
C	-0.063937	4.383772	-3.13254
H	0.684245	5.886457	-1.75276
H	-0.857852	2.698007	-4.24863
H	-3.48371	5.3585	3.992833
H	0.042794	5.025885	-4.01466
Pd	-1.399344	-1.42307	-0.25901
C	-1.461335	-2.41666	1.492048
C	-1.419494	-3.8271	1.541571
C	-1.58707	-1.70577	2.706342
C	-1.497075	-4.50826	2.77139
H	-1.305766	-4.40664	0.617133
C	-1.666013	-2.3884	3.935628
H	-1.61395	-0.6099	2.696463
C	-1.620372	-3.79197	3.973378
H	-1.454481	-5.60467	2.786157
H	-1.763203	-1.81583	4.866727
H	-1.679995	-4.32244	4.930846
C	-4.590521	-2.27597	0.249454
C	-3.488988	-2.10226	-0.783
C	-4.902907	-0.99543	1.039975
H	-4.320888	-3.09079	0.946076
H	-5.506347	-2.61466	-0.28609
C	-3.307302	-0.78999	-1.37311
C	-3.226492	-3.34077	-1.6212
C	-5.090009	0.193013	0.124543
H	-5.793028	-1.13973	1.679398
H	-4.049653	-0.79615	1.720395
O	-2.350394	-0.59203	-2.23355
C	-4.235059	0.319511	-1.00233
H	-4.182006	-3.67466	-2.08269
H	-2.501755	-3.15195	-2.42602
H	-2.862217	-4.18112	-1.00393
C	-6.034169	1.203811	0.377482
C	-4.325498	1.456268	-1.83213
H	-6.700934	1.106529	1.243613
C	-6.12955	2.328263	-0.46051
H	-3.651642	1.53118	-2.69066
C	-5.268116	2.457456	-1.56347
H	-6.877124	3.102382	-0.25201
H	-5.334075	3.336202	-2.21452

(*R*)-**11**_conf2

Symbol	X	Y	Z
P	-1.829768	1.863244	-0.04199
C	-2.475011	0.584913	-1.24587
C	-1.578423	3.337018	-1.15476
C	-3.382795	2.339657	0.867064
C	-2.574913	-0.75971	-0.84124
C	-2.85631	0.948773	-2.5746
C	-0.366428	3.394213	-1.88361
C	-2.486783	4.412002	-1.26945
C	-4.675319	2.32489	0.296566
C	-3.239517	2.761462	2.206298
C	-2.210358	-1.19416	0.555224
C	-3.126878	-1.74116	-1.74375
C	-3.349667	0.01302	-3.4656
H	-2.751304	1.992076	-2.88811
C	-0.090282	4.482592	-2.72623
H	0.383439	2.600375	-1.77856
C	-2.200114	5.505942	-2.1034
H	-3.423615	4.397025	-0.70329
C	-5.79139	2.731217	1.044926
H	-4.807418	1.994662	-0.74036
C	-4.355138	3.170109	2.954732
H	-2.243478	2.763341	2.665059
C	-0.899689	-1.51925	0.954929
C	-3.292091	-1.32241	1.512082
C	-3.50813	-1.3475	-3.07918
C	-3.313836	-3.10738	-1.36778
H	-3.636349	0.313247	-4.48113
C	-1.005614	5.54215	-2.83996
H	0.853823	4.504918	-3.28249
H	-2.917205	6.332215	-2.17669
C	-5.633623	3.15573	2.375192
H	-6.787785	2.716693	0.587514
H	-4.225312	3.493315	3.993949
P	0.658415	-1.37149	-0.04935
C	-0.648016	-1.88456	2.319499
C	-3.016564	-1.73633	2.866735
C	-4.652721	-1.06333	1.159622
C	-4.036545	-2.32398	-3.9739
H	-3.040966	-3.41906	-0.35495
C	-3.840679	-4.03229	-2.25821
H	-0.784454	6.396214	-3.49049
H	-6.506291	3.470047	2.959084
C	1.591982	-2.92815	0.351206
C	0.333378	-1.67193	-1.85102
C	-1.66942	-1.9949	3.241117
H	0.379447	-2.07635	2.641476
C	-4.089327	-1.87563	3.795379
H	-4.884306	-0.74202	0.140951
C	-5.676715	-1.21137	2.084078
H	-4.315655	-2.00808	-4.98654
C	-4.202551	-3.64162	-3.57581
H	-3.981202	-5.07227	-1.94208
C	0.983785	-4.12356	0.789014
C	2.982236	-2.9196	0.103296
C	-0.10093	-2.93267	-2.31757
C	0.665192	-0.66752	-2.78279
H	-1.445083	-2.28072	4.275536
H	-3.855428	-2.19036	4.81971
C	-5.397536	-1.62163	3.414703
H	-6.710053	-1.0045	1.784631
H	-4.616495	-4.38072	-4.27098
C	1.753011	-5.2835	0.979247
H	-0.091254	-4.14761	0.997073
C	3.746529	-4.08242	0.284734
H	3.46209	-1.99061	-0.23031
C	-0.227576	-3.17273	-3.69237
H	-0.329871	-3.73428	-1.60857
C	0.537116	-0.91473	-4.16023
H	1.065779	0.28919	-2.43223
H	-6.214836	-1.73341	4.136042
C	3.133892	-5.26694	0.72641
H	1.267968	-6.2032	1.326062
H	4.824039	-4.05888	0.086559
C	0.089343	-2.16302	-4.61706
H	-0.57321	-4.15203	-4.04085
H	0.800804	-0.12716	-4.8751
H	3.731343	-6.1734	0.875656
H	-0.002865	-2.35577	-5.69217
Pd	1.999667	0.513444	0.446089
C	2.044205	0.117194	2.42871
C	1.205116	0.888375	3.262979
C	2.878163	-0.85172	3.027736
C	1.206131	0.702826	4.658002
H	0.529425	1.631206	2.821678
C	2.877185	-1.03718	4.424645
H	3.523423	-1.48627	2.409911
C	2.043694	-0.25996	5.245067
H	0.543529	1.312059	5.285636
H	3.533628	-1.79626	4.8686
H	2.045047	-0.40475	6.331781
C	4.468583	2.436067	1.613327
C	3.284443	2.370749	0.66162
C	5.474654	1.289057	1.423713
H	4.104422	2.440153	2.657238
H	4.979978	3.412992	1.457243

C	3.50055	1.78945	-0.64615
C	2.25328	3.467166	0.850075
C	5.845658	1.080097	-0.02758
H	6.379575	1.460337	2.035538
H	5.003043	0.361287	1.807286
O	2.505551	1.673769	-1.48098
C	4.84664	1.261314	-1.01907
H	2.75591	4.457868	0.805556
H	1.474839	3.444658	0.073124
H	1.770111	3.39848	1.841238
C	7.12022	0.640107	-0.42485
C	5.130407	0.980841	-2.37147
H	7.897813	0.497969	0.336322
C	7.404656	0.377304	-1.77517
H	4.332616	1.119828	-3.10798
C	6.40617	0.544378	-2.75085
H	8.407095	0.041884	-2.06561
H	6.624498	0.335985	-3.8044

(*S*)-**11**_conf1

Symbol	X	Y	Z
P	0.45467	-1.47806	0.138274
C	-0.795093	-1.19802	1.515696
C	1.760663	-2.58344	0.890401
C	-0.395284	-2.59968	-1.0587
C	-2.122626	-0.76505	1.295735
C	-0.323629	-1.3412	2.858123
C	2.71963	-1.98279	1.742279
C	1.90654	-3.9498	0.570078
C	-1.051709	-3.7679	-0.60484
C	-0.34429	-2.33657	-2.44264
C	-2.716369	-0.65287	-0.08287
C	-2.986336	-0.51583	2.429617
C	-1.13639	-1.09208	3.947386
H	0.700871	-1.67761	3.033075
C	3.768867	-2.73869	2.28525
H	2.649209	-0.91163	1.970116
C	2.966418	-4.70042	1.107555
H	1.203747	-4.4352	-0.11237
C	-1.619594	-4.66579	-1.51985
H	-1.124272	-3.97078	0.469403
C	-0.920177	-3.2367	-3.35465
H	0.145894	-1.43065	-2.80667
C	-2.460084	0.435504	-0.93889
C	-3.602696	-1.71467	-0.51814
C	-2.486052	-0.68251	3.771581
C	-4.348618	-0.1113	2.27246
H	-0.747169	-1.22619	4.964059
C	3.896259	-4.10184	1.970156
H	4.501647	-2.24635	2.933789
H	3.063467	-5.75929	0.840991
C	-1.550187	-4.40394	-2.89845
H	-2.130276	-5.56277	-1.15323
H	-0.871063	-3.01823	-4.42708
P	-1.366104	1.888567	-0.52749
C	-2.932835	0.372905	-2.29236
C	-4.128377	-1.7058	-1.86112
C	-3.999931	-2.78763	0.339496
C	-3.35104	-0.45488	4.882309
H	-4.744435	0.032742	1.263423
C	-5.170154	0.092422	3.371506
H	4.724377	-4.68909	2.38314
H	-1.995108	-5.10599	-3.61274
C	-2.173793	3.262836	-1.50632
C	-1.798403	2.442784	1.198974
C	-3.733566	-0.65822	-2.74016
H	-2.662439	1.174614	-2.98604
C	-5.018006	-2.73661	-2.28134
H	-3.608844	-2.82715	1.359535
C	-4.884869	-3.76739	-0.09075
H	-2.948464	-0.58811	5.893819
C	-4.671474	-0.08073	4.690474
H	-6.212449	0.395393	3.220374
C	-3.558194	3.372947	-1.77723
C	-1.323397	4.29683	-1.9552
C	-3.052439	2.97862	1.565353
C	-0.758889	2.420368	2.154377
H	-4.088907	-0.67025	-3.7779
H	-5.401921	-2.70726	-3.30851
C	-5.401321	-3.74739	-1.41264
H	-5.183679	-4.56578	0.598221
H	-5.32971	0.085207	5.550718
C	-4.069979	4.481493	-2.46897
H	-4.241178	2.575912	-1.46359
C	-1.835199	5.411005	-2.64081
H	-0.247581	4.22813	-1.76008
C	-3.265468	3.470762	2.861028
H	-3.86508	3.030073	0.83369
C	-0.977481	2.913522	3.452111
H	0.227775	2.025214	1.885369
H	-6.09628	-4.52763	-1.74292
C	-3.2107	5.5061	-2.90073
H	-5.145865	4.545367	-2.6701
H	-1.154523	6.201429	-2.97736
C	-2.228265	3.438876	3.808822
H	-4.244899	3.882509	3.13097
H	-0.159341	2.890193	4.180986
H	-3.612432	6.370961	-3.44079
H	-2.396245	3.828978	4.819436
Pd	1.785616	0.453457	-0.41816
C	2.089722	0.036878	-2.36554
C	2.898391	-1.04738	-2.77486
C	1.518888	0.862687	-3.35875
C	3.132117	-1.29435	-4.141
H	3.332761	-1.72065	-2.02621
C	1.749107	0.609785	-4.72536
H	0.875884	1.703355	-3.06929
C	2.558831	-0.46754	-5.12142
H	3.761227	-2.14335	-4.43673
H	1.29064	1.260329	-5.48104
H	2.739095	-0.66273	-6.18499
C	4.413976	2.247195	-1.32886
C	3.187818	2.194241	-0.42922
C	5.390428	1.078801	-1.10528
H	4.096215	2.266402	-2.38773
H	4.93931	3.211104	-1.14113
C	3.336801	1.557705	0.86929
C	2.263235	3.393575	-0.55436
C	5.714818	0.889541	0.35854
H	6.314788	1.22742	-1.69312
H	4.911202	0.156123	-1.49038
O	2.302884	1.415781	1.644972
C	4.674044	1.064074	1.306701
H	2.845509	4.327226	-0.39183
H	1.447618	3.36139	0.183559
H	1.828459	3.457789	-1.56788
C	6.985368	0.497399	0.814096
C	4.918707	0.845869	2.678725
H	7.794013	0.355512	0.086234
C	7.228491	0.2865	2.181508
H	4.096158	0.998833	3.385451
C	6.19364	0.462961	3.117279
H	8.228953	-0.00975	2.517543
H	6.384352	0.310205	4.185794

(*S*)-**11**_conf2

Symbol	X	Y	Z
C	5.392661	3.109431	0.073701
C	5.330709	3.858484	1.260341
C	4.373863	3.526717	2.2337
C	3.485317	2.454434	2.021746
C	3.538192	1.694224	0.832946
C	4.504358	2.038386	-0.13903
H	6.132914	3.359736	-0.69672
H	6.021655	4.693538	1.425216
H	4.314625	4.10294	3.165865
H	2.74003	2.223044	2.792609
H	4.565973	1.473094	-1.07717
O	1.787869	-2.1058	0.468812
C	3.088572	-2.0633	0.482747
C	3.773815	-1.3377	1.531582
C	3.872283	-2.67946	-0.63155
C	5.290831	-1.18404	1.388456
C	5.266784	-2.88866	-0.47589
C	5.932877	-2.46696	0.82174
H	5.734702	-0.93979	2.370619

H	5.531365	-0.33495	0.721635
H	7.01745	-2.31887	0.662938
H	5.836038	-3.29077	1.560654
C	5.976422	-3.51318	-1.51612
H	7.056271	-3.67468	-1.40708
C	3.215834	-3.11048	-1.80137
H	2.135605	-2.94598	-1.87597
C	3.936112	-3.73452	-2.82808
H	3.42336	-4.06732	-3.73792
C	5.321035	-3.93164	-2.68624
H	5.893086	-4.41364	-3.4876
C	3.214255	-1.35721	2.947016
H	3.370066	-0.39236	3.462822
H	2.138895	-1.59848	2.949913
H	3.741839	-2.13109	3.545892
P	0.533201	1.353433	-0.30286
C	-0.495067	0.251425	-1.39848
C	0.911799	2.780994	-1.43267
C	-0.400765	2.189005	1.052852
C	-1.884905	0.057808	-1.45734
C	0.355864	-0.42112	-2.33835
C	0.511087	2.823789	-2.78469
C	1.651625	3.865216	-0.90548
C	-1.221676	3.315212	0.831334
C	-0.153837	1.764562	2.376044
C	-2.436125	-0.75949	-2.52454
C	-2.869898	0.634889	-0.47563
C	-0.156291	-1.19697	-3.35809
H	1.441923	-0.30249	-2.24122
C	0.838747	3.927219	-3.59078
H	-0.054291	1.993257	-3.21728
C	1.971064	4.96568	-1.7133
H	1.984574	3.847713	0.136839
C	-1.799035	3.992189	1.916039
H	-1.405076	3.668093	-0.18812
C	-0.722213	2.45353	3.460184
H	0.499091	0.89934	2.54395
C	-1.56199	-1.37199	-3.49522
C	-3.842089	-0.96827	-2.67361
C	-3.105956	0.001401	0.761824
C	-3.667766	1.767014	-0.87442
H	0.517122	-1.6838	-4.07366
C	1.567463	5.001108	-3.05861
H	0.517602	3.943088	-4.63866
H	2.547327	5.79451	-1.28752
C	-1.547227	3.566646	3.231114
H	-2.441057	4.860285	1.731061
H	-0.506854	2.127524	4.484321
C	-2.114895	-2.13612	-4.56393
H	-4.525136	-0.52673	-1.94372
C	-4.352532	-1.70956	-3.73005
P	-1.912143	-1.31119	1.355221
C	-4.158158	0.487536	1.597916
C	-4.715925	2.244254	-0.00346
C	-3.454728	2.457338	-2.10866
H	1.822438	5.860897	-3.68858
H	-1.98541	4.109036	4.076707
H	-1.429785	-2.59148	-5.28931
C	-3.485775	-2.29855	-4.68768
H	-5.436143	-1.84343	-3.82296
C	-2.593864	-1.79852	3.017351
C	-2.313386	-2.79427	0.319257
C	-4.941603	1.566049	1.226822
H	-4.350023	-0.01146	2.552498
C	-5.496589	3.371339	-0.39715
H	-2.662129	2.110771	-2.7786
C	-4.225595	3.556627	-2.45933
H	-3.900851	-2.88193	-5.51718
C	-2.469392	-0.87402	4.083548
C	-3.092498	-3.08725	3.309058
C	-3.628952	-3.19175	-0.00866
C	-1.224631	-3.58016	-0.11811
H	-5.75214	1.909755	1.881663
H	-6.29081	3.717605	0.27576
C	-5.259337	4.019017	-1.59983
H	-4.037298	4.071363	-3.40821
C	-2.862887	-1.21518	5.385718
H	-2.065711	0.126088	3.886602
C	-3.470074	-3.43303	4.617578
H	-3.184831	-3.82862	2.509479
C	-3.852784	-4.36171	-0.75004
H	-4.478945	-2.58379	0.322473
C	-1.455008	-4.75304	-0.85776
H	-0.199994	-3.26251	0.112129
H	-5.864951	4.884798	-1.8902
C	-3.363582	-2.49934	5.659462
H	-2.767963	-0.47815	6.191917
H	-3.853265	-4.44024	4.819448
C	-2.765243	-5.14601	-1.17256
H	-4.877181	-4.66179	-1.00002
H	-0.604413	-5.35877	-1.19119
H	-3.661154	-2.77086	6.678689
H	-2.941809	-6.0603	-1.75113
Pd	2.341711	0.09918	0.5407

TS$_{RE}$OR

Symbol	X	Y	Z
C	-2.945289	4.374951	0.935261
C	-3.627238	4.644263	-0.26172
C	-3.768249	3.603407	-1.20525
C	-3.266731	2.322021	-0.94034
C	-2.577252	2.023369	0.27509
C	-2.409033	3.099383	1.188237
H	-2.801451	5.170697	1.677541
H	-4.039444	5.639665	-0.46159
H	-4.290653	3.789255	-2.15302
H	-3.436145	1.521484	-1.67177
H	-1.859834	2.941217	2.125236
Pd	-1.069706	0.455625	-0.09566
O	-2.840324	-0.85325	-0.36756
C	-3.813654	-0.46559	0.393879
C	-3.623483	0.480215	1.458697
C	-5.163838	-1.00592	0.119702
C	-4.873923	1.089317	2.08115
C	-6.30919	-0.26382	0.520818
C	-6.08762	1.10089	1.136003
H	-4.638926	2.122526	2.40389
H	-5.130462	0.527274	3.007406
H	-6.994638	1.442159	1.668252
H	-5.898141	1.835449	0.328092
C	-7.584921	-0.79156	0.268037
H	-8.469548	-0.21771	0.57217
C	-5.325586	-2.23756	-0.55619
H	-4.431048	-2.77399	-0.88772
C	-6.605994	-2.75596	-0.78421
H	-6.722385	-3.71999	-1.29293
C	-7.740181	-2.03483	-0.36988
H	-8.744666	-2.43498	-0.55
C	-2.56546	0.171238	2.513578
H	-3.008038	-0.45877	3.31433
H	-1.682599	-0.35774	2.1106
H	-2.20643	1.096905	2.999433
P	0.842741	1.742465	-0.07543
C	1.77519	0.933092	1.305495
C	0.956242	3.557428	0.286232
C	1.823525	1.753432	-1.65004
C	2.576206	-0.21962	1.191406
C	1.417701	1.422164	2.605835
C	1.983967	4.137981	1.062068
C	0.040062	4.397136	-0.38163
C	3.143204	2.249521	-1.71236
C	1.161008	1.413726	-2.84593
C	3.081548	-0.85253	2.395202
C	2.966878	-0.8058	-0.13821
C	1.884434	0.82327	3.757893
H	0.757843	2.292502	2.68192
C	2.090861	5.533709	1.166744
H	2.703632	3.5009	1.587267
C	0.157078	5.792384	-0.28086
H	-0.768896	3.960472	-0.97701
C	3.790784	2.382792	-2.94851
H	3.665961	2.539114	-0.79487
C	1.806884	1.558703	-4.08487
H	0.130454	1.044257	-2.79386
C	2.7333	-0.31835	3.69032
C	3.931936	-2.00111	2.361781
C	2.057204	-1.51551	-0.95158
C	4.338904	-0.65239	-0.5691
H	1.602446	1.220444	4.74044

C	1.179366	6.363691	0.492972
H	2.892731	5.971634	1.772461
H	-0.566678	6.429831	-0.80001
C	3.122935	2.041797	-4.13767
H	4.819453	2.758398	-2.98275
H	1.277611	1.294713	-5.00743
C	3.239109	-0.93629	4.870891
H	4.218841	-2.42201	1.39452
C	4.414253	-2.5787	3.528584
P	0.240158	-1.6271	-0.53358
C	2.515014	-2.08261	-2.18204
C	4.776186	-1.25145	-1.80675
C	5.295308	0.104898	0.178239
H	1.265973	7.45336	0.573297
H	3.628603	2.157109	-5.10317
H	2.958885	-0.51178	5.842585
C	4.067492	-2.04575	4.797501
H	5.069514	-3.45498	3.467501
C	-0.494953	-2.50525	-1.99431
C	0.14068	-2.94995	0.760019
C	3.830988	-1.97065	-2.58891
H	1.805955	-2.62356	-2.81373
C	6.130603	-1.09839	-2.22597
H	4.982733	0.580607	1.112094
C	6.603978	0.246468	-0.26138
H	4.452398	-2.51066	5.711884
C	-1.232109	-1.75297	-2.93222
C	-0.367415	-3.89945	-2.18478
C	1.234975	-3.7585	1.129238
C	-1.119519	-3.18141	1.357621
H	4.157407	-2.4291	-3.5304
H	6.441959	-1.56805	-3.16697
C	7.032317	-0.36534	-1.47023
H	7.314073	0.834261	0.331269
C	-1.806508	-2.37386	-4.05261
H	-1.382274	-0.6821	-2.75724
C	-0.944811	-4.51925	-3.30373
H	0.181913	-4.50158	-1.45261
C	1.076516	-4.77105	2.089789
H	2.211665	-3.59997	0.661993
C	-1.273885	-4.19911	2.31011
H	-1.977043	-2.5716	1.052574
H	8.070101	-0.25234	-1.80314
C	-1.66114	-3.75724	-4.24255
H	-2.382905	-1.77684	-4.7683
H	-0.838654	-5.60177	-3.43934
C	-0.175745	-4.99244	2.683872
H	1.937284	-5.38782	2.372169
H	-2.256873	-4.37056	2.763486
H	-2.114997	-4.24395	-5.11337
H	-0.296588	-5.78198	3.434308

TS$_{RE}$OS

Symbol	X	Y	Z
P	-0.706609	1.743798	-0.26214
C	-1.604652	0.654062	-1.46918
C	-0.765083	3.373281	-1.16448
C	-1.761227	2.17304	1.203525
C	-2.530418	-0.36063	-1.16656
C	-1.131042	0.797777	-2.81674
C	-1.857622	3.72661	-1.99234
C	0.261352	4.313826	-0.95437
C	-3.051335	2.733516	1.096451
C	-1.168415	2.059078	2.477774
C	-3.047125	-1.19658	-2.23488
C	-3.005043	-0.63073	0.231607
C	-1.615795	0.012587	-3.84158
H	-0.374079	1.557267	-3.03551
C	-1.917089	4.993443	-2.59097
H	-2.65811	3.004813	-2.18669
C	0.198004	5.581279	-1.55774
H	1.112821	4.061546	-0.31886
C	-3.740137	3.14972	2.244695
H	-3.520766	2.849427	0.114829
C	-1.854569	2.485059	3.626716
H	-0.154549	1.644114	2.546924
C	-2.597184	-0.9894	-3.58992
C	-3.997941	-2.23809	-2.00468
C	-2.160276	-1.24011	1.181434
C	-4.370227	-0.31869	0.582285
H	-1.247292	0.152795	-4.86495
C	-0.887894	5.926015	-2.37518
H	-2.769639	5.250169	-3.23054
H	1.013487	6.292544	-1.38668
C	-3.14323	3.02858	3.511434
H	-4.746092	3.572989	2.148695
H	-1.378857	2.397382	4.610305
C	-3.12781	-1.79023	-4.64186
H	-4.338812	-2.43214	-0.9845
C	-4.499907	-3.00139	-3.0507
P	-0.35818	-1.52801	0.778971
C	-2.683833	-1.58181	2.466369
C	-4.87114	-0.66452	1.890359
C	-5.25641	0.348729	-0.31972
H	-0.933704	6.914362	-2.8469
H	-3.681176	3.363687	4.40558
H	-2.772823	-1.61069	-5.66403
C	-4.06942	-2.77548	-4.38366
H	-5.233496	-3.78836	-2.842
C	0.290772	-2.3582	2.305077
C	-0.428411	-2.91205	-0.44806
C	-3.997479	-1.31376	2.806497
H	-2.035961	-2.07786	3.193836
C	-6.219454	-0.35036	2.232631
H	-4.890736	0.623202	-1.31369
C	-6.561221	0.649138	0.045359
H	-4.473385	-3.38265	-5.20146
C	0.387325	-1.60829	3.50236
C	0.776773	-3.68291	2.297126
C	-1.420518	-3.91435	-0.3678
C	0.555196	-2.98742	-1.4576
H	-4.379238	-1.59956	3.794345
H	-6.583793	-0.62551	3.229994
C	-7.052521	0.293723	1.330672
H	-7.219227	1.162424	-0.66495
C	0.924811	-2.17963	4.664942
H	0.028897	-0.57237	3.524392
C	1.331239	-4.24602	3.458401
H	0.722747	-4.27619	1.379397
C	-1.429793	-4.9757	-1.2846
H	-2.187141	-3.86198	0.413317
C	0.538735	-4.0542	-2.37259
H	1.342444	-2.22773	-1.50156
H	-8.086271	0.531179	1.605728
C	1.402313	-3.50122	4.645844
H	0.980465	-1.58673	5.585049
H	1.705882	-5.27567	3.432007
C	-0.450593	-5.04645	-2.29005
H	-2.205579	-5.74682	-1.21586
H	1.304541	-4.10502	-3.15507
H	1.832284	-3.94468	5.55091
H	-0.461559	-5.8742	-3.00848
O	2.723742	-1.02163	-0.34733
C	3.88727	-0.63315	0.046876
C	4.053052	0.356424	1.075623
C	5.08445	-1.22955	-0.59293
C	2.728492	1.95038	0.181567
C	5.438261	0.957254	1.262328
C	3.346723	0.121449	2.401687
C	6.314634	-0.51673	-0.58249
C	5.012242	-2.49687	-1.21481
C	3.206132	2.27296	-1.12213
C	2.755176	2.993885	1.146703
C	6.323075	0.877539	0.005698
H	5.326027	2.013883	1.575797
H	5.945416	0.439373	2.108071
H	3.997569	-0.48107	3.070894
H	2.388495	-0.4117	2.28831
H	3.148908	1.075421	2.9246
C	7.446091	-1.10311	-1.1715
H	4.050404	-3.0194	-1.22674
C	6.154467	-3.07283	-1.78379
C	3.717192	3.542172	-1.42399
H	3.20278	1.498564	-1.89984
C	3.28861	4.263455	0.847428
H	2.369438	2.818364	2.158866
H	7.356028	1.198017	0.235315
H	5.92955	1.590258	-0.74563
H	8.395128	-0.55211	-1.1687

C	7.376173	-2.37658	-1.76237
H	6.093796	-4.0635	-2.24914
C	3.779639	4.54973	-0.437
H	4.081195	3.747238	-2.43915
H	3.300733	5.03662	1.626925
H	8.272737	-2.82179	-2.20899
H	4.198878	5.53496	-0.66982
Pd	1.143331	0.432986	0.254622

TS$_{RE}$CR_conf1

Symbol	X	Y	Z
C	4.252752	-1.85741	1.931228
C	4.296402	-3.1697	1.438196
C	3.656221	-3.4637	0.21969
C	2.988857	-2.46342	-0.49774
C	2.922658	-1.13126	-0.01103
C	3.597433	-0.84525	1.207802
H	4.758712	-1.60247	2.870772
H	4.83081	-3.95314	1.986895
H	3.671944	-4.48641	-0.17586
H	2.501334	-2.72939	-1.44075
H	3.627033	0.17873	1.59264
Pd	1.181712	0.025048	-0.16634
C	3.266806	0.276712	-1.56102
C	4.712871	-0.21597	-1.58573
C	3.311716	1.822038	-1.44593
C	2.548308	-0.1663	-2.83799
O	5.07079	-1.14344	-2.32362
C	5.723337	0.509557	-0.73743
C	4.038484	2.329643	-0.19663
H	2.304449	2.261334	-1.51349
H	3.875944	2.188443	-2.33667
H	3.090222	0.229177	-3.72246
H	2.549499	-1.26143	-2.94742
H	1.509962	0.206841	-2.87441
C	5.420235	1.723415	-0.07091
C	7.021705	-0.03715	-0.64704
H	4.116856	3.433535	-0.22172
H	3.425935	2.081799	0.697266
C	6.429504	2.355141	0.68191
H	7.217655	-0.96851	-1.18823
C	8.010102	0.593565	0.113504
H	6.205557	3.303176	1.187719
C	7.711219	1.796065	0.78217
H	9.012606	0.156876	0.186835
H	8.482569	2.303627	1.373289
P	-0.558413	-1.64735	0.326711
C	-2.149971	-1.09901	1.150291
C	0.048532	-2.97207	1.479081
C	-1.171684	-2.61358	-1.13207
C	-3.004458	-0.15342	0.54331
C	-2.451196	-1.56146	2.46969
C	0.785136	-2.55407	2.610376
C	-0.166727	-4.35263	1.284065
C	-2.323871	-3.42897	-1.06855
C	-0.441068	-2.55919	-2.33615
C	-2.788203	0.315543	-0.8689
C	-4.15214	0.34425	1.264805
C	-3.55718	-1.10794	3.164261
H	-1.794489	-2.29955	2.937443
C	1.260009	-3.48875	3.541879
H	0.999999	-1.48919	2.749784
C	0.321626	-5.2881	2.210644
H	-0.715051	-4.7027	0.404131
C	-2.721582	-4.18515	-2.18198
H	-2.912829	-3.46741	-0.14533
C	-0.839256	-3.31667	-3.44931
H	0.437782	-1.90879	-2.39567
C	-1.766708	1.217194	-1.21981
C	-3.678698	-0.18593	-1.8981
C	-4.430997	-0.14081	2.594855
C	-5.029877	1.331485	0.716972
H	-3.770903	-1.49189	4.169464
C	1.028478	-4.85987	3.345218
H	1.828656	-3.14508	4.413139
H	0.147027	-6.35714	2.041967
C	-1.978097	-4.1337	-3.37296
H	-3.618981	-4.81123	-2.12058
H	-0.260569	-3.26256	-4.37839
P	-0.431769	1.825427	-0.08393
C	-1.560553	1.543448	-2.60213
C	-3.486798	0.21009	-3.27225
C	-4.763637	-1.06981	-1.60892
C	-5.562781	0.357811	3.304409
H	-4.830386	1.718946	-0.28642
C	-6.120708	1.802106	1.434263
H	1.407634	-5.59191	4.067431
H	-2.292163	-4.72316	-4.24181
C	-0.217249	3.609945	-0.60052
C	-1.113089	2.1536	1.612267
C	-2.393802	1.065037	-3.5929
H	-0.725453	2.196842	-2.8759
C	-4.379644	-0.26341	-4.277
H	-4.921952	-1.39609	-0.57778
C	-5.622155	-1.5093	-2.60765
H	-5.7583	-0.02716	4.312785
C	-6.396209	1.31061	2.738897
H	-6.776606	2.559181	0.989427
C	-1.174271	4.369448	-1.30982
C	0.958307	4.255601	-0.15737
C	-2.186103	3.037748	1.853396
C	-0.419459	1.601987	2.708514
H	-2.222565	1.341971	-4.6403
H	-4.214013	0.0532	-5.31407
C	-5.435072	-1.10303	-3.95475
H	-6.451098	-2.17912	-2.35216
H	-7.262869	1.686928	3.29389
C	-0.949195	5.728777	-1.5825
H	-2.10045	3.89929	-1.65636
C	1.177809	5.616271	-0.42091
H	1.700995	3.687212	0.414058
C	-2.566764	3.347042	3.166838
H	-2.720582	3.493749	1.013446
C	-0.795983	1.91863	4.024071
H	0.42334	0.926582	2.511019
H	-6.118948	-1.45648	-4.73451
C	0.226918	6.356464	-1.14175
H	-1.702624	6.300083	-2.13727
H	2.096079	6.097392	-0.06509
C	-1.870998	2.790802	4.254385
H	-3.407253	4.027804	3.341858
H	-0.246484	1.485109	4.867533
H	0.399769	7.417012	-1.35636
H	-2.16562	3.041988	5.279769

TS$_{RE}$CR_conf2

Symbol	X	Y	Z
C	-4.000716	-2.11393	-3.0705
C	-4.610116	-2.92885	-2.10387
C	-4.4739	-2.59062	-0.74535
C	-3.772783	-1.438	-0.36002
C	-3.162966	-0.58831	-1.32442
C	-3.288332	-0.96397	-2.68921
H	-4.076695	-2.37046	-4.13478
H	-5.170903	-3.82193	-2.40071
H	-4.908703	-3.2369	0.027133
H	-3.663383	-1.21319	0.706504
H	-2.822889	-0.35006	-3.46819
Pd	-1.344977	0.312459	-0.81508
C	-3.495948	1.464327	-1.20468
C	-2.915516	2.285691	-0.07591
C	-5.032839	1.360713	-1.00652
C	-3.240061	2.095429	-2.57773
O	-2.159143	3.264355	-0.26464
C	-3.44225	2.035405	1.31571
C	-5.5549	0.949588	0.381176
H	-5.460186	0.693541	-1.77662
H	-5.410086	2.38295	-1.23216
H	-3.598779	3.143661	-2.56738
H	-2.175261	2.108991	-2.85486
H	-3.797812	1.55858	-3.36319
C	-4.706962	1.438114	1.537758
C	-2.692215	2.519782	2.411043
H	-6.589338	1.327403	0.500313
H	-5.635752	-0.15063	0.433327
C	-5.180298	1.325016	2.861982

H	-1.731795	2.999877	2.200982
C	-3.167736	2.388535	3.719149
H	-6.167469	0.878766	3.038308
C	-4.421739	1.787792	3.945229
H	-2.573317	2.760176	4.561482
H	-4.812992	1.694574	4.9652
P	-0.105559	-1.61229	0.100193
C	1.743578	-1.81701	-0.1412
C	-0.760278	-3.22315	-0.55163
C	-0.299608	-1.78519	1.932958
C	2.656076	-0.84882	0.326586
C	2.236595	-2.93552	-0.88487
C	-0.853393	-3.36504	-1.95521
C	-1.178519	-4.2941	0.265444
C	0.441881	-2.72555	2.683351
C	-1.243381	-0.97316	2.593355
C	2.236478	0.314669	1.180208
C	4.064658	-0.9884	0.027233
C	3.582514	-3.09423	-1.15535
H	1.53302	-3.69288	-1.23859
C	-1.3112	-4.56175	-2.52516
H	-0.559395	-2.53155	-2.60247
C	-1.651209	-5.48611	-0.30748
H	-1.134219	-4.20089	1.354771
C	0.224376	-2.86154	4.06285
H	1.191543	-3.35062	2.185097
C	-1.458527	-1.1096	3.97447
H	-1.797533	-0.21946	2.02096
C	1.560224	1.435604	0.664818
C	2.6087	0.290429	2.582921
C	4.532432	-2.12867	-0.72235
C	5.032734	-0.01417	0.428088
H	3.931197	-3.97188	-1.71356
C	-1.711489	-5.6274	-1.70241
H	-1.368501	-4.65599	-3.61545
H	-1.970864	-6.30829	0.343433
C	-0.729289	-2.05708	4.709615
H	0.806322	-3.59307	4.635088
H	-2.192666	-0.46448	4.468866
P	0.780692	1.59151	-1.01229
C	1.212474	2.515337	1.5454
C	2.284351	1.408919	3.434204
C	3.300827	-0.8115	3.173319
C	5.921497	-2.26017	-1.01692
H	4.700383	0.864646	0.987946
C	6.377326	-0.16401	0.118302
H	-2.076123	-6.56026	-2.14744
H	-0.894427	-2.16301	5.787987
C	1.122939	3.387086	-1.40208
C	1.830734	0.805146	-2.32992
C	1.571838	2.509345	2.877349
H	0.654494	3.366781	1.142336
C	2.675865	1.393036	4.804382
H	3.544388	-1.67853	2.553732
C	3.671039	-0.79897	4.511488
H	6.254107	-3.13861	-1.58349
C	6.831946	-1.29909	-0.60578
H	7.095262	0.600001	0.437659
C	2.355667	4.0044	-1.08711
C	0.153082	4.128753	-2.10514
C	3.215981	1.019028	-2.4881
C	1.143523	0.056519	-3.3094
H	1.311509	3.357173	3.52293
H	2.421106	2.257488	5.429823
C	3.362555	0.312895	5.337965
H	4.204828	-1.6583	4.933119
H	7.896832	-1.40929	-0.83946
C	2.617187	5.322906	-1.48683
H	3.109484	3.459777	-0.50803
C	0.419032	5.44811	-2.50924
H	-0.830612	3.689701	-2.2861
C	3.896291	0.487698	-3.59362
H	3.772564	1.599535	-1.74655
C	1.822065	-0.46672	-4.42237
H	0.063634	-0.10617	-3.18616
H	3.661492	0.312922	6.392203
C	1.65132	6.046194	-2.20774
H	3.577297	5.787099	-1.2326
H	-0.349383	6.012491	-3.04983
C	3.201272	-0.2522	-4.56552
H	4.974493	0.654013	-3.69557
H	1.271158	-1.04	-5.17705
H	1.85646	7.076453	-2.52065
H	3.735258	-0.65821	-5.43235

TS_{RE}CR_conf3

Symbol	X	Y	Z
C	-3.261671	-3.81944	1.852083
C	-3.95288	-3.29165	2.956041
C	-4.23558	-1.91876	2.984425
C	-3.838541	-1.07677	1.929774
C	-3.128224	-1.58852	0.810988
C	-2.87125	-2.98895	0.789111
H	-3.033463	-4.89121	1.803598
H	-4.274753	-3.94446	3.775411
H	-4.764934	-1.48076	3.838796
H	-4.082312	-0.01343	2.01855
H	-2.363466	-3.45654	-0.06162
Pd	-1.525328	-0.45545	0.001675
C	-3.785681	-0.85613	-1.01172
C	-2.841983	-0.18492	-2.01204
C	-4.92908	0.070837	-0.58256
C	-4.314728	-2.16177	-1.59982
O	-2.165141	-0.86748	-2.8186
C	-2.967197	1.304476	-2.23024
C	-4.430997	1.476376	-0.20793
H	-5.506591	-0.40034	0.22832
H	-5.619945	0.169523	-1.44755
H	-4.902171	-1.91635	-2.50882
H	-3.513249	-2.8337	-1.93461
H	-4.975931	-2.69091	-0.8948
C	-3.798201	2.117764	-1.41635
C	-2.32505	1.866574	-3.35501
H	-5.241815	2.099445	0.210003
H	-3.632202	1.36424	0.55717
C	-3.982038	3.46849	-1.75575
H	-1.699995	1.207797	-3.96547
C	-2.507947	3.216729	-3.67822
H	-4.632874	4.093826	-1.13208
C	-3.346653	4.018453	-2.88143
H	-2.008621	3.643847	-4.55542
H	-3.511611	5.070948	-3.14028
P	-0.181333	1.138623	1.27135
C	1.584529	0.63062	1.670087
C	-0.979196	1.254325	2.94489
C	0.11769	2.932505	0.865762
C	2.510306	0.401792	0.633616
C	2.012466	0.516661	3.029382
C	-1.082292	0.089704	3.742885
C	-1.596977	2.439215	3.404223
C	0.87426	3.757351	1.731286
C	-0.38911	3.476711	-0.32851
C	2.178205	0.582974	-0.82614
C	3.876544	0.056418	0.962212
C	3.312896	0.180675	3.357004
H	1.297336	0.710705	3.832787
C	-1.730762	0.126358	4.985782
H	-0.649106	-0.85188	3.387165
C	-2.272561	2.46442	4.635894
H	-1.544244	3.351062	2.801468
C	1.100339	5.103551	1.412858
H	1.286792	3.34256	2.658112
C	-0.151439	4.824733	-0.65081
H	-0.965286	2.845897	-1.01231
C	1.503883	-0.39265	-1.59142
C	2.674758	1.779721	-1.47459
C	4.277808	-0.06483	2.342492
C	4.867283	-0.18296	-0.04123
H	3.614723	0.107056	4.409169
C	-2.330269	1.314185	5.43821
H	-1.783064	-0.78422	5.592983
H	-2.744827	3.394873	4.972169
C	0.587152	5.639799	0.218234
H	1.683101	5.733939	2.094873
H	-0.550267	5.229077	-1.58695
P	0.538346	-1.80796	-0.87893
C	1.281412	-0.15574	-2.98902
C	2.474878	1.968412	-2.89159
C	3.373777	2.802899	-0.76162

C	5.622435	-0.41896	2.659935
H	4.587599	-0.08968	-1.09442
C	6.168616	-0.52599	0.297561
H	-2.843615	1.340165	6.406118
H	0.769233	6.690931	-0.03388
C	0.574446	-3.06526	-2.25747
C	1.576667	-2.69934	0.380638
C	1.762205	0.973963	-3.61915
H	0.705679	-0.88566	-3.56417
C	2.983931	3.137171	-3.52874
H	3.522299	2.692794	0.315604
C	3.854199	3.932666	-1.40873
H	5.90346	-0.50712	3.716578
C	6.554543	-0.64714	1.659974
H	6.90648	-0.7015	-0.49365
C	1.75559	-3.38	-2.96977
C	-0.606348	-3.76228	-2.57969
C	2.95084	-2.98664	0.236354
C	0.890307	-3.21492	1.500422
H	1.594119	1.118468	-4.6936
H	2.822614	3.255514	-4.60742
C	3.666022	4.103762	-2.80572
H	4.382957	4.700953	-0.83354
H	7.585543	-0.91671	1.91537
C	1.761874	-4.37459	-3.95714
H	2.673631	-2.81503	-2.77734
C	-0.59923	-4.76377	-3.56781
H	-1.561691	-3.5175	-2.10135
C	3.618964	-3.77362	1.185254
H	3.512342	-2.58712	-0.61304
C	1.557342	-4.01228	2.446275
H	-0.178144	-2.99924	1.618826
H	4.053206	4.998763	-3.30574
C	0.582171	-5.07848	-4.25331
H	2.688209	-4.59624	-4.49994
H	-1.529215	-5.29352	-3.8043
C	2.922571	-4.29389	2.289949
H	4.689023	-3.97558	1.063942
H	1.005465	-4.41098	3.30553
H	0.583995	-5.85905	-5.0227
H	3.446482	-4.91322	3.027161

TS$_{RE}$CR_conf4

Symbol	X	Y	Z
C	4.336346	2.773336	-0.59478
C	4.519255	3.52256	0.580756
C	3.943901	3.058216	1.777871
C	3.230845	1.850855	1.807868
C	3.034841	1.073941	0.63035
C	3.612938	1.571756	-0.57429
H	4.765434	3.121846	-1.54213
H	5.106938	4.447297	0.567289
H	4.062414	3.633744	2.704355
H	2.812961	1.510259	2.760911
H	3.502713	1.010345	-1.50708
Pd	1.166509	0.130603	0.536868
C	3.43767	-0.91768	1.051998
C	4.925141	-0.59983	0.835665
C	3.095038	-2.13708	0.157328
C	3.20484	-1.21354	2.535183
O	5.642866	-0.20563	1.761887
C	5.522654	-0.85772	-0.52473
C	3.352591	-1.88456	-1.33311
H	2.064018	-2.4883	0.321492
H	3.761392	-2.97125	0.481049
H	3.833176	-2.07705	2.836293
H	3.523605	-0.37324	3.168871
H	2.155606	-1.46197	2.753016
C	4.789068	-1.47279	-1.57032
C	6.871632	-0.49719	-0.7309
H	3.115359	-2.78735	-1.92749
H	2.660728	-1.08624	-1.68168
C	5.426858	-1.70269	-2.80501
H	7.405194	-0.03434	0.105685
C	7.489632	-0.72163	-1.96436
H	4.867586	-2.1914	-3.6131
C	6.76256	-1.3272	-3.00701
H	8.534902	-0.43052	-2.11867
H	7.241594	-1.51592	-3.97494
P	-0.687396	-1.50851	0.889007
C	-2.459218	-0.9208	1.050998
C	-0.361599	-2.33467	2.518329
C	-0.902135	-2.93534	-0.28048
C	-3.067033	-0.22153	-0.00978
C	-3.199337	-1.16247	2.24912
C	-0.342905	-1.53095	3.685629
C	0.022545	-3.68844	2.631226
C	-1.836993	-3.96849	-0.04317
C	-0.134224	-2.96617	-1.46118
C	-2.391236	-0.03934	-1.3407
C	-4.415461	0.276052	0.144294
C	-4.497202	-0.71032	2.401561
H	-2.734445	-1.72764	3.061216
C	0.01139	-2.07511	4.927999
H	-0.610864	-0.4701	3.617888
C	0.395306	-4.22614	3.875141
H	0.033893	-4.32946	1.744455
C	-1.975559	-5.02331	-0.95719
H	-2.457014	-3.94612	0.860312
C	-0.278974	-4.01946	-2.37948
H	0.563256	-2.14624	-1.6648
C	-1.348884	0.884798	-1.55297
C	-2.874161	-0.83963	-2.45026
C	-5.136642	0.030591	1.369483
C	-5.071017	1.032597	-0.87666
H	-5.048103	-0.9197	3.326779
C	0.385108	-3.42673	5.027691
H	0.007833	-1.4375	5.81939
H	0.693467	-5.27896	3.93861
C	-1.193657	-5.05238	-2.12525
H	-2.700438	-5.82171	-0.76078
H	0.319531	-4.02721	-3.29724
P	-0.411884	1.777205	-0.23138
C	-0.754063	0.981567	-2.85683
C	-2.294528	-0.67737	-3.7618
C	-3.915501	-1.80714	-2.30217
C	-6.464462	0.52942	1.518028
H	-4.53783	1.235144	-1.81007
C	-6.361488	1.511562	-0.70033
H	0.674055	-3.84914	5.996397
H	-1.306813	-5.87507	-2.84028
C	-0.010833	3.411298	-1.02505
C	-1.563322	2.395732	1.088238
C	-1.219724	0.243755	-3.92559
H	0.082009	1.671961	-3.00692
C	-2.792106	-1.44222	-4.85586
H	-4.352004	-1.97137	-1.31363
C	-4.376942	-2.54213	-3.38612
H	-6.996499	0.328571	2.455965
C	-7.070019	1.256274	0.504911
H	-6.839149	2.090466	-1.49889
C	-0.818748	4.033531	-2.00455
C	1.123664	4.094953	-0.5408
C	-2.749033	3.110689	0.818705
C	-1.135089	2.261142	2.42538
H	-0.76154	0.357337	-4.91586
H	-2.340553	-1.29405	-5.84461
C	-3.820536	-2.35588	-4.67837
H	-5.178004	-3.2755	-3.23984
H	-8.090271	1.635731	0.630544
C	-0.493604	5.309341	-2.48916
H	-1.700474	3.516138	-2.3984
C	1.441822	5.37501	-1.02379
H	1.761525	3.628134	0.216206
C	-3.500224	3.659528	1.868081
H	-3.087741	3.242077	-0.21405
C	-1.880995	2.821991	3.475403
H	-0.199415	1.723531	2.626902
H	-4.197061	-2.93754	-5.52725
C	0.637622	5.984351	-1.99869
H	-1.128425	5.778508	-3.25006
H	2.330706	5.886845	-0.63874
C	-3.067042	3.518949	3.19794
H	-4.425711	4.202311	1.645574
H	-1.5317	2.717446	4.509197
H	0.890088	6.981009	-2.37867
H	-3.651713	3.957704	4.014586

TS$_{RE}$CR_conf5

Symbol	X	Y	Z
C	-3.957563	-2.50296	-2.74542
C	-4.566173	-3.45307	-1.90644
C	-4.536816	-3.25492	-0.51608
C	-3.918784	-2.11892	0.033265
C	-3.34481	-1.12414	-0.79556
C	-3.378723	-1.34518	-2.20019
H	-3.969548	-2.6387	-3.8342
H	-5.059025	-4.33478	-2.33161
H	-4.979593	-4.00106	0.154514
H	-3.880293	-2.01818	1.123256
H	-2.990079	-0.57547	-2.87863
Pd	-1.663462	0.05671	-0.39851
C	-4.117796	0.825752	-0.33769
C	-3.307618	2.095425	-0.33842
C	-4.807188	0.582202	1.00792
C	-5.120312	0.849761	-1.48884
O	-3.243953	2.829545	-1.35368
C	-2.837723	2.642049	0.994755
C	-3.86328	0.670463	2.211137
H	-5.342815	-0.38299	0.995552
H	-5.586427	1.369146	1.111011
H	-5.803218	1.70971	-1.33265
H	-4.625399	1.01555	-2.45625
H	-5.718712	-0.07466	-1.52985
C	-3.133581	1.998058	2.224309
C	-2.210934	3.907318	1.004898
H	-4.421614	0.533789	3.156581
H	-3.130533	-0.16054	2.152591
C	-2.775176	2.629784	3.430433
H	-2.037564	4.394245	0.040069
C	-1.84993	4.519266	2.212005
H	-3.029497	2.144171	4.381603
C	-2.13098	3.877359	3.431677
H	-1.367526	5.503757	2.205344
H	-1.871662	4.357813	4.382415
P	-0.100151	-1.66221	0.526987
C	1.602375	-1.88193	-0.23708
C	-0.78931	-3.38526	0.491129
C	0.290609	-1.39515	2.316884
C	2.566787	-0.85253	-0.1866
C	1.896526	-3.07019	-0.97552
C	-1.188877	-3.90868	-0.7607
C	-0.984733	-4.17554	1.643724
C	1.197872	-2.22373	3.015908
C	-0.347555	-0.34577	3.006253
C	2.389351	0.356951	0.686435
C	3.804698	-0.99294	-0.9186
C	3.090046	-3.23032	-1.65454
H	1.168316	-3.88459	-0.98948
C	-1.704903	-5.20811	-0.86388
H	-1.114838	-3.28732	-1.65972
C	-1.523841	-5.4688	1.540516
H	-0.713248	-3.78543	2.628881
C	1.434005	-2.02014	4.383458
H	1.72232	-3.02702	2.486487
C	-0.104819	-0.13949	4.373934
H	-1.013651	0.328497	2.456952
C	1.492151	1.396538	0.384695
C	3.181881	0.42904	1.900025
C	4.067921	-2.19815	-1.66549
C	4.793355	0.039365	-0.94688
H	3.292544	-4.16243	-2.19643
C	-1.870046	-5.99583	0.287047
H	-2.002338	-5.59369	-1.84493
H	-1.665771	-6.06728	2.447958
C	0.778618	-0.98111	5.066587
H	2.140519	-2.66782	4.914528
H	-0.601842	0.689724	4.889169
P	0.302645	1.417771	-1.0396
C	1.303537	2.459945	1.328772
C	3.024394	1.548661	2.795677
C	4.126389	-0.58039	2.26261
C	5.292206	-2.3294	-2.3848
H	4.606404	0.965357	-0.39563
C	5.973251	-0.11415	-1.66082
H	-2.281815	-7.00846	0.208392
H	0.969162	-0.82175	6.133963
C	0.371837	3.18422	-1.62335
C	1.110014	0.640334	-2.52661
C	2.05022	2.540352	2.486467
H	0.546013	3.223836	1.127665
C	3.822215	1.630818	3.973794
H	4.249134	-1.45193	1.614823
C	4.893156	-0.47195	3.415018
H	5.47397	-3.25594	-2.94311
C	6.231352	-1.30971	-2.38429
H	6.712812	0.694612	-1.66673
C	1.43693	4.072011	-1.35002
C	-0.660952	3.601118	-2.49153
C	2.29716	1.157006	-3.08835
C	0.439725	-0.40639	-3.19123
H	1.893062	3.370887	3.18518
H	3.686293	2.494465	4.636319
C	4.748163	0.644592	4.279071
H	5.615334	-1.25914	3.659974
H	7.16854	-1.42091	-2.94112
C	1.461257	5.35232	-1.92539
H	2.250368	3.767098	-0.6833
C	-0.629839	4.882263	-3.0659
H	-1.505938	2.936917	-2.69193
C	2.810061	0.623745	-4.27915
H	2.81632	1.989063	-2.60154
C	0.950088	-0.93552	-4.38808
H	-0.491825	-0.79744	-2.76198
H	5.359807	0.720862	5.18511
C	0.427853	5.76109	-2.78469
H	2.29427	6.029612	-1.7031
H	-1.445392	5.193941	-3.72794
C	2.136866	-0.42217	-4.93314
H	3.73639	1.030091	-4.70027
H	0.415461	-1.74675	-4.89554
H	0.447966	6.76157	-3.23202
H	2.534979	-0.83047	-5.86916

TS$_{RE}$CR_conf6

Symbol	X	Y	Z
C	3.510345	3.609908	-2.13908
C	3.463053	4.746466	-1.31304
C	3.076912	4.603887	0.030617
C	2.734161	3.342135	0.546428
C	2.774481	2.191824	-0.27396
C	3.190355	2.342811	-1.62267
H	3.831827	3.701036	-3.18434
H	3.735623	5.731529	-1.70838
H	3.026321	5.483746	0.683682
H	2.421402	3.269029	1.595027
H	3.329521	1.448661	-2.24347
Pd	1.521258	0.540263	-0.17802
C	3.926712	0.549538	0.611146
C	3.911883	-0.77403	-0.10378
C	3.686515	0.504493	2.122061
C	5.229308	1.289134	0.275857
O	4.292096	-0.88551	-1.29577
C	3.729709	-2.00954	0.747889
C	2.730114	-0.61073	2.575131
H	3.338245	1.492789	2.477882
H	4.669322	0.341833	2.616306
H	6.068041	0.717965	0.727546
H	5.399992	1.329359	-0.80847
H	5.243078	2.311803	0.687389
C	3.215868	-1.94451	2.068108
C	4.214027	-3.23779	0.25109
H	2.627871	-0.61884	3.676245
H	1.717818	-0.41391	2.154065
C	3.174811	-3.11311	2.849048
H	4.636836	-3.24829	-0.75761
C	4.167317	-4.39632	1.036743
H	2.781773	-3.05821	3.872365
C	3.641574	-4.3362	2.338887
H	4.543616	-5.34503	0.63766
H	3.609195	-5.23685	2.963248
P	-0.6	1.759407	0.248234
C	-2.23721	1.161208	-0.44472
C	-0.53255	3.511939	-0.36678
C	-0.98647	1.994789	2.047566
C	-2.7361	-0.12979	-0.17184

C	-2.96021	2.013319	-1.3384
C	-0.09848	3.729441	-1.69355
C	-0.88779	4.624853	0.423608
C	-2.28327	2.328913	2.496906
C	0.058819	1.911648	2.990022
C	-2.08608	-1.06478	0.81012
C	-3.94885	-0.57907	-0.82142
C	-4.13631	1.607636	-1.9395
H	-2.57929	3.016465	-1.54297
C	-0.04861	5.026476	-2.22393
H	0.206766	2.8787	-2.31067
C	-0.82467	5.923978	-0.10629
H	-1.21671	4.479076	1.456915
C	-2.52419	2.570897	3.858294
H	-3.10499	2.402467	1.776315
C	-0.18202	2.157603	4.351075
H	1.06557	1.65077	2.647678
C	-0.88922	-1.75103	0.53212
C	-2.76571	-1.30632	2.069149
C	-4.65953	0.305304	-1.71244
C	-4.47264	-1.89771	-0.63732
H	-4.67632	2.290621	-2.60672
C	-0.41113	6.128219	-1.43164
H	0.289504	5.177153	-3.25507
H	-1.10271	6.778118	0.52208
C	-1.47496	2.486197	4.788288
H	-3.53647	2.824071	4.193
H	0.641807	2.086875	5.070283
P	0.265267	-1.41127	-0.8756
C	-0.33861	-2.64169	1.513561
C	-2.21088	-2.23847	3.020857
C	-3.98909	-0.65493	2.418295
C	-5.85405	-0.14219	-2.34986
H	-3.94319	-2.5921	0.021005
C	-5.63124	-2.30947	-1.28083
H	-0.3634	7.142471	-1.84421
H	-1.6662	2.67282	5.851046
C	0.853415	-3.09918	-1.39255
C	-0.66072	-1.0229	-2.44023
C	-0.98206	-2.88677	2.709452
H	0.61449	-3.13948	1.308221
C	-2.89324	-2.48972	4.246951
H	-4.42787	0.061486	1.718989
C	-4.63306	-0.92142	3.618982
H	-6.37972	0.550536	-3.01839
C	-6.33643	-1.4246	-2.1404
H	-6.00643	-3.32684	-1.12223
C	0.101249	-4.2797	-1.20504
C	2.043181	-3.15364	-2.15011
C	-1.71448	-1.82329	-2.92771
C	-0.16481	0.022421	-3.24618
H	-0.54666	-3.58408	3.435368
H	-2.45208	-3.20453	4.952358
C	-4.08625	-1.84896	4.544123
H	-5.57283	-0.409	3.854292
H	-7.2537	-1.75906	-2.63776
C	0.529752	-5.49106	-1.76966
H	-0.82033	-4.25612	-0.61295
C	2.457781	-4.36618	-2.72527
H	2.663762	-2.25668	-2.26717
C	-2.27902	-1.5629	-4.18451
H	-2.09382	-2.65623	-2.32699
C	-0.72135	0.273672	-4.51144
H	0.673103	0.626608	-2.87405
H	-4.60387	-2.05235	5.488221
C	1.704617	-5.53561	-2.5389
H	-0.06206	-6.40071	-1.61392
H	3.3824	-4.39387	-3.31323
C	-1.78275	-0.51552	-4.97984
H	-3.10642	-2.1838	-4.5456
H	-0.321	1.084426	-5.13098
H	2.032414	-6.48069	-2.98703
H	-2.21932	-0.3214	-5.96628

TS$_{RE}$CR_conf7

Symbol	X	Y	Z
C	1.298179	5.150551	0.811083
C	1.539456	5.106896	2.195983
C	1.993104	3.909194	2.770082
C	2.209688	2.773584	1.966664
C	1.936548	2.790355	0.573445
C	1.495604	4.013077	0.01245
H	0.940207	6.076413	0.344425
H	1.388566	5.99862	2.814941
H	2.199212	3.854467	3.846391
H	2.592204	1.862057	2.437869
H	1.277747	4.076822	-1.0572
Pd	1.204529	0.891784	0.020873
P	0.592144	-1.39133	0.579511
C	-1.03457	-1.36545	1.519086
C	1.759079	-2.21994	1.774244
C	0.336102	-2.6672	-0.73357
C	-2.25767	-1.05226	0.886899
C	-1.01591	-1.55117	2.936657
C	2.197495	-1.4837	2.90123
C	2.272897	-3.51933	1.584123
C	-0.2428	-3.92642	-0.45829
C	0.754522	-2.36815	-2.04714
C	-2.41515	-0.96928	-0.60983
C	-3.4591	-0.91004	1.684492
C	-2.15426	-1.41716	3.709057
H	-0.08012	-1.82654	3.427857
C	3.081571	-2.04715	3.833021
H	1.834007	-0.45948	3.050954
C	3.175059	-4.07492	2.506955
H	1.977273	-4.10027	0.706021
C	-0.38616	-4.87811	-1.47872
H	-0.58526	-4.15907	0.556611
C	0.600443	-3.32326	-3.06614
H	1.205001	-1.39219	-2.27024
C	-2.01356	0.148616	-1.36933
C	-3.08602	-2.07283	-1.26517
C	-3.40197	-1.08682	3.115362
C	-4.73264	-0.60982	1.106297
H	-2.10502	-1.57899	4.792971
C	3.573779	-3.34905	3.63883
H	3.394553	-1.46291	4.706104
H	3.566078	-5.08424	2.334862
C	0.035876	-4.5771	-2.78493
H	-0.8396	-5.85057	-1.25642
H	0.923966	-3.08161	-4.08509
P	-1.02897	1.546895	-0.61902
C	-2.26249	0.164312	-2.77809
C	-3.32993	-2.02543	-2.68682
C	-3.53345	-3.23107	-0.55479
C	-4.58889	-0.95558	3.895456
H	-4.80525	-0.47543	0.02399
C	-5.87198	-0.49398	1.889466
H	4.271747	-3.78817	4.360637
H	-0.08237	-5.31928	-3.58282
C	-0.9403	2.774128	-2.01632
C	-2.18409	2.350204	0.580387
C	-2.89918	-0.8833	-3.41552
H	-1.94874	1.031418	-3.36404
C	-4.00252	-3.10798	-3.32687
H	-3.35414	-3.29619	0.521559
C	-4.19018	-4.26664	-1.20385
H	-4.51754	-1.09378	4.98126
C	-5.80508	-0.6669	3.298012
H	-6.83352	-0.26643	1.415387
C	-1.81624	3.872271	-2.13937
C	0.079375	2.59559	-2.98185
C	-3.57259	2.45663	0.342826
C	-1.6399	2.915102	1.753302
H	-3.08752	-0.83735	-4.49522
H	-4.17666	-3.04742	-4.40816
C	-4.43119	-4.20892	-2.60263
H	-4.52517	-5.13858	-0.63049
H	-6.71219	-0.57288	3.905515
C	-1.68737	4.768487	-3.21418
H	-2.59862	4.035531	-1.39152
C	0.192394	3.486429	-4.06164
H	0.803627	1.775408	-2.87072
C	-4.39947	3.125111	1.258665
H	-4.005	2.013103	-0.56126
C	-2.47132	3.583772	2.66601
H	-0.56514	2.83158	1.947594
H	-4.95214	-5.03258	-3.10367
C	-0.68852	4.575349	-4.18112

H	-2.37234	5.620855	-3.29232
H	0.984406	3.333364	-4.80375
C	-3.84942	3.691954	2.420472
H	-5.47596	3.198205	1.06674
H	-2.03621	4.020409	3.571924
H	-0.59037	5.274871	-5.01927
H	-4.49682	4.212281	3.135644
C	3.489521	1.786914	-0.60007
C	3.216925	0.494009	-1.32784
C	4.629035	1.719463	0.416652
C	3.672315	2.924069	-1.60318
O	2.564654	0.477654	-2.41772
C	4.023923	-0.72026	-0.94185
C	4.677192	0.401583	1.200499
H	4.579993	2.5826	1.103729
H	5.579267	1.825648	-0.15184
H	4.546779	2.682791	-2.24195
H	2.804564	3.023035	-2.27069
H	3.861817	3.887072	-1.10137
C	4.785085	-0.76493	0.253573
C	4.127065	-1.78235	-1.86656
H	5.513374	0.402758	1.923744
H	3.741325	0.293103	1.783453
C	5.611479	-1.87239	0.505819
H	3.564767	-1.70591	-2.80145
C	4.948804	-2.88482	-1.6018
H	6.199469	-1.89929	1.431493
C	5.69393	-2.93251	-0.41076
H	5.018975	-3.70169	-2.32938
H	6.348449	-3.78685	-0.20197

TS$_{RE}$CS_conf1

Symbol	X	Y	Z
P	0.598137	-1.63478	-0.57335
C	2.35073	-1.07506	-0.91936
C	0.228923	-2.77016	-1.99881
C	0.850555	-2.82228	0.82772
C	3.000023	-0.18253	-0.04112
C	3.021823	-1.49534	-2.10931
C	0.01241	-2.2004	-3.27614
C	0.048939	-4.16157	-1.84636
C	1.971413	-3.68225	0.877943
C	-0.11629	-2.89114	1.850671
C	2.379456	0.222628	1.268445
C	4.318716	0.307722	-0.36281
C	4.290276	-1.04298	-2.42455
H	2.524057	-2.19943	-2.78164
C	-0.34349	-2.99934	-4.37166
H	0.124176	-1.1185	-3.41015
C	-0.32551	-4.95912	-2.94185
H	0.197925	-4.62616	-0.86725
C	2.11581	-4.59808	1.930465
H	2.730544	-3.63512	0.088823
C	0.033535	-3.80902	2.903676
H	-0.98689	-2.22859	1.821376
C	1.322076	1.14638	1.35319
C	2.904874	-0.37172	2.482143
C	4.970085	-0.12665	-1.57522
C	5.014144	1.234978	0.474594
H	4.787838	-1.39099	-3.33828
C	-0.51816	-4.38485	-4.20702
H	-0.49805	-2.53724	-5.3534
H	-0.4661	-6.03693	-2.80009
C	1.145795	-4.66337	2.94567
H	2.989034	-5.26012	1.959544
H	-0.72749	-3.85287	3.690646
P	0.408905	1.87317	-0.09408
C	0.720309	1.411502	2.628101
C	2.323132	-0.03404	3.758964
C	3.999162	-1.29062	2.474115
C	6.270301	0.366275	-1.89139
H	4.534056	1.578885	1.395521
C	6.277487	1.699964	0.138125
H	-0.81005	-5.00877	-5.05916
H	1.260874	-5.3781	3.768725
C	0.113932	3.637776	0.464314
C	1.595947	2.269264	-1.47124
C	1.207903	0.850413	3.791262

H	-0.1422	2.083782	2.679516
C	2.863561	-0.5937	4.953068
H	4.444801	-1.57753	1.518251
C	4.506794	-1.81708	3.653976
H	6.749205	0.021839	-2.81618
C	6.916463	1.262039	-1.05353
H	6.788297	2.409858	0.798664
C	0.870029	4.294553	1.461907
C	-0.86554	4.382484	-0.22882
C	2.712972	3.117114	-1.31238
C	1.264134	1.81321	-2.76291
H	0.738716	1.082278	4.755192
H	2.406442	-0.31959	5.91175
C	3.94115	-1.46482	4.907436
H	5.35097	-2.51477	3.615711
H	7.915933	1.633268	-1.30647
C	0.636721	5.645482	1.766839
H	1.643482	3.748342	2.011471
C	-1.09184	5.735216	0.068634
H	-1.45056	3.902457	-1.01997
C	3.489849	3.481825	-2.42091
H	2.972118	3.502074	-0.32068
C	2.036927	2.185203	-3.8753
H	0.384125	1.169049	-2.88407
H	4.351358	-1.88628	5.831974
C	-0.34514	6.37081	1.073385
H	1.232451	6.133046	2.547377
H	-1.85599	6.291717	-0.48605
C	3.152397	3.019109	-3.70476
H	4.359955	4.133157	-2.28251
H	1.764043	1.825644	-4.87408
H	-0.52484	7.424982	1.312434
H	3.75643	3.314044	-4.57039
Pd	-1.17297	0.053292	-0.43701
C	-2.81747	-1.14963	0.014611
C	-2.96992	-2.46562	-0.48931
C	-3.22863	-0.89919	1.352566
C	-3.45124	-3.49674	0.328494
H	-2.68286	-2.69817	-1.51896
C	-3.69426	-1.94283	2.173386
H	-3.1913	0.115792	1.759466
C	-3.81383	-3.24593	1.665551
H	-3.53655	-4.51002	-0.0824
H	-3.99128	-1.72138	3.205879
H	-4.20399	-4.05396	2.294649
C	-3.44893	1.868429	-1.30781
C	-3.47942	0.324684	-1.42024
C	-3.81339	2.385238	0.086442
H	-2.47276	2.259195	-1.63541
H	-4.20588	2.259007	-2.02984
C	-4.89512	-0.129	-1.09187
C	-3.06609	-0.14622	-2.81347
C	-5.14351	1.825633	0.544599
H	-3.84552	3.491024	0.099783
H	-3.00194	2.092907	0.788332
O	-5.44626	-1.04865	-1.71214
C	-5.64464	0.628123	-0.02729
H	-3.05149	-1.24191	-2.90006
H	-2.0724	0.248105	-3.08823
H	-3.80238	0.217529	-3.56024
C	-5.9161	2.486483	1.520246
C	-6.90139	0.127888	0.376671
H	-5.54095	3.421669	1.955387
C	-7.15481	1.973127	1.929066
H	-7.25646	-0.79178	-0.09984
C	-7.65168	0.787749	1.353693
H	-7.74035	2.503302	2.689428
H	-8.62193	0.387094	1.668756

TS$_{RE}$CS_conf2

Symbol	X	Y	Z
P	-0.27086	-1.3362	0.943847
C	-1.62087	-0.25805	1.672489
C	0.662565	-1.89707	2.445538
C	-1.27064	-2.83933	0.505076
C	-2.51493	0.404203	0.811323
C	-1.77382	-0.11403	3.086394
C	1.287737	-0.9344	3.275641

C	0.870434	-3.26526	2.730135
C	-2.16956	-3.41204	1.434821
C	-1.16234	-3.39982	-0.78148
C	-2.47584	0.20611	-0.67799
C	-3.55157	1.245927	1.362725
C	-2.77075	0.675543	3.629407
H	-1.09618	-0.64854	3.756996
C	2.056005	-1.32962	4.3804
H	1.166779	0.132834	3.058156
C	1.661328	-3.65733	3.824048
H	0.405771	-4.02837	2.09852
C	-2.93499	-4.53273	1.083117
H	-2.27017	-2.97864	2.436425
C	-1.93552	-4.51992	-1.13194
H	-0.47285	-2.9618	-1.50902
C	-1.4997	0.796821	-1.4997
C	-3.52135	-0.60277	-1.27504
C	-3.67563	1.386044	2.793132
C	-4.46566	1.973347	0.53802
H	-2.8734	0.760701	4.718278
C	2.248041	-2.69409	4.658744
H	2.513078	-0.56638	5.020445
H	1.809014	-4.72439	4.026785
C	-2.81883	-5.08893	-0.20321
H	-3.62667	-4.96992	1.812696
H	-1.83942	-4.94568	-2.1366
P	0.057283	1.647722	-0.92799
C	-1.54287	0.566185	-2.91534
C	-3.56244	-0.78234	-2.70596
C	-4.52968	-1.24855	-0.49517
C	-4.6953	2.223808	3.333586
H	-4.38607	1.882335	-0.54919
C	-5.44347	2.786905	1.092908
H	2.854269	-3.00239	5.517898
H	-3.42039	-5.96278	-0.4789
C	0.212441	2.972961	-2.23481
C	-0.27431	2.703542	0.564957
C	-2.54401	-0.1829	-3.49987
H	-0.76824	1.009436	-3.54853
C	-4.60638	-1.55576	-3.2921
H	-4.50364	-1.15235	0.593261
C	-5.53011	-2.00207	-1.09356
H	-4.77428	2.314365	4.423854
C	-5.56511	2.913526	2.503299
H	-6.13018	3.334265	0.43731
C	-0.90827	3.703534	-2.69652
C	1.488868	3.28338	-2.74509
C	-1.34595	3.615254	0.675917
C	0.696825	2.667144	1.587653
H	-2.56203	-0.32597	-4.58728
H	-4.62089	-1.67202	-4.38275
C	-5.57867	-2.15269	-2.50448
H	-6.28776	-2.4881	-0.46874
H	-6.3444	3.555134	2.929603
C	-0.75022	4.736015	-3.63136
H	-1.91412	3.449414	-2.34494
C	1.640694	4.320968	-3.68345
H	2.365498	2.697928	-2.43888
C	-1.45012	4.458636	1.791125
H	-2.10987	3.667395	-0.1051
C	0.59735	3.52049	2.699548
H	1.546778	1.981	1.496823
H	-6.3768	-2.74551	-2.96528
C	0.528551	5.052645	-4.12329
H	-1.62866	5.291498	-3.98032
H	2.639394	4.545848	-4.07521
C	-0.47729	4.415541	2.804813
H	-2.29546	5.151569	1.867653
H	1.370667	3.486934	3.474887
H	0.650877	5.859715	-4.85501
H	-0.55734	5.082497	3.671009
Pd	1.442388	-0.39057	-0.62795
C	2.187436	-2.19153	-1.34704
C	2.376984	-3.37371	-0.59322
C	1.76776	-2.32667	-2.69792
C	2.150113	-4.63848	-1.16421
H	2.675872	-3.31503	0.459238
C	1.521704	-3.59111	-3.25866
H	1.650771	-1.43219	-3.32136
C	1.723917	-4.75665	-2.49776
H	2.297453	-5.53801	-0.55364
H	1.192835	-3.66352	-4.30277
H	1.560121	-5.74492	-2.94203
C	4.67904	-1.42122	-0.05969
C	3.90506	-0.83317	-1.23947
C	4.19956	-0.91229	1.302675
H	4.660523	-2.52459	-0.09784
H	5.74252	-1.12441	-0.19788
C	3.795763	0.663269	-1.17813
C	4.478274	-1.27015	-2.58322
C	4.222925	0.599482	1.352539
H	4.817628	-1.33117	2.118695
H	3.164321	-1.26794	1.476829
O	3.802028	1.358492	-2.22535
C	3.993412	1.339167	0.162902
H	5.497926	-0.84333	-2.67751
H	3.894185	-0.87336	-3.42559
H	4.546391	-2.3672	-2.66123
C	4.491059	1.293697	2.547398
C	4.070365	2.748532	0.195964
H	4.685305	0.719775	3.462411
C	4.539599	2.696803	2.575766
H	3.930124	3.290636	-0.74398
C	4.331424	3.427093	1.391863
H	4.762336	3.21771	3.514574
H	4.381738	4.521712	1.402214

TS$_{RE}$CS_conf3

Symbol	X	Y	Z
P	0.152933	1.361947	0.937559
C	1.535514	0.35152	1.704201
C	-0.81003	1.943833	2.412618
C	1.088366	2.888156	0.446362
C	2.484013	-0.27081	0.870039
C	1.645197	0.199981	3.121092
C	-1.43106	0.991622	3.257899
C	-1.04481	3.314547	2.662034
C	1.970857	3.523853	1.350261
C	0.93372	3.413622	-0.85002
C	2.491193	-0.04553	-0.61584
C	3.528225	-1.08141	1.450497
C	2.648252	-0.56218	3.692247
H	0.929487	0.707397	3.772852
C	-2.21667	1.400006	4.345608
H	-1.29709	-0.07867	3.064022
C	-1.85397	3.719406	3.737539
H	-0.5871	4.070405	2.017173
C	2.67391	4.673326	0.963181
H	2.105848	3.117125	2.35902
C	1.644893	4.562606	-1.23601
H	0.257556	2.923253	-1.55637
C	1.557267	-0.64437	-1.47872
C	3.52105	0.817804	-1.16238
C	3.604994	-1.23421	2.882861
C	4.498179	-1.76308	0.651599
H	2.716316	-0.65309	4.783366
C	-2.43305	2.767221	4.590036
H	-2.66901	0.644455	4.997977
H	-2.02192	4.788471	3.911985
C	2.511018	5.195228	-0.33243
H	3.353804	5.160119	1.672086
H	1.514004	4.961121	-2.24791
P	0.061988	-1.63344	-0.96651
C	1.604	-0.3425	-2.88077
C	3.575408	1.059431	-2.58374
C	4.504644	1.452883	-0.34324
C	4.634287	-2.04157	3.450897
H	4.454359	-1.65994	-0.43651
C	5.485853	-2.54544	1.232785
H	-3.05317	3.08583	5.435382
H	3.063848	6.091913	-0.63541
C	0.024424	-2.91444	-2.32389
C	0.476373	-2.7179	0.485687
C	2.582306	0.470273	-3.41663
H	0.853332	-0.78415	-3.54326
C	4.606844	1.881925	-3.12304
H	4.470902	1.306792	0.73932
C	5.496648	2.250823	-0.89672
H	4.67736	-2.14293	4.542226

C	5.559616	-2.68661	2.64518
H	6.217302	-3.05715	0.597051
C	1.205156	-3.47727	-2.86494
C	-1.22582	-3.37617	-2.78402
C	1.554139	-3.62895	0.503351
C	-0.42253	-2.70377	1.572118
H	2.604995	0.668345	-4.49528
H	4.630985	2.045578	-4.20742
C	5.557226	2.464537	-2.29884
H	6.238083	2.723238	-0.24258
H	6.346688	-3.30399	3.0925
C	1.134312	-4.49183	-3.83014
H	2.186334	-3.10428	-2.55104
C	-1.28959	-4.39459	-3.75244
H	-2.15357	-2.92106	-2.41435
C	1.738864	-4.49084	1.593242
H	2.253478	-3.66967	-0.33666
C	-0.2425	-3.57495	2.659592
H	-1.28008	-2.02107	1.548212
H	6.3476	3.093402	-2.72386
C	-0.11582	-4.95842	-4.2728
H	2.058413	-4.91587	-4.24032
H	-2.26931	-4.73754	-4.10445
C	0.839895	-4.46701	2.673548
H	2.587326	-5.18418	1.597789
H	-0.9593	-3.55908	3.487988
H	-0.17	-5.75102	-5.02817
H	0.981736	-5.1484	3.520382
Pd	-1.48539	0.279532	-0.63155
C	-2.35562	2.008315	-1.39186
C	-2.62978	3.187771	-0.65996
C	-1.93662	2.149067	-2.742
C	-2.48096	4.455058	-1.25048
H	-2.93565	3.127458	0.390174
C	-1.77056	3.417452	-3.323
H	-1.7557	1.254411	-3.34932
C	-2.05344	4.579478	-2.58311
H	-2.6923	5.352496	-0.65586
H	-1.44042	3.493562	-4.36645
H	-1.95112	5.569039	-3.04262
C	-4.79169	1.097273	-0.11939
C	-3.95371	0.518037	-1.26027
C	-4.28615	0.705664	1.272158
H	-4.86834	2.194584	-0.21629
H	-5.82314	0.700623	-0.24954
C	-3.73245	-0.96219	-1.12001
C	-4.53899	0.840911	-2.63068
C	-4.1674	-0.79673	1.404352
H	-4.95272	1.106227	2.05878
H	-3.29164	1.165464	1.438642
O	-3.69672	-1.71204	-2.12787
C	-3.86696	-1.57727	0.257438
H	-5.51851	0.32768	-2.71705
H	-3.91152	0.453639	-3.44609
H	-4.69476	1.923699	-2.76334
C	-4.37162	-1.44556	2.636822
C	-3.80819	-2.98337	0.369736
H	-4.62381	-0.84262	3.518427
C	-4.28557	-2.84289	2.744185
H	-3.61364	-3.56056	-0.53927
C	-4.00573	-3.6155	1.602598
H	-4.45979	-3.32902	3.711475
H	-3.95046	-4.70753	1.674722

TS$_{RE}$CS_conf4

Symbol	X	Y	Z
P	0.763603	-1.73491	-0.37414
C	2.331344	-1.04124	-1.13454
C	0.276297	-3.08033	-1.55892
C	1.441013	-2.67652	1.072595
C	3.017142	0.022254	-0.51362
C	2.822386	-1.56335	-2.37117
C	-0.1599	-2.71019	-2.85565
C	0.178112	-4.43744	-1.18224
C	2.555544	-3.53653	0.94962
C	0.822057	-2.53191	2.329476
C	2.642998	0.536915	0.849764
C	4.17605	0.596783	-1.15935
C	3.943779	-1.03872	-2.9871
H	2.306771	-2.40655	-2.83807
C	-0.64243	-3.67162	-3.75509
H	-0.10858	-1.6594	-3.16489
C	-0.32446	-5.39582	-2.07905
H	0.496717	-4.75088	-0.18341
C	3.023806	-4.25164	2.061663
H	3.055652	-3.64632	-0.01951
C	1.29471	-3.24504	3.44338
H	-0.02417	-1.84173	2.425475
C	1.52714	1.364372	1.082046
C	3.504711	0.174456	1.958656
C	4.641436	0.060517	-2.41522
C	4.884071	1.708715	-0.6052
H	4.305917	-1.46804	-3.92942
C	-0.73023	-5.0203	-3.36838
H	-0.96014	-3.36449	-4.75816
H	-0.39385	-6.44329	-1.76392
C	2.392062	-4.1092	3.309517
H	3.8873	-4.91834	1.955579
H	0.805752	-3.1211	4.416072
P	0.171588	1.710195	-0.12882
C	1.22841	1.782815	2.423029
C	3.214962	0.663158	3.285341
C	4.652239	-0.66255	1.797876
C	5.781808	0.636454	-3.04922
H	4.542625	2.133773	0.343066
C	5.985999	2.253304	-1.24931
H	-1.11801	-5.77029	-4.06664
H	2.761962	-4.66641	4.177651
C	-0.29586	3.477699	0.225582
C	0.857896	1.974225	-1.83497
C	2.050918	1.460093	3.482627
H	0.324703	2.371958	2.607287
C	4.081161	0.331843	4.367115
H	4.881129	-1.06714	0.808585
C	5.477287	-0.96997	2.871315
H	6.12114	0.210721	-4.00147
C	6.445297	1.712532	-2.48078
H	6.508322	3.10694	-0.80265
C	0.560788	4.429876	0.821944
C	-1.56484	3.897907	-0.22724
C	1.898664	2.88266	-2.12093
C	0.190151	1.34202	-2.90394
H	1.80916	1.809465	4.493885
H	3.842109	0.720467	5.364688
C	5.198563	-0.465	4.168265
H	6.351417	-1.61209	2.714442
H	7.319801	2.147845	-2.97722
C	0.152371	5.765157	0.964833
H	1.550164	4.127538	1.181752
C	-1.96726	5.235969	-0.09419
H	-2.23886	3.168819	-0.68841
C	2.274839	3.135331	-3.44806
H	2.414417	3.399163	-1.3048
C	0.558962	1.603812	-4.23402
H	-0.6315	0.650635	-2.67575
H	5.85923	-0.71155	5.006968
C	-1.11133	6.172707	0.506028
H	0.828624	6.491013	1.431409
H	-2.95564	5.54412	-0.45379
C	1.604478	2.499185	-4.50731
H	3.091719	3.835411	-3.65589
H	0.026158	1.111206	-5.05574
H	-1.42696	7.216262	0.617167
H	1.894662	2.70677	-5.54361
Pd	-1.14842	-0.26753	0.192574
C	-2.78598	0.28372	1.360992
C	-2.48752	0.018244	2.722857
C	-3.59034	1.419068	1.075797
C	-2.89463	0.895872	3.741565
H	-1.9283	-0.88195	2.998419
C	-4.01191	2.280327	2.098999
H	-3.88763	1.637634	0.045982
C	-3.66305	2.030777	3.437996
H	-2.62593	0.673091	4.781631
H	-4.61775	3.1581	1.843216
H	-4.00346	2.701542	4.234766
C	-3.30436	-1.743	-1.15701
C	-3.29526	-1.56163	0.379344
C	-3.8962	-0.54112	-1.90554

H	-2.30142	-1.98278	-1.54601
H	-3.93911	-2.6342	-1.37347
C	-4.72237	-1.28601	0.849353
C	-2.76061	-2.7954	1.112076
C	-5.28909	-0.21882	-1.41129
H	-3.91695	-0.73464	-2.99516
H	-3.2225	0.330096	-1.75391
O	-5.13591	-1.70577	1.938209
C	-5.66235	-0.57194	-0.08968
H	-3.50005	-3.61939	1.033408
H	-2.6318	-2.61386	2.189962
H	-1.8069	-3.14236	0.684294
C	-6.23559	0.4184	-2.23771
C	-6.96636	-0.29049	0.371101
H	-5.95651	0.681367	-3.26617
C	-7.52457	0.707551	-1.76752
H	-7.21623	-0.59007	1.394203
C	-7.8927	0.351162	-0.45606
H	-8.2473	1.20224	-2.42709
H	-8.90064	0.57325	-0.08734

TS$_{RE}$CS_conf5

Symbol	X	Y	Z
P	-0.989	1.776317	-0.55257
C	-2.49238	0.79005	-1.0594
C	-0.86087	3.079001	-1.87386
C	-1.49845	2.746658	0.933648
C	-2.98102	-0.25842	-0.25247
C	-3.07538	1.013672	-2.34558
C	-0.36839	2.690705	-3.14344
C	-1.13948	4.443639	-1.65004
C	-2.78651	3.305345	1.086289
C	-0.51895	2.969158	1.925918
C	-2.52473	-0.47625	1.167833
C	-4.0547	-1.09513	-0.7451
C	-4.09663	0.217558	-2.83007
H	-2.71158	1.842358	-2.95835
C	-0.18997	3.633538	-4.16616
H	-0.12768	1.635564	-3.32465
C	-0.94057	5.390261	-2.66993
H	-1.51128	4.771649	-0.67437
C	-3.08865	4.083618	2.213857
H	-3.55126	3.130334	0.32109
C	-0.82862	3.751705	3.051033
H	0.479332	2.527123	1.811601
C	-1.31944	-1.12546	1.516239
C	-3.43544	-0.05494	2.213775
C	-4.60932	-0.85931	-2.05658
C	-4.61093	-2.16219	0.027702
H	-4.5309	0.419092	-3.8171
C	-0.47167	4.990688	-3.93047
H	0.181567	3.309368	-5.14515
H	-1.1585	6.446596	-2.47422
C	-2.1091	4.30891	3.196392
H	-4.09186	4.50962	2.328649
H	-0.06426	3.920778	3.818038
P	-2.1E-05	-1.5556	0.264929
C	-1.02197	-1.34413	2.89961
C	-3.10719	-0.29432	3.598316
C	-4.67687	0.599033	1.933791
C	-5.66655	-1.68763	-2.53745
H	-4.21489	-2.35267	1.028745
C	-5.64259	-2.94845	-0.46455
H	-0.3196	5.731227	-4.72373
H	-2.34822	4.915348	4.07768
C	1.186446	-2.60058	1.241
C	-0.76838	-2.76615	-0.89774
C	-1.88237	-0.94757	3.904218
H	-0.09184	-1.84873	3.170359
C	-4.01123	0.111468	4.623498
H	-4.95153	0.797177	0.894533
C	-5.53869	0.980283	2.95241
H	-6.07012	-1.49023	-3.53814
C	-6.17777	-2.71416	-1.75969
H	-6.04961	-3.75703	0.153268
C	2.142309	-1.9294	2.040613
C	1.21551	-4.00927	1.182653
C	-1.70662	-3.73626	-0.48231
C	-0.34216	-2.74876	-2.2433
H	-1.62913	-1.14304	4.953476
H	-3.73702	-0.08381	5.667429
C	-5.20898	0.735071	4.311897
H	-6.48409	1.475755	2.703507
H	-6.99279	-3.34155	-2.13763
C	3.083257	-2.65571	2.786587
H	2.151997	-0.8318	2.065791
C	2.167675	-4.73215	1.921272
H	0.498062	-4.5469	0.554793
C	-2.20981	-4.67253	-1.39876
H	-2.04421	-3.75529	0.55979
C	-0.84496	-3.69045	-3.15523
H	0.385067	-1.99579	-2.56805
H	-5.89855	1.039514	5.107215
C	3.09954	-4.06003	2.727444
H	3.819908	-2.11695	3.392728
H	2.179584	-5.82676	1.861574
C	-1.7785	-4.65194	-2.73562
H	-2.9446	-5.41544	-1.06879
H	-0.50565	-3.66911	-4.19697
H	3.844039	-4.62668	3.29837
H	-2.17355	-5.38356	-3.44978
Pd	1.021657	0.384285	-0.61014
C	2.680596	-0.3888	-1.59787
C	3.361778	-1.57942	-1.24077
C	2.529039	-0.10145	-2.98036
C	3.78601	-2.48603	-2.22665
H	3.532267	-1.81897	-0.186
C	2.94807	-1.01749	-3.96444
H	2.079196	0.848831	-3.29561
C	3.574069	-2.21819	-3.59257
H	4.284001	-3.41472	-1.92091
H	2.794343	-0.77991	-5.02486
H	3.911067	-2.92865	-4.35589
C	4.784049	1.564164	-1.4014
C	3.435698	1.581163	-0.67163
C	5.80662	0.528196	-0.9164
H	4.617938	1.448721	-2.48836
H	5.223568	2.579819	-1.26814
C	3.504547	1.347198	0.792064
C	2.653737	2.833335	-1.08682
C	5.909397	0.488762	0.59092
H	6.798009	0.745439	-1.35751
H	5.517088	-0.47389	-1.28386
O	2.53496	1.567463	1.563833
C	4.793022	0.852192	1.387076
H	3.342226	3.709378	-1.08101
H	1.821421	3.049425	-0.40039
H	2.252585	2.765118	-2.11326
C	7.091549	0.067259	1.230754
C	4.889597	0.790102	2.796983
H	7.957681	-0.21145	0.617257
C	7.17786	0.006691	2.628716
H	4.012157	1.091497	3.378398
C	6.070599	0.372221	3.417912
H	8.11133	-0.31617	3.104499
H	6.136733	0.337438	4.511525

TS$_{RE}$CS_conf6

Symbol	X	Y	Z
P	-1.0995	1.685398	0.340794
C	-2.53555	0.802842	-0.47492
C	-1.08711	3.322993	-0.53895
C	-1.84533	2.141696	1.9834
C	-2.7781	-0.5599	-0.22051
C	-3.41454	1.52207	-1.34595
C	-0.87594	3.357233	-1.93903
C	-1.16799	4.548307	0.158434
C	-3.17818	2.599011	2.100216
C	-1.04171	2.049269	3.138387
C	-1.99358	-1.39782	0.758864
C	-3.90654	-1.2119	-0.85788
C	-4.4941	0.91414	-1.95715
H	-3.23182	2.584488	-1.52705
C	-0.78478	4.579249	-2.62068
H	-0.78984	2.419578	-2.49986
C	-1.05054	5.770984	-0.52461

H	-1.33008	4.548943	1.240795
C	-3.69397	2.965091	3.351934
H	-3.81373	2.669256	1.209942
C	-1.56517	2.420019	4.390185
H	-0.01126	1.682886	3.054542
C	-0.76417	-2.02472	0.451744
C	-2.64135	-1.68704	2.022323
C	-4.76659	-0.46497	-1.743
C	-4.20525	-2.59705	-0.65845
H	-5.1563	1.493698	-2.61223
C	-0.8668	5.793017	-1.91565
H	-0.63953	4.581996	-3.70717
H	-1.11421	6.710116	0.037245
C	-2.88656	2.877564	4.500166
H	-4.72854	3.319324	3.431501
H	-0.93287	2.342612	5.282068
P	0.407297	-1.42496	-0.86274
C	-0.21922	-2.97877	1.371668
C	-2.06017	-2.64366	2.93357
C	-3.85888	-1.04986	2.417509
C	-5.8678	-1.1132	-2.37673
H	-3.57012	-3.18732	0.007812
C	-5.28005	-3.20313	-1.29428
H	-0.78596	6.747504	-2.44796
H	-3.29181	3.162882	5.477997
C	1.541419	-2.87287	-1.1765
C	-0.47903	-1.30393	-2.47964
C	-0.84957	-3.29107	2.559742
H	0.722156	-3.4763	1.121239
C	-2.70883	-2.92727	4.170809
H	-4.30902	-0.30692	1.753958
C	-4.46301	-1.34015	3.63243
H	-6.50842	-0.52175	-3.04236
C	-6.12417	-2.45798	-2.16081
H	-5.48159	-4.26637	-1.12123
C	2.746469	-2.92603	-0.44209
C	1.282821	-3.88505	-2.12652
C	-1.5187	-2.17242	-2.87555
C	-0.01196	-0.33185	-3.39228
H	-0.41654	-4.04148	3.232655
H	-2.24867	-3.65964	4.845508
C	-3.89034	-2.29085	4.518431
H	-5.3892	-0.8257	3.912028
H	-6.97303	-2.94615	-2.65267
C	3.659041	-3.97673	-0.63073
H	2.965369	-2.13721	0.286518
C	2.200803	-4.92902	-2.32235
H	0.368174	-3.8538	-2.72716
C	-2.08422	-2.06693	-4.15635
H	-1.89892	-2.92518	-2.17717
C	-0.57055	-0.23851	-4.67745
H	0.795967	0.345417	-3.09141
H	-4.37864	-2.51366	5.473757
C	3.388814	-4.98047	-1.57415
H	4.582605	-3.99692	-0.04196
H	1.985398	-5.70541	-3.06582
C	-1.6088	-1.10306	-5.0608
H	-2.90185	-2.7377	-4.44297
H	-0.19287	0.514617	-5.37845
H	4.103512	-5.79632	-1.73254
H	-2.04931	-1.02479	-6.06139
Pd	1.257443	0.601494	0.088543
C	2.72131	1.483871	-1.11347
C	3.506135	0.54615	-1.83584
C	2.427155	2.718629	-1.73551
C	3.953513	0.831083	-3.13699
H	3.716298	-0.43744	-1.40459
C	2.880401	2.999549	-3.03816
H	1.808115	3.460365	-1.22315
C	3.651111	2.063116	-3.74454
H	4.531099	0.074312	-3.68207
H	2.61883	3.96076	-3.49746
H	4.009142	2.285808	-4.75612
C	4.665181	2.08381	0.768238
C	3.150946	1.877316	0.973736
C	5.525062	0.826456	0.572568
H	4.834729	2.789332	-0.06485
H	5.012673	2.600694	1.69041
C	2.846217	0.751734	1.924877
C	2.503516	3.198033	1.383175
C	5.125273	-0.32604	1.469398
H	6.587459	1.082311	0.749831
H	5.469516	0.503724	-0.48275
O	1.870714	0.795971	2.726495
C	3.842063	-0.36961	2.070409
H	3.024587	3.59231	2.280708
H	1.441283	3.084673	1.639726
H	2.607397	3.958651	0.591916
C	6.026808	-1.38035	1.721568
C	3.499587	-1.44912	2.917548
H	7.026419	-1.34385	1.269864
C	5.674077	-2.45678	2.5478
H	2.509708	-1.43256	3.384103
C	4.401326	-2.49147	3.149839
H	6.396121	-3.25993	2.736756
H	4.123218	-3.32492	3.80484

$TS_{RE}CS$_conf7

Symbol	X	Y	Z
P	0.749217	1.355789	0.89358
C	1.898127	0.0112	1.501339
C	0.181267	2.149059	2.470585
C	1.978027	2.585493	0.24215
C	2.479188	-0.87798	0.579487
C	2.218443	-0.10639	2.890417
C	-0.5317	1.375328	3.419154
C	0.340885	3.530992	2.714675
C	3.133127	2.927657	0.98302
C	1.766878	3.179098	-1.01684
C	2.288217	-0.76432	-0.9103
C	3.378781	-1.90964	1.053118
C	3.077069	-1.08408	3.355961
H	1.785759	0.601454	3.601655
C	-1.03714	1.960533	4.589375
H	-0.67628	0.302131	3.246631
C	-0.1838	4.117605	3.879176
H	0.88227	4.150863	1.993842
C	4.053405	3.854198	0.472535
H	3.311219	2.467768	1.961845
C	2.693674	4.104622	-1.52626
H	0.874851	2.921789	-1.59567
C	1.133238	-1.21386	-1.58595
C	3.416837	-0.27028	-1.67454
C	3.671743	-2.01622	2.461713
C	3.994844	-2.85489	0.173791
H	3.313777	-1.14581	4.425353
C	-0.86804	3.336289	4.822849
H	-1.57015	1.33936	5.318286
H	-0.04791	5.191893	4.049284
C	3.834741	4.444435	-0.78494
H	4.944307	4.114134	1.056119
H	2.515248	4.560025	-2.5065
P	-0.50946	-1.5345	-0.74068
C	1.129781	-1.22647	-3.01695
C	3.376457	-0.28686	-3.11664
C	4.593878	0.248768	-1.05008
C	4.550972	-3.0383	2.926785
H	3.789625	-2.79526	-0.8986
C	4.84307	-3.84194	0.655755
H	-1.2688	3.794503	5.733934
H	4.556154	5.166647	-1.18423
C	-1.41769	-2.59374	-1.96985
C	-0.20571	-2.72734	0.637434
C	2.212771	-0.79304	-3.75717
H	0.249892	-1.60585	-3.5422
C	4.495728	0.186458	-3.86283
H	4.640675	0.295014	0.040876
C	5.663896	0.711455	-1.80231
H	4.759747	-3.0979	4.001949
C	5.128656	-3.93746	2.044422
H	5.299772	-4.55186	-0.0431
C	-2.07509	-1.94582	-3.03999
C	-1.49933	-4.00067	-1.87471
C	0.744998	-3.76825	0.564823
C	-1.03807	-2.63628	1.77267
H	2.181996	-0.83614	-4.85288
H	4.442707	0.157604	-4.95805
C	5.622925	0.674921	-3.22174
H	6.548285	1.11224	-1.29457

H	5.803656	-4.71822	2.412486
C	-2.75909	-2.69048	-4.01447
H	-2.07511	-0.8538	-3.09781
C	-2.19348	-4.7405	-2.84404
H	-1.024	-4.52344	-1.03955
C	0.868602	-4.69207	1.614053
H	1.394666	-3.8531	-0.31253
C	-0.91856	-3.56717	2.817134
H	-1.79508	-1.84574	1.827792
H	6.47735	1.037732	-3.80405
C	-2.81836	-4.08893	-3.92085
H	-3.26871	-2.16722	-4.83068
H	-2.24481	-5.83199	-2.75566
C	0.036365	-4.59346	2.74182
H	1.621303	-5.48568	1.550662
H	-1.57943	-3.48942	3.687676
H	-3.361	-4.66945	-4.67585
H	0.132797	-5.31709	3.559409
Pd	-1.29018	0.754676	-0.35545
C	-1.82313	2.680508	-0.93347
C	-1.71108	3.84896	-0.1455
C	-1.60759	2.79813	-2.33326
C	-1.37526	5.083004	-0.73016
H	-1.85942	3.799359	0.939167
C	-1.25228	4.028344	-2.9111
H	-1.74628	1.92156	-2.97805
C	-1.14114	5.180976	-2.11252
H	-1.28646	5.973125	-0.09471
H	-1.08534	4.088991	-3.99381
H	-0.88921	6.146887	-2.56472
C	-4.15769	2.004393	0.82573
C	-3.734	1.622409	-0.59025
C	-3.68279	1.024205	1.901927
H	-3.83168	3.033102	1.061026
H	-5.27003	2.029665	0.831834
C	-3.8873	0.167505	-0.92051
C	-4.43612	2.47907	-1.64266
C	-4.10698	-0.38848	1.57594
H	-4.05114	1.324924	2.90053
H	-2.57311	1.071709	1.942565
O	-4.06848	-0.21508	-2.10206
C	-4.12625	-0.80117	0.218172
H	-5.52375	2.264329	-1.58532
H	-4.11285	2.216889	-2.65923
H	-4.28151	3.556374	-1.46807
C	-4.47078	-1.3086	2.576409
C	-4.50085	-2.12275	-0.10391
H	-4.47913	-0.98219	3.62463
C	-4.83253	-2.62607	2.248556
H	-4.52502	-2.39982	-1.16231
C	-4.84092	-3.03473	0.903229
H	-5.11932	-3.32768	3.040782
H	-5.12391	-4.06063	0.641187

TS$_{RE}$ηR

Symbol	X	Y	Z
P	1.957484	-1.82477	-0.24258
C	2.629268	-0.33861	-1.15987
C	1.688143	-3.04375	-1.62683
C	3.505172	-2.50511	0.540468
C	2.648117	0.914115	-0.51837
C	3.121181	-0.44998	-2.49687
C	2.650725	-3.97656	-2.07419
C	0.40582	-3.05565	-2.2224
C	4.813205	-2.29049	0.052224
C	3.338421	-3.30295	1.693637
C	2.14389	1.069468	0.892511
C	3.225283	2.054206	-1.18575
C	3.644741	0.642095	-3.16613
H	3.078277	-1.4228	-2.99742
C	2.343601	-4.88226	-3.10315
H	3.643743	-4.00117	-1.61217
C	0.104524	-3.95654	-3.25629
H	-0.38129	-2.37858	-1.8679
C	5.921039	-2.86667	0.694865
H	4.965378	-1.66945	-0.83801
C	4.444637	-3.88378	2.333387
H	2.330213	-3.46452	2.09416
C	0.79403	1.313534	1.20064
C	3.109874	0.976899	1.968132
C	3.72262	1.914928	-2.5337
C	3.32972	3.333532	-0.55841
H	4.017369	0.535861	-4.19261
C	1.072539	-4.87155	-3.70109
H	3.102105	-5.60092	-3.43587
H	-0.89831	-3.94679	-3.69745
C	5.739494	-3.66599	1.83561
H	6.929513	-2.69135	0.301691
H	4.295424	-4.50063	3.227115
P	-0.64325	1.449254	0.028478
C	0.376777	1.341425	2.57283
C	2.672886	1.067889	3.340594
C	4.507826	0.815231	1.721649
C	4.284231	3.04798	-3.19269
H	2.96693	3.448963	0.46783
C	3.89115	4.414896	-1.22265
H	0.834937	-5.58205	-4.50142
H	6.604965	-4.11355	2.337591
C	-1.54423	2.942887	0.667549
C	-0.12251	2.052217	-1.64855
C	1.283424	1.224908	3.606585
H	-0.68766	1.452344	2.804986
C	3.633104	0.99431	4.391412
H	4.857898	0.742688	0.688483
C	5.420237	0.755578	2.765648
H	4.653799	2.926403	-4.21826
C	4.370716	4.27518	-2.55316
H	3.96786	5.384135	-0.71675
C	-0.91839	4.00322	1.357909
C	-2.92453	3.033551	0.385063
C	0.20362	3.400583	-1.91101
C	-0.20182	1.144034	-2.7253
H	0.936463	1.251823	4.646376
H	3.279906	1.063037	5.427678
C	4.983352	0.8445	4.113792
H	6.486699	0.63343	2.546513
H	4.811038	5.136308	-3.06844
C	-1.65943	5.127991	1.756363
H	0.148413	3.944147	1.598956
C	-3.66113	4.162671	0.774814
H	-3.4153	2.206289	-0.14318
C	0.462264	3.823943	-3.22293
H	0.241054	4.126455	-1.09276
C	0.061577	1.56937	-4.03688
H	-0.48907	0.103492	-2.53135
H	5.712787	0.792844	4.929945
C	-3.03064	5.211512	1.464539
H	-1.16141	5.9413	2.296849
H	-4.73152	4.218084	0.545925
C	0.392196	2.910327	-4.28775
H	0.715166	4.872868	-3.41333
H	-0.00386	0.851537	-4.86215
H	-3.60669	6.089818	1.777421
H	0.58667	3.246485	-5.31275
Pd	-2.00747	-0.41656	-0.00164
C	-2.54014	-1.02322	1.952156
C	-1.56438	-1.65326	2.759841
C	-3.43824	-0.11853	2.575442
C	-1.51078	-1.41457	4.143761
H	-0.81777	-2.30926	2.301595
C	-3.37038	0.129156	3.957203
H	-4.16446	0.431297	1.968685
C	-2.4183	-0.53198	4.752843
H	-0.74105	-1.91397	4.744617
H	-4.06489	0.847133	4.410507
H	-2.37818	-0.35245	5.833284
C	-4.60968	-2.78469	1.261782
C	-3.31168	-2.39451	0.537162
C	-5.78706	-1.79969	1.164696
H	-4.39197	-3.00662	2.322035
H	-4.91593	-3.7475	0.796592
C	-3.54303	-1.81414	-0.82553
C	-2.31416	-3.54805	0.539848
C	-5.91604	-1.11607	-0.18278
H	-6.72695	-2.33566	1.398819
H	-5.68484	-1.03041	1.95126
O	-2.70855	-1.98192	-1.78191
C	-4.83794	-1.1037	-1.10311
H	-2.69864	-4.35455	-0.11448

H	-1.32555	-3.26002	0.15087
H	-2.19551	-3.96032	1.555977
C	-7.11425	-0.46476	-0.53662
C	-4.98351	-0.45037	-2.34845
H	-7.95745	-0.48336	0.165899
C	-7.247	0.194479	-1.76704
H	-4.13608	-0.48146	-3.04038
C	-6.17488	0.202308	-2.67873
H	-8.19038	0.692124	-2.02026
H	-6.27495	0.709492	-3.64499

TS$_{RE}$ηS

Symbol	X	Y	Z
P	-0.82571	2.057553	-0.05581
C	-2.46846	1.397178	-0.65457
C	-0.5509	3.528789	-1.16274
C	-1.26655	2.874039	1.552114
C	-2.93919	0.173484	-0.14143
C	-3.21542	2.066148	-1.67296
C	-0.21578	3.286061	-2.51742
C	-0.53972	4.862979	-0.69995
C	-2.49069	3.537451	1.789602
C	-0.29405	2.851046	2.574482
C	-2.25242	-0.5195	1.006065
C	-4.17275	-0.38538	-0.64147
C	-4.39752	1.542635	-2.1645
H	-2.84188	3.015455	-2.06818
C	0.095803	4.344257	-3.38385
H	-0.21065	2.258099	-2.89864
C	-0.21402	5.920863	-1.56656
H	-0.78838	5.079352	0.343754
C	-2.73112	4.168614	3.019687
H	-3.25556	3.560963	1.005001
C	-0.53324	3.486969	3.803702
H	0.653372	2.327566	2.401062
C	-1.11567	-1.33777	0.875534
C	-2.85342	-0.34902	2.318401
C	-4.90658	0.308016	-1.67314
C	-4.70358	-1.62299	-0.15947
H	-4.95904	2.078442	-2.93986
C	0.101268	5.667761	-2.91064
H	0.345121	4.132528	-4.42995
H	-0.21357	6.949171	-1.18605
C	-1.75205	4.146492	4.027758
H	-3.68546	4.679762	3.192395
H	0.231414	3.461484	4.588449
P	-0.0519	-1.70396	-0.60685
C	-0.54458	-1.9456	2.045138
C	-2.28208	-1.0061	3.468539
C	-4.02248	0.446923	2.523949
C	-6.11876	-0.25393	-2.1727
H	-4.16513	-2.15935	0.62777
C	-5.88328	-2.14691	-0.66919
H	0.353899	6.494082	-3.58464
H	-1.94228	4.639007	4.988253
C	-0.11071	-3.56291	-0.68705
C	-0.87187	-1.30957	-2.22463
C	-1.11108	-1.79611	3.293299
H	0.365682	-2.54595	1.942909
C	-2.89399	-0.85577	4.746926
H	-4.47003	0.964655	1.67179
C	-4.60035	0.570703	3.780129
H	-6.66251	0.288865	-2.95564
C	-6.60127	-1.45847	-1.68468
H	-6.26713	-3.09779	-0.2825
C	-1.09711	-4.35513	-0.06466
C	0.880215	-4.1962	-1.46935
C	-2.06638	-1.92665	-2.65287
C	-0.15735	-0.49454	-3.12878
H	-0.65538	-2.28253	4.16388
H	-2.44057	-1.36637	5.605372
C	-4.03621	-0.08603	4.904765
H	-5.49933	1.18502	3.903555
H	-7.53376	-1.88011	-2.07635
C	-1.09212	-5.75129	-0.2241
H	-1.86606	-3.88324	0.556063
C	0.876678	-5.58869	-1.63707
H	1.654414	-3.58244	-1.94709
C	-2.55267	-1.70641	-3.94974
H	-2.61546	-2.58976	-1.97689
C	-0.64218	-0.28354	-4.43105
H	0.798817	-0.05271	-2.81759
H	-4.50086	0.019045	5.891619
C	-0.10896	-6.37104	-1.01145
H	-1.86295	-6.35501	0.268964
H	1.649535	-6.06444	-2.25174
C	-1.84207	-0.88447	-4.84138
H	-3.48576	-2.18576	-4.26594
H	-0.07025	0.3403	-5.12754
H	-0.10825	-7.46001	-1.13475
H	-2.2178	-0.72486	-5.85865
Pd	2.103122	-0.87773	-0.46315
C	3.165094	-1.78856	1.107752
C	2.979291	-1.09349	2.329784
C	3.256623	-3.19853	1.150755
C	2.872587	-1.78591	3.547145
H	2.904282	0.000838	2.326766
C	3.165581	-3.8887	2.372541
H	3.371961	-3.77009	0.223924
C	2.974973	-3.1879	3.576007
H	2.709757	-1.22504	4.475612
H	3.23774	-4.98316	2.378642
H	2.910323	-3.72909	4.526873
C	5.559069	-0.41109	0.847799
C	4.553617	-0.88605	-0.22634
C	6.354879	0.83087	0.393914
H	6.235426	-1.25024	1.095805
H	5.042701	-0.14307	1.783249
C	3.982663	0.236374	-1.05651
C	5.068068	-2.04387	-1.07324
C	5.389807	1.961762	0.105155
H	7.076297	1.12404	1.179052
H	6.953493	0.600856	-0.51199
O	3.446096	0.038121	-2.20432
C	4.192581	1.644319	-0.58486
H	5.311186	-2.92003	-0.44954
H	4.344496	-2.32963	-1.85235
H	5.998808	-1.72889	-1.58739
C	5.63703	3.298286	0.463364
C	3.285105	2.66401	-0.93343
H	6.563214	3.550678	0.994934
C	4.713037	4.306659	0.142934
H	2.38542	2.400611	-1.49811
C	3.535214	3.99021	-0.55831
H	4.915559	5.343222	0.436926
H	2.813382	4.773139	-0.81475

[(BINAP)Pd-((*R*)-**6**)]_conf1

Symbol	X	Y	Z
C	-2.51111	0.285621	-1.94354
C	-2.23758	-1.12624	-1.95578
C	-2.89125	-1.94519	-0.98104
C	-3.75256	-1.39899	-0.03836
C	-4.04413	0.002971	-0.0222
C	-3.425	0.813116	-0.97628
H	-2.25449	0.903076	-2.81311
H	-1.77771	-1.60018	-2.83124
H	-2.71511	-3.02738	-0.99408
H	-4.23152	-2.05232	0.697963
H	-3.62585	1.889454	-1.00129
Pd	-0.41356	-0.07852	-1.09568
C	-4.95612	0.591694	1.083047
C	-6.1002	-0.41851	1.365134
C	-5.60374	1.945083	0.686685
C	-4.11593	0.772183	2.370447
O	-5.97169	-1.30482	2.218484
C	-7.36099	-0.30987	0.562335
C	-6.53711	1.832546	-0.52962
H	-4.81972	2.704033	0.507079
H	-6.1852	2.308252	1.557163
H	-4.72992	1.198876	3.185466
H	-3.72115	-0.19627	2.714393
H	-3.26873	1.451498	2.171044
C	-7.58974	0.762272	-0.33685
C	-8.34729	-1.30703	0.739526
H	-7.02289	2.80498	-0.73639

H	-5.93718	1.585611	-1.42893
C	-8.8082	0.803399	-1.04246
H	-8.13325	-2.11235	1.449899
C	-9.54703	-1.25694	0.026539
H	-8.99814	1.634962	-1.73266
C	-9.77717	-0.19461	-0.86901
H	-10.3049	-2.03624	0.164269
H	-10.7191	-0.14107	-1.42734
P	1.22415	-1.7529	-0.69804
C	2.988097	-1.13382	-0.66791
C	1.231786	-3.00712	-2.07258
C	1.09304	-2.79943	0.821889
C	3.408441	-0.15294	0.25722
C	3.895425	-1.57306	-1.68172
C	1.014325	-2.52476	-3.38523
C	1.367876	-4.39849	-1.88108
C	2.162131	-3.58164	1.311762
C	-0.14826	-2.83338	1.49186
C	2.548563	0.290998	1.413934
C	4.742637	0.393131	0.158814
C	5.176222	-1.06142	-1.78341
H	3.569054	-2.33871	-2.3912
C	0.963514	-3.40336	-4.4771
H	0.873942	-1.44709	-3.53915
C	1.297833	-5.27983	-2.97363
H	1.520684	-4.79902	-0.87368
C	1.985193	-4.39462	2.442124
H	3.135102	-3.54978	0.808128
C	-0.32262	-3.64932	2.621255
H	-0.97345	-2.2137	1.121618
C	1.482269	1.204335	1.278564
C	2.875895	-0.22989	2.726156
C	5.635298	-0.06456	-0.87931
C	5.225836	1.394493	1.058461
H	5.854599	-1.42474	-2.56536
C	1.102334	-4.78734	-4.27339
H	0.801035	-3.00834	-5.48666
H	1.397502	-6.35833	-2.80432
C	0.741935	-4.43243	3.095784
H	2.822049	-4.99501	2.816885
H	-1.29105	-3.66698	3.133903
P	0.810951	1.780867	-0.35451
C	0.690708	1.532384	2.427498
C	2.090083	0.158504	3.873372
C	3.970948	-1.12249	2.947308
C	6.94981	0.481295	-0.97104
H	4.564104	1.758195	1.849823
C	6.510075	1.906772	0.943835
H	1.048364	-5.47773	-5.12273
H	0.606872	-5.06573	3.980043
C	0.078429	3.45554	0.025733
C	2.234223	2.320447	-1.42106
C	0.984952	1.034034	3.680933
H	-0.17052	2.19623	2.306373
C	2.429294	-0.33742	5.16595
H	4.579479	-1.43819	2.095765
C	4.280863	-1.5845	4.218932
H	7.611654	0.117902	-1.76676
C	7.384921	1.447501	-0.07772
H	6.85349	2.672102	1.649276
C	0.587705	4.36799	0.977701
C	-1.02186	3.850719	-0.76423
C	3.203201	3.258707	-1.00786
C	2.269674	1.840626	-2.74631
H	0.367099	1.3107	4.544023
H	1.816588	-0.02653	6.021049
C	3.50754	-1.1905	5.342641
H	5.131374	-2.26219	4.355371
H	8.398045	1.857617	-0.15637
C	0.008565	5.637097	1.132711
H	1.43136	4.081895	1.615648
C	-1.59161	5.126275	-0.6205
H	-1.42737	3.143699	-1.49731
C	4.191054	3.699506	-1.90001
H	3.186489	3.649361	0.014692
C	3.255763	2.28558	-3.64186
H	1.509944	1.114275	-3.06223
H	3.76164	-1.56248	6.341509
C	-1.0799	6.021785	0.331573
H	0.412474	6.329699	1.88039
H	-2.44122	5.417454	-1.24907
C	4.218095	3.215689	-3.21935
H	4.94354	4.421475	-1.56359
H	3.271128	1.90455	-4.6695
H	-1.52829	7.014399	0.452769
H	4.988784	3.565236	-3.91588

[(BINAP)Pd-((*R*)-**6**)]_conf2

Symbol	X	Y	Z
C	-9.19225	-1.36094	0.200802
C	-9.69267	-0.47092	-0.76126
C	-8.95864	0.684824	-1.0709
C	-7.73748	0.943473	-0.43015
C	-7.21387	0.05242	0.533315
C	-7.96831	-1.10155	0.839204
H	-9.75423	-2.26614	0.459369
H	-10.6455	-0.67513	-1.26276
H	-9.33659	1.393552	-1.81699
H	-7.18227	1.854995	-0.67455
H	-7.60978	-1.8124	1.591737
Pd	-0.37312	-0.06936	-1.1439
C	-5.90502	0.396457	1.286451
C	-4.94386	1.132125	0.307017
C	-5.16743	-0.85591	1.830923
C	-6.25756	1.345302	2.457772
O	-4.97362	2.373169	0.22103
C	-4.0142	0.316373	-0.51137
C	-4.71245	-1.81031	0.715631
H	-5.80225	-1.3857	2.565866
H	-4.28054	-0.50324	2.394033
H	-5.35233	1.6016	3.038307
H	-6.69425	2.282307	2.079201
H	-6.98384	0.85921	3.133053
C	-3.8492	-1.10055	-0.30785
C	-3.26014	0.967387	-1.50289
H	-4.16547	-2.67211	1.142444
H	-5.60788	-2.22933	0.212895
C	-2.91989	-1.79077	-1.07688
H	-3.43493	2.037191	-1.65873
C	-2.32139	0.270674	-2.30292
H	-2.79842	-2.87208	-0.93215
C	-2.11872	-1.14031	-2.07422
H	-1.9255	0.75123	-3.20552
H	-1.62004	-1.75755	-2.83112
P	1.163548	-1.77217	-0.6025
C	2.944988	-1.21628	-0.62964
C	1.106148	-3.13822	-1.8628
C	0.98618	-2.67575	1.001011
C	3.417844	-0.20549	0.235592
C	3.817137	-1.75217	-1.62867
C	0.9998	-2.76457	-3.22452
C	1.077168	-4.51094	-1.53797
C	2.012255	-3.47742	1.547489
C	-0.24651	-2.57764	1.680144
C	2.615516	0.365547	1.381253
C	4.782009	0.262199	0.096131
C	5.117549	-1.308	-1.77437
H	3.446683	-2.54067	-2.28914
C	0.898073	-3.73529	-4.23153
H	0.993441	-1.6989	-3.48689
C	0.954121	-5.48163	-2.54693
H	1.145632	-4.82673	-0.49205
C	1.802948	-4.17485	2.747418
H	2.976112	-3.55266	1.031178
C	-0.45263	-3.27604	2.880659
H	-1.03788	-1.94616	1.259165
C	1.625128	1.361879	1.226808
C	2.984746	-0.0633	2.715542
C	5.636544	-0.29132	-0.92689
C	5.336149	1.273649	0.943324
H	5.766622	-1.7443	-2.54363
C	0.870394	-5.10011	-3.89466
H	0.826512	-3.42494	-5.28033
H	0.926846	-6.5429	-2.27364
C	0.570269	-4.07644	3.414408
H	2.606592	-4.79132	3.165874
H	-1.41192	-3.18899	3.403331
P	0.875377	1.811537	-0.42199
C	1.007883	1.924157	2.38884

C	2.345151	0.524593	3.868696
C	3.98424	-1.05928	2.951132
C	6.977395	0.176495	-1.06096
H	4.711638	1.708842	1.727891
C	6.645309	1.707397	0.790952
H	0.775957	-5.85961	-4.67885
H	0.410977	-4.61815	4.353687
C	-0.05157	3.390115	-0.08581
C	2.257923	2.380606	-1.50743
C	1.355968	1.525529	3.664864
H	0.244798	2.696969	2.260143
C	2.724629	0.1087	5.179083
H	4.481396	-1.52339	2.095351
C	4.334969	-1.44152	4.238019
H	7.605965	-0.26102	-1.84628
C	7.478798	1.156479	-0.21919
H	7.040164	2.481377	1.458968
C	0.498448	4.68336	-0.2188
C	-1.41269	3.265236	0.270834
C	3.364876	3.120871	-1.03943
C	2.151108	2.109918	-2.88842
H	0.875008	1.985924	4.536666
H	2.224676	0.572703	6.038126
C	3.703821	-0.85385	5.366582
H	5.107236	-2.20535	4.384187
H	8.511739	1.505379	-0.32876
C	-0.29621	5.818583	0.009662
H	1.543651	4.808652	-0.51914
C	-2.20735	4.398994	0.5009
H	-1.85958	2.267867	0.346963
C	4.338077	3.58778	-1.93674
H	3.47139	3.323536	0.031691
C	3.121134	2.582976	-3.78589
H	1.29909	1.519715	-3.24836
H	3.990767	-1.16231	6.378134
C	-1.64714	5.680332	0.3703
H	0.143359	6.816615	-0.10363
H	-3.26531	4.263591	0.748778
C	4.215292	3.32432	-3.3111
H	5.197995	4.152025	-1.55919
H	3.024877	2.367325	-4.85621
H	-2.2651	6.570493	0.535322
H	4.976179	3.690325	-4.00981

[(BINAP)Pd-((*R*)-**6**)]_conf3

Symbol	X	Y	Z
C	2.966851	2.749701	2.627074
C	2.952128	1.521556	3.343768
C	2.999071	0.318486	2.650409
C	3.05553	0.288876	1.216681
C	3.117729	1.530479	0.47567
C	3.048977	2.747912	1.239357
H	2.926748	3.702091	3.168469
H	2.92106	1.525623	4.439342
H	3.013827	-0.63254	3.192565
H	3.350301	-0.65224	0.739377
H	3.104784	3.705292	0.706629
Pd	0.949837	0.595339	0.461552
C	3.729781	1.616565	-0.95818
C	3.93552	0.191117	-1.53039
C	5.127375	2.307427	-0.88237
C	2.823142	2.405363	-1.92533
O	3.043225	-0.36098	-2.18647
C	5.234648	-0.51223	-1.27084
C	6.130875	1.516594	-0.03267
H	5.010913	3.330981	-0.48342
H	5.522401	2.408842	-1.91321
H	3.309099	2.512596	-2.91281
H	1.865806	1.879014	-2.05847
H	2.614044	3.41545	-1.53419
C	6.291661	0.104283	-0.55081
C	5.388254	-1.8297	-1.76125
H	7.113349	2.024605	-0.01433
H	5.775154	1.487404	1.017655
C	7.478169	-0.62382	-0.33883
H	4.551609	-2.26835	-2.31437
C	6.569937	-2.53994	-1.53847
H	8.303238	-0.15175	0.208885
C	7.619498	-1.93197	-0.82283
H	6.680774	-3.56155	-1.91862
H	8.553619	-2.47852	-0.64841
P	-0.24078	-1.42652	0.94317
C	-2.10126	-1.31627	1.140752
C	0.324673	-2.13794	2.559984
C	-0.0454	-2.84696	-0.22932
C	-2.91235	-0.79558	0.109739
C	-2.70783	-1.69597	2.378933
C	0.21933	-1.33446	3.72265
C	0.990145	-3.37913	2.663379
C	-0.80734	-4.0312	-0.10763
C	0.90833	-2.74239	-1.26182
C	-2.38838	-0.49948	-1.27178
C	-4.32936	-0.61827	0.337263
C	-4.06367	-1.54582	2.603521
H	-2.08418	-2.12456	3.167924
C	0.724121	-1.77821	4.953191
H	-0.26636	-0.35408	3.660061
C	1.518299	-3.8099	3.892358
H	1.097602	-4.01522	1.779322
C	-0.60349	-5.09837	-0.99488
H	-1.55864	-4.11709	0.685995
C	1.10361	-3.8116	-2.15276
H	1.48738	-1.81882	-1.37849
C	-1.64825	0.656329	-1.59079
C	-2.71993	-1.44351	-2.32136
C	-4.90967	-0.99061	1.604647
C	-5.19768	-0.063	-0.65434
H	-4.50138	-1.85626	3.560474
C	1.381391	-3.01793	5.043104
H	0.616298	-1.14621	5.842445
H	2.032599	-4.77648	3.948233
C	0.354243	-4.99032	-2.01849
H	-1.19742	-6.01391	-0.89117
H	1.840022	-3.71339	-2.95833
P	-0.90721	1.807655	-0.34175
C	-1.18509	0.843893	-2.93532
C	-2.28613	-1.19886	-3.67603
C	-3.47877	-2.62956	-2.0743
C	-6.30598	-0.80247	1.824975
H	-4.77814	0.226593	-1.62195
C	-6.55202	0.113801	-0.40922
H	1.787548	-3.35966	6.001836
H	0.507047	-5.82275	-2.71517
C	-0.92538	3.4716	-1.18846
C	-2.1551	2.190036	0.982521
C	-1.50201	-0.04191	-3.94504
H	-0.56559	1.715929	-3.16692
C	-2.64156	-2.11292	-4.71022
H	-3.80405	-2.84894	-1.05412
C	-3.81082	-3.50143	-3.10209
H	-6.72553	-1.09428	2.795655
C	-7.11678	-0.26038	0.839975
H	-7.19245	0.543561	-1.18797
C	-1.83646	3.864319	-2.19393
C	-0.0004	4.422372	-0.70594
C	-3.4644	2.63739	0.709296
C	-1.70794	2.155898	2.319461
H	-1.14502	0.135151	-4.96691
H	-2.30211	-1.90218	-5.73184
C	-3.39621	-3.24292	-4.43505
H	-4.39699	-4.4005	-2.88009
H	-8.18847	-0.11995	1.02062
C	-1.81148	5.170497	-2.70905
H	-2.56272	3.144475	-2.58701
C	0.012992	5.732686	-1.20912
H	0.711072	4.122466	0.072963
C	-4.31458	3.025117	1.754975
H	-3.82283	2.684962	-0.32427
C	-2.55655	2.55346	3.365975
H	-0.6815	1.821592	2.521666
H	-3.66645	-3.93769	-5.23826
C	-0.88927	6.108976	-2.21732
H	-2.52065	5.456372	-3.49479
H	0.735386	6.45889	-0.8185
C	-3.86163	2.986602	3.085125
H	-5.33365	3.359352	1.530218
H	-2.19531	2.527245	4.400691
H	-0.8737	7.128331	-2.6195
H	-4.52591	3.297332	3.899793

[(BINAP)Pd-((*S*)-**6**)]_conf1

Symbol	X	Y	Z
P	-0.80161	-1.73114	0.305974
C	-2.23004	-1.01511	1.287574
C	0.073402	-2.78438	1.563898
C	-1.66464	-3.01315	-0.73254
C	-3.07006	-0.01451	0.755262
C	-2.45646	-1.47956	2.622326
C	0.738205	-2.14032	2.636957
C	0.208766	-4.1843	1.434964
C	-2.78582	-3.74009	-0.27399
C	-1.15559	-3.29025	-2.01761
C	-3.00766	0.479278	-0.6704
C	-4.13378	0.530148	1.575883
C	-3.47037	-0.9725	3.412338
H	-1.81231	-2.26491	3.026485
C	1.490343	-2.87673	3.562431
H	0.65036	-1.05307	2.750998
C	0.982554	-4.91672	2.351953
H	-0.29565	-4.70785	0.616757
C	-3.37672	-4.72279	-1.08304
H	-3.19667	-3.53581	0.721244
C	-1.74327	-4.27704	-2.82599
H	-0.29483	-2.71873	-2.3829
C	-2.08956	1.452597	-1.12377
C	-4.03153	-0.00448	-1.57371
C	-4.33008	0.047948	2.921732
C	-5.0162	1.55422	1.105727
H	-3.62359	-1.35701	4.428346
C	1.621327	-4.26991	3.420767
H	1.982822	-2.3597	4.394284
H	1.076678	-6.00223	2.230944
C	-2.85611	-4.99443	-2.35987
H	-4.24732	-5.27826	-0.71528
H	-1.33509	-4.47921	-3.82283
P	-0.50014	1.861103	-0.24956
C	-2.20073	1.949713	-2.46335
C	-4.12229	0.521472	-2.91573
C	-4.98153	-1.00205	-1.18897
C	-5.37646	0.590829	3.724495
H	-4.8896	1.938145	0.089577
C	-6.0236	2.066551	1.911199
H	2.219016	-4.84422	4.137417
H	-3.32073	-5.76043	-2.99107
C	-0.02638	3.537043	-0.91921
C	-0.87	2.330042	1.508577
C	-3.18685	1.512129	-3.32556
H	-1.4819	2.698479	-2.80954
C	-5.14094	0.049309	-3.79433
H	-4.92692	-1.42617	-0.18296
C	-5.95953	-1.44522	-2.06798
H	-5.50556	0.205093	4.743202
C	-6.21173	1.581721	3.233499
H	-6.6831	2.850025	1.520734
C	-0.89848	4.646771	-0.98672
C	1.312975	3.697666	-1.32945
C	-1.99659	3.075551	1.913654
C	0.108992	2.000071	2.469657
H	-3.25543	1.922944	-4.34053
H	-5.19065	0.466915	-4.80753
C	-6.04783	-0.91477	-3.38243
H	-6.67002	-2.21397	-1.74381
H	-7.01373	1.990393	3.858439
C	-0.4381	5.886654	-1.45342
H	-1.94447	4.540829	-0.67945
C	1.776989	4.943563	-1.78602
H	1.984092	2.830928	-1.29712
C	-2.13897	3.481419	3.249142
H	-2.77953	3.324974	1.190243
C	-0.02571	2.418385	3.803505
H	0.98101	1.412678	2.15563
H	-6.8267	-1.27034	-4.0663
C	0.902747	6.03899	-1.84961
H	-1.12606	6.738706	-1.50434
H	2.822024	5.054241	-2.09778
C	-1.15113	3.160473	4.195325
H	-3.02769	4.046706	3.551364
H	0.746563	2.160081	4.537409
H	1.261188	7.0103	-2.20942
H	-1.26238	3.483729	5.236651
Pd	0.60319	-0.14538	-0.70512
C	3.564533	-0.69736	-1.46422
C	2.320439	-1.19282	-1.96691
C	4.079003	0.490431	-2.02303
C	1.601812	-0.44029	-2.94183
H	2.020066	-2.22163	-1.75584
C	3.398026	1.191675	-3.03829
H	5.054656	0.865652	-1.69399
C	2.14768	0.747621	-3.47903
H	0.696552	-0.8657	-3.39095
H	3.848836	2.093123	-3.46846
H	1.599094	1.295599	-4.25284
C	3.933438	-0.85777	1.041106
C	4.317929	-1.45404	-0.34855
C	4.473728	0.559825	1.267941
H	2.829548	-0.86652	1.127267
H	4.330832	-1.5295	1.827887
C	5.851307	-1.32023	-0.58363
C	3.988605	-2.96496	-0.34554
C	5.971227	0.632219	1.060126
H	4.220085	0.910096	2.286226
H	3.966266	1.257148	0.570136
O	6.431547	-2.07905	-1.36565
C	6.611922	-0.26818	0.168978
H	4.620783	-3.48256	0.396861
H	4.189296	-3.41653	-1.33
H	2.937073	-3.13908	-0.061
C	6.757972	1.589227	1.731175
C	8.009499	-0.19139	-0.03247
H	6.273302	2.28346	2.428971
C	8.142876	1.659625	1.525055
H	8.46129	-0.90609	-0.72833
C	8.774079	0.76558	0.638435
H	8.734981	2.410056	2.061772
H	9.856778	0.819406	0.478812

[(BINAP)Pd-((*S*)-**6**)]_conf2

Symbol	X	Y	Z
P	0.476675	-1.38347	-1.15961
C	2.343415	-1.33202	-1.05583
C	0.1482	-2.25404	-2.76918
C	-0.03582	-2.63684	0.096738
C	3.017307	-0.80545	0.06889
C	3.102007	-1.72712	-2.20258
C	0.280918	-1.49873	-3.96024
C	-0.31967	-3.58158	-2.86554
C	0.70816	-3.80352	0.378937
C	-1.25804	-2.4214	0.767232
C	2.336151	-0.45774	1.372299
C	4.46035	-0.67684	0.034783
C	4.477231	-1.60022	-2.24963
H	2.579172	-2.14964	-3.06455
C	-0.01608	-2.06296	-5.20908
H	0.619653	-0.45659	-3.89639
C	-0.63716	-4.13865	-4.11629
H	-0.44452	-4.18388	-1.96033
C	0.232426	-4.73786	1.311697
H	1.661579	-3.97897	-0.13204
C	-1.73418	-3.35896	1.697264
H	-1.83119	-1.51132	0.552514
C	1.639682	0.751409	1.595696
C	2.521003	-1.38233	2.472607
C	5.19547	-1.07054	-1.14354
C	5.212747	-0.17349	1.143079
H	5.030449	-1.92049	-3.14125
C	-0.48178	-3.38704	-5.29103
H	0.103001	-1.46301	-6.11876
H	-1.00687	-5.1693	-4.1686
C	-0.98937	-4.51762	1.969887
H	0.819372	-5.63786	1.527751
H	-2.68627	-3.17784	2.207656
P	1.14794	1.896599	0.204799
C	1.131348	1.03479	2.902617
C	1.992722	-1.06905	3.779293
C	3.228183	-2.61724	2.325753

C	6.615748	-0.9419	-1.17084
H	4.683372	0.124153	2.052021
C	6.594986	-0.0641	1.089626
H	-0.73024	-3.82505	-6.26424
H	-1.35683	-5.24831	2.699708
C	0.506422	3.405563	1.085418
C	2.731073	2.508821	-0.52872
C	1.301297	0.159975	3.958353
H	0.598838	1.974451	3.071735
C	2.187021	-1.97918	4.860018
H	3.639684	-2.8784	1.347279
C	3.404068	-3.48184	3.396449
H	7.149156	-1.24766	-2.0793
C	7.308837	-0.44987	-0.07651
H	7.1411	0.321477	1.958226
C	1.271417	4.562003	1.350334
C	-0.86048	3.399589	1.45119
C	3.893258	2.760701	0.232087
C	2.744727	2.792195	-1.91116
H	0.90955	0.411837	4.951675
H	1.775683	-1.71672	5.842499
C	2.881357	-3.16459	4.678568
H	3.953204	-4.41902	3.250327
H	8.400563	-0.36025	-0.10646
C	0.688367	5.675006	1.97828
H	2.325887	4.599343	1.058648
C	-1.43508	4.504362	2.09768
H	-1.47303	2.522159	1.211089
C	5.041082	3.290924	-0.37759
H	3.899336	2.536206	1.304275
C	3.891324	3.326737	-2.51926
H	1.846841	2.58175	-2.50471
H	3.027908	-3.85469	5.517049
C	-0.6623	5.648021	2.360967
H	1.296336	6.566645	2.171255
H	-2.49358	4.477712	2.381198
C	5.041001	3.577689	-1.75274
H	5.939714	3.473235	0.222249
H	3.88853	3.541705	-3.59403
H	-1.1134	6.517221	2.853137
H	5.938938	3.989899	-2.227
Pd	-0.4064	0.803304	-1.17904
C	-4.23468	1.358738	-1.30106
C	-3.7518	2.682401	-1.0568
C	-3.43902	0.497151	-2.06488
C	-2.53666	3.115701	-1.57587
H	-4.35913	3.392877	-0.48681
C	-2.18826	0.913104	-2.61884
H	-3.80721	-0.50574	-2.30655
C	-1.72136	2.249968	-2.36478
H	-2.20613	4.145083	-1.39625
H	-1.76038	0.314999	-3.43224
H	-0.92428	2.685747	-2.9796
C	-6.24215	1.77199	0.287028
C	-5.6798	0.950882	-0.90266
C	-5.46607	1.536574	1.59165
H	-6.26577	2.847424	0.033077
H	-7.29792	1.472089	0.438029
C	-5.69046	-0.54928	-0.50853
C	-6.59299	1.147203	-2.13955
C	-5.45652	0.068578	1.959285
H	-5.90147	2.12816	2.418891
H	-4.42297	1.892389	1.466443
O	-5.81074	-1.43114	-1.36733
C	-5.55718	-0.91433	0.941507
H	-7.64458	0.904752	-1.8966
H	-6.27158	0.490616	-2.96248
H	-6.54327	2.196553	-2.47987
C	-5.36793	-0.3515	3.300347
C	-5.5765	-2.2855	1.283657
H	-5.29855	0.403271	4.093561
C	-5.38426	-1.71372	3.632351
H	-5.6661	-3.01131	0.468767
C	-5.49185	-2.68729	2.619965
H	-5.32508	-2.01836	4.683711
H	-5.51771	-3.75186	2.879015

[(BINAP)Pd-((*S*)-**6**)]_conf3

Symbol	X	Y	Z
P	1.384184	1.855078	0.110001
C	2.615977	0.969509	1.199642
C	0.899644	3.342844	1.112531
C	2.417661	2.582035	-1.24344
C	3.269229	-0.20868	0.774568
C	2.806439	1.430829	2.539848
C	-0.08318	3.155783	2.11437
C	1.382814	4.648122	0.878683
C	3.706078	3.115421	-1.01945
C	1.870996	2.637507	-2.54231
C	3.204459	-0.71572	-0.64482
C	4.109574	-0.92986	1.704401
C	3.609571	0.751018	3.436282
H	2.30928	2.350077	2.861281
C	-0.54085	4.235894	2.88298
H	-0.49234	2.152356	2.282092
C	0.907065	5.732397	1.635565
H	2.131831	4.821809	0.099629
C	4.424101	3.701965	-2.07314
H	4.147681	3.069512	-0.01761
C	2.588967	3.226702	-3.59503
H	0.879834	2.201896	-2.71831
C	2.103396	-1.42836	-1.16358
C	4.3664	-0.49661	-1.48303
C	4.275269	-0.4457	3.054061
C	4.792378	-2.13296	1.341212
H	3.745082	1.134082	4.455276
C	-0.04875	5.530928	2.643228
H	-1.29305	4.068343	3.662526
H	1.290884	6.739555	1.435563
C	3.865749	3.761852	-3.36114
H	5.425026	4.108296	-1.88871
H	2.152875	3.260537	-4.5999
P	0.467137	-1.58599	-0.29966
C	2.124929	-1.85599	-2.53154
C	4.375367	-0.97574	-2.84483
C	5.534736	0.180481	-1.01273
C	5.102164	-1.16541	3.966265
H	4.678509	-2.51933	0.324512
C	5.590176	-2.81183	2.251111
H	-0.41554	6.378125	3.23358
H	4.430207	4.217209	-4.18273
C	-0.27224	-3.15224	-0.98254
C	0.767195	-2.11016	1.455791
C	3.221355	-1.64283	-3.34323
H	1.248421	-2.36856	-2.93948
C	5.52822	-0.77497	-3.659
H	5.552005	0.557988	0.013072
C	6.644823	0.356225	-1.82719
H	5.214515	-0.77797	4.986312
C	5.751266	-2.32644	3.57691
H	6.102035	-3.73075	1.943225
C	0.463446	-4.31817	-1.29581
C	-1.67577	-3.17938	-1.11318
C	1.571539	-3.21232	1.814395
C	0.05703	-1.42606	2.463657
H	3.214483	-1.9917	-4.38323
H	5.510329	-1.14998	-4.68969
C	6.648083	-0.12591	-3.16281
H	7.527777	0.873017	-1.4341
H	6.386257	-2.86964	4.28574
C	-0.19651	-5.47698	-1.73291
H	1.556544	-4.31734	-1.21764
C	-2.33759	-4.34184	-1.54103
H	-2.25858	-2.28161	-0.88295
C	1.670725	-3.6123	3.154633
H	2.124485	-3.762	1.045942
C	0.151082	-1.83057	3.805145
H	-0.57319	-0.57441	2.177377
H	7.530842	0.017689	-3.79591
C	-1.59757	-5.49248	-1.85365
H	0.386537	-6.37216	-1.97957
H	-3.4288	-4.32693	-1.63341
C	0.959454	-2.9239	4.152393
H	2.306804	-4.46369	3.421258
H	-0.40777	-1.29051	4.578103
H	-2.10762	-6.40045	-2.19555
H	1.035492	-3.2421	5.198436
Pd	-0.46925	0.561936	-0.58202
C	-7.35067	-0.02825	0.111647

C	-8.11454	0.843476	0.917998
C	-7.79307	-0.25863	-1.21027
C	-9.26891	1.47043	0.420628
H	-7.8186	1.035335	1.955119
C	-8.945	0.367799	-1.70957
H	-7.22926	-0.94454	-1.85111
C	-9.68855	1.239141	-0.89792
H	-9.84039	2.143168	1.07098
H	-9.26146	0.1717	-2.7407
H	-10.5868	1.731103	-1.28791
C	-5.42552	-0.05102	1.841033
C	-6.12218	-0.78984	0.667599
C	-4.85256	1.315149	1.432154
H	-6.12147	0.057041	2.694207
H	-4.60143	-0.69818	2.201162
C	-5.08064	-0.96091	-0.47609
C	-6.59277	-2.18778	1.137828
C	-3.91642	1.199291	0.246221
H	-4.32753	1.785364	2.284906
H	-5.68895	1.994574	1.169254
O	-5.10443	-1.97427	-1.19956
C	-4.081	0.11135	-0.67635
H	-5.74846	-2.76141	1.56216
H	-7.00207	-2.76292	0.292918
H	-7.3741	-2.08461	1.911744
C	-2.924	2.151482	0.013099
C	-3.22556	0.021738	-1.79218
H	-2.81114	2.992966	0.707748
C	-2.05904	2.086649	-1.12897
H	-3.37675	-0.81242	-2.48568
C	-2.22908	0.988558	-2.04206
H	-1.50282	2.980366	-1.42934
H	-1.70073	0.9814	-3.00296

6_conf1

Symbol	X	Y	Z
C	1.665988	0.119166	0.091797
C	2.420539	-0.80325	0.848983
C	1.945771	0.22279	-1.28875
C	3.408774	-1.59979	0.248835
H	2.251263	-0.90039	1.926813
C	2.930892	-0.57424	-1.89041
H	1.389816	0.942345	-1.89837
C	3.665958	-1.49313	-1.12589
H	3.978393	-2.30671	0.86324
H	3.123201	-0.47377	-2.96481
H	4.433783	-2.11789	-1.59581
C	0.00617	0.465809	2.047199
C	0.629745	1.060557	0.756629
C	-0.76887	-0.83643	1.793132
H	0.786308	0.311045	2.815356
H	-0.68498	1.22362	2.465878
C	-0.5226	1.310862	-0.25365
C	1.324235	2.405773	1.077281
C	-1.80727	-0.67296	0.70351
H	-1.25389	-1.18461	2.724455
H	-0.05944	-1.63559	1.497126
O	-0.48072	2.261522	-1.04272
C	-1.67297	0.352439	-0.26646
H	0.621164	3.100684	1.571701
H	1.683449	2.886279	0.154262
H	2.183942	2.236213	1.748974
C	-2.9223	-1.52971	0.629108
C	-2.64912	0.502731	-1.27781
H	-3.04109	-2.3193	1.381552
C	-3.88021	-1.38138	-0.383
H	-2.5113	1.312726	-2.00154
C	-3.74457	-0.36018	-1.34289
H	-4.74118	-2.05881	-0.42003
H	-4.49469	-0.24244	-2.13271

6_conf2

Symbol	X	Y	Z
C	-1.97843	0.129136	0.064973
C	-3.12147	0.68434	0.670942
C	-2.17124	-0.86651	-0.91824
C	-4.41248	0.262739	0.308727
H	-3.01752	1.45589	1.440351
C	-3.45543	-1.29217	-1.28084
H	-1.30058	-1.32937	-1.39749
C	-4.58603	-0.7266	-0.66843
H	-5.28399	0.711922	0.798964
H	-3.57324	-2.07284	-2.04115
H	-5.59201	-1.05856	-0.9489
C	-0.05009	1.464402	-0.83209
C	-0.55609	0.620216	0.37561
C	1.416426	1.901132	-0.69464
H	-0.16319	0.864285	-1.75502
H	-0.70587	2.347399	-0.94937
C	0.395737	-0.58506	0.62032
C	-0.48676	1.448577	1.687153
C	2.334828	0.730934	-0.40836
H	1.511261	2.650441	0.118094
H	1.745239	2.418573	-1.61545
O	-0.00569	-1.60725	1.181326
C	1.836246	-0.43768	0.223112
H	0.557067	1.68768	1.955033
H	-0.92375	0.878355	2.523834
H	-1.03092	2.40461	1.58237
C	3.700184	0.782376	-0.7505
C	2.706188	-1.51725	0.499075
H	4.092814	1.67847	-1.24717
C	4.555763	-0.29224	-0.47296
H	2.279869	-2.39939	0.98805
C	4.057786	-1.44927	0.155906
H	5.613728	-0.2313	-0.75319
H	4.725709	-2.29048	0.371867

TS$_{\mathbf{RE}}$**CR**$^{\mathbf{ind}}$_conf1

Symbol	X	Y	Z
C	-1.77368	4.909324	-1.07875
C	-2.0534	4.719459	-2.444
C	-2.40697	3.438142	-2.89695
C	-2.49488	2.365876	-1.98951
C	-2.1852	2.535378	-0.61557
C	-1.83647	3.834743	-0.17716
H	-1.48805	5.902009	-0.70982
H	-2.00647	5.562256	-3.14285
H	-2.63337	3.268099	-3.95686
H	-2.79635	1.379973	-2.36533
H	-1.58619	4.009017	0.873515
Pd	-1.33038	0.747442	0.067701
C	-3.60168	1.52438	0.705763
C	-3.28403	0.235117	1.43509
C	-4.78696	1.213152	-0.22454
C	-3.80621	2.685258	1.668743
O	-2.66016	0.140659	2.527984
C	-4.13437	-0.84281	0.835871
H	-4.64211	1.561418	-1.26234
H	-5.68265	1.753438	0.1474
H	-4.65046	2.432843	2.342936
H	-2.92154	2.838801	2.306073
H	-4.04629	3.626477	1.14804
C	-5.00706	-0.28417	-0.11935
C	-4.22458	-2.19681	1.19724
C	-5.95973	-1.0865	-0.75986
H	-3.55795	-2.60862	1.962306
C	-5.18422	-2.99795	0.559466
H	-6.64935	-0.65961	-1.49903
C	-6.0421	-2.44875	-0.41433
H	-5.27134	-4.05849	0.821687
H	-6.79115	-3.08658	-0.89784
P	-0.56102	-1.53947	-0.32529
C	0.947148	-1.36241	-1.423
C	-1.68202	-2.59976	-1.37417
C	-0.0767	-2.68795	1.041522
C	2.198301	-0.90674	-0.95371
C	0.7546	-1.54689	-2.82877
C	-2.60107	-1.93661	-2.21505
C	-1.64306	-4.01004	-1.39604
C	0.781105	-3.79306	0.845738
C	-0.64447	-2.47051	2.31646
C	2.518447	-0.76304	0.512251
C	3.261297	-0.64237	-1.90222

C	1.762233	-1.30325	-3.74217
H	-0.21317	-1.90313	-3.19078
C	-3.4325	-2.65853	-3.0847
H	-2.66042	-0.84264	-2.17431
C	-2.48245	-4.73339	-2.25803
H	-0.96219	-4.54986	-0.73095
C	1.050776	-4.67856	1.900479
H	1.252268	-3.95257	-0.1304
C	-0.37019	-3.36196	3.367868
H	-1.29966	-1.6053	2.48834
C	2.047118	0.310301	1.295175
C	3.395504	-1.74733	1.109774
C	3.03838	-0.84748	-3.3134
C	4.554578	-0.18587	-1.49662
H	1.586672	-1.46881	-4.8123
C	-3.37252	-4.06087	-3.11073
H	-4.13824	-2.12444	-3.73058
H	-2.43923	-5.82877	-2.26107
C	0.47103	-4.46687	3.162431
H	1.725436	-5.52656	1.738371
H	-0.81474	-3.18393	4.353808
P	0.874727	1.583799	0.592985
C	2.415436	0.391234	2.674664
C	3.758415	-1.63601	2.502221
C	3.930126	-2.85001	0.371508
C	4.09419	-0.60483	-4.241
H	4.74737	-0.01402	-0.43453
C	5.563374	0.039964	-2.42193
H	-4.02543	-4.62792	-3.7841
H	0.684175	-5.15817	3.98599
C	0.694971	2.789761	1.998115
C	1.910419	2.5005	-0.63473
C	3.242366	-0.54905	3.259535
H	2.039371	1.220924	3.278582
C	4.625916	-2.60554	3.087138
H	3.668676	-2.9588	-0.68447
C	4.77539	-3.77544	0.966819
H	3.899541	-0.77028	-5.30773
C	5.337448	-0.17268	-3.80811
H	6.544182	0.388125	-2.07869
C	1.43281	3.987931	2.096979
C	-0.26143	2.488885	2.998144
C	3.278048	2.778373	-0.41225
C	1.297315	2.965584	-1.81697
H	3.516673	-0.45935	4.317793
H	4.887334	-2.49936	4.147083
C	5.130475	-3.65604	2.337402
H	5.173698	-4.60573	0.372522
H	6.14289	0.007847	-4.52881
C	1.233004	4.860844	3.180152
H	2.161925	4.24872	1.323578
C	-0.44355	3.357434	4.086473
H	-0.88661	1.588471	2.911676
C	4.013336	3.519249	-1.35009
H	3.7674	2.411818	0.497331
C	2.038088	3.704082	-2.75382
H	0.24015	2.748278	-2.00159
H	5.799905	-4.39228	2.796319
C	0.301315	4.545674	4.181406
H	1.810312	5.791016	3.237639
H	-1.18415	3.106967	4.854617
C	3.393677	3.984776	-2.52196
H	5.073786	3.727833	-1.16768
H	1.549041	4.059261	-3.6677
H	0.148208	5.226813	5.026381
H	3.970229	4.560124	-3.25538

TS$_{RE}$CR^{ind}_conf2

Symbol	X	Y	Z
C	-4.32955	-1.89455	-2.02794
C	-4.46225	-3.15926	-1.43564
C	-3.88224	-3.38925	-0.17344
C	-3.18275	-2.37363	0.490181
C	-3.01906	-1.09512	-0.10559
C	-3.63165	-0.86821	-1.36556
H	-4.79009	-1.68911	-3.00254
H	-5.02143	-3.95419	-1.94168
H	-3.97856	-4.37216	0.303436
H	-2.77394	-2.57168	1.485901
H	-3.57905	0.121952	-1.83386
Pd	-1.26225	0.023793	0.112471
C	-3.3654	0.547326	1.17364
C	-4.70948	-0.10235	1.568838
C	-3.77238	1.802114	0.348212
C	-2.6128	0.798076	2.501182
O	-4.86783	-0.95815	2.442426
C	-5.7942	0.590648	0.825693
H	-3.23141	1.888528	-0.61095
H	-3.52695	2.716885	0.924316
H	-3.25537	1.403744	3.176929
H	-2.43744	-0.15876	3.019556
H	-1.65263	1.323949	2.384883
C	-5.26929	1.689698	0.124385
C	-7.1669	0.29238	0.816704
C	-6.12391	2.508174	-0.6301
H	-7.53972	-0.56686	1.385086
C	-8.0189	1.110933	0.064102
H	-5.73585	3.369978	-1.18736
C	-7.49738	2.209422	-0.65468
H	-9.09423	0.902306	0.028442
H	-8.17683	2.842294	-1.23781
P	0.454873	-1.65701	-0.26491
C	2.059812	-1.16704	-1.09806
C	-0.10582	-3.0919	-1.30457
C	0.997396	-2.47055	1.308256
C	2.926024	-0.19978	-0.54284
C	2.34594	-1.6886	-2.39906
C	-0.86219	-2.79814	-2.46104
C	0.17513	-4.44076	-1.00169
C	2.172205	-3.24908	1.398373
C	0.174143	-2.3423	2.446305
C	2.733552	0.320589	0.854364
C	4.065354	0.26293	-1.30024
C	3.445576	-1.2715	-3.1253
H	1.682794	-2.4449	-2.82659
C	-1.29568	-3.82551	-3.31215
H	-1.1309	-1.7595	-2.68073
C	-0.26974	-5.46838	-1.84871
H	0.740088	-4.69298	-0.09896
C	2.507372	-3.89297	2.599639
H	2.83096	-3.34255	0.527964
C	0.507348	-2.99124	3.645623
H	-0.72594	-1.71955	2.381384
C	1.716831	1.232928	1.191419
C	3.638291	-0.15027	1.885709
C	4.328039	-0.28227	-2.6101
C	4.952323	1.270477	-0.80692
H	3.647176	-1.70212	-4.11396
C	-0.99887	-5.16414	-3.00917
H	-1.88354	-3.57915	-4.20329
H	-0.04482	-6.51147	-1.5975
C	1.673866	-3.76907	3.723425
H	3.426376	-4.4867	2.65902
H	-0.1428	-2.88274	4.521071
P	0.384036	1.829341	0.044926
C	1.526761	1.596443	2.566446
C	3.456175	0.275953	3.251954
C	4.726831	-1.03404	1.607856
C	5.453949	0.178878	-3.35335
H	4.76385	1.705097	0.179168
C	6.038059	1.702451	-1.55562
H	-1.34577	-5.96803	-3.66849
H	1.938345	-4.27177	4.660649
C	0.183697	3.617989	0.552844
C	1.079222	2.122032	-1.65296
C	2.366102	1.138589	3.561368
H	0.700406	2.263785	2.832235
C	4.35658	-0.17403	4.260685
H	4.882283	-1.37967	0.582564
C	5.594808	-1.44764	2.609553
H	5.636802	-0.25165	-4.34554
C	6.297915	1.151317	-2.83942
H	6.701537	2.476109	-1.15251
C	1.179594	4.385923	1.19736
C	-1.01807	4.256672	0.177875
C	2.126129	3.032773	-1.9103
C	0.429468	1.500668	-2.73901
H	2.203515	1.440093	4.603303
H	4.196516	0.16435	5.291692

C	5.413727	-1.01565	3.949529
H	6.426967	-2.11643	2.361698
H	7.160448	1.498068	-3.41954
C	0.968463	5.747062	1.47128
H	2.123491	3.918151	1.49643
C	-1.22477	5.619514	0.441735
H	-1.79573	3.680086	-0.33377
C	2.52045	3.304502	-3.22808
H	2.627377	3.541663	-1.08043
C	0.821042	1.777702	-4.05885
H	-0.38759	0.797596	-2.53065
H	6.104269	-1.34968	4.731965
C	-0.23339	6.368448	1.095588
H	1.751365	6.324231	1.976979
H	-2.16518	6.094822	0.140092
C	1.867279	2.68021	-4.30476
H	3.338592	4.008907	-3.41525
H	0.30575	1.289198	-4.89377
H	-0.39574	7.430509	1.310936
H	2.172578	2.90144	-5.3339

TS$_{RE}$CR^{ind}_conf3

Symbol	X	Y	Z
C	-4.02511	-0.28237	-3.64776
C	-4.74261	-1.39643	-3.18682
C	-4.71154	-1.71252	-1.81571
C	-3.98925	-0.91719	-0.91491
C	-3.26166	0.21649	-1.36319
C	-3.30045	0.520467	-2.74778
H	-4.03921	-0.01853	-4.71276
H	-5.31965	-2.01348	-3.88442
H	-5.24686	-2.59458	-1.44375
H	-3.98195	-1.18175	0.148737
H	-2.77432	1.404763	-3.12587
Pd	-1.40407	0.694428	-0.54994
C	-3.48203	1.968855	-0.24824
C	-2.59205	2.326358	0.949151
C	-4.80648	1.544125	0.44292
C	-3.63274	3.095915	-1.25758
O	-1.81078	3.293489	1.039861
C	-3.10136	1.535726	2.120441
H	-5.41786	0.825125	-0.12496
H	-5.41825	2.466149	0.559637
H	-4.01852	3.997553	-0.74153
H	-2.6692	3.371357	-1.71574
H	-4.33978	2.829495	-2.06071
C	-4.39651	1.066255	1.821703
C	-2.53275	1.359221	3.390936
C	-5.1295	0.36148	2.787034
H	-1.53367	1.752222	3.605324
C	-3.26828	0.655132	4.357752
H	-6.14022	-0.00587	2.571332
C	-4.55275	0.155492	4.054683
H	-2.84548	0.492421	5.355873
H	-5.11588	-0.38831	4.822389
P	-0.3049	-1.53683	-0.37704
C	1.533434	-1.73814	-0.70532
C	-1.04591	-2.72942	-1.59369
C	-0.51818	-2.42664	1.234657
C	2.49352	-1.00392	0.020664
C	1.969956	-2.6096	-1.75271
C	-1.07745	-2.34382	-2.95389
C	-1.60619	-3.97198	-1.23083
C	0.226156	-3.58527	1.552766
C	-1.48939	-1.96497	2.145148
C	2.136145	-0.14553	1.202969
C	3.893222	-1.11625	-0.32749
C	3.306471	-2.74602	-2.07787
H	1.22891	-3.19253	-2.3049
C	-1.61958	-3.19415	-3.92742
H	-0.66685	-1.37265	-3.25014
C	-2.16297	-4.81578	-2.20638
H	-1.60601	-4.28729	-0.18311
C	-0.00917	-4.27239	2.753092
H	0.988411	-3.95104	0.855783
C	-1.72491	-2.65643	3.34498
H	-2.05238	-1.05247	1.918705
C	1.532262	1.119487	1.079237
C	2.490714	-0.63874	2.520585
C	4.303146	-1.9995	-1.39175
C	4.909052	-0.35894	0.336423
H	3.610482	-3.43401	-2.87646
C	-2.16572	-4.43435	-3.55662
H	-1.6275	-2.88056	-4.97739
H	-2.59302	-5.77821	-1.90529
C	-0.98786	-3.81093	3.649907
H	0.575641	-5.16878	2.989567
H	-2.48156	-2.2788	4.040845
P	0.787925	1.82962	-0.46214
C	1.224809	1.875372	2.261214
C	2.223122	0.166633	3.687262
C	3.115561	-1.90822	2.72069
C	5.684048	-2.10311	-1.73257
H	4.620903	0.324085	1.140747
C	6.244538	-0.47364	-0.02323
H	-2.59682	-5.09613	-4.3165
H	-1.16836	-4.34793	4.588237
C	1.179598	3.642987	-0.29661
C	1.826541	1.451555	-1.9543
C	1.573748	1.423317	3.51765
H	0.69128	2.825714	2.15807
C	2.602232	-0.30809	4.976477
H	3.317201	-2.54178	1.853282
C	3.473954	-2.34477	3.988843
H	5.972272	-2.78447	-2.54243
C	6.641404	-1.35675	-1.0635
H	7.000339	0.12085	0.502535
C	2.388882	4.116135	0.259828
C	0.270121	4.572511	-0.83821
C	3.218013	1.668688	-2.03263
C	1.131683	1.063446	-3.11892
H	1.350168	2.030662	4.40362
H	2.392167	0.324228	5.847945
C	3.221546	-1.53942	5.13002
H	3.954885	-3.32221	4.108825
H	7.699542	-1.44283	-1.33491
C	2.683365	5.487861	0.262796
H	3.097793	3.411161	0.707948
C	0.569329	5.94425	-0.84241
H	-0.68904	4.218706	-1.22552
C	3.898224	1.485489	-3.24535
H	3.776681	1.981993	-1.1454
C	1.810382	0.890329	-4.33641
H	0.046054	0.908854	-3.05694
H	3.510958	-1.89311	6.125968
C	1.776042	6.405018	-0.29399
H	3.624015	5.840537	0.701678
H	-0.1513	6.655014	-1.26265
C	3.196364	1.099713	-4.40049
H	4.981115	1.647106	-3.28794
H	1.25458	0.594559	-5.23375
H	2.0067	7.476525	-0.29136
H	3.730465	0.966352	-5.34826

TS$_{RE}$CR^{ind}_conf4

Symbol	X	Y	Z
C	-3.57601	2.236435	2.471604
C	-3.83181	3.544156	2.031681
C	-3.61435	3.867447	0.67908
C	-3.12408	2.9079	-0.21764
C	-2.8141	1.59808	0.224926
C	-3.09769	1.265268	1.573362
H	-3.77151	1.955751	3.514113
H	-4.22108	4.297497	2.725793
H	-3.83289	4.879113	0.315604
H	-3.00676	3.167162	-1.27473
H	-2.95856	0.237352	1.928702
Pd	-1.18697	0.512735	-0.49171
C	-3.45547	0.035573	-1.11395
C	-4.85843	0.67106	-1.06653
C	-3.63005	-1.34175	-0.40785
C	-3.02503	0.030873	-2.59404
O	-5.24711	1.620466	-1.75223
C	-5.71083	-0.15824	-0.17457
H	-2.86762	-1.55269	0.361935
H	-3.5339	-2.15547	-1.15518

H	-3.79477	-0.49283	-3.20124
H	-2.97846	1.065183	-2.97365
H	-2.06214	-0.47037	-2.78044
C	-5.01851	-1.32192	0.202592
C	-7.03549	0.079529	0.228609
C	-5.64469	-2.2702	1.026795
H	-7.54666	0.992293	-0.09686
C	-7.65914	-0.86792	1.050584
H	-5.12142	-3.18392	1.334837
C	-6.96509	-2.03302	1.446388
H	-8.6887	-0.70947	1.391466
H	-7.46779	-2.76675	2.087755
P	0.191531	-1.57078	-0.62038
C	2.045718	-1.52701	-0.89748
C	-0.41306	-2.74909	-1.92222
C	0.065097	-2.55112	0.948645
C	2.878481	-0.77676	-0.04162
C	2.622746	-2.20455	-2.01584
C	-0.32815	-2.34711	-3.27881
C	-1.08573	-3.9545	-1.62455
C	0.702191	-3.80249	1.108029
C	-0.67831	-2.02031	2.022159
C	2.376296	-0.15525	1.232635
C	4.289312	-0.66405	-0.33826
C	3.974335	-2.12582	-2.29551
H	1.984733	-2.81407	-2.66057
C	-0.86426	-3.14218	-4.30211
H	0.170645	-1.40482	-3.53625
C	-1.63661	-4.74112	-2.65046
H	-1.18283	-4.28268	-0.58531
C	0.569463	-4.5176	2.307573
H	1.303736	-4.21758	0.29125
C	-0.806	-2.7351	3.224522
H	-1.12947	-1.0257	1.913735
C	1.591489	1.013548	1.268608
C	2.756752	-0.79643	2.477952
C	4.841938	-1.34444	-1.48408
C	5.175938	0.124845	0.45955
H	4.391224	-2.66929	-3.15241
C	-1.52347	-4.34372	-3.99149
H	-0.77565	-2.81552	-5.34445
H	-2.15418	-5.67302	-2.39512
C	-0.18933	-3.98762	3.365709
H	1.065617	-5.48848	2.419379
H	-1.3823	-2.3073	4.052356
P	0.785238	1.827681	-0.18354
C	1.137023	1.516784	2.534803
C	2.336541	-0.23139	3.737373
C	3.540442	-1.99111	2.518335
C	6.233253	-1.22495	-1.7734
H	4.776108	0.655562	1.328425
C	6.524441	0.228975	0.148664
H	-1.95183	-4.9605	-4.78924
H	-0.28791	-4.54677	4.302993
C	0.884372	3.639023	0.229978
C	1.912138	1.824143	-1.65913
C	1.509374	0.928346	3.72559
H	0.481747	2.393173	2.552546
C	2.739002	-0.84461	4.958869
H	3.847291	-2.45741	1.578635
C	3.915886	-2.5681	3.724175
H	6.632732	-1.75486	-2.64679
C	7.063657	-0.4545	-0.97462
H	7.17978	0.842747	0.777017
C	1.884421	4.212757	1.047218
C	-0.0511	4.488005	-0.39747
C	3.246025	2.280124	-1.61671
C	1.344304	1.482376	-2.90429
H	1.164254	1.347008	4.678914
H	2.412438	-0.39083	5.9027
C	3.521541	-1.98957	4.95869
H	4.520597	-3.48209	3.720709
H	8.131067	-0.36888	-1.20689
C	1.942209	5.602729	1.230702
H	2.615224	3.571095	1.551469
C	0.013978	5.879191	-0.21937
H	-0.83926	4.051973	-1.01882
C	4.000849	2.374658	-2.79496
H	3.696364	2.567271	-0.66109
C	2.097762	1.58584	-4.08541
H	0.298579	1.148111	-2.93388
H	3.827831	-2.45115	5.904133
C	1.009263	6.439996	0.595815
H	2.722454	6.032918	1.869461
H	-0.72174	6.523998	-0.71317
C	3.428027	2.029859	-4.03148
H	5.038995	2.72184	-2.74729
H	1.641773	1.326138	-5.04784
H	1.05729	7.525375	0.739649
H	4.017808	2.113743	-4.95147

TS$_{RE}$CR^{ind}_conf5

Symbol	X	Y	Z
C	-4.51226	-2.32078	-2.27067
C	-4.98164	-3.23892	-1.31522
C	-4.66997	-3.03674	0.040249
C	-3.90015	-1.93168	0.439868
C	-3.43178	-0.99094	-0.50679
C	-3.76429	-1.20082	-1.872
H	-4.75483	-2.45744	-3.33218
H	-5.59058	-4.09682	-1.62183
H	-5.01744	-3.75151	0.796092
H	-3.66236	-1.81078	1.503629
H	-3.47511	-0.45128	-2.61967
Pd	-1.68275	0.13048	-0.37677
C	-3.96158	1.005483	0.0765
C	-3.282	2.249935	-0.45358
C	-3.92273	1.091332	1.615
C	-5.3579	0.867585	-0.52768
O	-3.38076	2.699275	-1.61407
C	-2.73531	2.993987	0.734544
H	-3.53935	0.179633	2.106418
H	-4.95199	1.230431	2.008339
H	-5.9649	1.734045	-0.18951
H	-5.31199	0.910909	-1.62632
H	-5.86713	-0.0594	-0.21825
C	-3.08889	2.323927	1.923914
C	-2.09846	4.24578	0.768906
C	-2.74575	2.869032	3.16863
H	-1.8774	4.77353	-0.16393
C	-1.76639	4.796296	2.018119
H	-3.02268	2.360683	4.100983
C	-2.07671	4.107386	3.208333
H	-1.27298	5.773909	2.070439
H	-1.81847	4.553246	4.176157
P	-0.1903	-1.7054	0.340833
C	1.549764	-1.89514	-0.33212
C	-0.91109	-3.39077	0.05722
C	0.059953	-1.63225	2.17248
C	2.528291	-0.89952	-0.13067
C	1.860205	-3.01656	-1.16344
C	-1.35355	-3.69264	-1.25094
C	-1.05795	-4.36714	1.064082
C	0.955328	-2.49065	2.849492
C	-0.68806	-0.69896	2.918397
C	2.299171	0.254264	0.801073
C	3.810713	-1.0107	-0.78641
C	3.093209	-3.14951	-1.77375
H	1.109562	-3.79689	-1.31046
C	-1.88784	-4.95333	-1.55255
H	-1.29083	-2.92923	-2.03343
C	-1.60566	-5.62494	0.762204
H	-0.74805	-4.14895	2.090561
C	1.073851	-2.43242	4.246366
H	1.565154	-3.20148	2.28081
C	-0.56524	-0.63974	4.315731
H	-1.34787	-0.00171	2.38716
C	1.433519	1.317921	0.493381
C	3.003436	0.25197	2.069745
C	4.096765	-2.15365	-1.61829
C	4.820009	-0.00482	-0.66446
H	3.307601	-4.03013	-2.39171
C	-2.01247	-5.92565	-0.54661
H	-2.22427	-5.16861	-2.57257
H	-1.71129	-6.37223	1.557277
C	0.309843	-1.51098	4.982573
H	1.77302	-3.10173	4.759966
H	-1.14743	0.097261	4.880014
P	0.304956	1.436562	-0.9767

C	1.199853	2.343055	1.469639
C	2.79179	1.324702	3.010205
C	3.910896	-0.78693	2.442814
C	5.363535	-2.25701	-2.26459
H	4.618313	0.877106	-0.04944
C	6.040863	-0.12866	-1.31261
H	-2.43702	-6.90869	-0.78006
H	0.408181	-1.46486	6.073041
C	0.494497	3.219672	-1.48582
C	1.079927	0.672609	-2.48437
C	1.861658	2.351716	2.680116
H	0.477012	3.135835	1.250475
C	3.4993	1.330943	4.2474
H	4.077273	-1.62059	1.755734
C	4.589813	-0.7524	3.65362
H	5.561602	-3.13701	-2.8887
C	6.321987	-1.26629	-2.11645
H	6.795375	0.658559	-1.20284
C	1.651246	3.983868	-1.2144
C	-0.52438	3.778267	-2.28864
C	2.340998	1.064335	-2.98046
C	0.294365	-0.22068	-3.24153
H	1.669633	3.15208	3.404766
H	3.323913	2.159749	4.944248
C	4.389046	0.315997	4.566028
H	5.285766	-1.56036	3.906752
H	7.291488	-1.355	-2.61932
C	1.787527	5.27926	-1.7379
H	2.447127	3.571071	-0.58532
C	-0.37923	5.073602	-2.81379
H	-1.45366	3.224322	-2.46323
C	2.812134	0.556803	-4.19959
H	2.954268	1.776246	-2.41881
C	0.761487	-0.71934	-4.46876
H	-0.69354	-0.51064	-2.85841
H	4.931337	0.333963	5.518078
C	0.774718	5.82607	-2.54335
H	2.690533	5.860515	-1.51706
H	-1.18196	5.495707	-3.42906
C	2.022032	-0.33236	-4.94848
H	3.797342	0.862362	-4.56911
H	0.136335	-1.4062	-5.05086
H	0.884184	6.836452	-2.95402
H	2.387978	-0.71706	-5.90737

TS$_{RE}$CS^{ind}_conf1

Symbol	X	Y	Z
P	0.599022	-1.65723	-0.55338
C	2.34619	-1.03025	-0.80285
C	0.318161	-2.68806	-2.08025
C	0.836276	-2.95794	0.74831
C	2.925098	-0.12033	0.105646
C	3.08143	-1.41977	-1.96515
C	-0.33545	-2.07818	-3.17361
C	0.701712	-4.0415	-2.19129
C	2.06712	-3.62456	0.941763
C	-0.27307	-3.32216	1.539542
C	2.220579	0.297746	1.368051
C	4.245302	0.404952	-0.1487
C	4.352265	-0.93394	-2.21554
H	2.629945	-2.1247	-2.66898
C	-0.57573	-2.79055	-4.35855
H	-0.67868	-1.04046	-3.07179
C	0.452261	-4.75822	-3.37353
H	1.190674	-4.54028	-1.34803
C	2.181033	-4.64158	1.902604
H	2.937877	-3.34855	0.336771
C	-0.15361	-4.33953	2.499756
H	-1.23026	-2.80991	1.40692
C	1.166216	1.229601	1.360722
C	2.664506	-0.26704	2.625461
C	4.968632	-0.01068	-1.3264
C	4.872612	1.347487	0.724235
H	4.899745	-1.25779	-3.10935
C	-0.18104	-4.13493	-4.46092
H	-1.0865	-2.30025	-5.19511
H	0.751399	-5.81061	-3.44141
C	1.07031	-5.00203	2.683377
H	3.141483	-5.15126	2.042395
H	-1.02622	-4.61076	3.104428
P	0.349196	1.865908	-0.17955
C	0.484637	1.529369	2.586352
C	1.996929	0.097421	3.852464
C	3.760077	-1.18031	2.710799
C	6.272569	0.512098	-1.57249
H	4.33637	1.679069	1.618092
C	6.141211	1.841144	0.456087
H	-0.37762	-4.69797	-5.38038
H	1.161203	-5.79502	3.434655
C	-0.12275	3.619525	0.265477
C	1.639302	2.289785	-1.45379
C	0.889972	0.990593	3.791324
H	-0.37629	2.205147	2.564982
C	2.450039	-0.44376	5.090675
H	4.276717	-1.47488	1.793622
C	4.182389	-1.68661	3.932188
H	6.807178	0.181623	-2.47146
C	6.852596	1.41973	-0.69989
H	6.59972	2.561613	1.142998
C	0.48625	4.384127	1.285784
C	-1.09029	4.243157	-0.55327
C	2.649457	3.251194	-1.23221
C	1.507441	1.723467	-2.73769
H	0.359202	1.245397	4.716765
H	1.927289	-0.15338	6.010201
C	3.525618	-1.31741	5.135607
H	5.029547	-2.38083	3.966777
H	7.855519	1.813922	-0.89867
C	0.12023	5.724549	1.491823
H	1.247297	3.930376	1.929247
C	-1.44668	5.585984	-0.35579
H	-1.56094	3.672008	-1.36171
C	3.513844	3.62501	-2.27051
H	2.753956	3.72099	-0.24861
C	2.369422	2.102983	-3.77973
H	0.723957	0.977717	-2.91433
H	3.866745	-1.72564	6.0935
C	-0.84678	6.329743	0.673525
H	0.600223	6.29872	2.292914
H	-2.19772	6.049799	-1.00537
C	3.373805	3.054606	-3.54759
H	4.297301	4.367792	-2.08289
H	2.252463	1.654078	-4.77263
H	-1.12965	7.375925	0.835363
H	4.045608	3.355507	-4.35956
Pd	-1.19623	-0.00014	-0.46045
C	-2.87428	-1.19155	-0.07935
C	-3.12969	-2.34882	-0.86145
C	-3.25099	-1.21578	1.289388
C	-3.67427	-3.50002	-0.27789
H	-2.89424	-2.34955	-1.92988
C	-3.80362	-2.37368	1.867009
H	-3.11367	-0.32572	1.913932
C	-4.01512	-3.52327	1.089032
H	-3.83789	-4.38945	-0.89845
H	-4.0787	-2.36617	2.929036
H	-4.45777	-4.42153	1.534216
C	-3.63123	1.749186	0.128064
C	-3.45789	0.636493	-0.94866
H	-2.89248	1.690729	0.946791
H	-3.48387	2.745561	-0.33283
C	-4.87655	0.042515	-1.11587
C	-3.02561	1.077318	-2.36632
C	-5.04615	1.60563	0.656544
O	-5.25111	-0.68281	-2.03952
C	-5.7476	0.621177	-0.05997
H	-3.00913	0.200965	-3.03544
H	-2.04325	1.573578	-2.40477
H	-3.78338	1.775357	-2.78493
C	-5.68821	2.305922	1.689809
C	-7.09413	0.324742	0.21002
H	-5.15959	3.078151	2.262439
C	-7.03184	2.007042	1.974798
H	-7.60981	-0.44217	-0.37845
C	-7.73361	1.024699	1.24109
H	-7.54652	2.548925	2.777173
H	-8.78119	0.813241	1.484055

$\mathbf{TS_{RE}CS^{ind}}$_conf2

Symbol	X	Y	Z
P	-0.26499	1.493698	0.513028
C	1.139762	0.950236	1.626065
C	-1.40932	2.374219	1.690806
C	0.444318	2.906776	-0.45068
C	2.316513	0.368769	1.104208
C	0.955766	0.996501	3.043148
C	-2.36579	1.603449	2.390945
C	-1.39101	3.771228	1.891694
C	1.421933	3.774849	0.085688
C	-0.06168	3.158849	-1.74264
C	2.599585	0.381446	-0.37344
C	3.307593	-0.17343	2.002633
C	1.902479	0.489725	3.915199
H	0.048768	1.454141	3.447685
C	-3.2551	2.209879	3.292167
H	-2.42231	0.521314	2.222209
C	-2.29209	4.377768	2.782022
H	-0.67429	4.392563	1.346389
C	1.877498	4.875933	-0.65521
H	1.829859	3.585034	1.084632
C	0.396324	4.262541	-2.48013
H	-0.8157	2.492738	-2.17234
C	1.941873	-0.47153	-1.2786
C	3.572103	1.335756	-0.8696
C	3.096422	-0.11081	3.428826
C	4.508827	-0.78791	1.530233
H	1.741785	0.552857	4.99862
C	-3.22199	3.600176	3.489757
H	-3.98486	1.58906	3.82247
H	-2.26423	5.46492	2.920598
C	1.363967	5.123252	-1.93936
H	2.641711	5.536544	-0.23059
H	-0.00881	4.445351	-3.48177
P	0.640451	-1.71724	-0.80442
C	2.133198	-0.27671	-2.68637
C	3.796948	1.459086	-2.28943
C	4.340678	2.172854	-0.00257
C	4.084025	-0.64117	4.310518
H	4.67836	-0.85498	0.451699
C	5.451488	-1.29465	2.412847
H	-3.92353	4.075723	4.184828
H	1.722483	5.982936	-2.51715
C	0.927435	-3.07336	-2.05345
C	1.188283	-2.57163	0.752475
C	3.026584	0.654858	-3.17611
H	1.56438	-0.89292	-3.38912
C	4.769802	2.380944	-2.77456
H	4.184639	2.105671	1.07716
C	5.29028	3.053297	-0.50328
H	3.906101	-0.58176	5.39128
C	5.242822	-1.21953	3.816609
H	6.36464	-1.75932	2.023871
C	2.196204	-3.36753	-2.60629
C	-0.17575	-3.8777	-2.41268
C	2.310302	-3.42393	0.816569
C	0.377573	-2.4215	1.895959
H	3.160318	0.775982	-4.25804
H	4.922559	2.456082	-3.85821
C	5.511647	3.161656	-1.90135
H	5.873214	3.671162	0.189317
H	5.995897	-1.62159	4.503663
C	2.353468	-4.43358	-3.50576
H	3.06423	-2.74999	-2.35085
C	-0.01011	-4.94753	-3.3086
H	-1.15437	-3.67982	-1.95667
C	2.620729	-4.09954	2.005994
H	2.935599	-3.5753	-0.06914
C	0.689511	-3.09636	3.0861
H	-0.49966	-1.76684	1.839923
H	6.262618	3.860952	-2.28573
C	1.249554	-5.2266	-3.86079
H	3.342846	-4.64394	-3.92871
H	-0.87576	-5.56578	-3.57347
C	1.811585	-3.93796	3.142762
H	3.496359	-4.75747	2.043676
H	0.053302	-2.96586	3.969015
H	1.373569	-6.05883	-4.56337
H	2.053821	-4.47111	4.069191
Pd	-1.41858	-0.34882	-0.68855
C	-2.92409	0.677848	-1.6953
C	-3.62037	1.748259	-1.07657
C	-2.59621	0.806025	-3.06973
C	-3.93849	2.910392	-1.79514
H	-3.88309	1.685035	-0.01444
C	-2.92047	1.973324	-3.78672
H	-2.08125	-0.01089	-3.58724
C	-3.59518	3.030411	-3.15539
H	-4.44873	3.73529	-1.28303
H	-2.65091	2.044899	-4.84778
H	-3.85888	3.934601	-3.71543
C	-4.98639	-0.84118	-0.72511
C	-3.61787	-1.27284	-1.31547
H	-5.34798	0.14458	-1.05853
H	-5.73588	-1.58831	-1.0679
C	-3.06059	-2.18819	-0.2223
C	-3.68064	-1.93647	-2.68118
C	-4.83237	-0.97707	0.776786
O	-2.45282	-3.26848	-0.37802
C	-3.71023	-1.77909	1.067011
H	-4.29714	-2.8549	-2.6098
H	-2.68249	-2.24522	-3.03142
H	-4.13434	-1.27521	-3.4377
C	-5.66629	-0.53495	1.813204
C	-3.42769	-2.20035	2.375946
H	-6.5485	0.082178	1.603835
C	-5.36691	-0.9222	3.133462
H	-2.58265	-2.87126	2.565838
C	-4.26051	-1.7522	3.414077
H	-6.01526	-0.59157	3.953666
H	-4.06089	-2.05666	4.447961

$\mathbf{TS_{RE}CS^{ind}}$_conf3

Symbol	X	Y	Z
P	0.589043	1.770855	-0.25732
C	1.973697	1.276864	0.902496
C	0.417785	3.597137	0.027105
C	1.317762	1.666872	-1.95525
C	2.670255	0.056448	0.770071
C	2.24029	2.104853	2.039494
C	-0.15935	4.032002	1.245166
C	0.748149	4.571809	-0.94061
C	2.560529	2.261015	-2.27126
C	0.588911	1.012526	-2.97151
C	2.600889	-0.81862	-0.45281
C	3.611114	-0.34412	1.799022
C	3.135071	1.735748	3.023677
H	1.743586	3.073238	2.124344
C	-0.31626	5.399272	1.519762
H	-0.50199	3.294269	1.978583
C	0.561245	5.938262	-0.6752
H	1.156707	4.266529	-1.90797
C	3.051218	2.222752	-3.58484
H	3.144785	2.753566	-1.48616
C	1.086519	0.973752	-4.28488
H	-0.36044	0.513916	-2.73173
C	1.540043	-1.70357	-0.74071
C	3.764218	-0.78807	-1.32095
C	3.832872	0.49987	2.9466
C	4.343723	-1.57128	1.732333
H	3.323414	2.403604	3.873338
C	0.047086	6.358814	0.561767
H	-0.75028	5.70964	2.476685
H	0.828364	6.676169	-1.44047
C	2.312377	1.583302	-4.59467
H	4.017486	2.684035	-3.81711
H	0.511949	0.459345	-5.0631
P	-0.10811	-1.66722	0.162357
C	1.654351	-2.56853	-1.87494
C	3.840473	-1.66733	-2.46077
C	4.862144	0.102721	-1.10216
C	4.751885	0.09746	3.959608
H	4.196289	-2.23179	0.873911
C	5.233251	-1.938	2.732816
H	-0.08578	7.426323	0.770981
H	2.698625	1.553345	-5.61992
C	-0.78991	-3.36979	-0.18663

C	0.317717	-1.77635	1.960392
C	2.761098	-2.55967	-2.70022
H	0.850846	-3.27518	-2.09147
C	4.984899	-1.63421	-3.31079
H	4.829816	0.790521	-0.2533
C	5.964156	0.114004	-1.946
H	4.901746	0.76007	4.820864
C	5.443849	-1.09931	3.859552
H	5.779347	-2.88468	2.650266
C	-0.80507	-4.41508	0.762753
C	-1.32352	-3.61903	-1.47479
C	1.305454	-2.65619	2.455724
C	-0.42289	-0.99807	2.874207
H	2.815825	-3.24931	-3.55145
H	5.016957	-2.3164	-4.16914
C	6.03333	-0.76312	-3.06075
H	6.78849	0.808931	-1.74898
H	6.150215	-1.39833	4.64209
C	-1.29341	-5.68674	0.420312
H	-0.43546	-4.24372	1.777528
C	-1.78341	-4.90162	-1.81961
H	-1.44008	-2.79299	-2.18721
C	1.539008	-2.76028	3.835166
H	1.896812	-3.25848	1.75774
C	-0.1887	-1.1034	4.25487
H	-1.17912	-0.30073	2.49245
H	6.909693	-0.74721	-3.71844
C	-1.76428	-5.94222	-0.87697
H	-1.29613	-6.48252	1.174317
H	-2.18099	-5.07566	-2.82638
C	0.790114	-1.98642	4.737442
H	2.31618	-3.4387	4.203777
H	-0.77135	-0.4907	4.95212
H	-2.12933	-6.94006	-1.14602
H	0.975214	-2.06777	5.814637
Pd	-1.44007	0.454644	-0.17897
C	-2.85869	1.792242	0.503451
C	-3.46557	1.190893	1.639186
C	-2.93362	3.194849	0.370844
C	-4.06368	1.978928	2.638107
H	-3.50141	0.098275	1.727528
C	-3.58134	3.973318	1.346661
H	-2.4842	3.692613	-0.49386
C	-4.13803	3.374875	2.489566
H	-4.50531	1.492054	3.516433
H	-3.63197	5.061015	1.215062
H	-4.63972	3.98691	3.247878
C	-5.0485	0.964556	-0.93858
C	-3.59045	0.89331	-1.42043
H	-5.26303	1.752442	-0.19933
H	-5.67455	1.189425	-1.83195
C	-3.33834	-0.55886	-1.71254
C	-3.1977	1.882513	-2.50004
C	-5.36096	-0.43842	-0.46926
O	-2.56309	-1.00431	-2.59638
C	-4.38443	-1.33553	-0.95594
H	-3.5157	2.906665	-2.24243
H	-3.69936	1.601858	-3.44858
H	-2.1145	1.890819	-2.69653
C	-6.46312	-0.90512	0.258505
C	-4.53653	-2.72034	-0.77177
H	-7.21934	-0.20738	0.638486
C	-6.59187	-2.28803	0.47916
H	-3.80176	-3.41812	-1.17959
C	-5.64026	-3.18781	-0.04167
H	-7.44709	-2.67071	1.048574
H	-5.76315	-4.26412	0.125808

TS$_{RE}$CS^{ind}_conf4

Symbol	X	Y	Z
P	0.001678	1.470276	0.838266
C	1.401124	0.542946	1.667025
C	-0.93995	2.167297	2.285106
C	0.860407	2.962365	0.153601
C	2.387748	-0.0929	0.886044
C	1.435729	0.400403	3.088055
C	-1.73876	1.288004	3.054389
C	-0.9498	3.540609	2.608671
C	1.888993	3.614667	0.87156
C	0.46337	3.473973	-1.09802
C	2.449024	0.115259	-0.60154
C	3.399703	-0.8979	1.524123
C	2.40855	-0.3584	3.714804
H	0.683391	0.909383	3.696327
C	-2.49535	1.764384	4.135219
H	-1.77393	0.221536	2.801692
C	-1.72457	4.018617	3.680087
H	-0.35306	4.243282	2.01994
C	2.498618	4.764757	0.3491
H	2.213835	3.218495	1.840394
C	1.078575	4.624809	-1.61776
H	-0.32592	2.972243	-1.66529
C	1.540975	-0.49456	-1.48382
C	3.478077	0.991897	-1.12452
C	3.405993	-1.03552	2.960609
C	4.407506	-1.58538	0.779271
H	2.42051	-0.44214	4.808572
C	-2.49334	3.134281	4.451148
H	-3.09632	1.063549	4.725999
H	-1.72265	5.090281	3.910247
C	2.092691	5.2731	-0.89667
H	3.296625	5.260888	0.913295
H	0.758281	5.012385	-2.59114
P	0.107915	-1.58188	-0.9809
C	1.580915	-0.154	-2.87633
C	3.533479	1.266506	-2.53985
C	4.464307	1.603195	-0.29026
C	4.409912	-1.83307	3.585506
H	4.411211	-1.49627	-0.31107
C	5.370304	-2.35489	1.41576
H	-3.09314	3.508206	5.288507
H	2.571946	6.170288	-1.30493
C	0.109421	-2.80548	-2.39015
C	0.643196	-2.67668	0.425758
C	2.543096	0.694284	-3.38735
H	0.83945	-0.59047	-3.55245
C	4.565011	2.102421	-3.05823
H	4.434303	1.424755	0.787749
C	5.46122	2.407926	-0.82464
H	4.400489	-1.92428	4.67854
C	5.376587	-2.48016	2.831681
H	6.133229	-2.87078	0.82179
C	1.308149	-3.26797	-2.98644
C	-1.12091	-3.32441	-2.84455
C	1.65558	-3.65506	0.319511
C	-0.07919	-2.5752	1.63208
H	2.559216	0.927374	-4.45904
H	4.588464	2.294347	-4.13791
C	5.518903	2.659681	-2.22067
H	6.209241	2.855611	-0.16045
H	6.144054	-3.08876	3.32296
C	1.276143	-4.24283	-3.994
H	2.272374	-2.84624	-2.68188
C	-1.14535	-4.30161	-3.85642
H	-2.07151	-2.94853	-2.44403
C	1.947759	-4.49668	1.402253
H	2.211065	-3.77368	-0.61505
C	0.21555	-3.41595	2.716993
H	-0.88705	-1.83851	1.708147
H	6.311217	3.296078	-2.63056
C	0.046873	-4.76824	-4.42822
H	2.214269	-4.58792	-4.44434
H	-2.11138	-4.68705	-4.20205
C	1.230318	-4.37803	2.60458
H	2.739169	-5.24842	1.305517
H	-0.36117	-3.32402	3.643727
H	0.022347	-5.52839	-5.21769
H	1.458188	-5.04063	3.447471
Pd	-1.57175	0.18099	-0.54875
C	-2.70862	1.796273	-1.15719
C	-3.05235	2.936978	-0.40033
C	-2.56435	1.936247	-2.56202
C	-3.19868	4.190016	-1.02141
H	-3.18411	2.86301	0.684856
C	-2.6885	3.195388	-3.17449
H	-2.37008	1.050566	-3.17936
C	-3.00923	4.32938	-2.40733
H	-3.45186	5.065943	-0.41126
H	-2.55688	3.282859	-4.26018

H	-3.12892	5.307451	-2.88692
C	-4.38212	0.124782	0.794748
C	-4.05101	0.075508	-0.70828
H	-3.88741	0.944854	1.342898
H	-5.47396	0.285519	0.927142
C	-3.70689	-1.35135	-1.03694
C	-5.10973	0.634652	-1.64894
C	-4.00263	-1.24859	1.324205
O	-3.74135	-1.87479	-2.17186
C	-3.61707	-2.09729	0.265063
H	-6.04634	0.058148	-1.49381
H	-4.8142	0.493402	-2.70003
H	-5.3222	1.700779	-1.46927
C	-4.07274	-1.74816	2.632199
C	-3.34907	-3.45792	0.482163
H	-4.38461	-1.10362	3.463887
C	-3.78037	-3.10758	2.859327
H	-3.08486	-4.10604	-0.36056
C	-3.42938	-3.95805	1.791387
H	-3.84795	-3.51366	3.875572
H	-3.21894	-5.01552	1.987005

TS$_{RE}$CS^{ind}_conf5

Symbol	X	Y	Z
P	0.540213	1.44595	0.814921
C	1.775267	0.219688	1.489059
C	-0.13186	2.224124	2.363815
C	1.651219	2.764291	0.133526
C	2.474462	-0.6323	0.614094
C	2.00963	0.134334	2.897129
C	-0.99834	1.452689	3.174883
C	0.120065	3.563025	2.728074
C	2.84177	3.140738	0.797105
C	1.294566	3.416544	-1.06373
C	2.335388	-0.56551	-0.88481
C	3.425192	-1.58095	1.154369
C	2.914872	-0.76643	3.425742
H	1.468812	0.80415	3.570556
C	-1.57392	1.99935	4.330967
H	-1.22083	0.416026	2.894856
C	-0.47296	4.114099	3.878034
H	0.778033	4.181251	2.110077
C	3.653886	4.157677	0.273673
H	3.131698	2.637284	1.72658
C	2.113299	4.431912	-1.58561
H	0.374844	3.133284	-1.58462
C	1.238387	-1.11268	-1.5854
C	3.447268	-0.00437	-1.62594
C	3.641626	-1.64859	2.579601
C	4.166825	-2.4817	0.326736
H	3.085103	-0.80609	4.508706
C	-1.31609	3.335894	4.684962
H	-2.2327	1.382156	4.952538
H	-0.2704	5.158751	4.14098
C	3.290488	4.805365	-0.9197
H	4.573796	4.442678	0.797381
H	1.823025	4.930845	-2.51676
P	-0.38623	-1.57192	-0.77128
C	1.269838	-1.13707	-3.01534
C	3.44437	-0.03743	-3.06862
C	4.573414	0.593953	-0.97903
C	4.57435	-2.58833	3.109728
H	4.019535	-2.45169	-0.75629
C	5.065605	-3.38775	0.871715
H	-1.7745	3.766631	5.58221
H	3.927548	5.597593	-1.32947
C	-1.18182	-2.70776	-2.01028
C	0.012111	-2.7247	0.616689
C	2.335239	-0.62824	-3.73301
H	0.432823	-1.58485	-3.55709
C	4.547796	0.503508	-3.79226
H	4.592635	0.646448	0.112487
C	5.630418	1.11707	-1.70972
H	4.723197	-2.61984	4.195992
C	5.276579	-3.44399	2.275827
H	5.620254	-4.06398	0.211353
C	-1.14388	-4.11693	-1.91762
C	-1.888	-2.11656	-3.08227
C	1.027437	-3.70309	0.537608
C	-0.79795	-2.66898	1.769694
H	2.334129	-0.67865	-4.8288
H	4.523475	0.462695	-4.88808
C	5.625237	1.068972	-3.12935
H	6.476549	1.574704	-1.18498
H	5.991989	-4.16092	2.694041
C	-1.76906	-4.91124	-2.89106
H	-0.62996	-4.59908	-1.08084
C	-2.50106	-2.9145	-4.06135
H	-1.97975	-1.02796	-3.13792
C	1.230029	-4.60419	1.59397
H	1.666733	-3.75445	-0.35022
C	-0.5962	-3.57463	2.823674
H	-1.59901	-1.92373	1.832451
H	6.468166	1.482041	-3.69464
C	-2.44168	-4.31301	-3.9701
H	-1.72856	-6.00333	-2.8042
H	-3.05068	-2.43487	-4.87827
C	0.417356	-4.54194	2.738598
H	2.030002	-5.34954	1.52464
H	-1.23827	-3.52273	3.709968
H	-2.93062	-4.93612	-4.72777
H	0.576733	-5.24649	3.56299
Pd	-1.40401	0.618746	-0.39251
C	-2.18658	2.482668	-0.86772
C	-2.22043	3.613902	-0.02123
C	-2.09876	2.695687	-2.2687
C	-2.11581	4.910851	-0.5522
H	-2.31467	3.487309	1.063189
C	-1.97459	3.993704	-2.79367
H	-2.1531	1.839231	-2.95158
C	-1.98181	5.109455	-1.93792
H	-2.13151	5.772939	0.126339
H	-1.89404	4.131926	-3.87917
H	-1.90579	6.123105	-2.34744
C	-4.05782	1.01399	1.104709
C	-3.8396	1.104788	-0.4203
H	-3.29511	1.545717	1.701001
H	-5.03109	1.478212	1.3723
C	-3.90891	-0.29978	-0.96435
C	-4.81005	2.018305	-1.16468
C	-4.10156	-0.4772	1.405239
O	-4.0967	-0.6179	-2.15487
C	-4.01259	-1.22663	0.213378
H	-5.84097	1.655547	-0.96571
H	-4.6472	1.956265	-2.25161
H	-4.74221	3.070535	-0.84463
C	-4.29036	-1.13225	2.630325
C	-4.15262	-2.62301	0.212651
H	-4.37468	-0.56426	3.565634
C	-4.41416	-2.53638	2.639514
H	-4.1094	-3.17345	-0.73314
C	-4.35098	-3.2753	1.440451
H	-4.5811	-3.05871	3.589094
H	-4.46206	-4.36531	1.470248

TS$_{RE}$CS^{ind}_conf6

Symbol	X	Y	Z
P	-0.38952	-1.6796	0.081859
C	-1.96982	-1.40447	1.053214
C	0.491649	-2.97839	1.071804
C	-1.03129	-2.60928	-1.38974
C	-2.91561	-0.46294	0.603957
C	-2.21718	-2.1236	2.263257
C	0.941705	-2.63406	2.370381
C	0.865107	-4.23278	0.542107
C	-1.85567	-3.74921	-1.25477
C	-0.71273	-2.14249	-2.68104
C	-2.78649	0.236687	-0.72013
C	-4.09744	-0.2038	1.395328
C	-3.35458	-1.90264	3.017989
H	-1.49654	-2.8751	2.595827
C	1.712018	-3.52944	3.125617
H	0.679805	-1.65638	2.792324
C	1.654628	-5.12024	1.293913
H	0.541038	-4.52014	-0.46309
C	-2.32714	-4.42143	-2.3919

H	-2.12866	-4.10991	-0.25643
C	-1.1903	-2.81408	-3.81871
H	-0.0964	-1.24258	-2.78696
C	-1.88225	1.292363	-0.94642
C	-3.67568	-0.18512	-1.7862
C	-4.31549	-0.93176	2.621619
C	-5.06816	0.776263	1.018676
H	-3.52602	-2.47788	3.936149
C	2.076379	-4.77635	2.587638
H	2.035934	-3.24898	4.134544
H	1.936642	-6.08768	0.862828
C	-1.99177	-3.95673	-3.67562
H	-2.96235	-5.30719	-2.27619
H	-0.93784	-2.43904	-4.81665
P	-0.48649	1.783346	0.167633
C	-1.8322	1.905231	-2.24416
C	-3.63494	0.483481	-3.06428
C	-4.60774	-1.25723	-1.62921
C	-5.48022	-0.66716	3.401168
H	-4.91648	1.34462	0.096504
C	-6.18828	1.017096	1.801531
H	2.687165	-5.47279	3.172769
H	-2.36386	-4.48155	-4.56275
C	-0.38502	3.620764	-0.11558
C	-1.0194	1.784743	1.946311
C	-2.6854	1.527247	-3.26028
H	-1.10545	2.70315	-2.42479
C	-4.52918	0.086656	-4.10007
H	-4.64053	-1.79912	-0.68044
C	-5.46487	-1.62413	-2.65796
H	-5.62884	-1.23477	4.327927
C	-6.40262	0.28763	3.002345
H	-6.91517	1.775669	1.489844
C	-1.52587	4.433031	-0.31418
C	0.884668	4.229097	-0.05564
C	-2.21213	2.374159	2.415174
C	-0.09407	1.278564	2.883686
H	-2.63805	2.028794	-4.23472
H	-4.48345	0.61562	-5.05998
C	-5.43561	-0.9444	-3.90368
H	-6.16933	-2.44966	-2.50566
H	-7.29359	0.483005	3.609522
C	-1.39542	5.822864	-0.44638
H	-2.51968	3.976846	-0.38338
C	1.010883	5.622821	-0.18524
H	1.779239	3.615944	0.087736
C	-2.47757	2.441005	3.790744
H	-2.9405	2.780611	1.706523
C	-0.3545	1.356473	4.2618
H	0.838912	0.83386	2.512563
H	-6.12058	-1.23977	-4.70632
C	-0.12503	6.422038	-0.38
H	-2.28825	6.439739	-0.60223
H	2.007746	6.075277	-0.14402
C	-1.54889	1.936	4.716959
H	-3.41321	2.891596	4.140222
H	0.378252	0.967888	4.978561
H	-0.02456	7.508488	-0.4851
H	-1.75644	1.998029	5.791338
Pd	1.233192	0.186572	-0.33065
C	2.934299	1.28437	-0.83034
C	2.906964	1.926264	-2.097
C	3.697825	1.891817	0.203582
C	3.571431	3.144414	-2.3013
H	2.36486	1.46556	-2.93016
C	4.374678	3.103499	-0.01338
H	3.773194	1.406159	1.183721
C	4.315793	3.739919	-1.26642
H	3.519563	3.625905	-3.2856
H	4.956733	3.547841	0.803773
H	4.85615	4.677396	-1.43952
C	3.598577	-1.44265	0.429204
C	3.37045	-0.75614	-0.95071
H	2.981858	-1.01189	1.242018
H	3.310895	-2.51051	0.376302
C	4.792905	-0.35698	-1.42156
C	2.791458	-1.63902	-2.07522
C	5.074269	-1.28319	0.742859
O	5.104541	0.001751	-2.55722
C	5.74413	-0.6459	-0.31474
H	3.541173	-2.39603	-2.39066
H	2.573302	-1.03059	-2.96908
H	1.876894	-2.17176	-1.77672
C	5.790706	-1.68263	1.882301
C	7.12889	-0.41017	-0.28105
H	5.285926	-2.18456	2.716867
C	7.174224	-1.43904	1.930257
H	7.617365	0.078278	-1.13153
C	7.842088	-0.80774	0.857171
H	7.747001	-1.74893	2.812514
H	8.922056	-0.63172	0.920474

TS$_{RE}$CS^{ind}_conf7

Symbol	X	Y	Z
P	0.806485	-1.68563	-0.59613
C	2.183631	-0.54601	-1.15452
C	0.466963	-2.71223	-2.10942
C	1.73141	-2.8951	0.475608
C	2.646947	0.478381	-0.30585
C	2.77022	-0.70197	-2.44975
C	0.021721	-2.07813	-3.29472
C	0.529421	-4.12111	-2.08386
C	3.080609	-3.22892	0.221181
C	1.05683	-3.5229	1.544239
C	2.18256	0.650324	1.119514
C	3.700232	1.361352	-0.76407
C	3.768834	0.139577	-2.90339
H	2.427264	-1.51588	-3.0938
C	-0.32399	-2.8311	-4.42671
H	-0.04862	-0.98467	-3.33056
C	0.163824	-4.87382	-3.21302
H	0.857261	-4.63476	-1.17558
C	3.744357	-4.16777	1.025096
H	3.613022	-2.75674	-0.61201
C	1.726879	-4.4642	2.344165
H	-0.00539	-3.31006	1.710119
C	0.984001	1.309112	1.475809
C	3.094558	0.221926	2.161043
C	4.255097	1.196054	-2.0857
C	4.22906	2.412331	0.049815
H	4.205408	-0.00851	-3.89886
C	-0.25761	-4.23509	-4.38891
H	-0.65123	-2.319	-5.33912
H	0.213692	-5.96788	-3.16906
C	3.069471	-4.78579	2.091127
H	4.791001	-4.41845	0.816087
H	1.190797	-4.95082	3.167204
P	-0.38981	1.638109	0.261684
C	0.705219	1.549558	2.859373
C	2.780746	0.473744	3.547398
C	4.325002	-0.44805	1.873553
C	5.285185	2.074005	-2.53645
H	3.83359	2.551961	1.059674
C	5.233294	3.249871	-0.41466
H	-0.53632	-4.82456	-5.26963
H	3.58887	-5.51902	2.719012
C	-1.51412	2.808489	1.169406
C	0.290721	2.714763	-1.07487
C	1.57157	1.153476	3.859164
H	-0.21432	2.072568	3.133198
C	3.686289	0.058344	4.567204
H	4.585924	-0.6565	0.833135
C	5.186681	-0.84134	2.887343
H	5.689513	1.929224	-3.54575
C	5.768665	3.084178	-1.7204
H	5.619547	4.044114	0.234312
C	-1.49314	4.207277	0.986427
C	-2.46686	2.254567	2.055934
C	1.293599	3.683196	-0.85286
C	-0.28643	2.603785	-2.35879
H	1.335998	1.369249	4.908661
H	3.424174	0.262986	5.612486
C	4.870787	-0.58652	4.248265
H	6.120063	-1.35637	2.633938
H	6.562671	3.750568	-2.07571
C	-2.39077	5.031377	1.685134
H	-0.77854	4.658201	0.290935
C	-3.35195	3.08163	2.764744
H	-2.51454	1.166556	2.188304

C	1.719977	4.516763	-1.89835
H	1.746522	3.78041	0.139187
C	0.135942	3.446593	-3.3993
H	-1.07334	1.862205	-2.53876
H	5.560652	-0.90106	5.039355
C	-3.31843	4.473688	2.579132
H	-2.36165	6.115898	1.527914
H	-4.0761	2.628069	3.450121
C	1.140555	4.401219	-3.17288
H	2.510875	5.252709	-1.71596
H	-0.32308	3.353339	-4.39008
H	-4.0172	5.119861	3.122766
H	1.473559	5.05402	-3.98792
Pd	-1.42843	-0.45557	-0.33432
C	-3.13864	0.108781	-1.38893
C	-3.92895	1.196927	-0.93872
C	-2.9788	-0.05672	-2.7886
C	-4.48606	2.108967	-1.84805
H	-4.08461	1.353944	0.133961
C	-3.54088	0.860364	-3.69616
H	-2.41672	-0.9132	-3.17359
C	-4.29505	1.950247	-3.233
H	-5.06669	2.957989	-1.46689
H	-3.39693	0.706886	-4.77316
H	-4.73996	2.6597	-3.93975
C	-4.84811	-1.38229	0.179409
C	-3.51383	-1.78331	-0.4918
H	-5.40069	-0.57472	-0.3281
H	-5.50375	-2.28075	0.161404
C	-2.6731	-2.27738	0.674979
C	-3.59795	-2.75525	-1.65141
C	-4.48359	-1.07859	1.620608
O	-1.89759	-3.26052	0.674193
C	-3.21083	-1.61262	1.911657
H	-4.20955	-2.35761	-2.47835
H	-4.06197	-3.69863	-1.2998
H	-2.5973	-3.01309	-2.03396
C	-5.23252	-0.45912	2.630735
C	-2.67993	-1.57714	3.210404
H	-6.22748	-0.04678	2.422782
C	-4.6932	-0.39487	3.93031
H	-1.70201	-2.02432	3.419833
C	-3.42918	-0.95116	4.219717
H	-5.27058	0.079548	4.732695
H	-3.0353	-0.89782	5.241148

TS$_{RE}$CS^{ind}_conf8

Symbol	X	Y	Z
P	-0.26493	1.493679	0.513167
C	1.13964	0.949949	1.626294
C	-1.40933	2.374205	1.690867
C	0.444735	2.906692	-0.45037
C	2.316344	0.3684	1.104426
C	0.955628	0.996169	3.043374
C	-2.36542	1.603371	2.39147
C	-1.3916	3.771317	1.891092
C	1.422109	3.774828	0.086346
C	-0.06066	3.158587	-1.74259
C	2.599509	0.381344	-0.3732
C	3.307314	-0.17403	2.002822
C	1.902271	0.489223	3.915407
H	0.048689	1.453917	3.447924
C	-3.25486	2.209855	3.292525
H	-2.4216	0.521146	2.223205
C	-2.29283	4.377902	2.781245
H	-0.67519	4.392694	1.345428
C	1.878041	4.87579	-0.6545
H	1.829562	3.585158	1.085512
C	0.397714	4.262159	-2.48003
H	-0.81452	2.492434	-2.17252
C	1.941843	-0.47144	-1.27858
C	3.572077	1.335743	-0.86909
C	3.09613	-0.11147	3.429016
C	4.508457	-0.78866	1.530387
H	1.741588	0.552348	4.99883
C	-3.2223	3.60026	3.48947
H	-3.9843	1.588985	3.823201
H	-2.26539	5.465131	2.9193
C	1.365126	5.122923	-1.93893
H	2.64205	5.536468	-0.22962
H	-0.00694	4.444836	-3.48189
P	0.640299	-1.71716	-0.80473
C	2.133285	-0.27634	-2.6863
C	3.797024	1.45936	-2.28889
C	4.340627	2.172629	-0.00185
C	4.083646	-0.64203	4.310682
H	4.67798	-0.85567	0.451847
C	5.451024	-1.29561	2.412977
H	-3.92395	4.075838	4.184407
H	1.723944	5.982505	-2.51669
C	0.927116	-3.07295	-2.05414
C	1.187978	-2.57195	0.751996
C	3.026717	0.655319	-3.17578
H	1.564515	-0.89241	-3.38921
C	4.769941	2.381285	-2.77377
H	4.184526	2.105218	1.077863
C	5.290296	3.053139	-0.50231
H	3.905728	-0.58266	5.391448
C	5.242353	-1.22055	3.816742
H	6.364102	-1.76041	2.023983
C	2.195864	-3.36723	-2.60697
C	-0.17624	-3.87687	-2.41377
C	2.309668	-3.4247	0.815859
C	0.377432	-2.42168	1.895577
H	3.160543	0.776647	-4.25768
H	4.922776	2.456636	-3.85739
C	5.511758	3.161785	-1.90033
H	5.87321	3.670837	0.190457
H	5.995358	-1.62278	4.503777
C	2.352933	-4.43296	-3.50684
H	3.064016	-2.74998	-2.35123
C	-0.01081	-4.94638	-3.3101
H	-1.15482	-3.67896	-1.95766
C	2.619957	-4.10058	2.005165
H	2.934817	-3.57619	-0.06994
C	0.689237	-3.0968	3.085598
H	-0.49959	-1.7667	1.839716
H	6.262783	3.861132	-2.28452
C	1.24884	-5.22555	-3.86229
H	3.342296	-4.64343	-3.92977
H	-0.87659	-5.5643	-3.5753
C	1.810997	-3.93884	3.142038
H	3.495325	-4.75888	2.042664
H	0.05316	-2.96618	3.96859
H	1.372702	-6.05753	-4.56519
H	2.053121	-4.4722	4.068372
Pd	-1.41851	-0.34847	-0.68875
C	-2.92406	0.678318	-1.69522
C	-3.62021	1.748776	-1.07645
C	-2.5963	0.806439	-3.06969
C	-3.93828	2.910939	-1.795
H	-3.88281	1.685591	-0.01428
C	-2.92047	1.973781	-3.78665
H	-2.08153	-0.01057	-3.58726
C	-3.59504	3.030935	-3.15526
H	-4.44842	3.735879	-1.28285
H	-2.65099	2.04533	-4.84773
H	-3.85867	3.935158	-3.71528
C	-4.98626	-0.84057	-0.72438
C	-3.61803	-1.27243	-1.31526
H	-5.34759	0.145462	-1.05729
H	-5.73612	-1.58726	-1.06732
C	-3.06053	-2.18811	-0.2225
C	-3.68136	-1.93582	-2.68107
C	-4.8319	-0.97708	0.777422
O	-2.45288	-3.2684	-0.3787
C	-3.70977	-1.77929	1.067103
H	-4.29832	-2.85395	-2.60973
H	-2.68341	-2.24501	-3.03148
H	-4.13484	-1.27421	-3.43742
C	-5.66549	-0.53517	1.814198
C	-3.42686	-2.20089	2.375853
H	-6.5477	0.082103	1.60525
C	-5.36576	-0.92278	3.134268
H	-2.58182	-2.87191	2.565325
C	-4.25932	-1.75293	3.414347
H	-6.01383	-0.5923	3.954749
H	-4.05942	-2.05765	4.448098

$\mathbf{TS_{RE}CR^{dif}}$_conf1

Symbol	X	Y	Z
C	4.712874	2.582468	-2.07821
C	4.938651	3.727395	-1.29396
C	4.476807	3.754587	0.033022
C	3.781727	2.657413	0.570383
C	3.545369	1.502033	-0.20696
C	4.048806	1.469497	-1.53362
H	5.090688	2.535471	-3.10731
H	5.483656	4.583627	-1.70728
H	4.642416	4.643367	0.65404
H	3.419323	2.718471	1.603428
H	3.974847	0.541114	-2.11433
Pd	1.91331	0.220378	-0.15466
C	4.191907	-0.35698	0.821669
C	3.894282	-1.66886	0.152882
C	3.845923	-0.2649	2.308209
C	5.65208	0.028477	0.557509
O	4.349203	-1.94465	-0.98449
C	3.290591	-2.75841	1.010331
C	2.585974	-1.04795	2.709057
H	3.767009	0.795805	2.613819
H	4.708556	-0.67294	2.879755
H	6.296393	-0.71413	1.074205
H	5.89059	-0.0152	-0.51377
H	5.894336	1.030364	0.947887
C	2.710051	-2.48649	2.27569
C	3.43582	-4.09602	0.58565
H	2.405419	-0.97631	3.797529
H	1.704487	-0.59236	2.201701
C	2.273132	-3.55692	3.076353
H	3.917178	-4.27459	-0.37989
C	2.990988	-5.1544	1.387703
H	1.833788	-3.34371	4.059214
C	2.405602	-4.88502	2.637536
H	3.107457	-6.1887	1.045005
H	2.068134	-5.70858	3.277858
P	0.126476	1.89657	0.226225
C	-1.5562	1.731046	-0.57383
C	0.583187	3.629156	-0.25336
C	-0.31582	2.090548	2.020843
C	-2.35894	0.560892	-0.35518
C	-2.0231	2.746356	-1.4405
C	1.260757	3.819214	-1.47699
C	0.253048	4.754638	0.530915
C	-1.62155	2.39451	2.463107
C	0.71344	1.97021	2.979415
C	-2.03714	-0.58309	0.54935
C	-3.58152	0.541141	-1.02647
C	-3.25843	2.682777	-2.11825
H	-1.40419	3.632492	-1.59612
C	1.581093	5.111587	-1.91861
H	1.555902	2.949799	-2.07342
C	0.586576	6.046109	0.091903
H	-0.26842	4.62328	1.484531
C	-1.89037	2.559695	3.83185
H	-2.43541	2.496206	1.738672
C	0.447835	2.1534	4.344831
H	1.728301	1.727936	2.648157
C	-1.00753	-1.5622	0.357473
C	-2.84882	-0.78754	1.669049
C	-4.01875	1.548677	-1.88443
H	-3.59087	3.480847	-2.78689
C	1.245171	6.2279	-1.13506
H	2.111632	5.242895	-2.86784
H	0.326895	6.912407	0.711212
C	-0.85737	2.442765	4.775452
H	-2.91362	2.773717	4.157761
H	1.261069	2.059925	5.073471
P	0.30156	-1.41948	-0.94497
C	-0.83366	-2.60114	1.304926
C	-2.67439	-1.82689	2.58054
H	1.50358	7.236978	-1.47587
H	-1.06953	2.572489	5.842597
C	0.571365	-3.17504	-1.504
C	-0.50134	-0.81844	-2.51057
C	-1.66022	-2.76174	2.435078
H	-0.02004	-3.31822	1.161632
C	-0.42891	-4.17117	-1.45901
C	1.790652	-3.45229	-2.15796
C	-1.73801	-1.31239	-2.97638
C	0.236682	0.063109	-3.32577
H	-1.50669	-3.5742	3.149523
C	-0.21221	-5.42119	-2.05822
H	-1.3804	-3.97534	-0.95325
C	1.995313	-4.70149	-2.76788
H	2.598401	-2.71101	-2.1583
C	-2.2358	-0.91368	-4.22535
H	-2.31935	-2.00786	-2.36097
C	-0.25346	0.446831	-4.58564
H	1.202194	0.440857	-2.96485
C	0.997689	-5.68698	-2.72155
H	-0.99629	-6.18601	-2.01356
H	2.94805	-4.90346	-3.27059
C	-1.49158	-0.03699	-5.03483
H	-3.20875	-1.28676	-4.56349
H	0.333722	1.126527	-5.21373
H	1.161217	-6.66112	-3.19661
H	-1.87795	0.265104	-6.01488
O	-5.24837	1.190771	-2.41971
O	-4.52987	-0.47706	-1.013
O	-3.93167	-0.014	2.075308
O	-3.63879	-1.72822	3.575523
C	-5.54349	-0.08269	-1.9024
C	-4.42113	-0.6071	3.251811
F	-5.64514	-0.98256	-2.91535
F	-6.73822	-0.06491	-1.27282
F	-5.71371	-0.97182	3.086479
F	-4.39432	0.288819	4.269696

$\mathbf{TS_{RE}CR^{dif}}$_conf2

Symbol	X	Y	Z
C	-2.71949	4.807994	-0.79149
C	-2.91176	4.724405	-2.18225
C	-3.05742	3.460207	-2.77428
C	-3.01933	2.296167	-1.98312
C	-2.79014	2.361849	-0.58372
C	-2.66225	3.64669	-0.00447
H	-2.60042	5.786623	-0.3107
H	-2.96086	5.6335	-2.7922
H	-3.21992	3.370839	-3.85565
H	-3.16702	1.325952	-2.46968
H	-2.48974	3.747499	1.070773
Pd	-1.66386	0.670478	-0.02187
P	-0.56976	-1.41192	-0.62044
C	1.030667	-1.05019	-1.5274
C	-1.47492	-2.49481	-1.83045
C	-0.019	-2.58107	0.70293
C	2.14453	-0.48645	-0.81549
C	1.13065	-1.26359	-2.91993
C	-2.04764	-1.88135	-2.97012
C	-1.65402	-3.88158	-1.65085
C	0.900648	-3.62556	0.456254
C	-0.53973	-2.40344	2.001686
C	2.265301	-0.30559	0.665018
C	3.265457	-0.19425	-1.59434
C	2.278192	-0.94504	-3.67783
H	0.288673	-1.71596	-3.44831
C	-2.74116	-2.64052	-3.92439
H	-1.94052	-0.79792	-3.10937
C	-2.368	-4.63654	-2.5963
H	-1.2423	-4.37634	-0.7666
C	1.282022	-4.49015	1.493326
H	1.327689	-3.75429	-0.54492
C	-0.15147	-3.27196	3.036025
H	-1.24213	-1.58316	2.202149
C	1.606443	0.686744	1.464888
C	3.173438	-1.11508	1.348125
C	3.337425	-0.40094	-2.97158
H	2.327616	-1.12999	-4.75394
C	-2.90384	-4.02409	-3.73902
H	-3.16418	-2.14809	-4.80754
H	-2.50212	-5.71222	-2.43512
C	0.753493	-4.31521	2.784484
H	2.002591	-5.29106	1.296735

H	-0.5567	-3.12629	4.043902
P	0.340245	1.806767	0.685207
C	1.874939	0.766933	2.849945
C	3.424718	-1.02163	2.717187
H	-3.45355	-4.61784	-4.47801
H	1.056973	-4.98814	3.59468
C	-0.01779	2.997704	2.061856
C	1.337412	2.799839	-0.51568
C	2.784848	-0.08473	3.511997
H	1.363599	1.528686	3.443065
C	0.619984	4.249153	2.187065
C	-0.99006	2.616415	3.017396
C	2.709996	3.075077	-0.32563
C	0.684376	3.305209	-1.66099
H	2.979116	0.000359	4.584215
C	0.297441	5.1043	3.254575
H	1.36656	4.560467	1.448983
C	-1.29411	3.469742	4.090523
H	-1.52526	1.661866	2.905141
C	3.415606	3.842993	-1.26538
H	3.231764	2.675552	0.550755
C	1.392705	4.080805	-2.59246
H	-0.37647	3.086532	-1.82338
C	-0.65431	4.715729	4.210735
H	0.795228	6.077431	3.337152
H	-2.04518	3.162152	4.827117
C	2.757299	4.349645	-2.39845
H	4.483171	4.03634	-1.11567
H	0.873689	4.468901	-3.47603
H	-0.90213	5.384334	5.043088
H	3.310698	4.946705	-3.13222
C	-4.1028	1.009247	0.552169
C	-3.55971	-0.18989	1.287984
C	-5.16803	0.689359	-0.4964
C	-4.56904	2.068986	1.54841
O	-2.93837	-0.06178	2.38786
C	-4.06245	-1.5534	0.884666
C	-4.89274	-0.60019	-1.28045
H	-5.29884	1.545139	-1.18171
H	-6.13269	0.57255	0.044931
H	-5.38738	1.632084	2.157046
H	-3.76941	2.356054	2.245879
H	-4.95469	2.967941	1.040395
C	-4.75469	-1.76517	-0.33475
C	-3.95144	-2.61341	1.81095
H	-5.69064	-0.788	-2.02242
H	-3.94557	-0.48578	-1.84467
C	-5.29714	-3.03155	-0.609
H	-3.4528	-2.41293	2.763588
C	-4.49295	-3.87274	1.525324
H	-5.83317	-3.18902	-1.55307
C	-5.16599	-4.08503	0.309452
H	-4.40183	-4.68599	2.254535
H	-5.60189	-5.06515	0.083324
O	4.371987	-1.97219	3.072188
O	3.962877	-2.12931	0.817571
O	4.477976	0.329857	-1.15884
O	4.587334	-0.00697	-3.42845
C	4.722879	-2.63683	1.884121
C	5.295677	0.433672	-2.29656
F	5.694634	1.71726	-2.46876
F	6.417617	-0.30604	-2.13316
F	6.044129	-2.47405	1.633539
F	4.512437	-3.96854	2.01614

TS$_{\mathrm{RE}}$CS^{dif}_conf1

Symbol	X	Y	Z
P	0.303341	-1.4222	0.60801
C	-1.13326	-0.74507	1.590978
C	1.32677	-2.23136	1.932675
C	-0.47432	-2.87577	-0.24383
C	-2.24064	-0.14324	0.901842
C	-1.11783	-0.7785	3.002974
C	2.307892	-1.44644	2.579093
C	1.182329	-3.58424	2.306918
C	-1.59375	-3.55674	0.285855
C	0.101991	-3.33437	-1.44711
C	-2.40135	-0.10084	-0.58323
C	-3.25873	0.346176	1.717243
C	-2.15824	-0.25189	3.798908
H	-0.26881	-1.24317	3.510679
C	3.10886	-1.99436	3.593393
H	2.454576	-0.40296	2.274712
C	1.994329	-4.1343	3.312597
H	0.436788	-4.2125	1.809184
C	-2.12281	-4.67576	-0.37619
H	-2.05866	-3.2057	1.213439
C	-0.42724	-4.45785	-2.1023
H	0.961932	-2.80977	-1.87382
C	-1.63527	0.695461	-1.49686
C	-3.38404	-0.9034	-1.16827
C	-3.22371	0.306794	3.111638
H	-2.127	-0.2919	4.890791
C	2.955009	-3.3417	3.961096
H	3.864032	-1.36664	4.078225
H	1.873592	-5.18839	3.588207
C	-1.53867	-5.13047	-1.57038
H	-2.99782	-5.18717	0.03907
H	0.033112	-4.80222	-3.03516
P	-0.24777	1.798765	-0.93019
C	-1.83026	0.543583	-2.89101
C	-3.58745	-1.0074	-2.54319
H	3.587323	-3.7744	4.744847
H	-1.95474	-6.00293	-2.08697
C	-0.34798	3.236705	-2.12127
C	-0.87259	2.650173	0.602424
C	-2.80547	-0.30795	-3.44988
H	-1.20879	1.127638	-3.57597
C	-1.5335	3.648083	-2.77387
C	0.824208	4.007055	-2.28917
C	-2.08144	3.377657	0.640083
C	-0.04706	2.633205	1.744232
H	-2.94106	-0.40143	-4.53039
C	-1.53982	4.790953	-3.58841
H	-2.45712	3.071502	-2.65773
C	0.808838	5.154019	-3.10094
H	1.741963	3.712615	-1.76519
C	-2.4612	4.062875	1.803258
H	-2.72943	3.413017	-0.24187
C	-0.42449	3.325503	2.906736
H	0.893799	2.072493	1.711699
C	-0.36779	5.54704	-3.75641
H	-2.46722	5.092604	-4.08929
H	1.726891	5.74126	-3.2187
C	-1.63161	4.040285	2.93833
H	-3.40739	4.613867	1.824368
H	0.226929	3.305493	3.787921
H	-0.37538	6.439305	-4.39295
H	-1.92772	4.581382	3.844207
Pd	1.624387	0.162893	-0.70786
C	3.068405	-1.07342	-1.57452
C	3.636689	-2.10767	-0.78164
C	2.812788	-1.36408	-2.94154
C	3.929289	-3.36515	-1.33175
H	3.791943	-1.94597	0.289717
C	3.112134	-2.62546	-3.48908
H	2.354021	-0.60544	-3.58405
C	3.682057	-3.63089	-2.69182
H	4.337673	-4.15108	-0.6846
H	2.894157	-2.81685	-4.54717
H	3.925382	-4.6099	-3.11946
C	5.351916	0.417194	-1.5337
C	3.867859	0.871058	-1.49029
C	5.971677	-0.14262	-0.24271
H	5.493967	-0.30434	-2.35807
H	5.915054	1.333181	-1.81996
C	3.657928	1.760515	-0.28656
C	3.551518	1.588438	-2.80552
C	5.479567	0.52944	1.021935
H	7.073243	-0.04533	-0.30343
H	5.777035	-1.22772	-0.17815
O	3.073464	2.867206	-0.34654
C	4.365895	1.403389	0.99661
H	4.162884	2.510633	-2.86987
H	2.49661	1.885328	-2.89661
H	3.818645	0.955255	-3.66793
C	6.147207	0.318226	2.24655
C	3.964492	2.063984	2.180617
H	7.022315	-0.34374	2.268184

C	5.724842	0.95228	3.422692
H	3.137539	2.777954	2.109975
C	4.62522	1.832491	3.390647
H	6.264869	0.778725	4.360951
H	4.302125	2.345906	4.30333
O	-4.37986	0.8901	3.611616
O	-4.43965	0.959652	1.316074
O	-4.29077	-1.73377	-0.51851
O	-4.62694	-1.89588	-2.78508
C	-5.13908	1.275704	2.492622
F	-5.39575	2.605398	2.539963
F	-6.33805	0.646222	2.505832
C	-5.08622	-2.3107	-1.52309
F	-6.38126	-1.9462	-1.35676
F	-5.04675	-3.6608	-1.4338

TS$_{RE}$CS^{dif}_conf2

Symbol	X	Y	Z
P	0.189895	1.733291	0.788391
C	1.480498	0.639657	1.56806
C	-0.27969	2.853685	2.196078
C	1.205801	2.846785	-0.28859
C	2.233738	-0.25338	0.733777
C	1.704093	0.658869	2.963106
C	-1.16308	2.360667	3.184262
C	0.16689	4.187677	2.294355
C	2.55042	3.162107	0.010073
C	0.596016	3.425519	-1.42191
C	2.161641	-0.33299	-0.7582
C	3.185945	-1.02743	1.398021
C	2.658588	-0.15945	3.604227
H	1.130257	1.35288	3.581895
C	-1.57172	3.172688	4.252787
H	-1.53517	1.332767	3.111019
C	-0.25514	5.004532	3.357336
H	0.843611	4.593854	1.536548
C	3.27285	4.036998	-0.81582
H	3.035412	2.717383	0.885686
C	1.321202	4.307961	-2.23892
H	-0.44419	3.186355	-1.66468
C	1.086766	-0.87584	-1.54175
C	3.281893	0.077008	-1.48426
C	3.391998	-0.99499	2.777636
H	2.819968	-0.12069	4.684574
C	-1.12085	4.501212	4.340223
H	-2.25191	2.770183	5.012024
H	0.098105	6.040487	3.413944
C	2.658619	4.613467	-1.94072
H	4.318814	4.262652	-0.58243
H	0.834685	4.75257	-3.11424
P	-0.50046	-1.45673	-0.72915
C	1.206661	-0.93171	-2.94796
C	3.378842	0.012632	-2.87394
H	-1.44787	5.140448	5.167909
H	3.224998	5.295632	-2.58486
C	-1.26589	-2.48629	-2.07136
C	0.114924	-2.74396	0.455372
C	2.346948	-0.48668	-3.65081
H	0.384814	-1.3562	-3.52973
C	-1.14688	-3.89206	-2.13311
C	-2.01153	-1.82051	-3.06955
C	1.254713	-3.53497	0.187894
C	-0.62008	-2.98	1.634549
H	2.413388	-0.54837	-4.74001
C	-1.73839	-4.60857	-3.18454
H	-0.59305	-4.43122	-1.35902
C	-2.58826	-2.53904	-4.12867
H	-2.14632	-0.73607	-3.01356
C	1.651178	-4.53688	1.08695
H	1.83895	-3.36325	-0.72264
C	-0.2253	-3.98745	2.529517
H	-1.51523	-2.3868	1.844397
C	-2.45342	-3.93436	-4.18881
H	-1.63734	-5.69951	-3.2183
H	-3.16725	-2.00463	-4.88935
C	0.911161	-4.76555	2.259563
H	2.544039	-5.13353	0.873803
H	-0.81174	-4.16243	3.438351
H	-2.914	-4.49711	-5.00883
H	1.22286	-5.54785	2.960891
Pd	-1.74258	0.578217	-0.14846
C	-2.81732	2.292691	-0.61227
C	-2.90517	3.466741	0.166781
C	-2.88257	2.414734	-2.02471
C	-2.99463	4.728563	-0.44739
H	-2.87514	3.406752	1.260254
C	-2.95409	3.679408	-2.63491
H	-2.92583	1.511067	-2.64461
C	-3.00764	4.843955	-1.84854
H	-3.04365	5.628505	0.178028
H	-2.99453	3.749926	-3.72906
H	-3.08136	5.829539	-2.32199
C	-4.47388	0.755435	1.526542
C	-4.31563	0.724673	0.007236
C	-3.71153	-0.35801	2.256609
H	-4.18512	1.746652	1.922973
H	-5.55819	0.64665	1.750373
C	-4.20368	-0.62281	-0.63342
C	-5.4066	1.545219	-0.68498
C	-4.04275	-1.71336	1.681873
H	-3.90761	-0.32666	3.344891
H	-2.62108	-0.17579	2.106405
O	-4.39043	-0.77598	-1.86454
C	-4.16338	-1.8326	0.273367
H	-6.38123	1.058929	-0.4669
H	-5.27857	1.545904	-1.77566
H	-5.44637	2.583124	-0.31566
C	-4.19334	-2.85774	2.484886
C	-4.40528	-3.09803	-0.29975
H	-4.12042	-2.76004	3.575994
C	-4.43564	-4.11508	1.906125
H	-4.50567	-3.15115	-1.3884
C	-4.53144	-4.23534	0.508783
H	-4.55544	-4.99717	2.545928
H	-4.71665	-5.21446	0.052341
O	4.404403	-1.88447	3.10848
O	4.071546	-1.9402	0.836599
O	4.465207	0.604175	-0.97861
O	4.618122	0.497965	-3.2679
C	4.843844	-2.44534	1.896348
F	4.743097	-3.79543	1.945824
F	6.151927	-2.1585	1.698133
C	5.296989	0.842161	-2.08619
F	6.436514	0.118766	-1.97632
F	5.663228	2.145657	-2.12293

References

1. Gaussian 09, Revision B.01, M. J. Frisch, G. W. Trucks, H. B. Schlegel, G. E. Scuseria, M. A. Robb, J. R. Cheeseman, G. Scalmani, V. Barone, G. A. Petersson, H. Nakatsuji, X. Li, M. Caricato, A. Marenich, J. Bloino, B. G. Janesko, R. Gomperts, B. Mennucci, H. P. Hratchian, J. V. Ortiz, A. F. Izmaylov, J. L. Sonnenberg, D. Williams-Young, F. Ding, F. Lipparini, F. Egidi, J. Goings, B. Peng, A. Petrone, T. Henderson, D. Ranasinghe, V. G. Zakrzewski, J. Gao, N. Rega, G. Zheng, W. Liang, M. Hada, M. Ehara, K. Toyota, R. Fukuda, J. Hasegawa, M. Ishida, T. Nakajima, Y. Honda, O. Kitao, H. Nakai, T. Vreven, K. Throssell, J. A. Montgomery, Jr., J. E. Peralta, F. Ogliaro, M. Bearpark, J. J. Heyd, E. Brothers, K. N. Kudin, V. N. Staroverov, T. Keith, R. Kobayashi, J. Normand, K. Raghavachari, A. Rendell, J. C. Burant, S. S. Iyengar, J. Tomasi, M. Cossi, J. M. Millam, M. Klene, C. Adamo, R. Cammi, J. W. Ochterski, R. L. Martin, K. Morokuma, O. Farkas, J. B. Foresman, and D. J. Fox, Gaussian, Inc., Wallingford CT, 2010.
2. Cramer, C. J.; Truhlar D. G. Density functional theory for transition metals and transition metal chemistry. *Phys. Chem. Chem. Phys.*, **2009**, *11*, 10757.
3. CYLview, 1.0b; Legault, C. Y., Université de Sherbrooke, 2009 (http://www.cylview.org)
4. Culkin, D. A.; Hartwig, J. F. Carbon−Carbon Bond-Forming Reductive Elimination from Arylpalladium Complexes Containing Functionalized Alkyl Groups. Influence of Ligand Steric and Electronic Properties on Structure, Stability, and Reactivity. *Organometallics* **2004**, *23*, 3398.
